# Ultranarrow nanochannels in a staggered two-dimensional polymer membrane enhance electric double-layer coverage for osmotic energy harvesting

**DOI:** 10.1038/s41467-026-74696-4

**Published:** 2026-06-19

**Authors:** Feng Ni, Ye Yang, Shuangjie Zhao, Mahabir Prasad, Naveen Goyal, Xusheng Yang, Dongxu Wang, Jianjun Zhang, Mike Hambsch, Miroslav Polozij, Stefan C. B. Mannsfeld, Ute Kaiser, Grégory F. Schneider, Thomas D. Kühne, Thomas Heine, Zhiyong Wang, Xinliang Feng

**Affiliations:** 1 Max Planck Institute of Microstructure Physics, Halle (Saale), Germany; 2 Center for Advancing Electronics Dresden and Faculty of Chemistry and Food Chemistry, Technische Universität Dresden, Dresden, Germany; 3 Center for Advanced Systems Understanding, Görlitz, Germany; 4 Helmholtz-Zentrum Dresden-Rossendorf, Dresden, Germany; 5 Central Facility of Electron Microscopy, Electron Microscopy Group of Materials Science, Ulm University, Ulm, Germany; 6 Faculty of Electrical and Computer Engineering and Center for Advancing Electronics Dresden (cfaed), Technische Universität Dresden, Dresden, Germany; 7 Institute for Quantum Optics (IQO) and Centre for Integrated Quantum Science and Technology (IQST), Ulm University, Ulm, Germany; 8 Leiden Institute of Chemistry, Leiden University, Leiden, The Netherlands; 9 Institute of Artificial Intelligence, Chair of Computational System Sciences, Technische Universität Dresden, Dresden, Germany

**Keywords:** Porous materials, Devices for energy harvesting, Coordination polymers

## Abstract

Two-dimensional framework membranes (2DFMs) hold great promise for sustainable energy-harvesting technologies, yet their performance is often limited by low electric double-layer (EDL) coverage (*ƞ*_*EDL*_) arising from large channels and/or low charge densities. Here, we report an ultrathin ( ~ 50 nm), fully crystalline, ABC-stacked viologen-incorporated 2D polymer membrane (sV2DP) featuring vertically aligned triangular nanochannels (*D*_*eff*_ = 1.36 nm) densely decorated with pyridinium sites ( + 22.4 mC m^−2^). Compared with its non-staggered AA-stacked analogue, sV2DP exhibits a 3.2-fold enhancement in *ƞ*_*EDL*_ under a 50-fold KCl gradient, combining high anionic selectivity (*t*_− _= 0.85) with remarkable selective current density (14.6 kA m^−2^). Simulations reveal that spirally arranged charges generate a unique “screw-like” anion migration pathway, significantly enhancing transmembrane efficiency relative to non-staggered 2DP analogues. When integrated into micro-aperture osmotic power generators, the sV2DP membrane delivered a peak power density of 243 W m^−2^ under a 50-fold NaCl gradient, placing it among the highest-performing systems.

## Abstract (web summary)

Two-dimensional framework membranes are promising for ion-selective transport and osmotic energy harvesting, but limited electric double-layer coverage constrains their performance. Here the authors report a charged two-dimensional polymer membrane with enhanced electric double-layer coverage through interlayer stacking engineering, enabling highly efficient osmotic power generation.

## Introduction

**Fig. 1:**
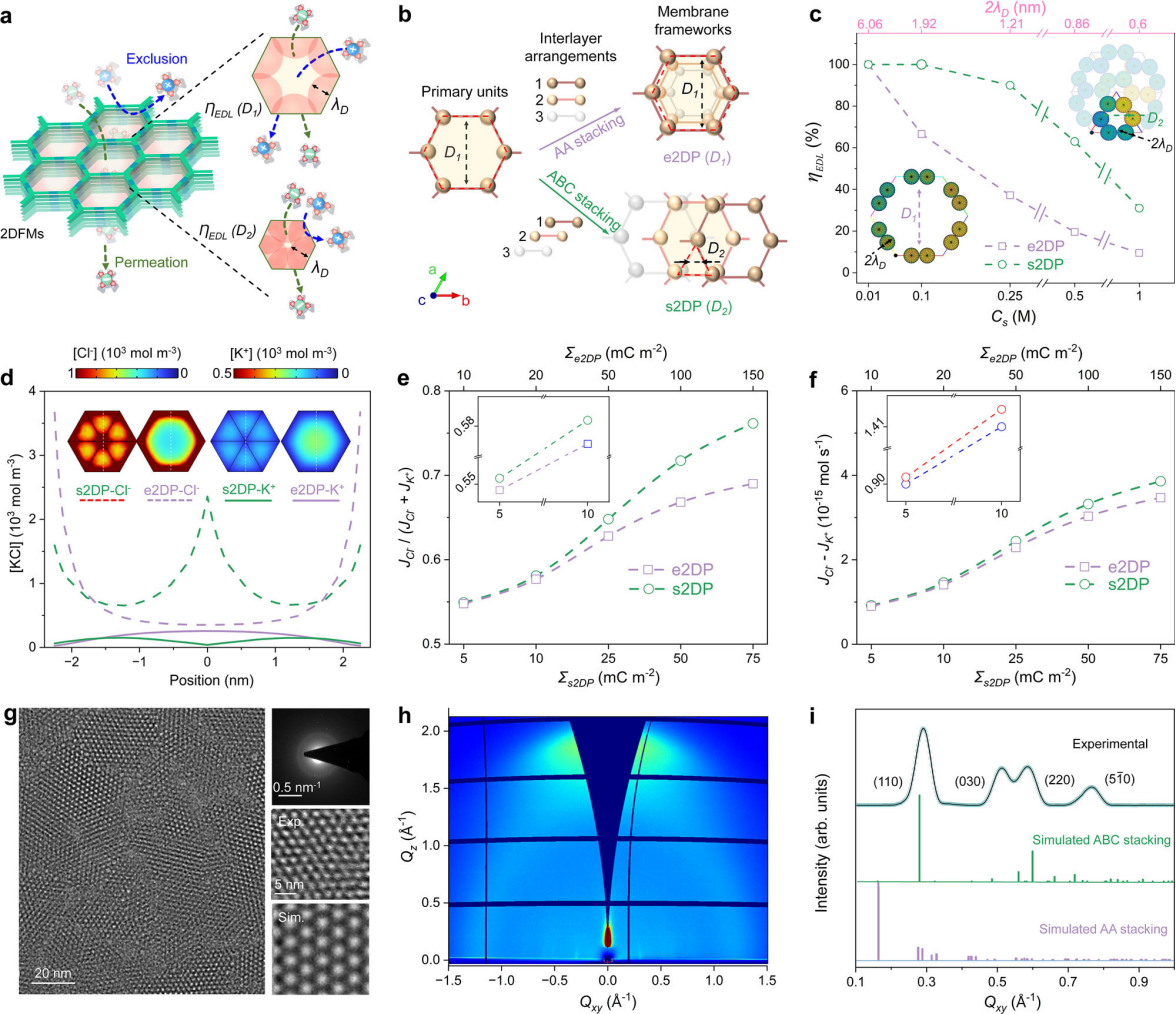
Intensified electrostatic effects in confined nanochannels. **a** Schematic of anion-selective transport through 1D nanochannels within 2DFMs. Green spheres represent hydrated anions, and blue spheres represent hydrated cations. Right inset: comparison of *ƞ*_*EDL*_ in nanochannels with different characteristic sizes (*D*_*1*_ > *D*_*2*_). Red regions denote EDL, whose spatial range is defined by ${\lambda }_{D}$. Narrower nanochannels (*D*_*2*_) exhibit higher *ƞ*_*EDL*_ *(D*_*2*_), ensuring more complete EDL overlap and effective cation exclusion, whereas wider nanochannels (*D*_*1*_) contain larger non-EDL regions that allow cation leakage, thus diminishing anionic selectivity. **b** Regulating the effective channel size (*D*) through interlayer engineering: ABC-stacked s2DP transforms large intrinsic hexagonal pores into confined triangular subchannels along the ***c***-axis, whereas eclipsed e2DP retains the original in-plane pore size (*D*_*1*_). **c** Calculated *ƞ*_*EDL*_ values as a function of solution concentrations (*C*_*s*_), with corresponding${\,2\lambda }_{D}$ values shown. Inset: EDL extents in 0.5 M electrolyte within (left) e2DP channels and (right) s2DP subchannels. Crystallographic axis orientation as in (**b**). **d** Radial ion concentration distributions (along dashed line in inset). Inset: corresponding ion concentration maps under a 0.5/0.01 M KCl gradient. **e**, **f** Ion flux in 2DP models with varying *Σ*: **e** Cl^−^ selective ratio (*J*_*Cl*_^*−*^ / (*J*_*Cl*_^*−*^ + *J*_*K*_^*+*^)) versus *Σ*; **f** Net Cl^-^ flux (*J*_*Cl*_^*−*^- *J*_*K*_^*+*^) versus *Σ*. **g** HRTEM image of a sV2DP membrane. Insets: SAED pattern, enlarged HRTEM, and simulated TEM (bottom). **h** 2D GIWAXS pattern of the sV2DP membrane. **i** Experimental and simulated in-plane projections near *Q*_*z*_ = 0, suggesting the ABC-stacked interlayer architecture of the sV2DP membrane.

Two-dimensional framework membranes (2DFMs), including covalent organic frameworks (COFs)^1,2^, metal-organic frameworks (MOFs)^3,4^, and 2D polymers (2DPs)^5–7^, have emerged as transformative platforms for next-generation sustainable technologies, with applications in water desalination^8,9^, resource recovery^10,11^, and energy harvesting/storage devices^12–16^. Leveraging their nanometer-scale pore structures and functionalized surfaces, 2DFMs confine ion transport within one-dimensional (1D) nanochannels, beyond the stochastic Brownian diffusion in conventional polymeric membranes and intercalation-assisted transport in laminar membranes^17,18^. In thin 2DFMs, typically <1 µm^19^, where transport pathways are short, ion transport is primarily governed by electrostatic interactions across the electric double layer (EDL)^20,21^, manifesting the Gibbs-Donnan effect^22^. Within the spatial extent of the EDL, characterized by the Debye length (*λ*_*D*_), fixed surface charges in 2DFMs create a unipolar ionic environment^20,23^. This electrostatic asymmetry permits counterion permeation while excluding co-ions, thereby enabling effective ion separation (Fig. 1a)^24^. In this context, two key channel parameters: the effective channel size (*D*_*eff*_) and surface charge density (*Σ*), dictate the extent and overlap degree of the resulting EDL^25^. To quantify the interplay between electrostatic screening and channel geometry in a structure-aware yet simple manner, we propose an EDL coverage efficiency (*ƞ*_*EDL*_), a dimensionless, first-order design metric, defined as:

1 $${{\eta }}_{{EDL}}=\frac{\rho \pi {{{\lambda }}_{D}}^{2}}{2f(D)}$$

where *ρ* represents the number of charge sites per projected area (reflecting *Σ*) and *f(D)* denotes the effective cross-sectional area available for a given *D*. Conceptually, *ƞ*_*EDL*_ estimates the fraction of the channel cross-section expected to lie within an EDL-dominated region, and higher *ƞ*_*EDL*_ values imply stronger electrostatic effects and greater ionic selectivity. Note that this is a heuristic scaling descriptor intended for design guidance; its derivation, assumptions, and scope are detailed in Supplementary Note 1. However, most reported 2DFMs either feature relatively wide 1D nanochannels (*D* > 1.5 nm) and/or sparsely distributed charges (low *ρ*), reflecting a practical trade-off between pore narrowing and dense incorporation of ionic sites in crystalline frameworks^26,27^. As a result, the achievable *η*_*EDL*_ is often limited, leading to only moderate permselectivity and constraining overall membrane performance (Supplementary Table 1). Therefore, overcoming these limitations and maximizing *ƞ*_*EDL*_ in 2DFMs is critical for advancing ion-selective transport and energy-harvesting technologies.

Herein, we report an ultrathin (~ 50 nm), staggered viologen-based 2DP (sV2DP) membrane with ultranarrow nanochannels and densely decorated charges, which substantially increase *ƞ*_*EDL*_ and, in turn, enhance anion-selective transport and osmotic energy harvesting. The sV2DP is fully crystalline, exhibiting face-on orientation with through-plane triangular nanochannels (*D*_*eff*_: ~1.36 nm) and strongly positively charged surfaces (effective *Σ*: +22.4 mC m^−2^). This structural configuration promotes extensive EDL coverage, leading to a high inferred chloride transference number (*t*_*Cl*_*−*: ~ 0.85) and an ultrahigh net chloride flux (current output of 14.6 kA m^−2^) under a 50-fold KCl gradient, far exceeding its eclipsed-stacking counterparts (es-V2DP, AA mode). The ABC-stacked sV2DP architecture not only enhances EDL coverage (up to a 3.3-fold enhancement in *ƞ*_*EDL*_) by effectively narrowing the transport cross-section, but also organizes pyridinium charges into a staggered distribution along the transport direction. Molecular dynamics (MD) reveal that this structured electrostatic landscape guides anions into a characteristic “screw-like” migration pathway along the ***c***-axis, in contrast to the more random trajectories observed in non-staggered 2DP analogs. When applied in single micro-aperture osmotic power generators (OPGs), the sV2DP membrane delivers a peak power density (*P*_*out*_) of 243 W m^−2^ under a 50-fold NaCl gradient, which exceeds many previously reported values for 2DFM-based systems (< 100 W m^−2^). Owing to its high mechanical robustness, the ultrathin sV2DP membrane was successfully integrated into an aperture-array OPG with a total aperture area of 6,358.5 μm^2^ and sustained stable power generation over 9 days, suggesting its potential for scaled-area operation and durability. Our results establish the ABC-stacked 2DP architecture as a versatile platform for efficient osmotic energy harvesting and highlight interlayer-stacking engineering as an effective strategy for advancing 2DFM-based osmotic energy-harvesting systems.

## Results

### Intensified electrostatic effects in confined nanochannels

In principle, 2DFMs can adopt different stacking modes, where the interlayer offset critically defines *D* and *Σ*, thereby tuning corresponding *ƞ*_*EDL*_ and offering new opportunities to improve their ion-selective transport^28^. Compared to the common AA-stacked mode in 2DFMs, the ABC-stacked arrangement reduces the effective channel size and brings intrinsic charges into closer proximity, thereby intensifying electrostatic effects in confined nanochannels. To explore this concept, we constructed two theoretical 2DP models based on a hexagonally arranged unit cell (intrinsic *D* = 4.4 nm) bearing two skeletal charges per wall, resulting in a high charge density (*ρ* = 4.11)^29^. In the eclipsed-stacked configuration (AA-stacked mode, e2DP), its channel geometry directly mirrors the primary units, preserving 4.4 nm channels in the resulting frameworks. In contrast, a staggered interlayer arrangement (ABC-stacked mode, s2DP) generates 1D triangular nanochannels along the ***c***-axis with a drastically reduced effective size (*D*_*2*_ = 1.36 nm) (Fig. 1b and Supplementary Fig. 1). This architecture shift imposes stronger geometric confinement and topological alignment, thereby enhancing the electrostatic environments along the transport pathways. As a result, s2DP exhibits substantially increased *ƞ*_*EDL*_ within the channels (inset of Fig. 1c). Quantitative analysis reveals a 1.5 to 3.3-fold enhancement across 0.1–1 M electrolytes (Fig. 1c and Supplementary Fig. 2). Notably, the most pronounced enhancement occurs at higher ionic strengths, where the shortened *λ*_*D*_ typically suppresses EDL extent in conventional framework channels. Such intensified electrostatics in the ABC-stacked structure promote stronger ion partitioning for enhanced ion selectivity, while simultaneously activating additional surface transport pathways for boosted net counterion permeability^30–32^. To elucidate these effects, Poisson-Nernst-Planck (PNP) simulations are performed with the equivalent architectures (Supplementary Fig. 3). The results demonstrate that s2DP consistently shows stronger co-ion (K^+^) depletion and counterion (Cl^-^) enrichment than e2DP across a range of gradients (inset of Fig. 1d and Supplementary Fig. 4). This indicates that the ultranarrow s2DP channels impose stronger anion selectivity than their e2DP counterparts. By contrast, e2DP displays nearly equal K^+^/Cl^−^ distributions at the channel center, indicative of bulk-like ion transport due to weakened electrostatic screening. Under a 0.5/0.01 M KCl gradient, Cl^-^ enrichment at the s2DP channel center rises by 81%, while K^+^ penetration decreases by 39.6% relative to e2DP, confirming enhanced anion-selective transport (Supplementary Fig. 5). This electrostatic advantage further amplifies with increasing surface charge density *Σ*, as evidenced by elevated ion partitioning coefficients (*J*_*Cl*_^*−*^ */* (*J*_*Cl*_^*−*^ *+ J*_*K*_^*+*^)) and higher net counterion flux (*J*_*Cl*_^*−*^ *− J*_*K*_^*+*^) (Fig. 1e, f and Supplementary Fig. 6). Collectively, these features translate into improved open-circuit voltage (*V*_*oc*_) and short-circuit current (*I*_*sc*_) in s2DP relative to e2DP, suggesting that intensified electrostatic effects in confined nanochannels boost anion-selective transport (Supplementary Note 2).

### Synthesis and structural characterization of sV2DP membrane

To experimentally realize these theoretical principles, we synthesized 50-nm-thick viologen-based 2DP (V2DP) membranes with AA and ABC interlayer stacking, denoted as es-V2DP and sV2DP, respectively, using the surfactant-monolayer-assisted interfacial synthesis (SMAIS) method (Supplementary Figs. 7 and 8)^33–35^. Specifically, the sV2DP membrane was obtained via polycondensation between 1,1’-bis(4-formylphenyl)-[4,4’-bipyridine]−1,1’-diium dichloride and 2,2’,2”-(benzene-1,3,5-triyl)triacetonitrile (Supplementary Figs. 9–11). High-resolution transmission electron microscopy (HRTEM) confirms that the as-synthesized sV2DP membrane is fully crystalline, exhibiting a periodic hexagonal lattice (Fig. 1g). The corresponding selected-area electron diffraction (SAED) pattern (Fig. 1g, top inset) reveals a diffraction ring at 0.45 nm⁻¹, corresponding to a *d*-spacing of ~2.2 nm. An enlarged HRTEM image (Fig. 1g, middle inset) closely matches the simulated image of the ABC-stacked configuration (Fig. 1g, bottom inset). To assess the macroscopic layer orientation and stacking characteristics, we further conducted grazing-incidence wide-angle X-ray scattering (GIWAXS) measurements. A prominent scattering arc at *Q*_*z*_ = 1.90 Å^−1^ indicates an interlayer spacing of 3.3 Å and confirms the preferential face-on layer orientation of sV2DP, thus providing vertical alignment of its 1D nanochannels (Fig. 1h). In-plane diffraction peaks at *Q*_*xy*_ = 0.29, 0.51, 0.59, and 0.77 Å^−1^ are assigned to the (110), (030), (220) and (5$\bar{1}$0) Bragg reflections of the hexagonal unit cell, respectively, which align well with the simulated pattern of the ABC-stacked structure (Fig. 1i). For comparison, we further synthesized AA-stacked es-V2DP membranes via 2D polycondensation between 1,1′-bis(4-aminophenyl)-[4,4′-bipyridine]−1,1’-diium chloride and 2,4,6-trihydroxybenzene-1,3,5-tricarbaldehyde (Supplementary Fig. 12)^29,36^. The es-V2DP membrane shares nearly identical lattice parameters, backbone composition, and charge density with sV2DP but differs fundamentally in its stacking mode. In sV2DP, steric hindrance from bulky acetonitrile substituents enforces a staggered ABC configuration, whereas the absence of such constraints in es-V2DP promotes an eclipsed AA stacking arrangement, resulting in significantly wider nanochannels (~ 4.4 nm) (Supplementary Fig. 13)^28,37^. To ensure a rigorous comparison of ion transport, both membranes were prepared with comparable thicknesses (~ 50 nm) and exhibited similar crystallinity (Supplementary Fig. 14).

### Selective ion transport in sV2DP membrane

**Fig. 2:**
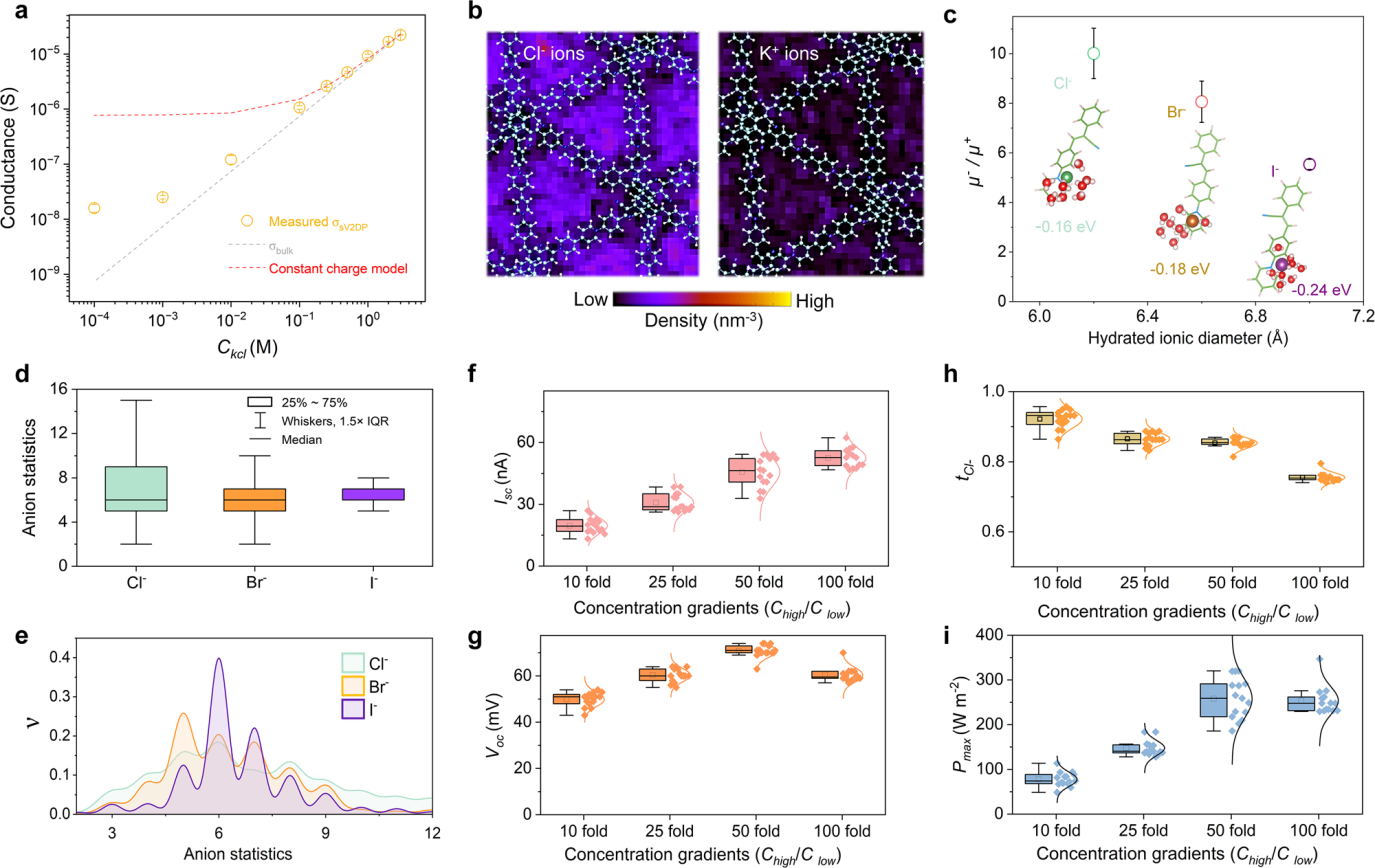
Selective ion transport in sV2DP membrane. **a** Transmembrane conductance of the sV2DP membrane as a function of KCl concentration (*C*_*KCl*_). The red curve shows the theoretical fit using a constant-charge model (effective $\varSigma$ = +22.4 mC m^−2^ in 0.25 M KCl). Data are mean ± SD from *n* = 6 independent measurements. **b** Simulated 2D ion density maps showing the spatial distributions of Cl^−^ and K^+^ within the sV2DP nanochannels, demonstrating pronounced enrichment of Cl^-^ and exclusion of K^+^, indicative of strong anion selectivity. **c** Experimentally measured *µ⁻/µ⁺* values across the sV2DP membrane under tenfold KX (X = Cl, Br, I) gradients. Data are mean ± SD. Inset: calculated binding energies between each halide ion and the positively charged pyridinium sites in sV2DP. **d** Time-resolved statistics of halide-ion residence within 5.5 Å of individual pyridinium sites during transmembrane transport. Data are mean ± SD. **e** Frequency distributions of halide-ion dwell times corresponding to (**d**). Performance metrics extracted under varied KCl gradients: **f** *I*_*sc*,_ **g** *V*_*oc*_, **h** inferred *t*_*Cl*_*−*, and **i** *P*_*max*_. Individual data points are shown alongside the box plots, with half-violin plots indicating the data distribution. Boxes indicate the 25th–75th percentiles, center lines indicate the median, whiskers extend to values within 1.5× the interquartile range, and squares indicate mean values. Data were obtained from *n* = 15 independent measurements.

For electrochemical measurements, the as-synthesized sV2DP membrane was transferred and sealed over a 2 μm-diameter aperture on a SiN_x_ substrate (Supplementary Fig. 15). The sV2DP membrane exhibits characteristics consistent with charged nanochannels, as indicated by the asymmetric *I*–*V* response (Supplementary Fig. 16)^29,38^. Further investigations into *Cₛ*-dependent ion conductance reveal a strong deviation of sV2DP conductance (*σ*_*sV2DP*_) from the linear bulk trend (*σ*_*bulk*_) at low ionic strength, with *σ*_*sV2DP*_ values several orders of magnitude higher (Fig. 2a and Supplementary Fig. 17). This conductance saturation indicates that mobile counterions near the charged channel walls dominate transport under dilute conditions^31^. By fitting a constant-charge model, an effective (or apparent) fitted surface charge density (*Σ*) of sV2DP is further estimated to be +22.4 mC m^−2^ (Supplementary Note 3), significantly higher than those of typical 2DFMs (<10 mC m^−2^)^38–42^. Such a high charge density promotes the surface conduction dominated by counterions across the sV2DP nanochannels, effectively compensating for the increased energy barrier associated with ultranarrow confinement (Supplementary Note 2). Consequently, *σ*_*sV2DP*_ reaches 1.06 µS at 0.1 M KCl, approaching that of monolayer 2DP membranes (~ 1.73 µS)^13^.

Next, we assessed the permselectivity of sV2DP under KCl gradients. Ion selectivity arises from densely distributed viologen moieties (pyridinium sites) along the nanochannel walls, which act as ion-transport sites by leveraging strong positive charges to facilitate anion transport. This is supported by 2D ion density maps and energy barrier profiles for Cl^-^ and K^+^ (Fig. 2b and Supplementary Fig. 18). When subjected to a 10-fold KCl gradient, sV2DP generates a pronounced *V*_*oc*_ of 50 mV and *I*_*sc*_ of 17.82 nA, in sharp contrast to near-zero values from the bare substrate, confirming its strong Cl^−^ selectivity (Supplementary Fig. 19). Similar behavior was observed for Br^−^ and I^−^, demonstrating enhanced anion transport over K^+^ transport (Supplementary Fig. 20). Relative mobilities (*µ*^*−*^*/µ⁺*) in sV2DP correlate inversely with the hydrated ionic diameters, Cl^-^ (6.2 Å) > Br^−^ (6.6 Å) > I^−^ (7.0 Å) (Fig. 2c and Supplementary Table 2). This trend is also consistent with the binding energy sequence for pyridinium sites (inset of Fig. 2c), where larger anions encounter greater steric and electrostatic barriers, thereby reducing mobility. Trajectory analysis of anions within ~5.5 Å of pyridinium sites reveals distinct dynamics: Cl^-^ displays a broad distribution of coordination numbers, indicative of frequent binding-release events, resulting in short residence times and high mobility (Fig. 2d). In contrast, I^−^ maintains a nearly constant coordination number (~ 6), suggesting stronger, more persistent interactions and reduced mobility (Fig. 2e). These observations align with calculated effective diffusion coefficients (*D*_*eff*_) (Supplementary Fig. 21) and experimental ionic selectivity and power output (Supplementary Fig. 22).

Further increasing the KCl gradients across the sV2DP membrane led to a continuous rise in *I*_*sc*_, reaching up to 52.59 nA under a 100-fold gradient (Fig. 2f and Supplementary Fig. 24). When normalized by the effective membrane area, the corresponding areal current densities ranged from 6.24 to 17.53 kA m^−2^, exceeding many reported values for 2DFMs (0.4–6 kA m^−2^)^29,42,43^. This high performance can be attributed to high ionic selectivity and low ionic resistance within the sV2DP membranes. Meanwhile, *V*_*oc*_ follows a volcano-shaped dependence on the gradient, peaking at 70.7 mV under a 50-fold gradient before showing a slight decline at 100-fold gradient (Fig. 2g). Based on measured *V*_*oc*_ values, the sV2DP membrane maintains the inferred Cl^-^ selectivity coefficients (*S*_*Cl*_*−*) above 50% across all tested KCl gradients, with an inferred *t*^*−*^ of up to 0.75 even under a 100-fold gradient (Fig. 2h and Supplementary Fig. 25). These features enable an optimal balance between anion selectivity and permeability in the sV2DP membrane, delivering a maximum power density (*P*_*max*_) of ~ 253.69 W m^−2^ under a 100-fold KCl gradient (Fig. 2i).

To elucidate the dominant ion-transport pathway, we performed a series of leakage-sensitive control experiments, including an intentional nanoscale notch introduced by focused ion beam (FIB), thickness-dependent measurements, post-measurement imaging, and pH-dependent selectivity tests (Supplementary Figs. 26–29). The intentional-defect experiment shows that introducing a nanoscale notch leads to transport behavior approaching that of the bare substrate, accompanied by a pronounced loss of ion selectivity, consistent with leakage-dominated transport (Supplementary Fig. 26). In contrast, intact membranes exhibit significantly lower conductance and preserved selectivity. Thickness-dependent measurements further reveal consistent trends in *V*_*oc*_, *I*_*sc*_, and *P*_*max*_ across membranes with different thicknesses, supporting that ion transport is governed by the intrinsic nanochannel structure rather than uncontrolled leakage pathways (Supplementary Fig. 27). In addition, pre- and post-measurement optical and SEM imaging confirm that the membranes remain structurally intact during operation, excluding failure-induced leakage (Supplementary Fig. 28). The pH-dependent selectivity measurements further demonstrate stable ion-selective transport behavior across different conditions (Supplementary Fig. 29). Overall, these results suggest that transport in intact devices is primarily governed by the designed sV2DP nanochannels rather than by leakage pathways.

To gain deeper insight into the role of ultranarrow confinement in sV2DP ion transport, we performed control experiments using es-V2DP (Supplementary Fig. 30). In stark contrast, the es-V2DP membrane exhibits substantially lower *V*_*oc*_ under identical KCl concentration gradients, reflecting a pronounced loss of anion selectivity, especially at elevated ionic strengths (Supplementary Fig. 31). Its inferred *S*_*Cl*_*−* drops to just 21.6% under a 50-fold gradient, less than one-third of the value observed for the sV2DP membrane (Supplementary Fig. 32). These significant reductions are attributed to the wider (4.4 nm), less confined nanochannels in es-V2DP, which diminish EDL coverage and thus weaken electrostatic effects. Furthermore, net Cl^-^ permeability across the es-V2DP membrane is also decreased due to less efficient ion partitioning, leading to lower *I*_*sc*_ and *P*_*max*_ (Supplementary Fig. 33).

### Molecular insights into efficient transmembrane anion transport in sV2DP

**Fig. 3:**
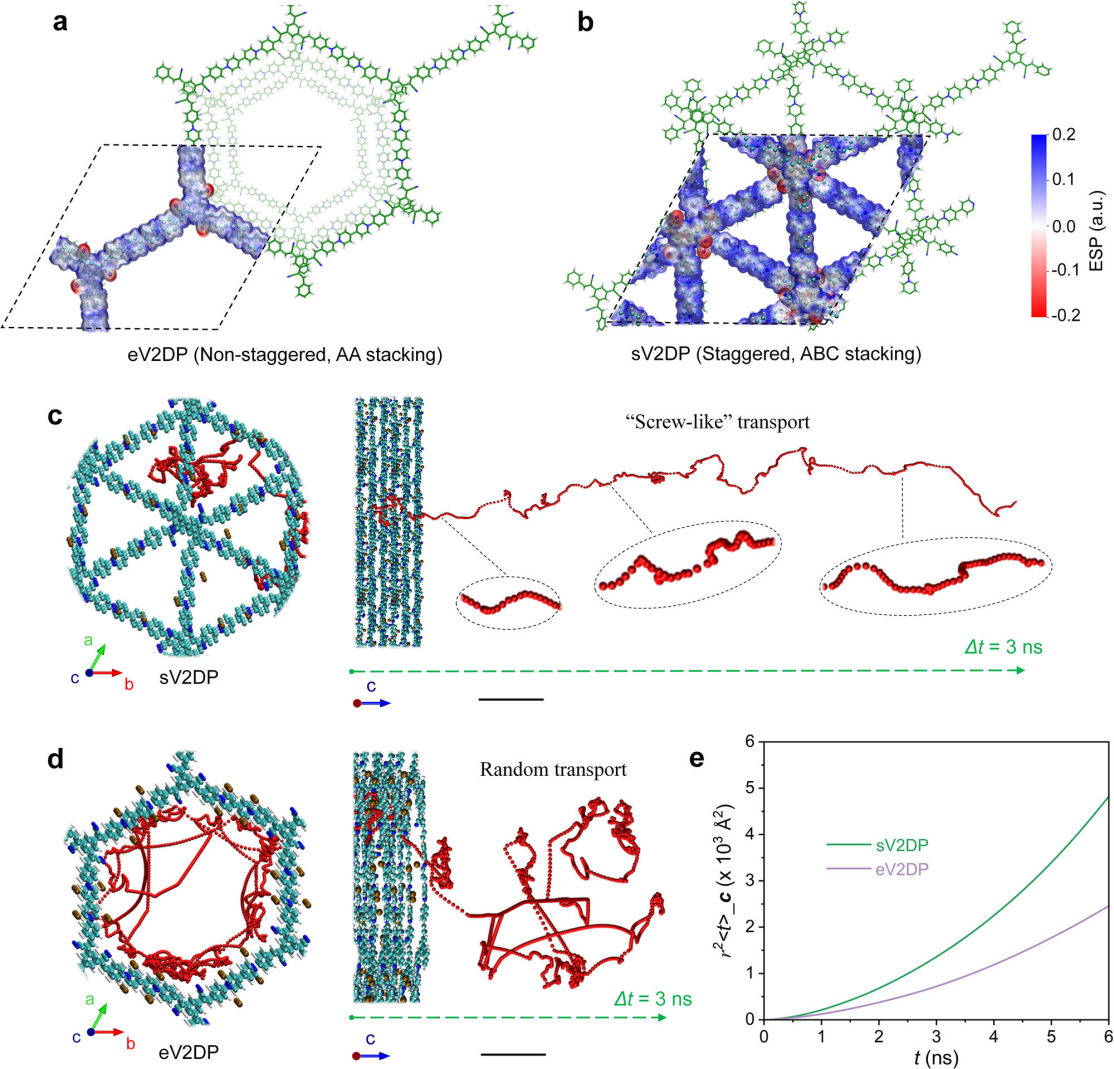
MD simulations for transmembrane anion transport. **a**, **b** Electrostatic surface potential (ESP) maps illustrating the enhanced surface charge effects in **a** AA-stacked eV2DP channels compared to **b** ABC-stacked sV2DP channels. **c** Representative trajectories of individual Cl^-^ions traversing the sV2DP membrane. Left: projection onto the ***ab*** plane; right: projection along the ***c***-axis. Data points were recorded every 0.25 fs over a total timescale of 3 ns. Scale bar: 21.7 Å. Inset: enlarged trajectories of the characteristic “screw-like” transmembrane transport guided by the staggered channel environments. **d** Corresponding Cl^-^ trajectories in the eV2DP membrane, showing increased lateral diffusion and less axial transport, indicative of a more random migration pattern (same sampling interval and scale as sV2DP). Scale bar: 21.7 Å. **e** Time-averaged MSD curves of Cl^-^ ions along the ***c***‑axis (*r*^*2*^ < *t* > _***c***) in sV2DP versus eV2DP over extended time windows, confirming more efficient axial transport in sV2DP.

Ion transport through the 2DP membrane channels is governed not merely by passive, gradient-driven diffusion but also by a series of activated processes shaped by local structural and electrostatic environments^44^. In sV2DP, the interlayer-staggered ABC-stacked architecture results in a denser arrangement of pyridinium sites compared to the eclipsed-stacked eV2DP, thereby intensifying local electrostatic interactions (Fig. 3a, b). This amplified surface charge effect is consistent with the observed high effective *Σ* of the resulting sV2DP membranes. Consequently, a stronger anion partitioning at the sV2DP channel entrance is evident, as confirmed by potential of mean force (PMF) analysis (*ΔE_part*: −3.77 kJ mol^−1^ for sV2DP vs. −3.03 kJ mol^−1^ for eV2DP; Supplementary Fig. 34).

In addition, the ABC stacking in sV2DP gives rise to ultranarrow nanochannels oriented along the ***c***-axis, where pyridinium sites adopt a distinct spiral configuration (Supplementary Fig. 35a, b). This confinement promotes substantial coverage of electrostatic domains (i.e., high *ƞ*_*EDL*_) within the channels, ensuring continuous electrostatic interactions with anions and providing a fast conduction pathway^45,46^. Meanwhile, the spiral distribution of pyridinium charges introduces a pronounced axial electrostatic component that acts as a built-in guiding field, thereby facilitating directional anion migration along the channel axis (Supplementary Fig. 35c, d)^47^. MD simulations support this structure-induced mechanism, revealing that Cl^−^ ions in sV2DP follow a unique “screw-like” migration trajectory along the ***c***-axis (Fig. 3c and Supplementary Fig. 37), indicative of a surface-governed anion diffusion pathway tightly regulated by local electrostatic interactions. Although the experimental comparison between sV2DP and es-V2DP is not a pure stacking-only control because chemistry/linkage differences remain, MD simulations using the corresponding structural models support the view that the distinct channel geometry and spatial distribution of fixed pyridinium charges play a major role in regulating Cl^-^ migration pathways. In contrast, Cl^−^ transport in the non-staggered eV2DP membrane lacks such directional confinement and predominantly undergoes lateral diffusion within the ***ab*** plane, exhibiting a random, bulk-like motion (Fig. 3d). This difference is consistent with insufficient EDL coverage (low *ƞ*_*EDL*_) in the wider eV2DP channels, which leads to isolated electrostatic domains that fail to fully overlap, thereby weakening electrostatic control over ion movement. During a 3 ns simulation, Cl^-^ ions in sV2DP travel nearly twice the axial distance compared to eV2DP. Mean square displacement (MSD, *γ²<t* > ) results further confirm the enhanced transport dynamics along the ***c***-axis over extended time windows (Fig. 3e). The effective Cl^-^ diffusion coefficients along the ***c***-axis and ***ab*** plane (*D*_*eff*_***c***_ and *D*_*eff*_***ab***_) reveal pronounced diffusion anisotropy in sV2DP (*D*_*eff*_***c***_**/***D*_*eff*_***ab***_ = 2.43), significantly higher than the nearly isotropic values (~1) observed in eV2DP (Supplementary Figs. 38 and 39). It should be noted that the connection between simulation and experiment remains primarily qualitative, as the experimentally observed transport metrics (e.g., conductance, selectivity, and power output) can be further influenced by factors such as interfacial polarization, structural defects, and device-level configurations that are not fully captured in the MD models. Overall, these results suggest that, in sV2DP, the coupled geometric confinement and electrostatic landscape promote anion transmembrane diffusion, while the wider, AA-stacked eV2DP channels exhibit weaker electrostatic regulation, resulting in bulk-like ion diffusion and a higher transmembrane energy barrier (Supplementary Figs. 34 and  40).

### Osmotic energy harvesting and scalability demonstration

**Fig. 4:**
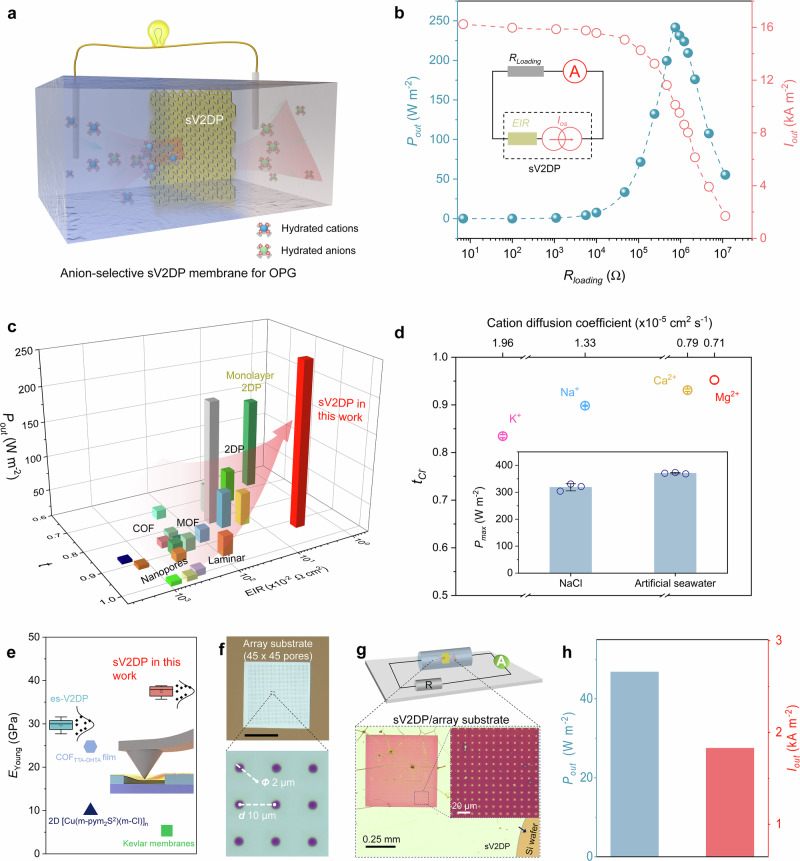
Demonstration of high-performance and scalable osmotic energy harvesting enabled by sV2DP membrane. **a** Schematic illustration of the OPG process based on an anion-selective sV2DP membrane, which facilitates Cl^−^ transport while effectively excluding cations to generate electricity. **b** *P*_*out*_ of the single micro-aperture OPG as a function of external load resistance under a 50-fold NaCl gradient. Inset: Equivalent electrical circuit model showing that the maximum *P*_*out*_ is achieved when the external load resistance matches the system’s *EIR*. **c** Comparison of the peak power density of sV2DP with reported 2DFM-based and other 2D nanofluidic membranes under comparable salinity-gradient conditions. *P*_*out*_ is plotted as a function of the inferred *t* values and *EIR*, reflecting area-normalized ion conductance. **d** Output power density of the sV2DP-based OPG under artificial seawater/river water conditions. Data are mean ± SD from *n* = 3 independent measurements. Inset: comparison of *P*_*max*_ under NaCl and artificial seawater/river water gradients. **e** Young’s modulus comparison: sV2DP membranes exhibit a significantly higher modulus compared to es-V2DP membranes (29.8 ± 1.3 GPa), Kevlar membranes (2.4 GPa)^53^, and most layer-stacked 2D MOF and COF films (typically <30 GPa)^54,55^. Individual data points are shown alongside the box plots, and half-violin plots indicate the data distribution. Boxes indicate the 25th–75th percentiles, center lines indicate the median, and whiskers extend to values within 1.5× the interquartile range, and squares indicate mean values. Data were obtained from *n* = 8 independent measurements. **f** Optical image of an sV2DP membrane integrated onto a substrate with a 45 × 45 aperture array. **g** Enlarged optical/SEM image showing uniform membrane coverage over the aperture-array substrate with minimal wrinkling and no visible cracks. **h** Output power density of the resulting aperture-array OPG under artificial seawater/river water conditions.

We then evaluated osmotic energy harvesting by integrating the resulting sV2DP membrane into a single micro-aperture OPG under a 50-fold NaCl gradient (Fig. 4a). Under this condition, the membrane exhibits a high osmotic potential (*V*_*os*_*)* of 79.8 mV and osmotic current (*I*_*os*_) of 49 nA, respectively (Supplementary Figs. 41 and 42). The inferred *t*_*Cl−*_ reaches ~0.90, higher than that obtained under the same KCl gradient (Supplementary Fig. 43). This enhancement stems from the larger hydrated radius of Na^+^ (~ 7.16 Å) compared to K^+^ (~ 6.62 Å), which amplifies cation exclusion and thus improves anion selectivity. The *P*_*out*_ reaches 243 W m^−2^ at an equivalent internal resistance (EIR) of 0.75 MΩ under the same 50-fold NaCl gradient (Fig. 4b). This exceptional performance results from an optimized combination between high ion selectivity (as indicated by the high *t* values) and efficient ion conduction (reflected by low areal resistances). When benchmarked against reported ion-selective membranes, including 2DFMs (2DPs^13,42^, 2D COFs^48,49^/MOFs^50^), 2D laminar membranes^43,51^ and porous 2D membranes^52^, sV2DP shows competitive performance among these membrane-based OPG systems (Fig. 4c and Supplementary Table 3).

Further enhancement of OPG performance was observed under an artificial seawater/river water gradient, which contains substantial concentrations of divalent cations (e.g., Mg^2+^ and Ca^2+^), compared with pure NaCl conditions (Supplementary Fig. 44). The improved performance is attributed to the larger hydration shells of these divalent cations, which more effectively impede their transport and thereby enhance the inferred Cl^−^ selectivity. Consequently, *P*_*max*_ increased from 318.6 W m^−2^ (in NaCl) to 370.9 W m^−2^ (inset of Fig. 4d and Supplementary Fig. 45), with a corresponding *P*_*out*_ of 323 W m^−2^ under a 0.75-MΩ load (Supplementary Fig. 46). The *P*_*out*_ of the 12.58-µm^2^ device slightly decreased to 218 W m^−2^, and tandem integration in series and parallel configurations successfully amplified the electrical output, achieving 0.97 V and 1.31 μA, respectively (Supplementary Figs. 47 and 48).

To assess the scalability and device-level applicability of the sV2DP membrane in aperture-array OPGs, we characterized its mechanical properties via nanoindentation measurements. As shown in Fig. 4e and Supplementary Fig. 49, sV2DP membranes exhibit a high Young modulus (*E*_*Young*_) value of 37.5 ± 1.2 GPa, higher than that of es-V2DP membranes (29.8 ± 1.3 GPa), Kevlar membranes (2.4 GPa)^53^, and most layer-stacked 2D MOF and COF films (typically less <30 GPa)^54,55^. This mechanical strength offers crucial structural integrity necessary for integration into aperture-array OPGs. Then, sV2DP membranes were transferred onto substrates with a 45 × 45 aperture array, expanding the total effective aperture area to 6358.5 μm^2^ (Fig. 4f). The membrane adhered firmly to the substrate surface with only minimal wrinkling, indicating uniform coverage without visible cracks (Fig. 4g). When operated under an artificial seawater/river water salinity-gradient, the resulting aperture-array OPG delivered a *P*_*out*_ of 46.7 W m^−2^. This value represents competitive performance among reported aperture-array or membrane-based OPGs measured under comparable testing conditions and device configurations (Supplementary Fig. 50 and Table 6).

However, a similar scale-up issue is observed for sV2DP-based aperture-array OPGs^13,56^. We attribute this behavior to two main factors: (i) a nonlinear decline in membrane resistance with effective aperture area, and (ii) the presence of intrinsic grain boundaries and defects in larger-area membranes. Based on a semi-quantitative analysis of resistance partitioning, the decrease in intrinsic membrane resistance (*R*_*mem*_) with increasing aperture area likely enhances the relative contributions of reservoir and interfacial resistances, which may contribute to the observed decline in *P*_*out*_ (Supplementary Fig. 51). In addition, grain boundaries and structural defects are expected to introduce relatively open, less ion-selective transport pathways (e.g., larger-pore-area 7-membered lattice motifs) (Supplementary Fig. 52), thereby reducing the overall permselectivity. Consequently, the device performance shows an approximately exponential decay with increasing effective aperture area in aperture-array OPGs (Supplementary Fig. 53). These interpretations are further supported by our device-level defect-sensitivity test: introducing an intentional single-pore defect causes a dramatic loss of ion selectivity (short-circuit-like behavior) (Supplementary Fig. 54). Despite this scale-up challenge, sV2DP-based aperture-array OPGs maintain stable operation. Under continuous operation under artificial seawater/river water conditions, stable output is maintained over nine days without noticeable degradation (Supplementary Fig. 55). Post-measurement structural, chemical, and device-level characterizations reveal no discernible changes compared to the pre-measurement state (Supplementary Figs. 56–58), confirming the robustness of the membrane. Overall, these results suggest a feasible pathway toward extending the performance of sV2DP membranes to aperture-array OPG formats, although a fully quantitative description of the scaling behavior remains an important subject for future investigation.

## Discussion

In summary, we demonstrate that the ABC-stacked sV2DP membrane with ultranarrow nanochannels and densely distributed pyridinium sites substantially increases *ƞ*_*EDL*_, thereby enhancing anion-selective transport and osmotic energy harvesting. Computational modeling and experimental investigations indicate that its ultranarrow nanochannels (*D*_*eff*_ = 1.36 nm) and strongly charged channel surface (effective *Σ*, +22.4 mC m^−2^) in sV2DP promote extensive *ƞ*_*EDL*_, intensifying the electrostatic effects and boosting anion-selective transport compared with its eclipsed counterpart. As a result, sV2DP achieves a high inferred *t*_*Cl*_*−* of 0.85 and a selective current density of 14.6 kA m^−2^ under a 50-fold KCl gradient. MD simulations further suggest that ultranarrow confinement and spirally arranged pyridinium sites guide anions along a “screw-like” transport pathway with a lower energy barrier, facilitating anion transmembrane migration relative to random diffusion in non-staggered analogs. When integrated into a single micro-aperture OPG, the sV2DP membrane delivers a peak power density of 243 W m^−2^ under a 50-fold NaCl gradient. In an aperture-array OPG with a total aperture area of 6358.5 μm^2^, the membrane sustains a *P*_*out*_ of 46.7 W m^−2^ over nine days under artificial seawater/river water conditions, suggesting its potential for scaled-area operation and long-term durability. It should be noted that residual concentration polarization and device-level resistance contributions may partially limit the measured transport performance, and these effects could be further mitigated through membrane structural asymmetry or system-level optimization, such as stirring or thermal convection, in future studies. This work identifies interlayer-stacking engineering as an effective strategy for regulating charged nanochannels and advancing ion-selective transport in 2DFM-based sustainable energy-harvesting technologies.

## Methods

### General characterization

Optical microscopy (Zeiss) and scanning electron microscopy (SEM, Zeiss Gemini 500) were employed to observe the membrane morphology. HRTEM images were acquired using a JEOL F200 and an image-side Cs-corrected FEI TITAN 80–300 operated at 300 kV. AFM was conducted on a Park FX40 system, and FTIR spectra were obtained using a Bruker Optics ALPHA-E spectrometer with an Attenuated Total Reflectance (ATR) module. For GIWAXS measurements, the membranes were transferred onto Si wafers. Data were collected at the XRD1 beamline at Elettra Sincrotrone Trieste, Italy, with a photon energy of 12.39 keV and a beam diameter of 200 µm. A DECTRIS Pilatus 2 M area detector was positioned 400 mm behind the sample to record the 2D diffraction patterns. The setup was calibrated using a lanthanum hexaboride standard. The beam incidence angle was set at 0.1°, with a 60 s exposure time. Data correction and analysis were carried out using WxDiff software.

### Synthesis of sV2DP membranes via Knoevenagel polycondensation on the water surface

The sV2DP synthesis was carried out following our previously reported procedure^35^. Briefly, a 20 μL solution of sodium (9Z)-octadec-9-en-1-yl sulfate (sodium oleyl sulfate, SOS, 1 mg mL^−1^ in chloroform) was spread evenly over the surface of 50 mL of Milli-Q water in a 60 mL crystallization dish (6 cm diameter) to form a uniform surfactant monolayer. After stabilizing for 30 min, 1 mL of an aqueous solution containing 1’-bis(4-formylphenyl)-[4,4’-bipyridine]-1,1’-diium dichloride (6.9 μmol) and Cs_2_CO_3_ (15.3 mmol) was carefully injected beneath the monolayer. Two hours later, 1 mL of an aqueous solution of 2,2’,2”-(benzene-1,3,5-triyl)triacetonitrile (4.6 μmol) was added. The reaction mixture was maintained at 50 °C for 14 days to complete the polymerization. The resulting sV2DP membrane formed on the water surface was transferred onto the desired substrates, rinsed with ethanol and Milli-Q water, and dried under a gentle nitrogen flow.

### Synthesis of es-V2DP membranes via Schiff-base reaction on the water surface

As described in our previous works^29,36^, a 20 μL solution of SOS (1 mg mL^−1^ in chloroform) was spread onto the surface of 50 mL of Milli-Q water in a 60 mL crystallization dish (6 cm diameter) to form a uniform surfactant monolayer. Subsequently, 1 mL of an aqueous solution of 1,1′-bis(4-aminophenyl)-[4,4′-bipyridine]-1,1’-diium chloride (2.4 µmol) containing trifluoromethanesulfonic acid (TfOH) (3.7 µmol) was gently introduced into the subphase. After 1 h, 1 mL of an aqueous solution of 2,4,6-trihydroxybenzene-1,3,5-tricarbaldehyde (1.6 µmol) was added. The reaction mixture was then left undisturbed at room temperature for 1 week to allow polymerization.

### Ion transport measurements

The membrane sample was prepared as follows. An as-prepared membrane was transferred onto a freestanding 200-nm-thick SiN_x_ film supported by a silicon wafer, covering a 2 μm-diameter aperture (Supplementary Fig. 15a). After drying at room temperature for 1 h, membrane coverage was confirmed by SEM and optical microscopy. To remove residual contaminants, the membrane/substrate assembly was sequentially rinsed with ethanol and Milli-Q water, followed by drying at 80 °C for 1 h to ensure stability and cleanliness.

All ion transport measurements were performed using a custom-built H-cell device (Supplementary Fig. 15b). The membrane/substrate assembly was clamped between two chambers (reservoir volume: 8.0 mL each), allowing contact with aqueous electrolyte solutions (~ 7 mL) on both sides. Standard Ag/AgCl electrodes with 3 M KCl salt bridges (CHI111) were used to record voltage and current signals, minimizing liquid-junction potentials caused by salt concentration differences. The electrode spacing was approximately 25 mm. KCl solutions at pH 6.7 were chosen as electrolytes due to the similar bulk diffusion coefficients of K^+^ and Cl^−^ ions. Electrochemical tests were performed on a CHI 770E workstation to evaluate ion transport and OPG performance. All measurements were conducted at room temperature (25 °C), and I–V scans were recorded at a scan rate of 10 mV s^−1^. Before each I–V measurement, both chambers were rinsed three times with ultrapure water to minimize cross-contamination. The *V*_*oc*_ was then monitored and allowed to stabilize until a steady value was reached before data acquisition, ensuring minimal influence from transient interfacial polarization (Supplementary Fig. 23).

In ion-selective transport experiments, *V*_*oc*_ and *I*_*sc*_ were extracted from the intercepts of the *I–V* curves at zero current and zero voltage, respectively, under varying concentration gradients. The *P*_max_ was calculated as:

2 $${P}_{\max }=\frac{{V}_{{oc}}\times {I}_{{sc}}}{4A}$$

where *A* is the effective membrane area. The conductance was extracted from the ohmic (linear) region of the *I*–*V* curve by performing a linear fit within a narrow bias window of −50 to +50 mV, using:

3 $$G=\frac{{dI}}{{dV}}$$

The ion selectivity coefficient (*S*) was calculated using the following equation^56^:

4 $$S=\frac{{V}_{{oc}}F}{{RT}{{\mathrm{ln}}}\frac{{{\mbox{a}}}_{{\rm{high}}}}{{{\mbox{a}}}_{{\rm{low}}}}}$$

where *a*_*high*_ and *a*_*low*_ are the activities on the concentrated and diluted sides, respectively (*a* = γ c). For a conservative estimate and to facilitate direct comparison with prior reports, the concentration ratio (*C*_*high*_*/C*_*low*_) was used as an approximation of *a*_*high*_/*a*_*low*_ to obtain the inferred *S* values in this work. In addition, the inferred *t*_*-*_ is calculated as^57^:

5 $${t}_{-}=\frac{S+1}{2}$$

The *P*_*out*_ under an external load *R*_*loading*_ is determined using:

6 $${P}_{{out}}=\frac{{{I}_{{out}}}^{2}\times {R}_{{loading}}\,}{A}$$

The mobility ratio (*µ⁻/µ*^*+*^) was estimated from the corresponding *V*_*oc*_ measurements, using the Henderson equation^58^:

7 $$\frac{{\mu }^{-}}{{\mu }^{+}}=-\frac{{z}^{+}{{\mathrm{ln}}}\left({\varDelta} {{\mathrm{C}}}\right)+{{\mathrm{z}}}^{-}{{\mathrm{F{V}}_{{oc}}/{RT}}}}{{z}^{-}{{\mathrm{ln}}}\left(\varDelta {{\mathrm{C}}}\right)+{{\mathrm{z}}}^{+}{{\mathrm{F{V}}_{{oc}}/{RT}}}}$$

where *z*^*+*^ and *z*^*-*^ denote the valences of cations and anions, respectively, and $\varDelta C$ represents the concentration gradient.

### DFT calculations

The sV2DP and eV2DP molecular models were constructed using BIOVIA Materials Studio 2020. Cell and geometry optimizations were performed using the QUICKSTEP module in CP2K version 2024.3, employing the mixed Gaussian and plane-wave approach. The PBE exchange-correlation functional was used together with the DZVP-MOLOPT double-zeta basis set and Goedecker-Teter-Hutter pseudopotentials, with a plane-wave cutoff of 400 Ry. Electrostatic surface potential calculations were performed using VASP version 5.4.4 with PBE functional plus D3BJ. The ESP maps were visualized using VMD version 1.9.3. Binding energy calculations were carried out using the ADF module in AMS version 2024.104 with the BLYP functional plus D3BJ.

## Supplementary information

Supplementary Information

Transparent Peer Review file

## Source data

Source Data

## Data Availability

All data supporting the findings of this study are available within the paper and its Supplementary Information. Simulation data related to ion binding energy, hydration, and mobility are available in the NOMAD online database at: 10.17172/NOMAD/2026.03.31-1. All data are available from the corresponding authors upon request. Source data are provided with this paper.

## Code availability

The codes used for the DFT and MD simulations in this study are available from the corresponding authors upon request.
