# Supplementary material for: Ultranarrow nanochannels in a staggered two-dimensional polymer membrane enhance electric double-layer coverage for osmotic energy harvesting
Source: Nat Commun. 2026 Jun 19;17:5460. doi: 10.1038/s41467-026-74696-4 (PMC13282372; doi:10.1038/s41467-026-74696-4)
Supplement: Supplementary file 1 — Supplementary Information [file 41467_2026_74696_MOESM1_ESM.pdf]

Supplementary information for

Ultranarrow nanochannels in a staggered two-dimensional polymer membrane enhance electric double-layer coverage for osmotic energy harvesting

Feng Ni[1#], Ye Yang[2#], Shuangjie Zhao[2#], Mahabir Prasad[3,4#], Naveen Goyal[2,5], Xusheng Yang[1,2], Dongxu Wang[1,2], Jianjun Zhang[2], Mike Hambsch[6], Miroslav Polozij[2,3,4], Stefan C. B. Mannsfeld[6], Ute Kaiser[5,7], Grégory F. Schneider[8], Thomas D. Kühne[3,4,9], Thomas Heine[2,3,4], Zhiyong Wang[1,2]*, Xinliang Feng[1,2]*

[1]Max Planck Institute of Microstructure Physics, 06120 Halle (Saale), Germany

[2]Center for Advancing Electronics Dresden and Faculty of Chemistry and Food Chemistry, Technische Universität Dresden, 01069 Dresden, Germany

[3]Center for Advanced Systems Understanding, 02826 Görlitz, Germany

[4]Helmholtz-Zentrum Dresden-Rossendorf, 01328 Dresden, Germany

[5]Central Facility of Electron Microscopy, Electron Microscopy Group of Materials Science, Ulm University, Ulm 89081, Germany

[6]Center for Advancing Electronics Dresden & Faculty of Electrical and Computer Engineering, Technische Universität Dresden, 01062 Dresden, Germany

[7]Institute for Quantum Optics (IQO) and Centre for Integrated Quantum Science and Technology (IQST), Ulm University, Ulm 89081, Germany

[8]Leiden Institute of Chemistry, Leiden University, 2333CC Leiden, The Netherlands

[9]Institute of Artificial Intelligence, Chair of Computational System Sciences, Technische Universität Dresden, 01069 Dresden, Germany

[#]These authors contributed equally: F.N., Y.Y., S. Z., and Mahabir P.

Email: zhiyong.wang@mpi-halle.mpg.de; xinliang.feng@mpi-halle.mpg.de

Table of Contents

Supplementary Methods 5

Supplementary Fig. 1. 8

Supplementary Fig. 2. 9

Supplementary Fig. 3 12

Supplementary Fig. 4 13

Supplementary Fig. 5. 14

Supplementary Fig. 6 16

Supplementary Fig. 7 16

Supplementary Fig. 8 17

Supplementary Fig. 9 18

Supplementary Fig. 10 19

Supplementary Fig. 11 20

Supplementary Fig. 12 21

Supplementary Fig. 13. 22

Supplementary Fig. 14 23

Supplementary Fig. 15. 24

Supplementary Fig. 16 25

Supplementary Fig. 17 26

Supplementary Fig. 18 27

Supplementary Fig. 19 28

Supplementary Fig. 20 29

Supplementary Fig. 21. 30

Supplementary Fig. 22 31

Supplementary Fig. 23 32

Supplementary Fig. 24 33

Supplementary Fig. 25 34
Supplementary Fig. 26 35
Supplementary Fig. 27 36
Supplementary Fig. 28 37
Supplementary Fig. 29 38
Supplementary Fig. 30. 39
Supplementary Fig. 31 40
Supplementary Fig. 32 41
Supplementary Fig. 33. 42
Supplementary Fig. 34 43
Supplementary Fig. 35 44
Supplementary Fig. 36. 46
Supplementary Fig. 37 47
Supplementary Fig. 38 48
Supplementary Fig. 39 49
Supplementary Fig. 40. 50
Supplementary Fig. 41. 51
Supplementary Fig. 42 52
Supplementary Fig. 43 53
Supplementary Fig. 44 54
Supplementary Fig. 45. 55
Supplementary Fig. 46. 56
Supplementary Fig. 47. 57
Supplementary Fig. 48. 58
Supplementary Fig. 49 59
Supplementary Fig. 50 60

Supplementary Fig. 51 61

Supplementary Fig. 52 62

Supplementary Fig. 53 63

Supplementary Fig. 54 65

Supplementary Fig. 55 66

Supplementary Fig. 56 67

Supplementary Fig. 57 68

Supplementary Note 1 70

Supplementary Note 2 72

Supplementary Note 3 73

Supplementary Table 1 74

Supplementary Table 2 75

Supplementary Table 3 76

Supplementary Table 4 77

Supplementary Table 5 78

Supplementary Table 6 79

Supplementary References 80

Supplementary Methods

Materials

Sodium (9Z)-octadec-9-en-1-yl sulfate (sodium oleyl sulfate, SOS) was self-synthesized. Solvents (e.g., ethanol and chloroform), 2,2',2''-(benzene-1,3,5-triyl)triacetonitrile, cesium carbonate (Cs_2CO_3), electrolytes (KCl, NaCl, $CaCl_2 \cdot 2H_2O$, $MgCl_2 \cdot 6H_2O$, $MgSO_4$, KBr and KI), and trifluoromethanesulfonic acid (CF_3SO_3H) were all purchased from Sigma-Aldrich. 1,1′-bis(4-aminophenyl)-[4,4′-bipyridine]-1,1′-diium chloride was obtained from Ambeed, Inc. 1,1'-bis(4-formylphenyl)-[4,4'-bipyridine]-1,1'-diium dichloride was provided from BLD pharm. All chemicals were used as received without further purification. Water used in this work was purified using a Milli-Q purification system (Merck KGaA).

Molecular model construction

Both 2D polymer models, including sV2DP and eV2DP, were built using BIOVIA Materials Studio 2020. Their cell and geometry optimizations were optimized by CP2K.[1] The crystals were placed in a vacuum, with the simulation box treated as periodic in all three dimensions. Density functional theory (DFT) calculations were carried out using the QUICKSTEP module, which employs the mixed Gaussian and plane waves (GPW) approach. The Kohn–Sham orbitals were represented using the DZVP-MOLOPT double-zeta basis set[2], and the core electrons were described by Goedecker-Teter-Hutter (GTH) pseudopotentials[3,4]. A plane wave cutoff of 400 Ry was employed, and the PBE exchange-correlation density functional was chosen to be consistent with the basis set and pseudopotential.

Electrostatic surface potential (ESP) analysis

The ESP results were obtained using Vienna Ab initio Simulation Package (VASP, version 5.4.4) with PBE functional plus D3BJ (D3 with Becke-Johnson damping) dispersion. The result was subsequently visualized using VMD version 1.9.3.

Potential of Mean Force (PMF) analysis

All PMF simulations were performed using the GROMACS package[5] under canonical conditions (NVT) at 298 K. Temperature was controlled with a V-rescale thermostat[6] using a 1-ps time step. Lennard-Jones and Coulombic interactions were truncated at 1.2 nm, with long-range Coulombic interactions calculated via the Particle Mesh Ewald (PME) method[7,8]. The Lorentz–Berthelot combination rule was applied to determine the Lennard-Jones parameters

for cross-interactions between nonbonded atoms[9]. Newton's equations of motion were solved using the leapfrog integrator[10,11] with an integration time step of 1 fs.

For the water model, a rigid, non-polarizable SPC/E potential[12] was employed. This three-site model assigns a positive charge of 0.4238e to each hydrogen and a negative charge of –0.8476e to the oxygen atom, with a single Lennard-Jones site centered on oxygen. The intramolecular O-H distance was fixed at 0.1 nm, and the H-O-H bond angle was fixed at 109.47°. The eV2DP and sV2DP membranes were modeled using the OPLS all-atom force field[13]. Bond constraints for water molecules were handled by the SETTLE algorithm[14], while all other bonds involving hydrogen atoms were constrained using the LINCS algorithm[15,16].

The whole simulation system comprised approximately 20,450 atoms in a triclinic box with edge lengths of a = 4.5603 nm, b = 4.3687 nm, c = 12.0000 nm, and angles of $\alpha = 93.64^o$, $\beta = 88.08^o$, and $\gamma = 61.42^o$. Periodic boundary conditions were applied in all three directions. The system consisted of a six-layer eV2DP/sV2DP membrane, roughly 6,500 SPC/E water molecules, and 38 ions uniformly distributed on either side of the membrane. The six-layer membrane contained a total of 36e charges; consequently, 36 chloride ions served as counterions to neutralize the system, and an additional chloride and potassium ion were included to study their transport behavior. The simulation protocol began with steepest descent energy minimization to remove any unfavorable contacts, followed by a 5 ns NVT equilibration. From the equilibrated structure, 100 independent configurations (sampling windows) were generated using a force constant of 1000 kJ/mol/nm^2. Each configuration then underwent a 60 ns umbrella sampling simulation with a force constant of 100 kJ/mol/nm^2. Finally, the PMF for the transport of chloride and potassium ions across the membrane was extracted using the Weighted Histogram Analysis Method (WHAM) implemented in GROMACS via the wham module.

2D ion density analysis

The system comprises three-layer eV2DP/sV2DP membranes, approximately 25,300 water molecules, graphene walls positioned on either side of the membrane along the ***c***-axis, and a KCl solution confined within a triclinic box with edge lengths of a = 9.1242 nm, b = 8.7374 nm, and c = 25.0000 nm, and angles of $\alpha = 93.64^o$, $\beta = 88.08^o$, and $\gamma = 61.42^o$. The separation between the two graphene walls is 12.2 nm, and periodic boundary conditions are applied in all three directions. The membranes divide the simulation box into a 0.1 M KCl solution on the left and a 0.01 M KCl solution on the right. To ensure electrical neutrality, an extra 72 chloride

ions were added as counterions to the membrane. The system underwent energy minimization using the steepest descent method, followed by a 1 ns NVT equilibration. Finally, a 20 ns production run was performed to generate 2D density maps.

Binding energy calculation

Binding energy between anions and the pyridine N site in sV2DP was calculated by first cutting the minimal repeating unit from the 2DP framework and then positioning anions and water molecules around the adsorption nitrogen site. The resulting system was optimized using the ADF module within the Amsterdam Modeling Suite (AMS 2024.104), employing the BLYP functional plus D3BJ. Subsequently, the same function was utilized to compute the energies of the water-anion complex, the isolated 2DP framework, and the entire system, enabling the determination of the binding energy between the hydrated anion and the 2DP framework.

Mean Square Displacement (MSD) calculations

Non-equilibrium Molecular Dynamics (NEMD) simulations were conducted to investigate the transport behavior of anions (Cl^-, Br^-, and I^-) across the sV2DP membranes. The membranes were centrally positioned within a simulation box with dimensions of 91.21 Å × 87.37 Å × 100.00 Å. A total of 48 anions were placed on one side of the membrane, and 1,634 water molecules were used to fill the remaining volume of the box. The simulations were performed under the canonical ensemble (NVT), with the system temperature maintained at 300 K using the Nose-Hoover thermostat. A damping constant of 2 ps was applied to minimize artifacts while maximizing the capture of natural interactions within the system. The time step was set to 0.5 fs, and the total simulation duration was 6 ns. Identical simulations were performed for each anion type to ensure consistent data collection and reliable comparison. VMD 1.9.3 was used to visualize MSD results. Additionally, MSD results can be used to calculate the effective ion diffusion coefficients (D_{eff}), derived from the slopes (k) of the MSD-versus-time curves using the Einstein relation:

$$D_{eff} = k/2d \qquad (1)$$

where the factor d accounts for the dimensionality of the system (here d = 3). The slope used in this calculation is taken from the linear region of the MSD-versus-time plots.

Effective ion-accessible cross-sectional area (*f(D)*) and effective diameter (D_{eff}) of sV2DP: As shown in Supplementary Fig. 1, *f(D)* is defined as the effective ion-accessible cross-sectional area of the channel (units, nm^2), while *D* is an effective diameter (units, nm) derived from the same cross-section. For the ABC-stacked sV2DP membrane, the channel window can be approximated as an equilateral triangle with side length a = 2.36 nm (Fig. R1). To obtain a physically meaningful descriptor for ion transport, instead of using the triangular side length directly, we adopt two commonly used equivalent diameters:

1) Inscribed-circle (effective electrostatic) diameter:

$$D_{in} = \frac{\sqrt{3}}{3} a \approx 1.36 \text{ nm} \quad (2)$$

2) Hydraulic diameter:

$$D_h = \frac{4A}{P} = \frac{\sqrt{3}}{3} a \approx 1.36 \text{ nm} \quad (3)$$

From an equilateral triangle, $D_{in} = D_h$. We therefore adopt D = 1.36 nm as a conservative estimate of the ion-accessible permeation diameter. The corresponding effective channel area is then calculated as:

$$f(D) = \pi \left(\frac{D}{2}\right)^2 \approx 1.46 \text{ nm}^2 \quad (4)$$

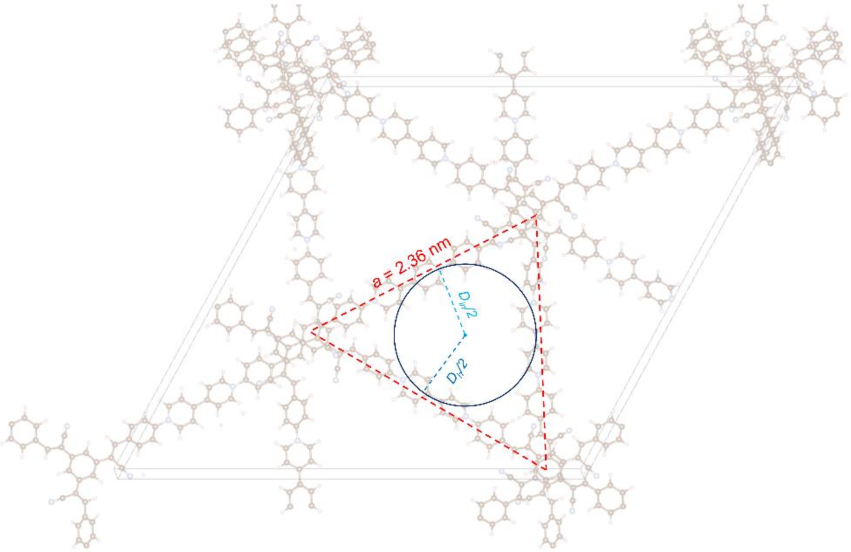

Supplementary Fig. 1| Definition of effective channel diameters in sV2DP. Schematics of effective D (D_{in} and D_h) within the sV2DP units.

e2DP

$C_{electrolyte}$ **(M)**	0.01	0.1	0.25	0.5	1
λ_D **(nm)**	3.03	0.96	0.605	0.43	0.3

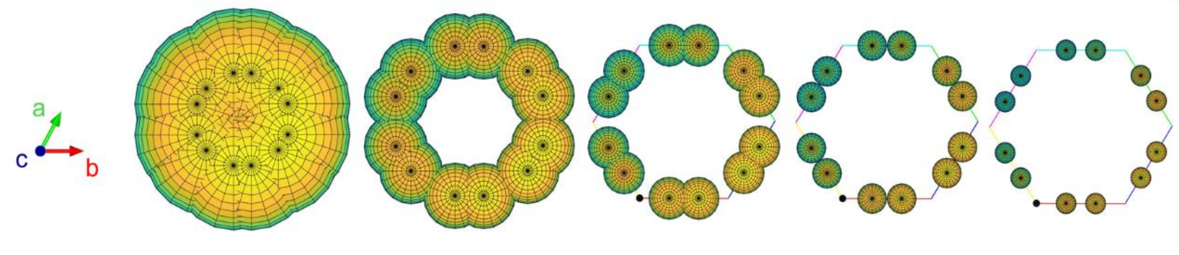

η_{EDL} **(%)**	100	66.6	37.3	19.6	9.5

s2DP

$C_{electrolyte}$ **(M)**	0.01	0.1	0.25	0.5	1
λ_D **(nm)**	3.03	0.96	0.605	0.43	0.3

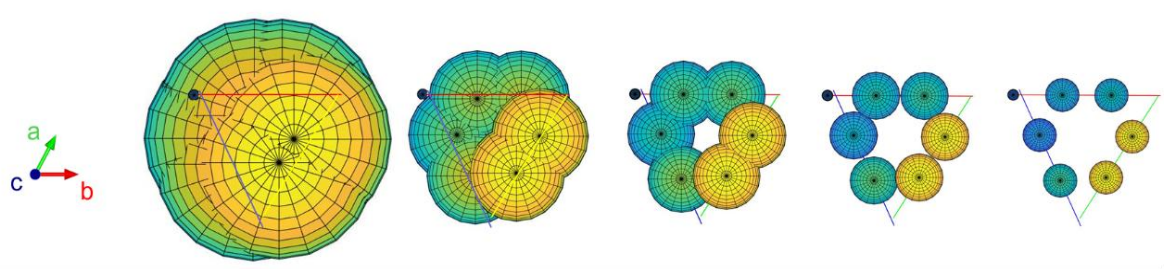

η_{EDL} **(%)**	100	100	90	63	31

Supplementary Fig. 2| Calculations of η_{EDL} in 2DP models. In this work, the charges on the 2DP framework are treated as point charges, and the resulting perturbation extends over a characteristic distance on the order of λ_D, which is approximated by the *Debye-Hückel* relation: $\lambda_D=(\varepsilon k_B T/2e^2 I)^{-1/2}$ (where $\boldsymbol{\varepsilon}$ is the solution permittivity, k_B is the Boltzmann constant, T represents the temperature, and e is the elementary charge).[17,18] The ionic strength, I, is defined as: $I=1/2\sum_i C_s z_i^2$ where C_s and z_i is the solution concentration and the charge of each ion species, respectively. The η_{EDL} is defined as the fraction of $f(D)$ (along the c-axis) covered by the resulting EDL under varying ionic strengths. Increasing ionic strength shortens λ_D, resulting in weaker EDL coverage and thus a lower η_{EDL}.

Numerical simulation:

Establishment of 2DP channel models. To investigate the influence of interlayer ABC stacking on ion transport behavior, two distinct 2DP nanochannel models with equivalent cross-sectional areas but different surface configurations were constructed: (1) the e2DP model features a single hexagonal channel with an intrinsic side length of a = 2.0 nm, while (2) the s2DP model comprises six ultranarrow triangular subchannels embedded within the same hexagonal framework as the e2DP (Supplementary Fig. 2). In both models, the total surface charge density (Σ) was assumed to be identical and uniformly distributed across all internal channel surfaces. Each simulation system consists of two electrolyte reservoirs connected by a single 2DP channel segment based on the respective geometry. For consistent comparison, the channel length was fixed at l = 50 nm for both models.

Estimation of Σ in designed channels. For e2DP channels, the total surface area (A) of the channel walls accommodates the total charge (σ), so $\Sigma = \sigma / A$. In the s2DP model, interlayer ABC stacking introduces six additional internal faces within the same hexagonal framework. Notably, each internal face is shared by two adjacent subchannels, effectively doubling the total surface area compared to e2DP. Given that both systems possess the same σ, the total $\sum$ of e2DP is approximately twice that of s2DP (i.e., $\sum_{e2DP} = 2\sum_{s2DP}$).

Finite element calculation based on PNP equations: To quantitatively describe ion transport within the above 2DP channels, the coupled Poisson–Nernst–Planck (PNP) equations were employed.[19,20] These equations were solved using appropriate boundary conditions to generate spatial profiles of ion concentrations and ionic fluxes. Specifically, the Nernst–Planck equation (5) governs the ion flux J_i within a charged nanochannel:

$$J_i = D_i\left(\nabla_{C_i} + \frac{z_i F c_i}{RT}\nabla\varphi\right) \qquad (5)$$

where j_i, D_i, c_i, z_i and φ represent the ionic flux, diffusion coefficient, ion concentration, valence and diffusion potential for each species i, respectively. The Poisson Equation (6) describes the relationship between diffusion potential and ion concentration:

$$\nabla^2\varphi = -\frac{e}{\varepsilon_0\varepsilon_r}\sum\nolimits_I z_i c_i \qquad (6)$$

where ε_0 and ε_r are the permittivity of free space and the relative permittivity of the medium. At steady state, ion fluxes must satisfy the continuity condition, as equation (7):

$$\nabla \cdot J_i = 0 \qquad (7)$$

The boundary condition for the electrostatic potential on the channel walls is defined by equation (8):

$$\mathrm{n} \cdot \nabla \varphi = \frac{\sigma}{\varepsilon_0 \varepsilon_r} \qquad (8)$$

where σ is the surface charge density, and the ion flux has zero normal components at boundaries:

$$\mathrm{n} \cdot J_i = 0 \qquad (9)$$

All numerical simulations were performed using COMSOL Multiphysics, a finite-element software package.

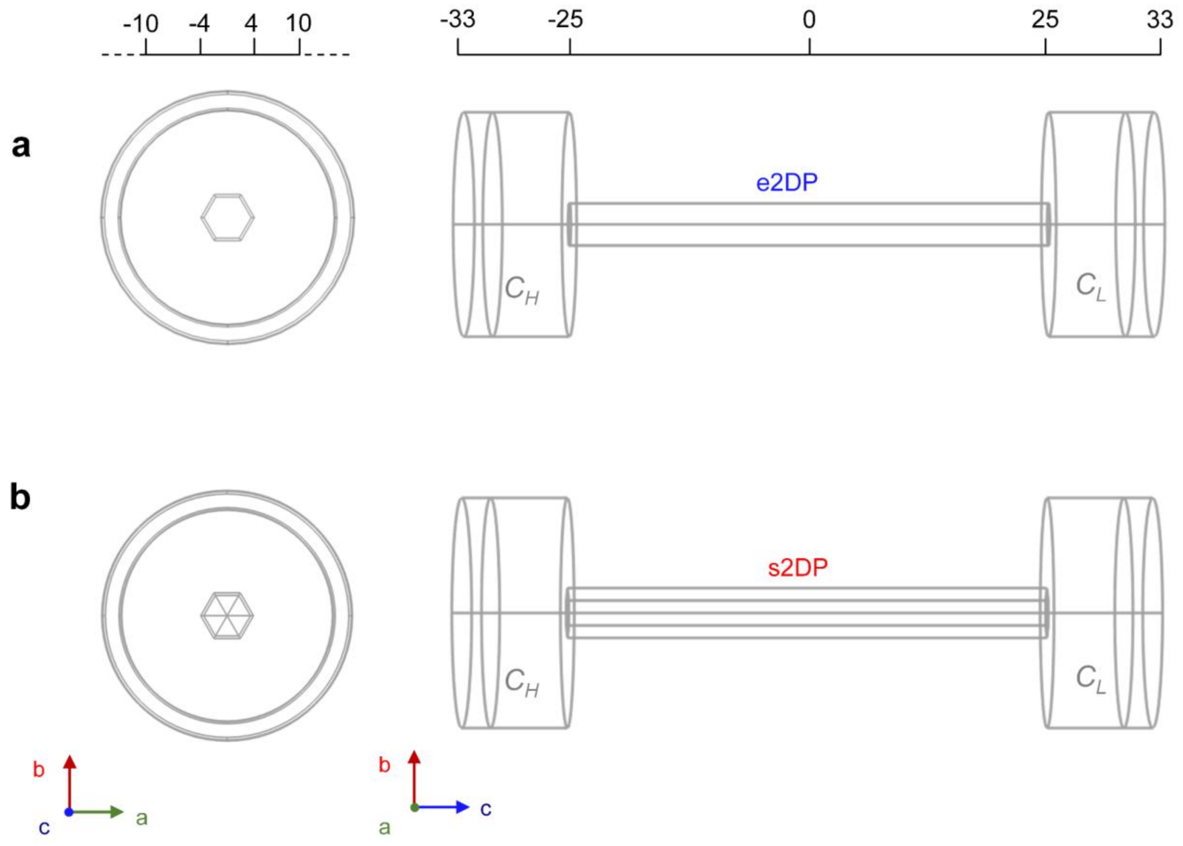

Supplementary Fig. 3| Schematics of 2DP models for numerical simulations. a, e2DP and **b**, s2DP models.

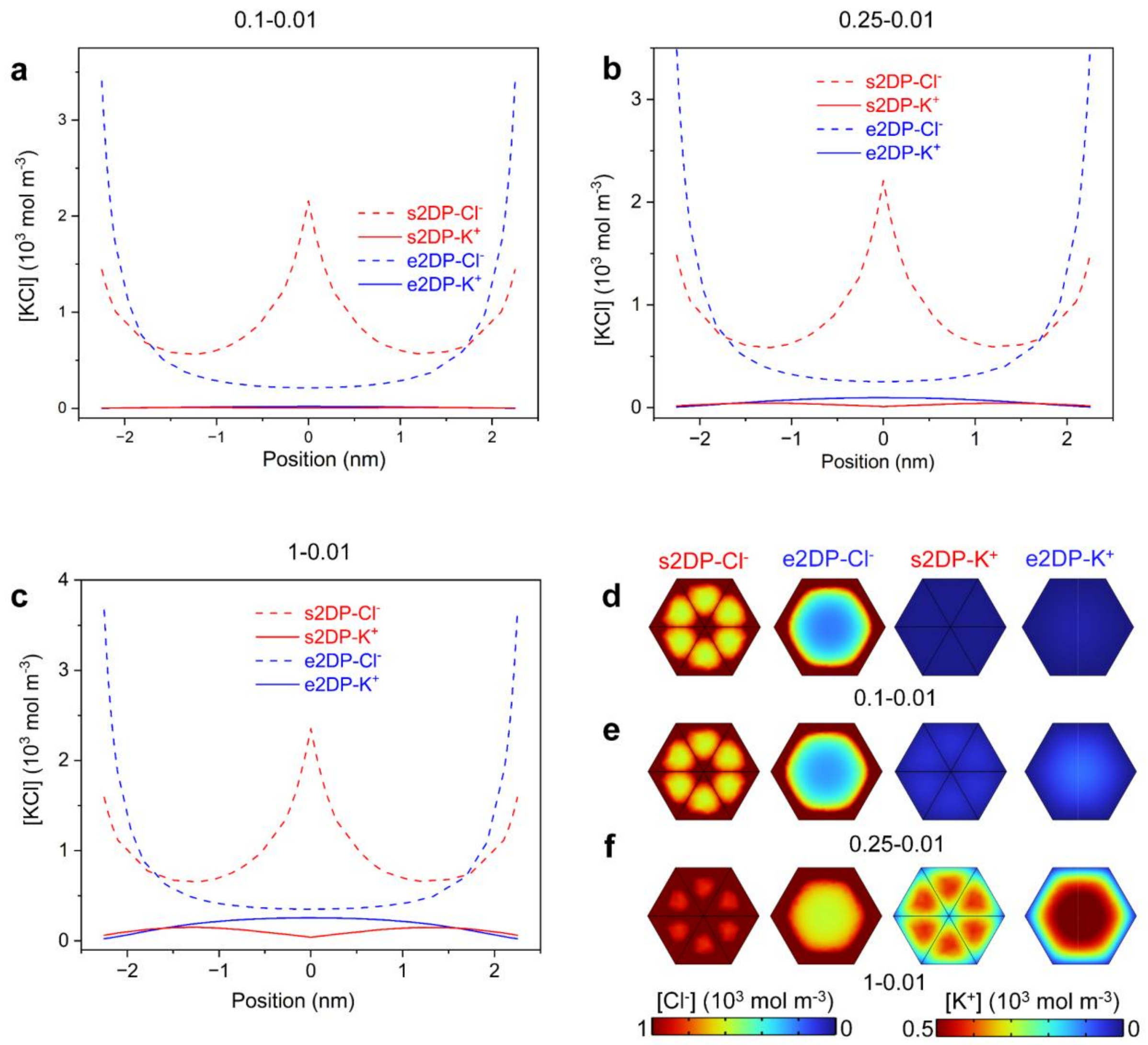

Supplementary Fig. 4| Ion distribution profiles across e2DP and s2DP under varying KCl gradients. a-c, Radial ion concentration distributions within 2DP channels under KCl gradients of (a) 0.1/0.01 M, (b) 0.25/0.01 M, and (c) 1/0.01 M. **d-f**, Corresponding ion concentration maps: (d) 0.1/0.01 M, (e) 0.25/0.01 M, and (f) 1/0.01 M.

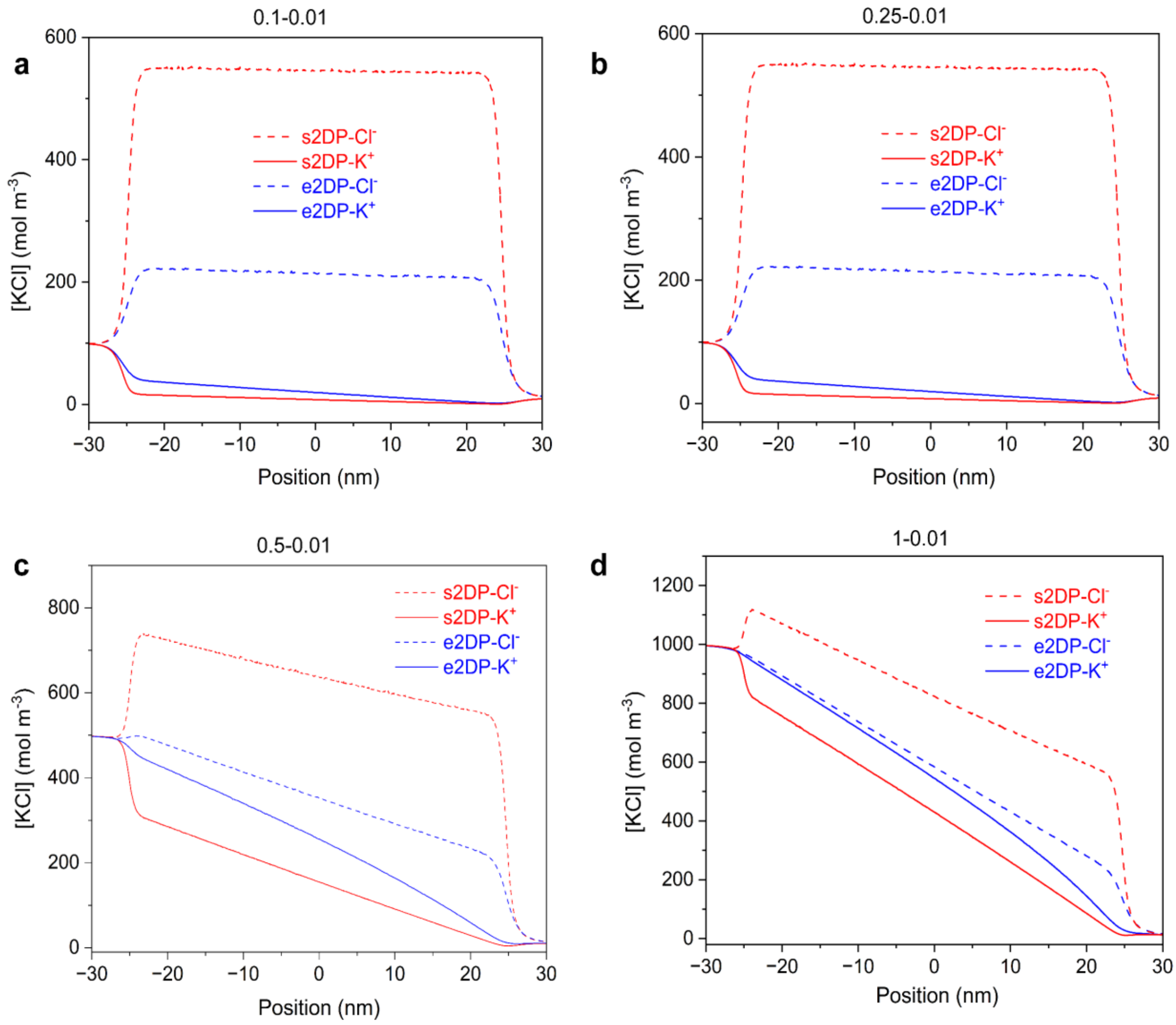

Supplementary Fig. 5| Longitudinal ion distribution profiles across e2DP and s2DP under varying KCl gradients. a, 0.1/0.01 M, **b**, 0.25/0.01 M, **c**, 0.5/0.01 M, and **d**, 1/0.01 M KCl gradients.

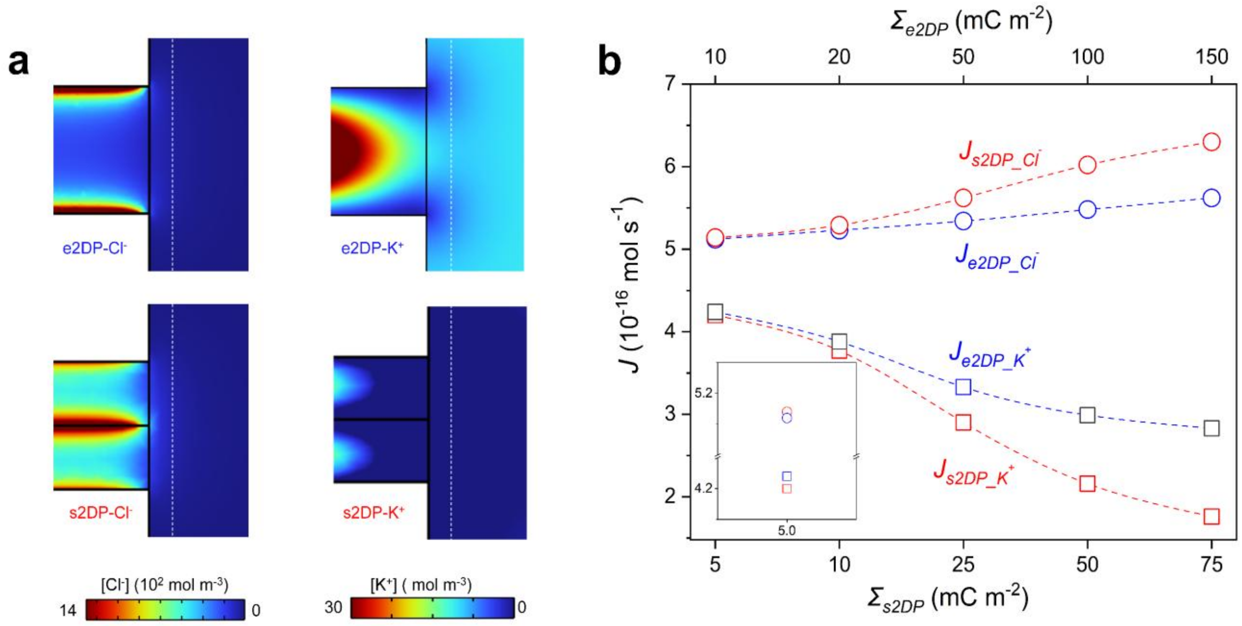

Supplementary Fig. 6| Calculated anion-selective transport across e2DP and s2DP under a 0.5/0.01 M KCl gradient. a, Simulated longitudinal ion distribution maps on the dilute side (0.01 M) demonstrate that the s2DP channel facilitates greater Cl^- enrichment while effectively suppressing K^+ accumulation compared to e2DP. **b**, Integrated ionic fluxes (*J*) across the white dashed plane in (**a**) quantitatively reflect the concentration variations on the dilute side, which correlate with the observed voltage and current outputs.

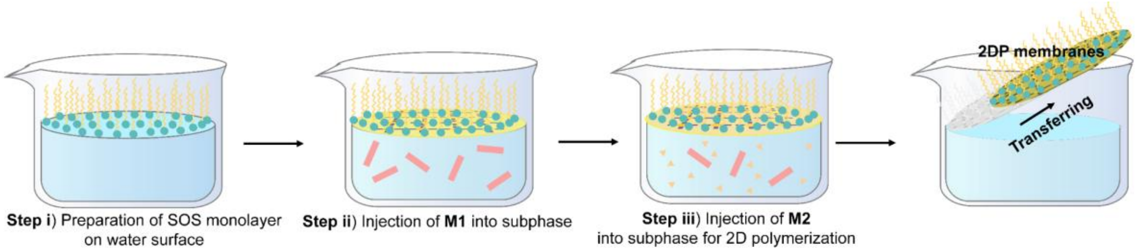

Supplementary Fig. 7| Surfactant-monolayer-assisted interfacial synthesis (SMAIS) method for producing 2DP membranes on the water surface. The method includes three steps: **Step i)** spreading surfactant molecules (sodium oleyl sulfate, SOS) on the water surface to create an organized floating monolayer. **Step ii)** injecting an aqueous solution of monomer 1 (**M1**), allowing the monomers to diffuse, be absorbed, and become preorganized beneath the surfactant monolayer. **Step iii)** adding monomer 2 (**M2)** to trigger 2D polymerization at the water-air interface.

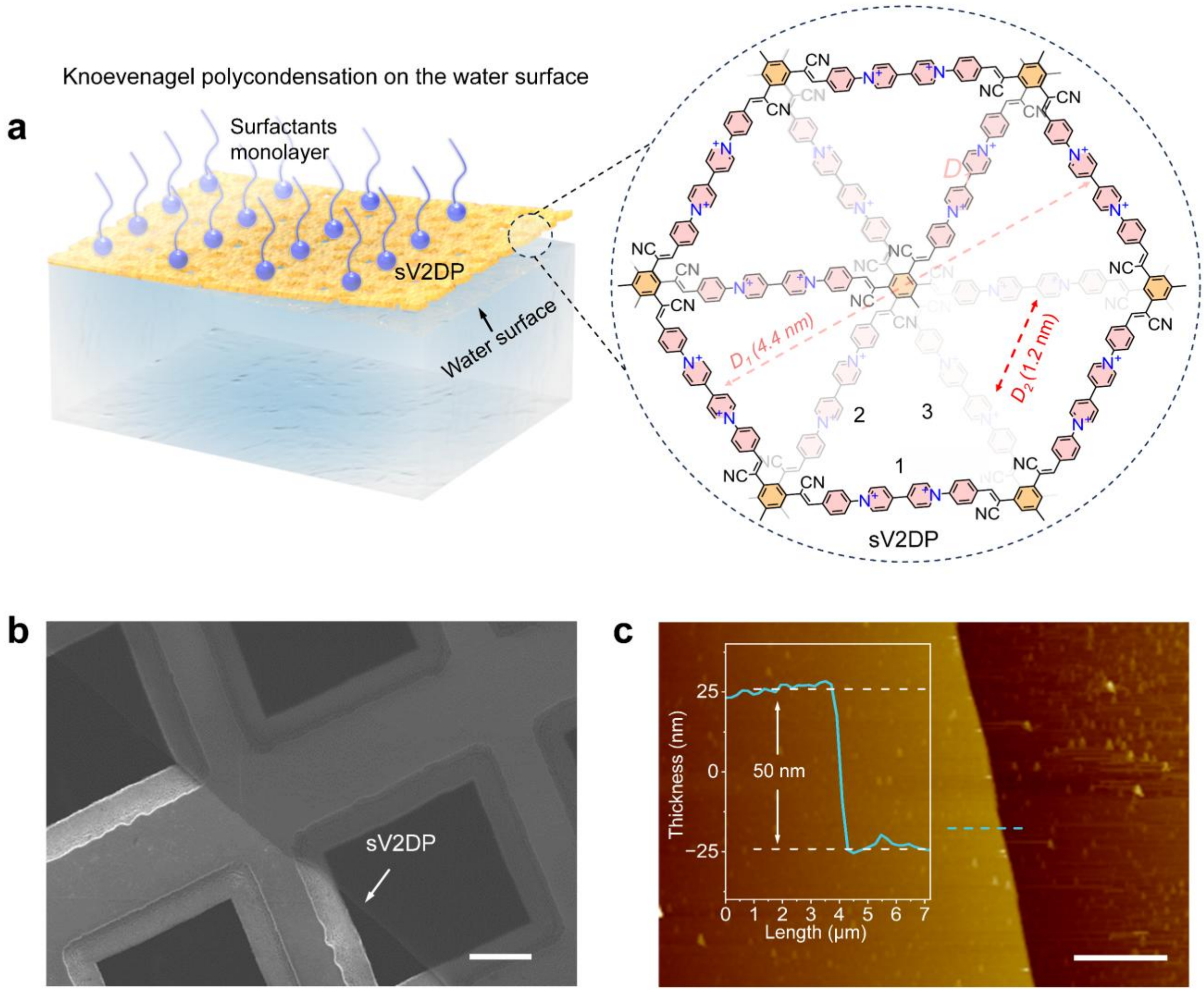

Supplementary Fig. 8| Synthesis of ABC-stacked sV2DP membrane on the water surface. **a**, Schematic of Knoevenagel polycondensation for synthesizing an sV2DP membrane on the water surface. Right inset: chemical and topological models of the sV2DP framework. **b**, Optical image of a uniform sV2DP membrane supported on a porous copper grid (scale bar: 20 µm). **c**, Atomic force microscopy (AFM) image and height profile of an sV2DP membrane (scale bar: 4 µm).

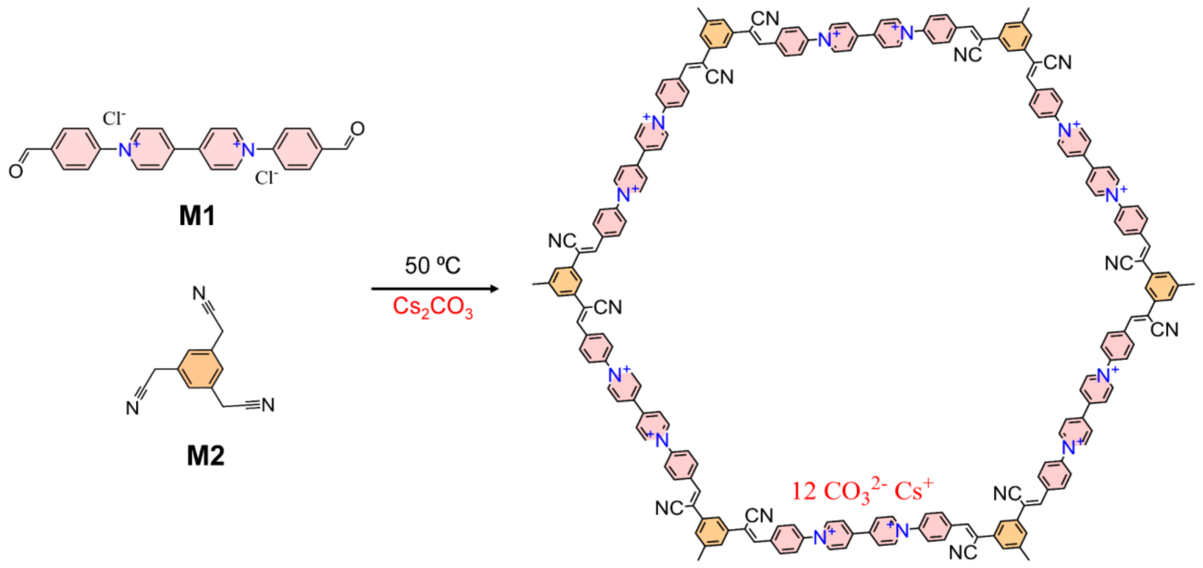

Supplementary Fig. 9| Synthetic route for sV2DP membranes via Knoevenagel polymerization. The polymerization was conducted at 50 °C using Cs_2CO_3 as the base to promote the irreversible Knoevenagel condensation.

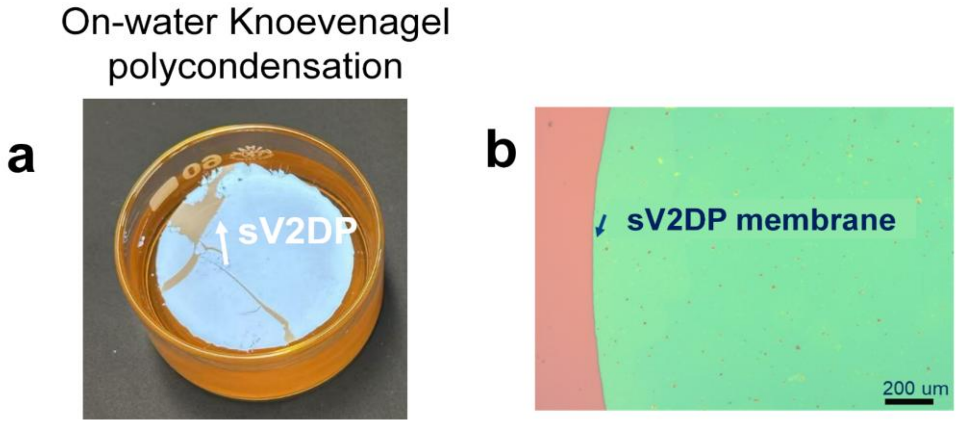

Supplementary Fig. 10| Visualizing a synthesized sV2DP membrane. a, A large-area (~ 28 cm^2), yellow, and uniform membrane formed on the water surface, showcasing the successful synthesis of an sV2DP membrane. **b**, Optical image of the resultant sV2DP membranes, high-lighting their continuous and defect-free morphology.

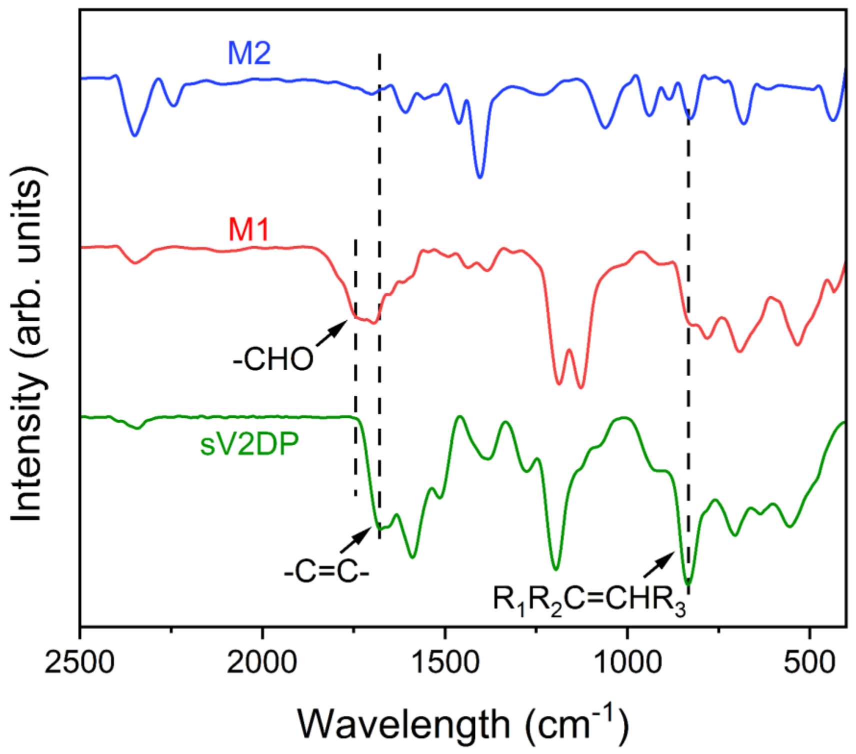

Supplementary Fig. 11| ATR-FTIR tests for the synthesis of sV2DP membranes. The results revealed the successful formation of vinylene linkages in sV2DP membranes, as indicated by the emergence of $\nu_{C=C}$ (~ 1,668 cm^{-1}) and $\nu_{R_2C=CHR}$ (~ 834 cm^{-1}) vibrations, along with the disappearance of the $\nu_{C=O}$ stretching vibration (~ 1,741 cm^{-1}).[21]

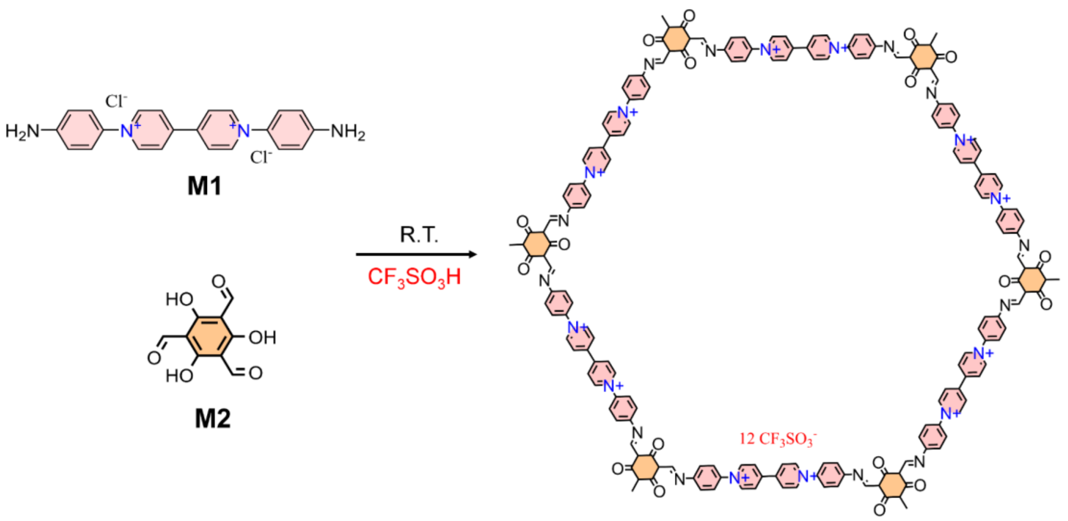

Supplementary Fig. 12| Synthetic route for es-V2DP membranes by a Schiff-base reaction. The polymerization was conducted at room temperature (R.T.) using the CF_3SO_3H-catalysed conditions.

The formation mechanisms for the ABC-stacking interlayer arrangement in sV2DP.

In normal imine-linked es-V2DPs (Supplementary Fig. 13a), the hydroxyl groups on node molecules can form hydrogen bonds with imine linkages, thereby reducing the rotation of polymer backbones. This facilitates the π-orbital overlap between adjacent layers, promoting the AA stacking of the es-V2DP layer. In contrast, sV2DP features bulky acetonitrile groups near the node molecules (Supplementary Fig. 13b). This steric hindrance within the molecular architecture induces torsional strain, causing the sV2DP backbone to deviate from planarity and limiting the π-π interaction. ABC stacking predominates in the sV2DP under such weakened interlayer interactions, as diminished electronic coupling lowers the energy barrier for staggered layer registry.[22-25]

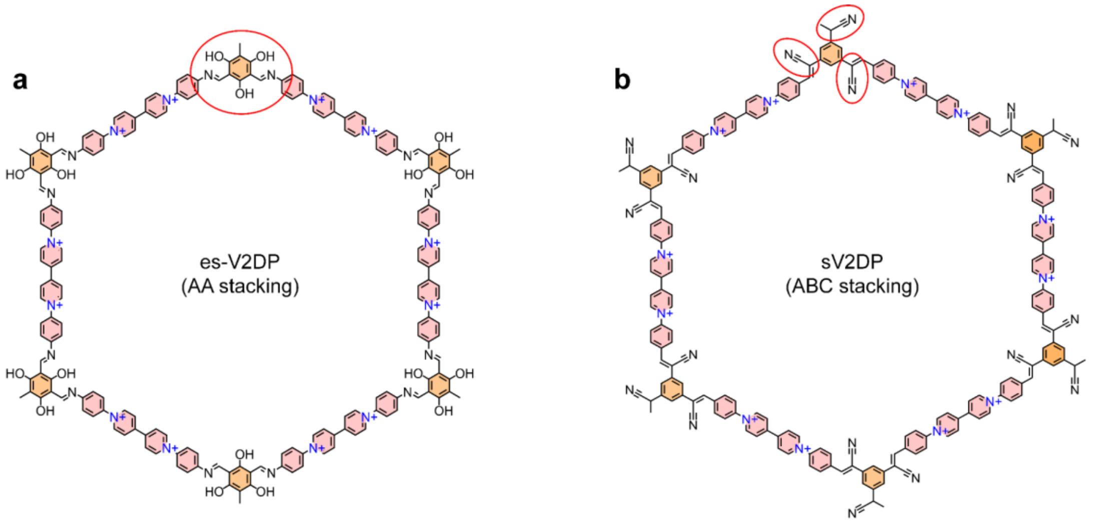

Supplementary Fig. 13| The formation mechanisms for ABC-stacking interlayer arrangement in sV2DP. Chemical structures for es-V2DPs (**a**) and sV2DP (**b**) units, respectively.

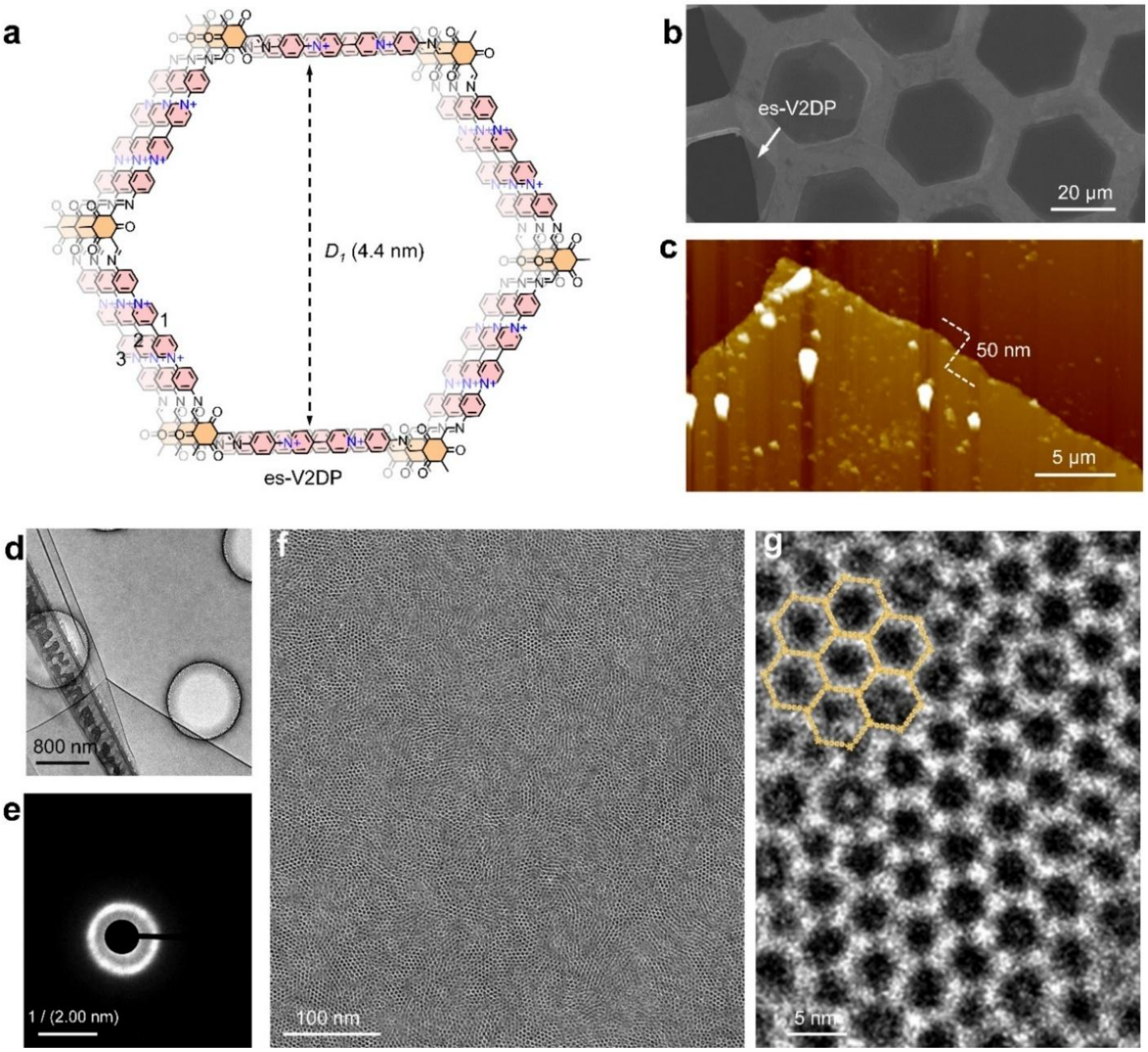

Supplementary Fig. 14| Synthesis of AA-stacked es-V2DP membrane. a, Chemical and topological models of the es-V2DP framework. **b**, Photograph of an ultrathin, self-supporting es-V2DP membrane transferred onto a porous copper grid. **c**, AFM measurements indicating a membrane thickness of ~ 50 nm. **d**, TEM image, **e**, corresponding SAED, and **f**, **g**, HRTEM images of an es-V2DP membrane, confirming its fully crystalline nature with a hexagonal lattice.

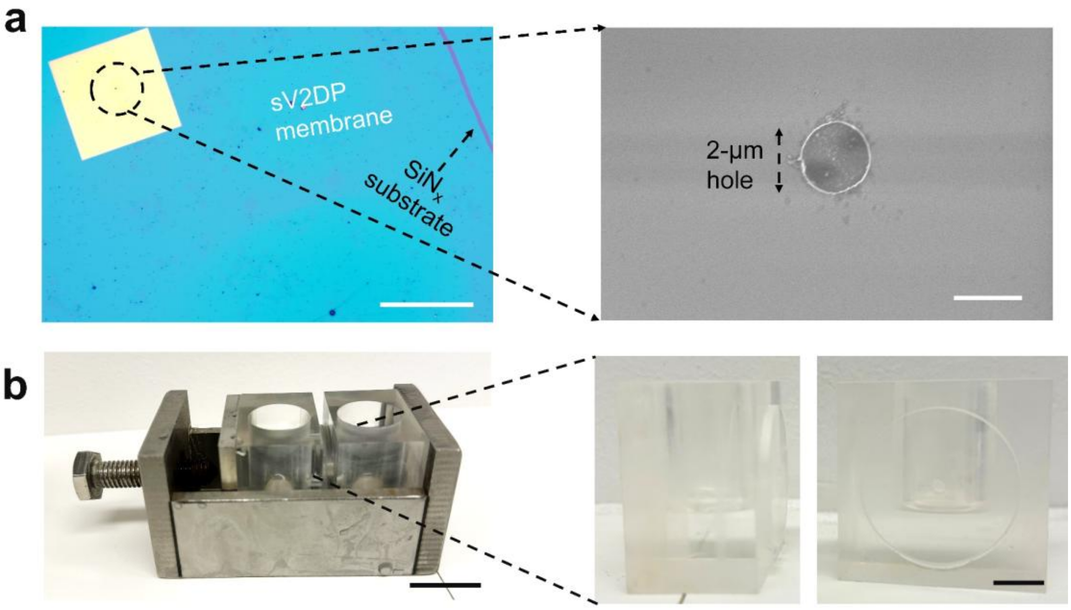

Supplementary Fig. 15| Setups for electrochemical measurements. a, A sV2DP membrane was transferred onto a 2-μm-diameter hole supported by a SiN_x substrate and subsequently sandwiched between two reservoirs for measurements. Scale bar: 100 μm (left inset) and 2 μm (right inset). **b**, Self-made H-cell setup with two reservoirs. The reservoir volume of each chamber is 8.0 ml. Scale bar: 3 cm (left inset) and 1 cm (right inset).

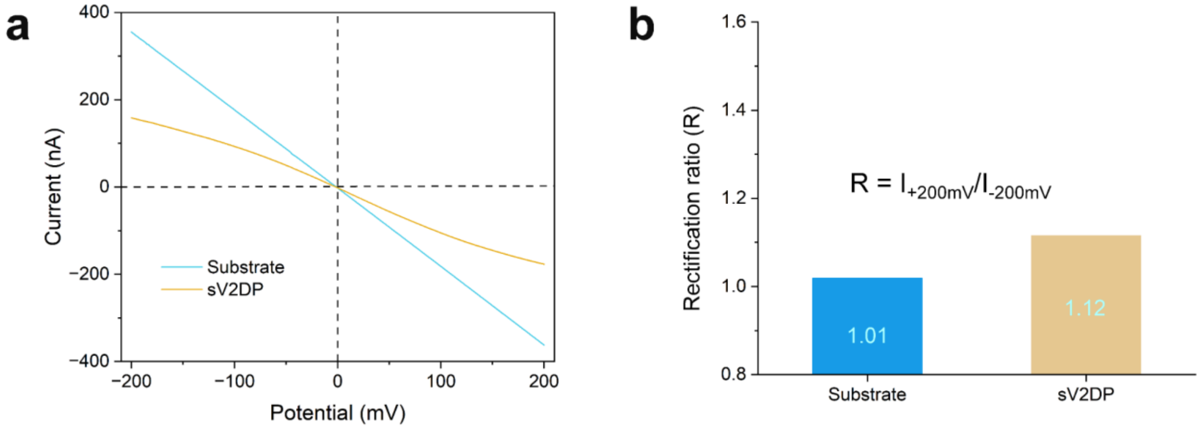

Supplementary Fig. 16| Ionic rectification of sV2DP membranes on substrates. a, *I*–*V* curves recorded in 0.1 M KCl for the bare SiN_x substrate (control) and after integration with a sV2DP membrane. **b**, Corresponding rectification ratio (R = $|I_+/I_-|$ at ± 200 mV). Note that this rectification behavior is attributed to the coupling between the charged membrane pores and the SiN_x substrate pore, which creates effectively asymmetric ionic boundary conditions and thus different degrees of concentration polarization (CP) under opposite bias polarities.

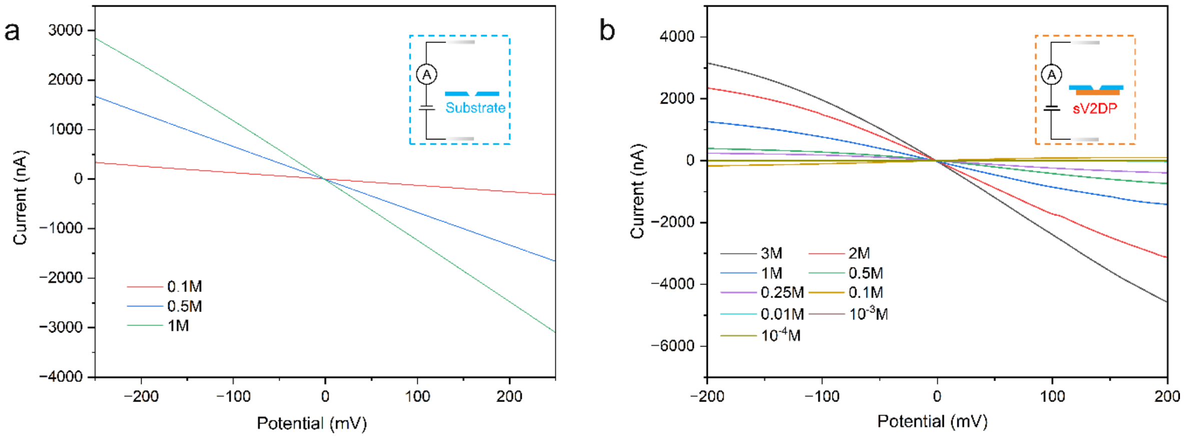

Supplementary Fig. 17| Concentration-dependent ionic conductance of bare substrates and sV2DP membranes. a, *I-V* curves of the bare SiN_x substrate with a 2-µm-diameter hole, measured in KCl solutions. **b**, *I-V* curves of a sV2DP membrane transferred onto identical substrate holes under the same KCl concentration range (10^{-5} to 1 M).

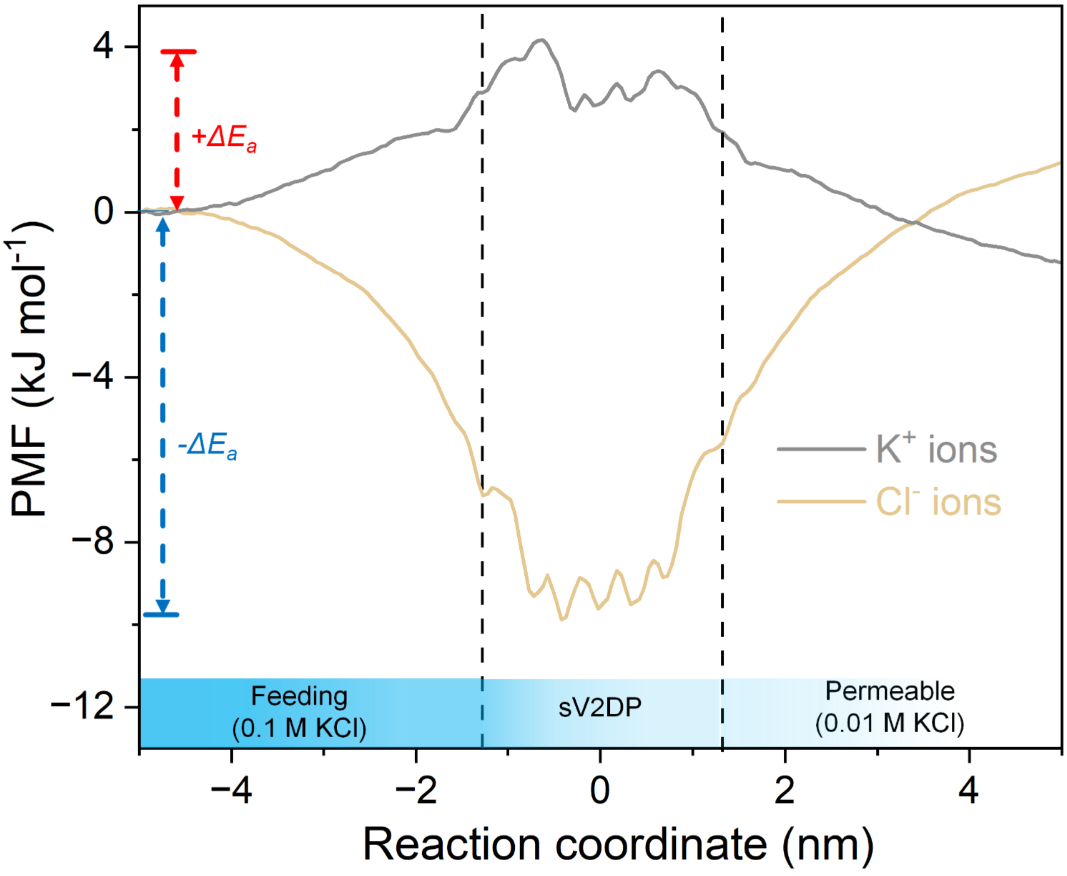

Supplementary Fig. 18| Transmembrane barrier analysis for Cl^- and K^+ ions across the sV2DP membrane. PMF results indicate a favorable free-energy profile for Cl^- transport (-10.71 kJ·mol^{-1}), whereas K^+ encounters an unfavorable barrier of +4.17 kJ·mol^{-1}, highlighting the energetically favorable Cl^- transport across a sV2DP membrane.

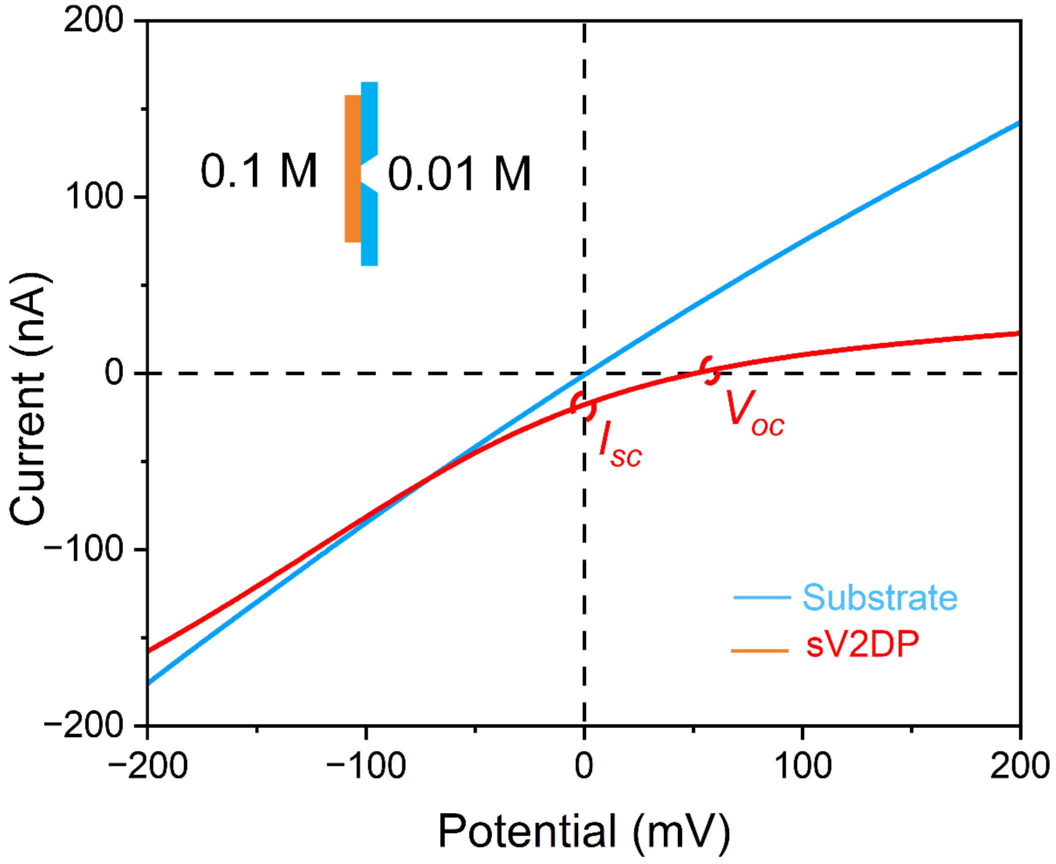

Supplementary Fig. 19| Comparison of substrate and sV2DP membrane responses under a 10-fold KCl gradient. *I*-*V* curves of the substrate before and after being covered with an sV2DP membrane under a 10-fold KCl gradient.

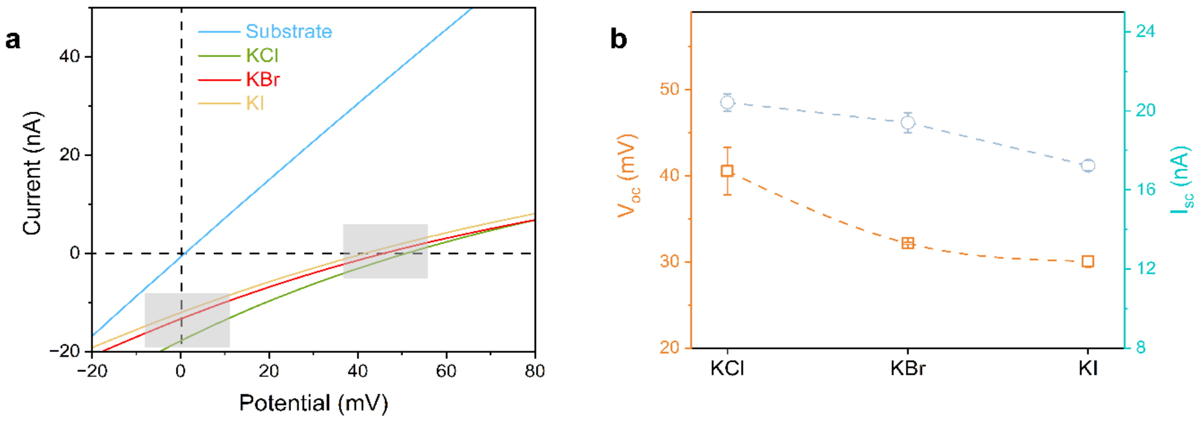

Supplementary Fig. 20| The transmembrane anion transport properties of the sV2DP membrane. a, *I-V* curves measured under the 10-fold gradients. **b**, The V_{oc} and I_{sc} values were derived from **Supplementary Fig. 1** the **a.** Data are mean ± SD from n = 3 independent measurements.

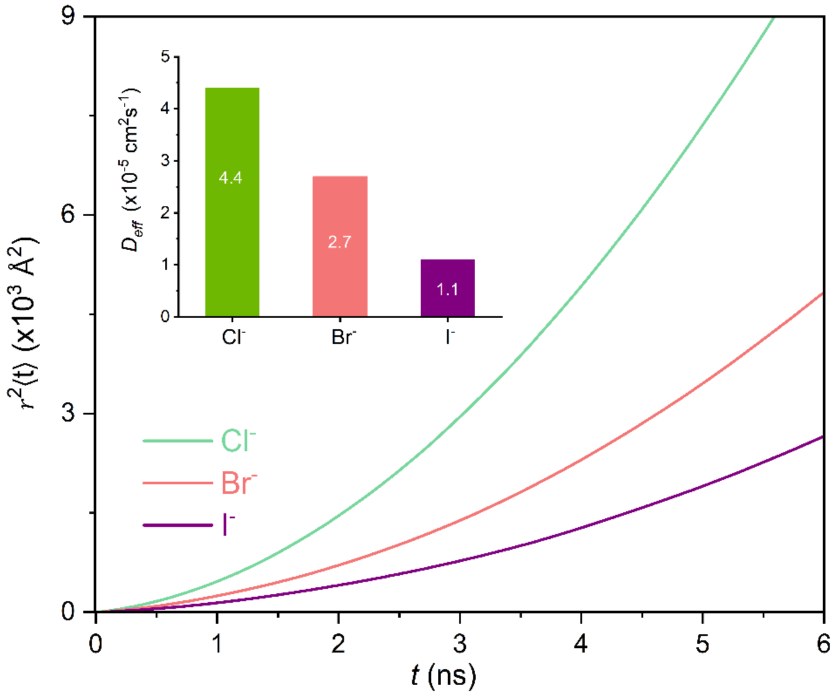

Supplementary Fig. 21| Halide-ion mobility in sV2DP nanochannels. Time-averaged mean square displacement (MSD, ⟨$r^2(t)$⟩) curves for Cl^-, Br^-, and I^- ions translocating across the sV2DP membrane. Inset: calculated D_{eff} values under a 10-fold gradient.

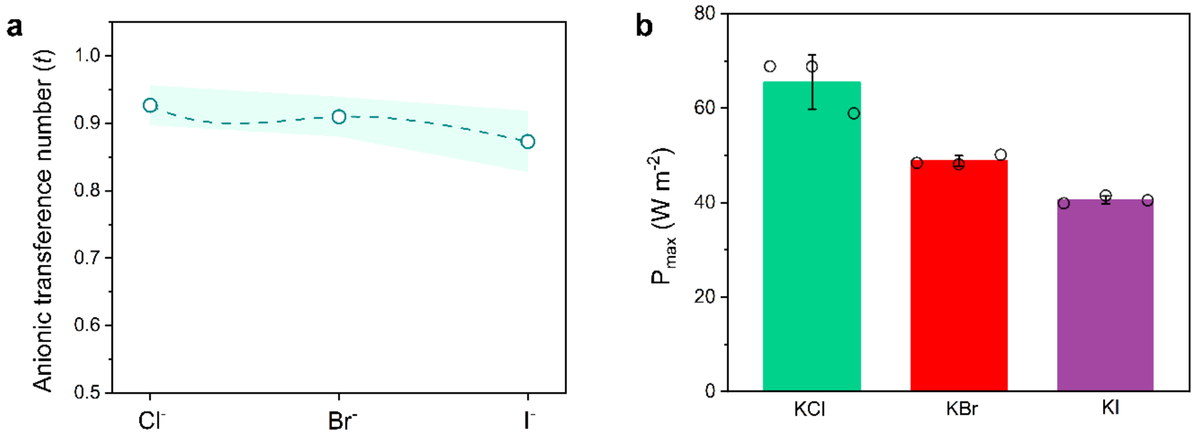

Supplementary Fig. 22| Halide-dependent osmotic power performance of sV2DP membranes. a, Anion transference number (t_-) and **b**, P_{max} values of sV2DP membranes under the 10-fold gradients, respectively. Data are mean ± SD from n = 3 independent measurements.

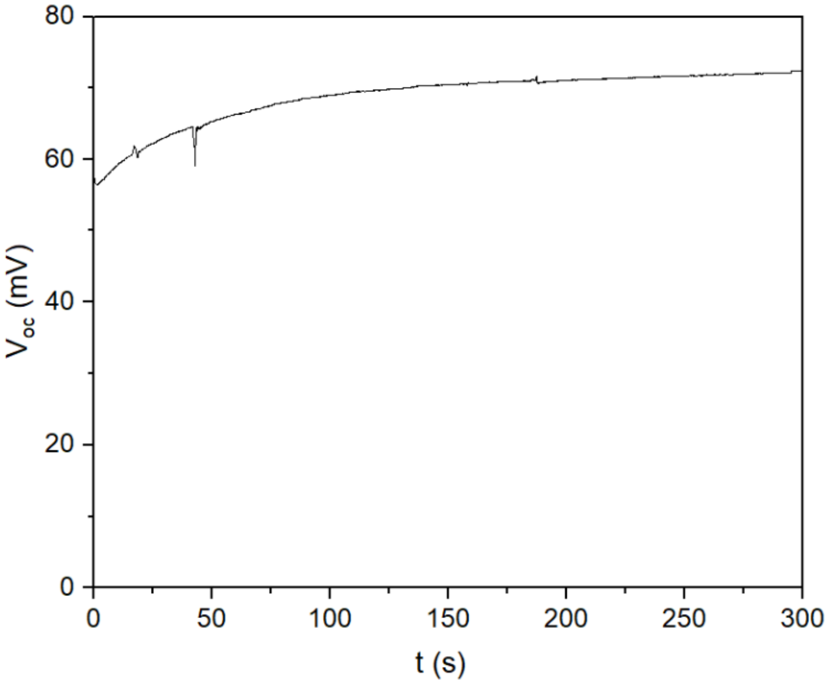

Supplementary Fig. 23| Stability of the *V_{oc}* under a KCl concentration gradient. V_{oc} measurements versus time (*t*) for SV2DP membrane under 0.5/0.01 KCl gradients.

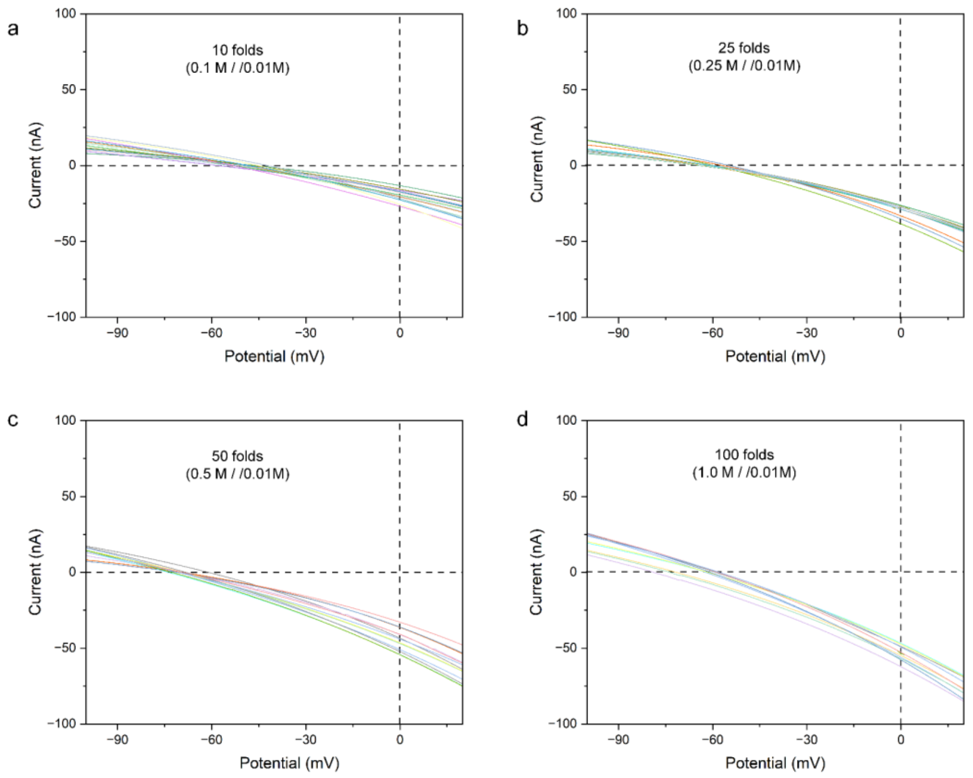

Supplementary Fig. 24| Reproducibility of osmotic current generation by sV2DP membranes. *I-V* curves of sV2DP membranes recorded from 15 independent measurements under KCl concentration gradients of (a) 10-fold (0.1/0.01 M), (b) 25-fold (0.25/0.01 M), (c) 50-fold (0.5/0.01 M), and (d) 100-fold (1/0.01 M).

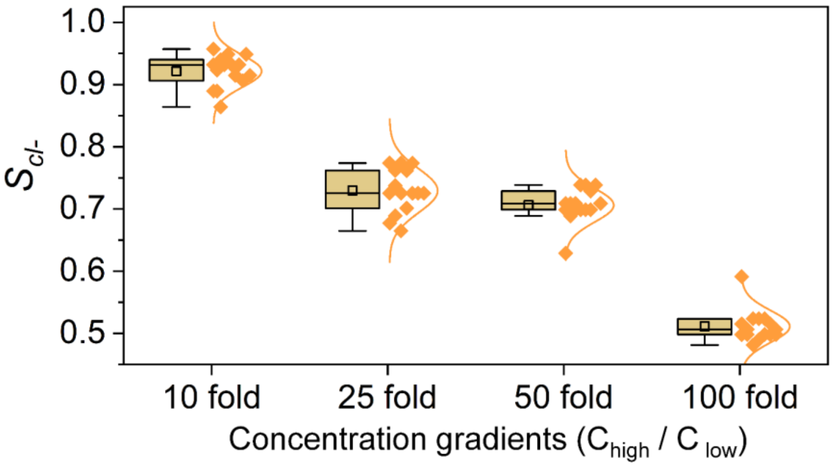

Supplementary Fig. 25| Inferred Cl^- selectivity coefficients (S_{cl^-}) of sV2DP under varied KCl gradients. Individual data points are shown alongside the box plots, with half-violin plots indicating the data distribution. Boxes indicate the 25th-75th percentiles, center lines indicate the median, and whiskers extend to values within 1.5× the interquartile range. Data were obtained from n = 15 independent measurements.

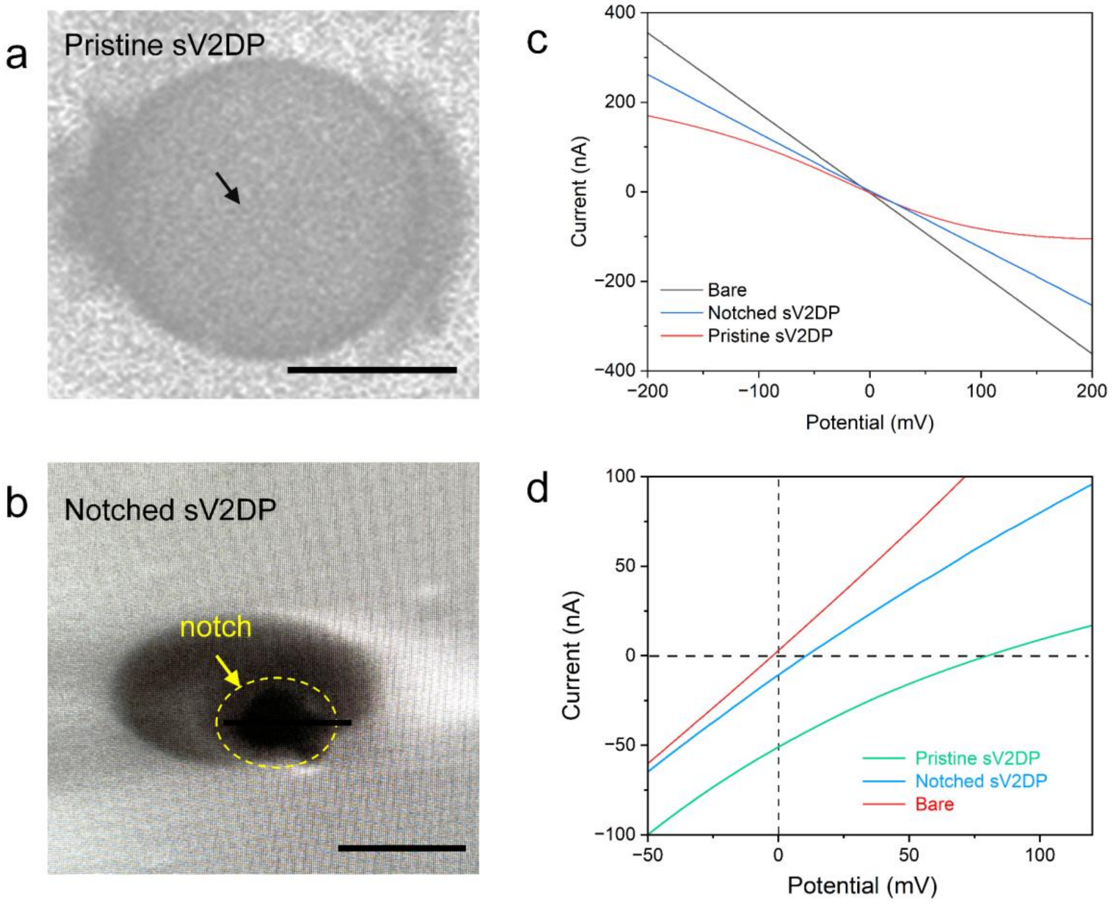

Supplementary Fig. 26| Intentional-defect (notch) control to probe leakage-sensitive transport. a, **b**, SEM images of representative devices: pristine and notched sV2DP membrane samples. Scale bar, 1 µm. **c**, *I-V* curves of pristine and notched sV2DP membranes, and a bare aperture substrate in 0.1 M KCl solutions, showing that the conductance follows the order: bare substrate (1.79 µS) > notched sV2DP (1.25 µS) > pristine sV2DP (1.10 µS). **d**, Corresponding *I-V* under 0.5/0.01 M KCl. The notched sV2DP exhibits *I-V* behavior shifted toward the bare-substrate response, consistent with leakage-dominated transport and a loss of ionic selectivity.

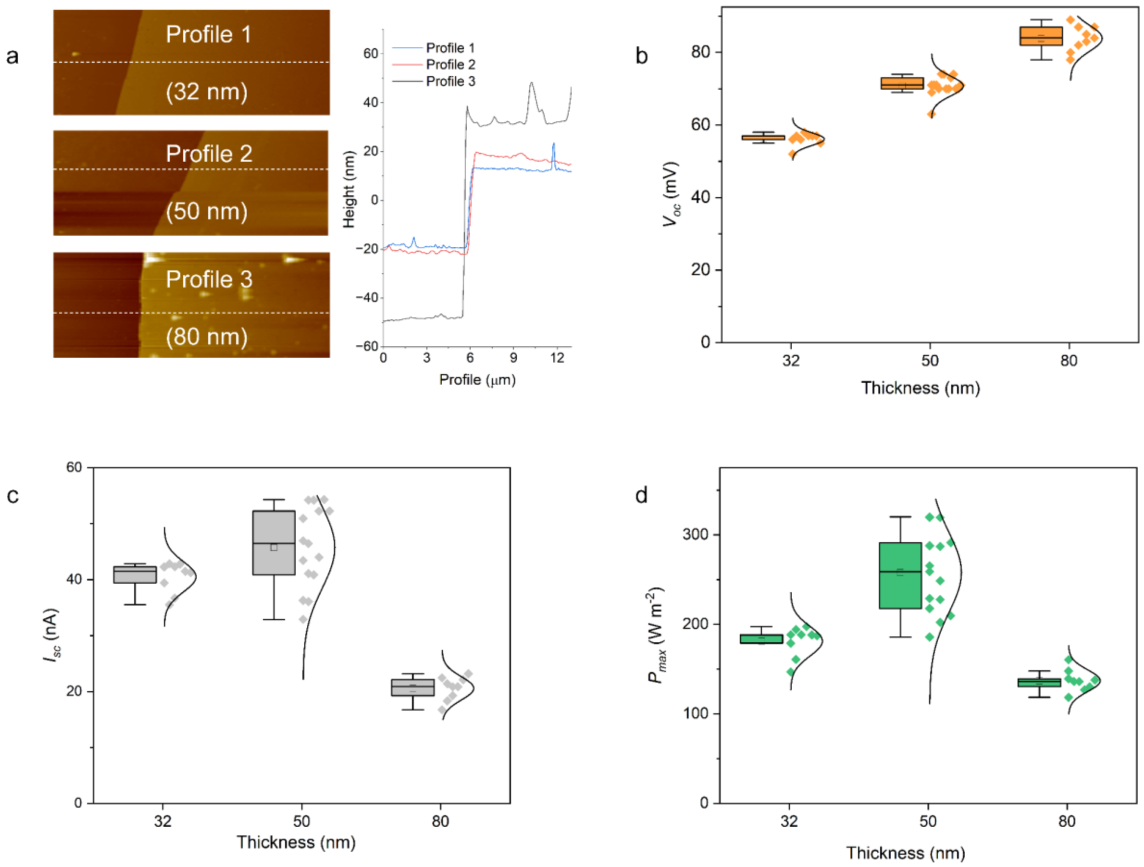

Supplementary Fig. 27| Thickness-dependent performance of sV2DP membranes. a, Representative AFM images and corresponding height profiles used to determine membrane thicknesses of ~32, 50, and 80 nm. Scale bar: 3 μm. **b-d**, Thickness dependence of key osmotic-energy metrics, (b) V_{oc}, (c) I_{sc}, and (d) P_{max}, all evaluated under 0.5/0.01 M KCl gradients. Individual data points are shown alongside the box plots, and half-violin plots indicate the data distribution. Boxes indicate the 25th-75th percentiles, center lines indicate the median, and whiskers extend to values within 1.5× the interquartile range. For 32 and 80 nm samples, n = 9 independent measurements; for 50 nm samples, n = 9 independent measurements

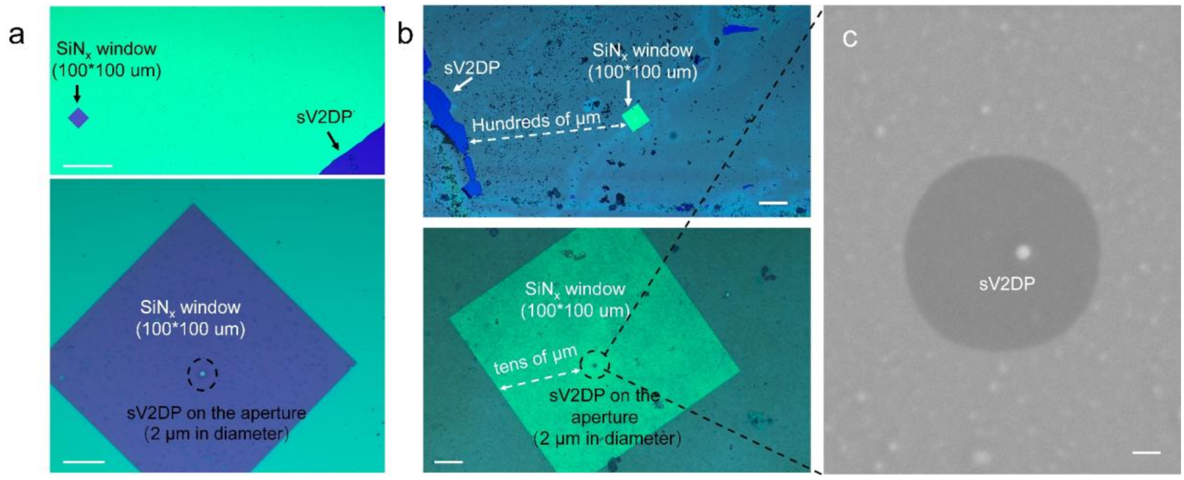

Supplementary Fig. 28| Pre- and post-measurement imaging of the sV2DP devices. a, Optical images of the device before measurement. Scale bars: 100 µm (upper) and 10 µm (bottom). **b**, Optical images of the device after measurement. Scale bars: 100 µm (upper) and 10 µm (bottom). **c**, SEM image of the device after measurement. Scale bars: 200 nm.

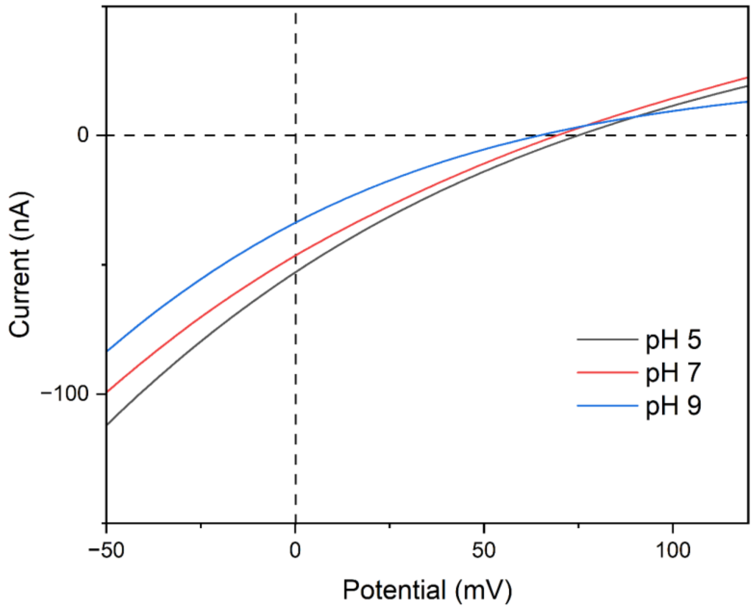

Supplementary Fig. 29| pH-dependent ion-selective transport of the sV2DP membrane. Ion-selective measurements of the sV2DP membrane were performed at pH 5, 7, and 9 under the 0.5/0.01 M KCl gradients.

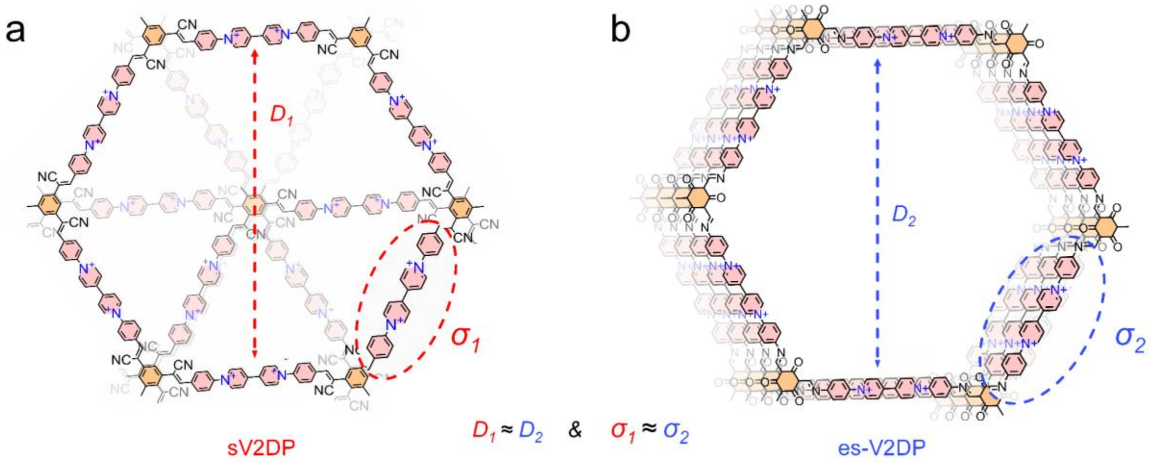

Supplementary Fig. 30| Schematic illustration of the chemical structures. a, sV2DP and **b**, es-V2DP membranes, emphasizing the effect of interlayer ABC stacking. D_1 and D_2 denote the lattice dimensions (~ 44 **Å**) of the intrinsic monolayer frameworks, while σ_1 and σ_2 refer to the theoretical overall charge densities.

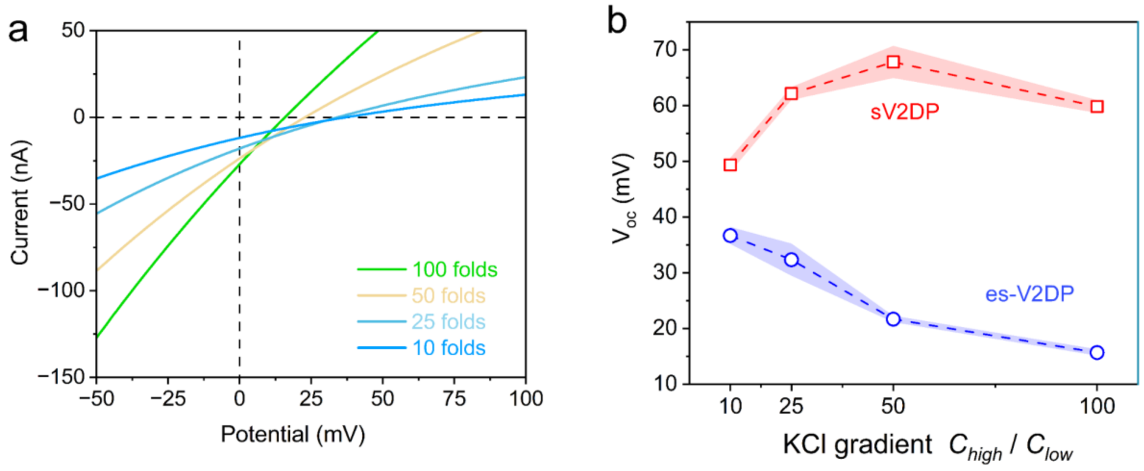

Supplementary Fig. 31| Comparison of ion selectivity between sV2DP and es-V2DP membranes. **a**, *I–V* curves of the es-V2DP membrane measured under varying KCl gradients (10- to 100-fold). **b**, Variations in V_{oc} as a function of KCl gradients, with squares representing sV2DP and circles representing es-V2DP. Data are presented as mean ± SD from n = 3 independent measurements. The shaded areas indicate SD.

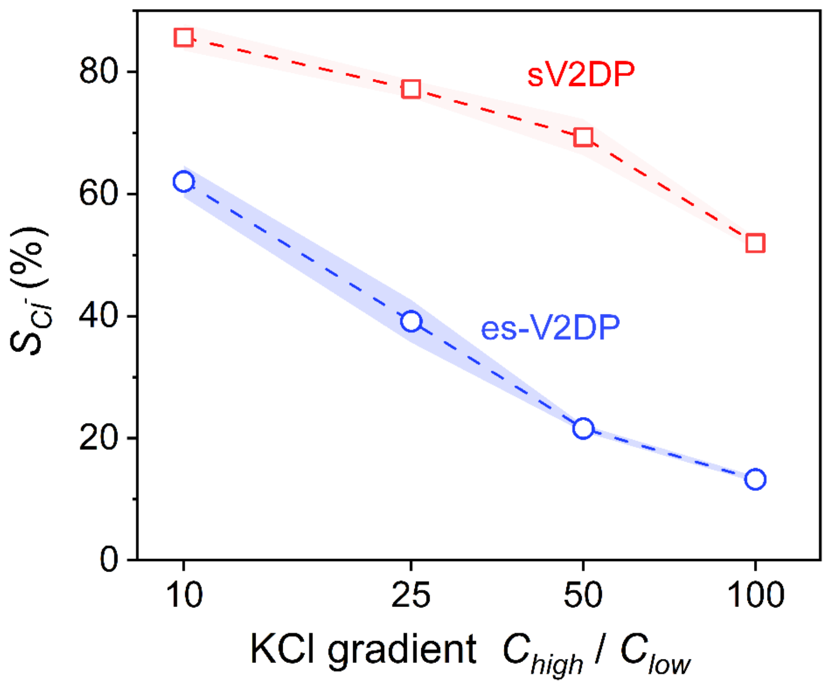

Supplementary Fig. 32| Comparison of chloride selectivity between sV2DP and es-V2DP membranes. S_{Cl^-} values of sV2DP and es-V2DP membranes measured under different KCl concentration gradients. Data are presented as mean ± SD from n = 3 independent measurements. The shaded areas indicate SD.

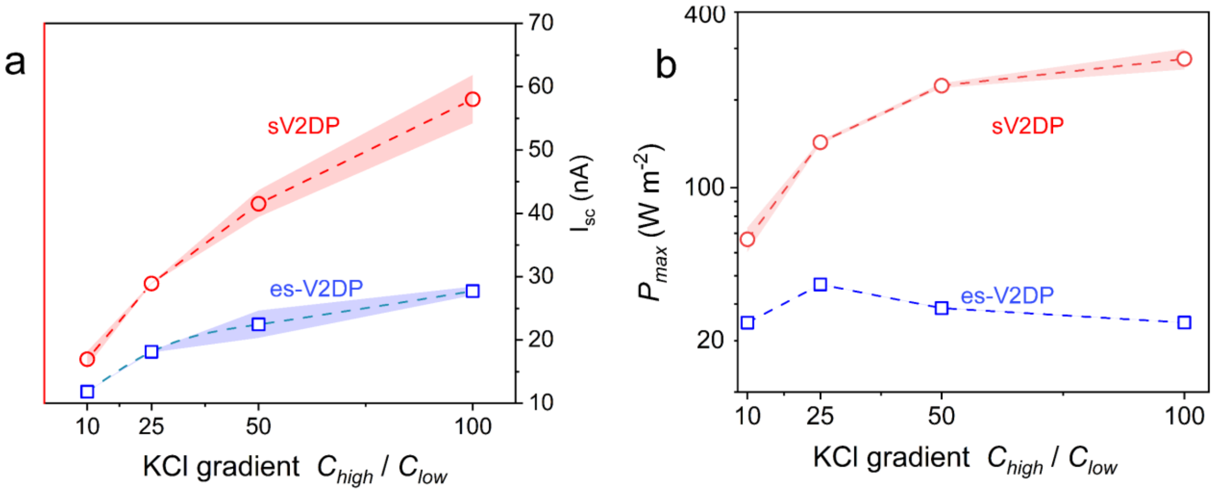

Supplementary Fig. 33| Comparison of osmotic current and power output between sV2DP and es-V2DP membranes. a, I_{sc} values of sV2DP and es-V2DP membranes measured as a function of KCl concentration gradient. **b**, Corresponding P_{max} values obtained from sV2DP and es-V2DP membranes under the same KCl gradients**.** Data are presented as mean ± SD from n = 3 independent measurements. The shaded areas indicate SD.

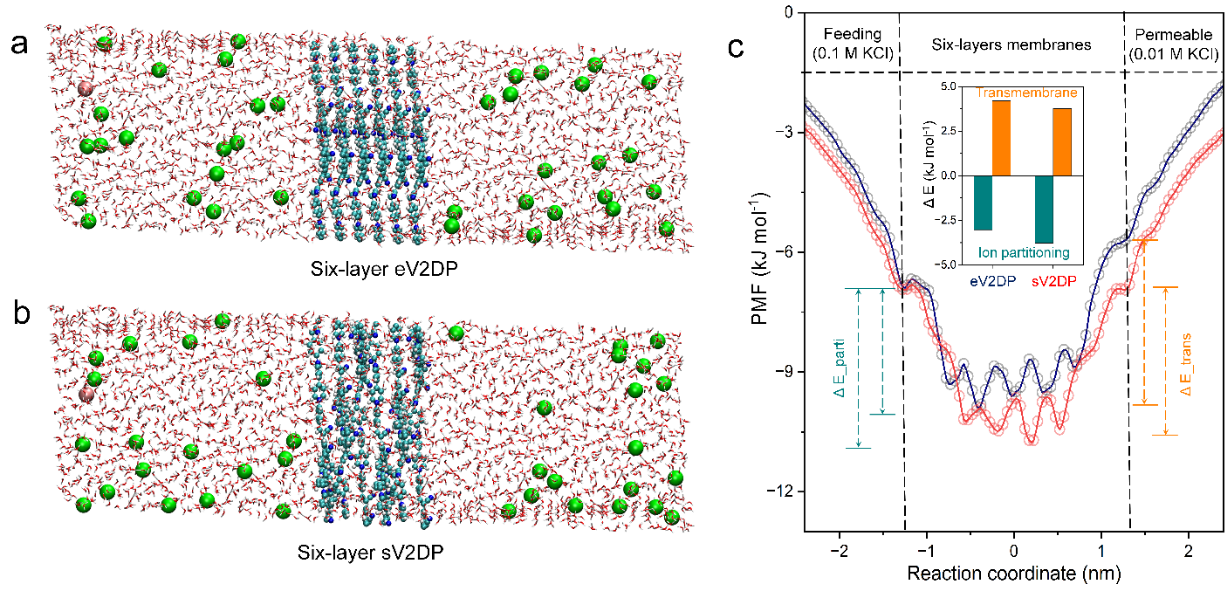

Supplementary Fig. 34| Free energy landscape of Cl^- transport across sV2DP and eV2DP membranes. a-b, MD simulation setups: six-layer-thick eV2DP (**a**) and sV2DP (**b**) membranes are positioned between a 0.1 M KCl feed solution and a 0.01 M KCl permeate solution, allowing both K^+ and Cl^- ions to traverse the membrane structures. **c**, 1D PMF profiles for Cl^- ions translocating across sV2DP and eV2DP membranes under a 0.1/0.01 M KCl gradient. Inset: Energy-barrier decomposition reveals that sV2DP facilitates more favorable partitioning (green) and lower exit resistance (red), collectively contributing to its lower transport barrier.

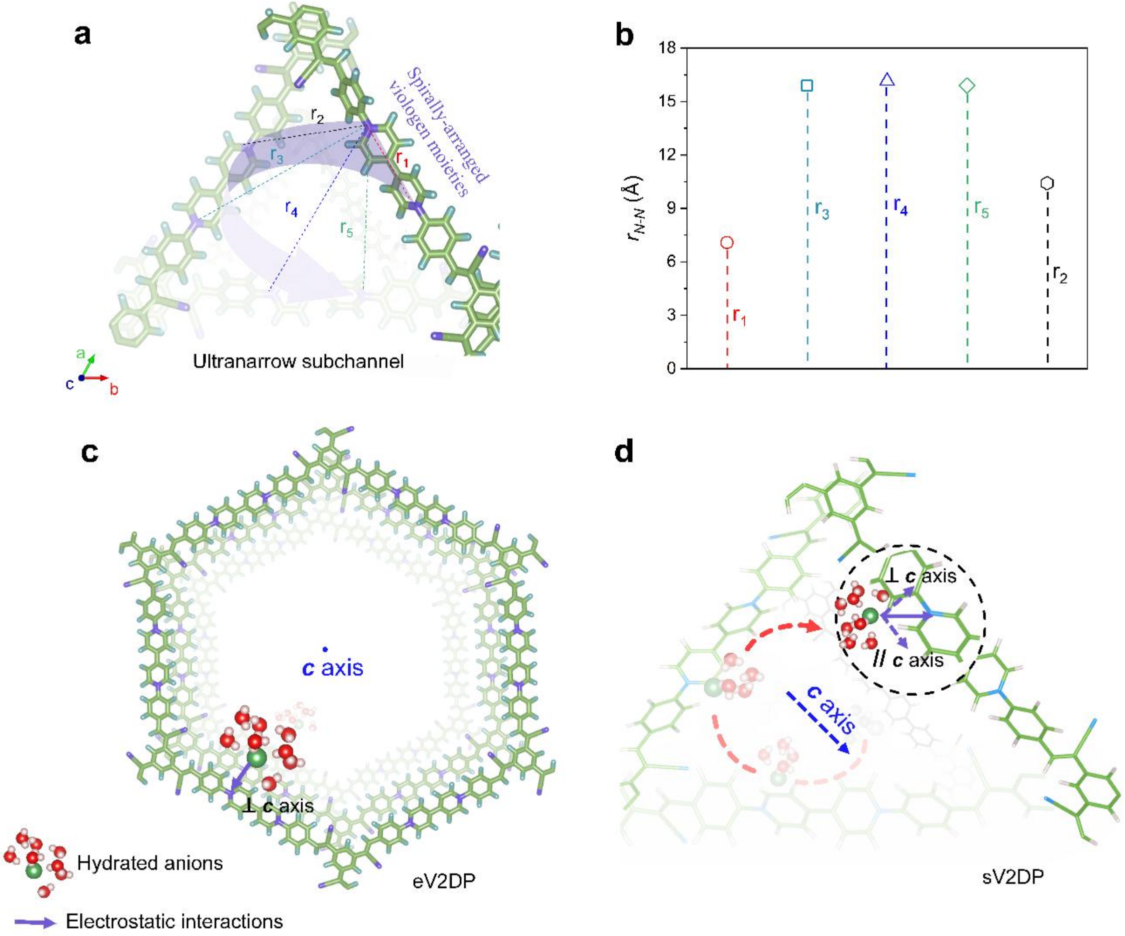

Supplementary Fig. 35| Structural origin of directional anion migration in sV2DP nanochannels. **a**, Schematic illustration of the ultranarrow geometry and spirally arranged pyridinium sites within the sV2DP channels. **b**, Interatomic distances between adjacent pyridinium sites ($r_{N\text{-}N}$) in sV2DP, ranging from 10.4 to 16.1 Å. **c-d**, Schematic representations of the interactions between hydrated anions and pyridinium sites in the V2DP membrane: (c) eV2DP and (d) sV2DP. The spiral distribution of pyridinium sites in sV2DP introduces an additional axial electrostatic field component (// ***c*** axis), which serves as a built-in guiding force to promote directional anion migration along the ***c***-axis.

MD Simulations for anion transport pathway across the membranes

To simulate the transmembrane behavior of anions across the sV2DP membrane, nine sV2DP layers were placed in a simulation box with dimensions of 45.603 Å × 43.687 Å × 33.072 Å. A total of 54 anions were positioned within the membranes near the adsorption sites (pyridine N). The optimal positions of the anions were determined by structural optimization using the Density Functional Tight Binding (DFTB) method with the 3ob-3-1 parameter set and UFF dispersion. Additionally, 579 water molecules were added to fill the simulation box. The MD simulations were performed in the canonical ensemble (NVT) at a controlled temperature of 300 K using the Nose-Hoover method. The damping constant was set to 2 ps to minimize artifacts and ensure the natural interaction of components within the system. The simulations employed a time step of 0.25 fs and ran for a total of 250 ps. Separate simulations were conducted for each type of anion to collect corresponding data.

To investigate the interaction behavior between anions and the absorption sites (pyridine N), MD simulations with dense sampling were performed to obtain trajectory files. Nine layers of the VS2DP membrane were placed in a simulation box with dimensions of 45.603 Å × 43.687 Å × 33.072 Å. A total of 54 anions were introduced near the adsorption sites, and 579 water molecules were added to fill the simulation box. The MD simulations were conducted in the canonical ensemble (NVT) at 300 K using the Nose-Hoover method, with a damping constant of 2 ps to enhance system accuracy. A time step of 0.25 fs was used, and the simulations ran for 15 ps. Trajectory data were recorded every 2.5 fs. Separate simulations were performed for each type of anion to obtain comprehensive interaction data.

All simulations were performed using the Amsterdam Modeling Suite (AMS 2024), with parameters derived from the UFF4MOFII force field. TIP3P model for water and Joung–Cheatham parameters for halide ions were used.[26] The Joung-Cheatham ion parameters were specifically optimized to reproduce key thermodynamic and structural properties (e.g., hydration free energies and crystal lattice properties) in combination with TIP3P water. As an internal consistency check relevant to ion hydration structure, we computed radial distribution functions *g(r)* for each halide ion in bulk aqueous solution and extracted the corresponding first-shell coordination numbers (Supplementary Fig. 32). The resulting coordination numbers are in good agreement with reported experimental values (Supplementary Table 4), supporting that the ion-water hydration environment is described reasonably within our simulation framework. Visualization of trajectory files and anion motion tracking were carried out using VMD 1.9.3. Analysis of trajectory files, including the extraction of interaction statistics

between anions and adsorption sites, was conducted using the MD Analysis package. The cut-off distance of interaction between the anions and absorption sites was set as 5.6 Å. For each step, the number of anions that fell in the interaction range within the adsorption sites was counted. Kernel Density Estimation (KDE) was used to evaluate the frequency of interaction numbers between anions and adsorption sites.

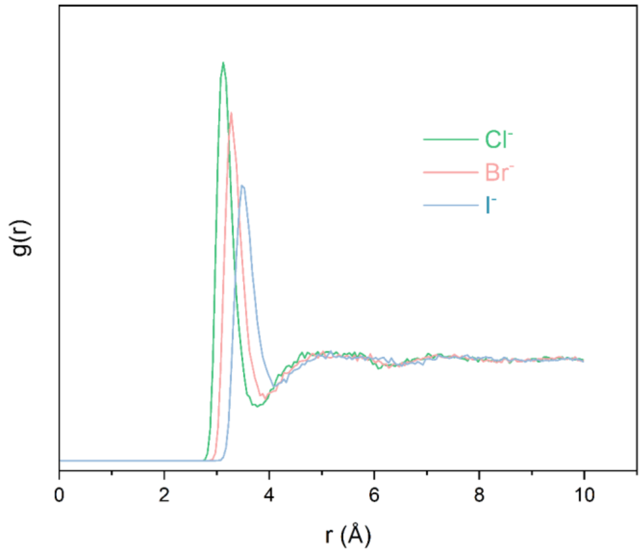

Supplementary Fig. 36| Hydration structures of halide ions in aqueous solution. Radial distribution functions (*g(r)*) between halide ions (Cl^-, Br^-, I^-) and water oxygen atoms in aqueous solution computed using TIP3P water and Joung-Cheatham ion parameters. The first-peak positions (in Å) increase from Cl^- to Br^- to I^-, consistent with the expected trend in hydration structure.

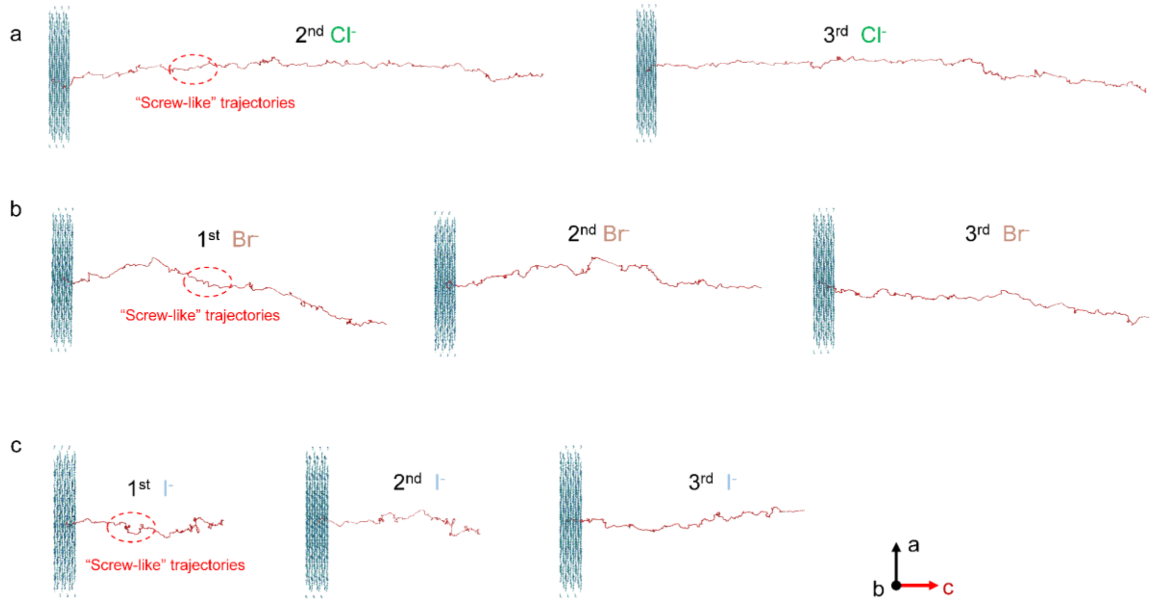

Supplementary Fig. 37| Trajectory visualizations of halide-ion transport in sV2DP. Representative trajectories of (a) Cl^-, (b) Br^-, and (c) I^- obtained from three independent MD runs in the sV2DP channel. The red traces show the projected ion transport paths, highlighting reproducible 'screw-like' motion along the c-axis.

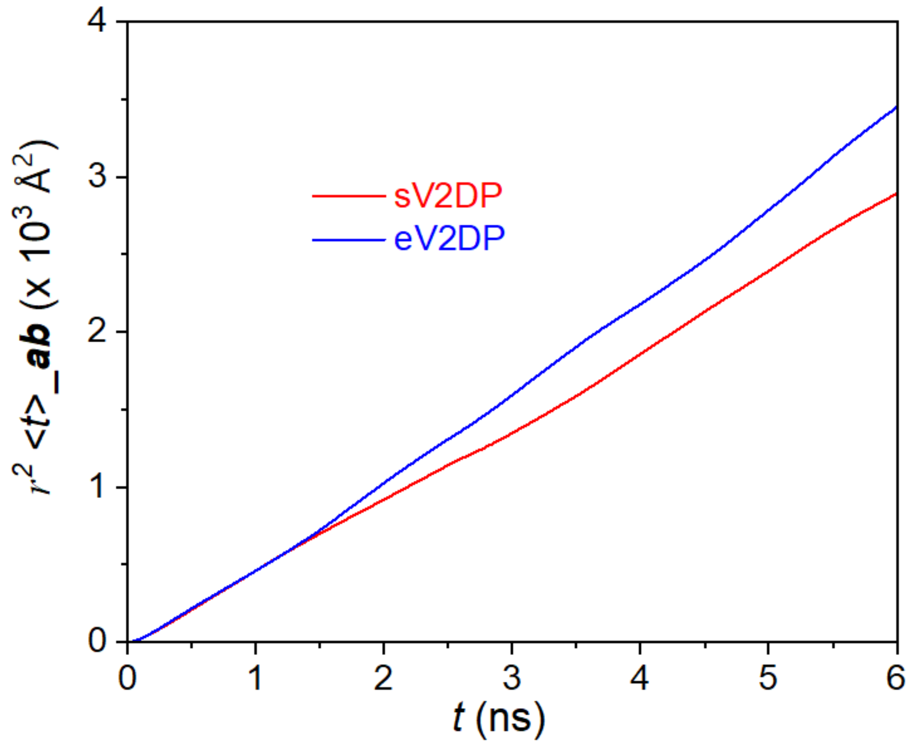

Supplementary Fig. 38| In-plane diffusion of chloride ions in sV2DP and eV2DP structures. Calculated MSD curves of Cl^- along the ***ab*** plane (r^2<*t*>_***ab***) in sV2DP versus eV2DP

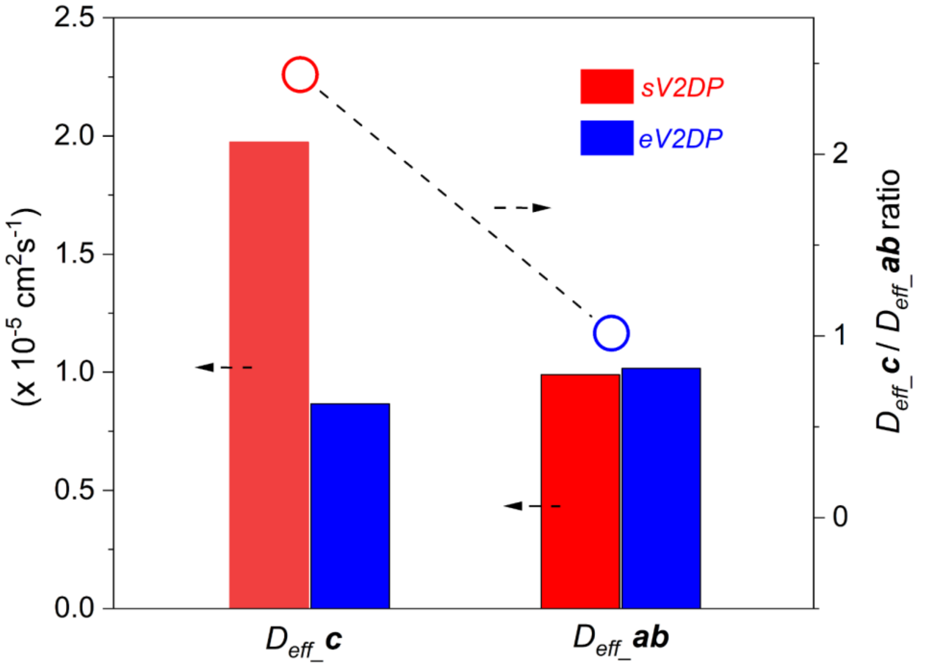

Supplementary Fig. 39| Directional diffusion analysis of Cl^- ions in sV2DP and eV2DP membranes. Comparison of effective diffusion coefficients of Cl^-ions in sV2DP and eV2DP membranes calculated from MSD results.

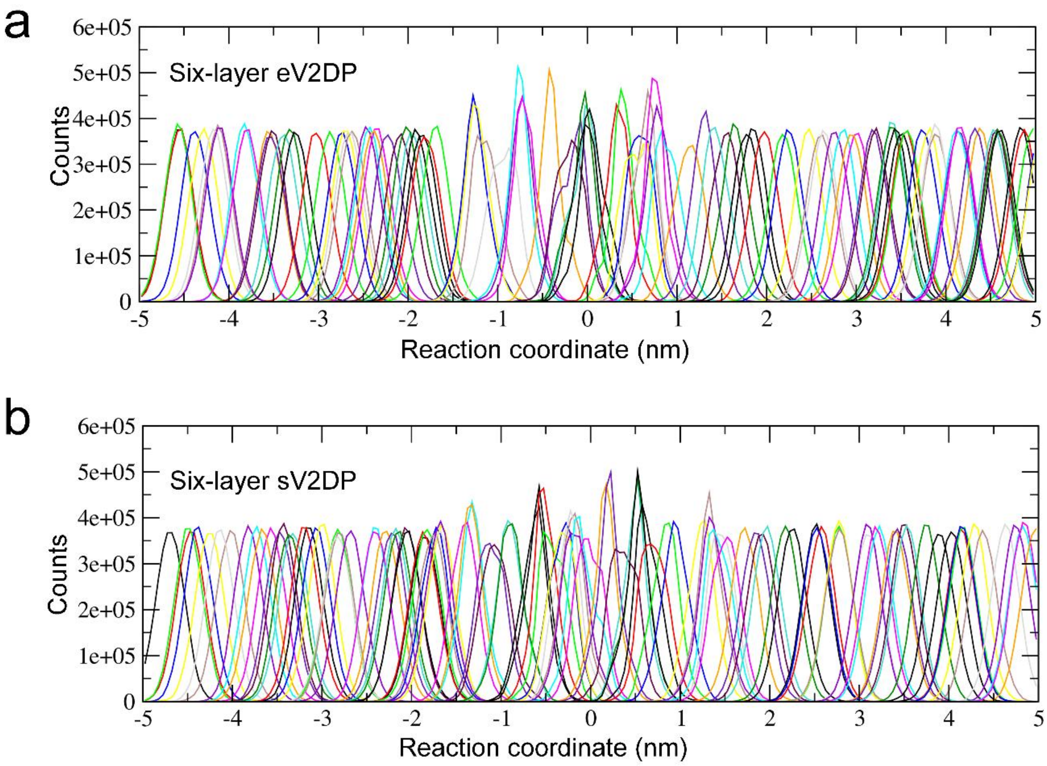

Supplementary Fig. 40| Umbrella-sampling windows for PMF calculations of Cl^- transport. Umbrella sampling employed to calculate the 1D PMF for Cl^- ions across the **a**, six-layer eV2DP and **b**, six-layer sV2DP.

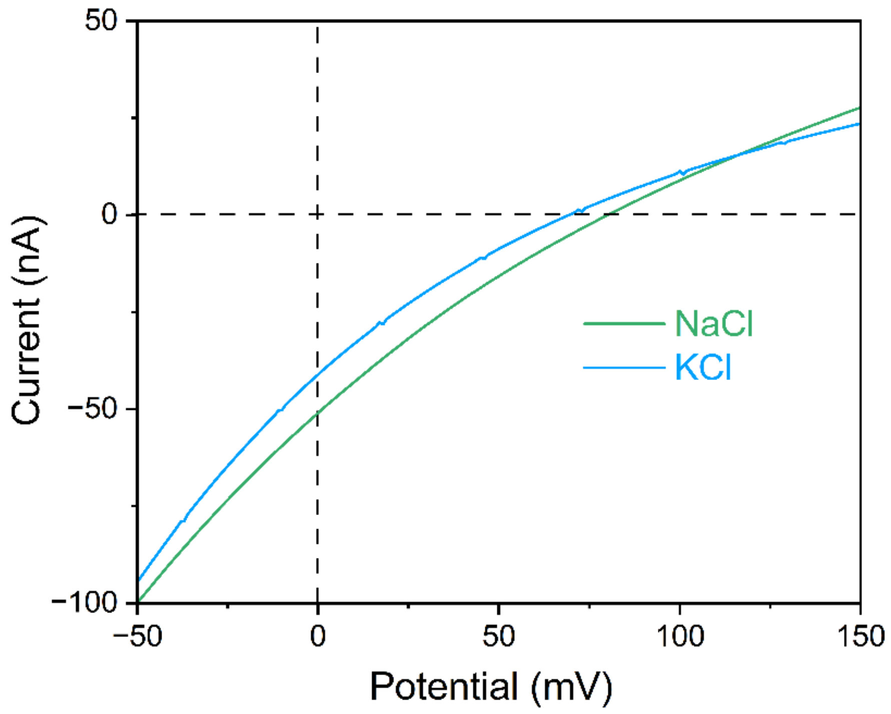

Supplementary Fig. 41| Osmotic current generation of sV2DP membranes in NaCl and KCl gradients. *I-V* curves of the sV2DP membrane in 50-fold NaCl and KCl, respectively.

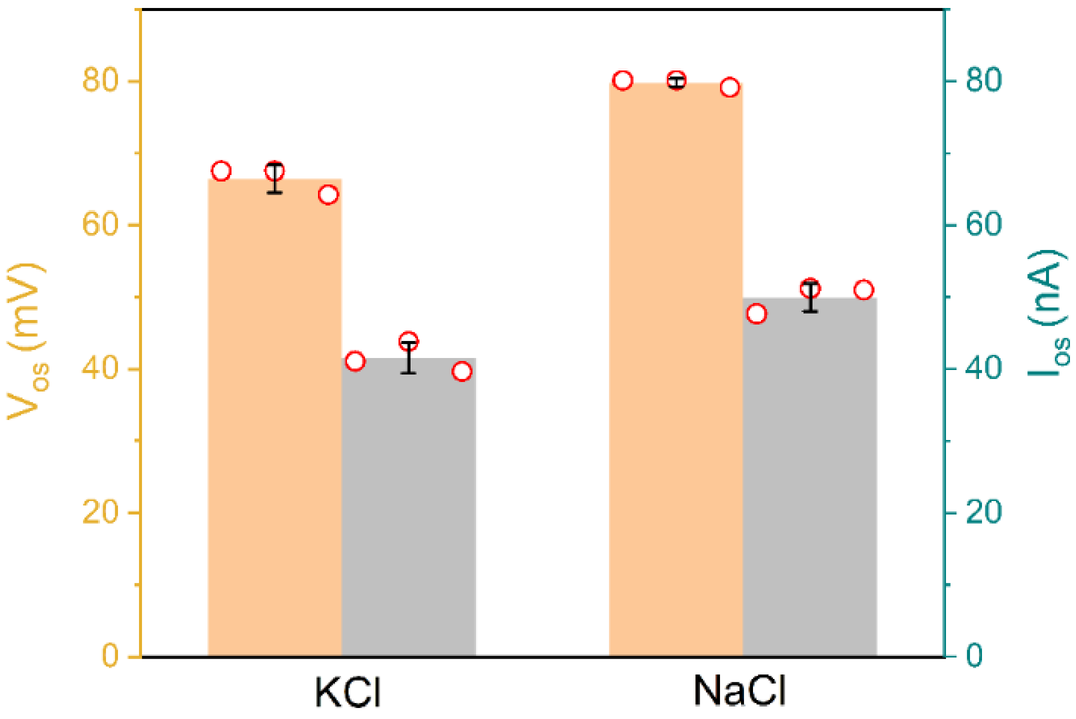

Supplementary Fig. 42| Comparison of osmotic performance of sV2DP membrane in KCl and NaCl gradients. Comparison of the V_{os} and I_{os} in NaCl and KCl solutions with a 50-fold concentration gradient. Data are mean ± SD from n = 3 independent measurements.

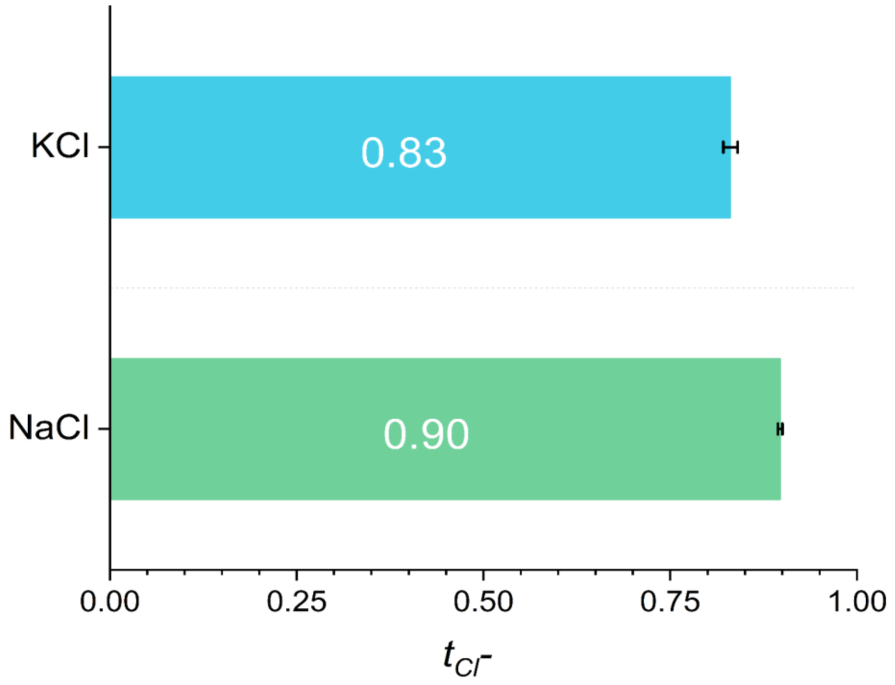

Supplementary Fig. 43| Chloride transference numbers of sV2DP membranes in KCl and NaCl gradients. Comparison of the t_{Cl^-} values in NaCl and KCl solutions with a 50-fold concentration gradient. Data are mean ± SD from n = 3 independent measurements.

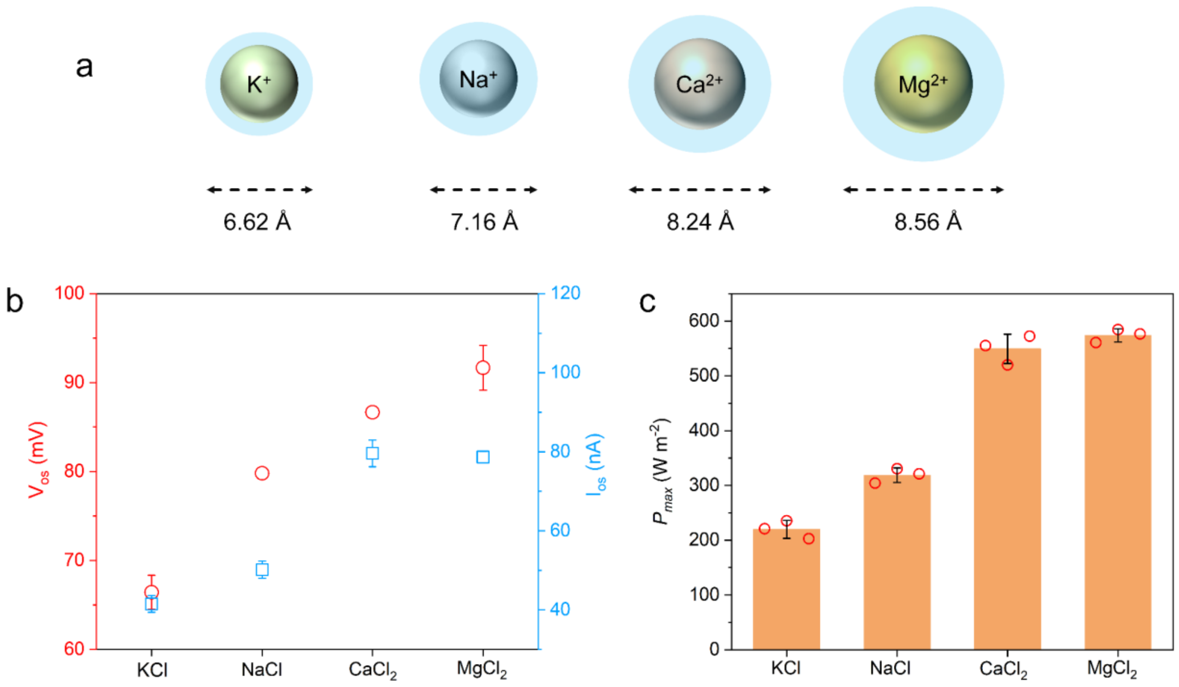

Supplementary Fig. 44| Multivalent-cation effects on osmotic performance of sV2DP. a, Schematic comparison of hydrated diameters for common seawater cations (K^+, Na^+, Ca^{2+} and Mg^{2+}). **b**, Comparison of the V_{os} and I_{os} of the sV2DP membrane under various 50-fold electrolyte gradients (KCl, NaCl, $CaCl_2$, and $MgCl_2$). c, P_{max} values derived from (b). Data are mean ± SD from n = 3 independent measurements in (b) and (c).

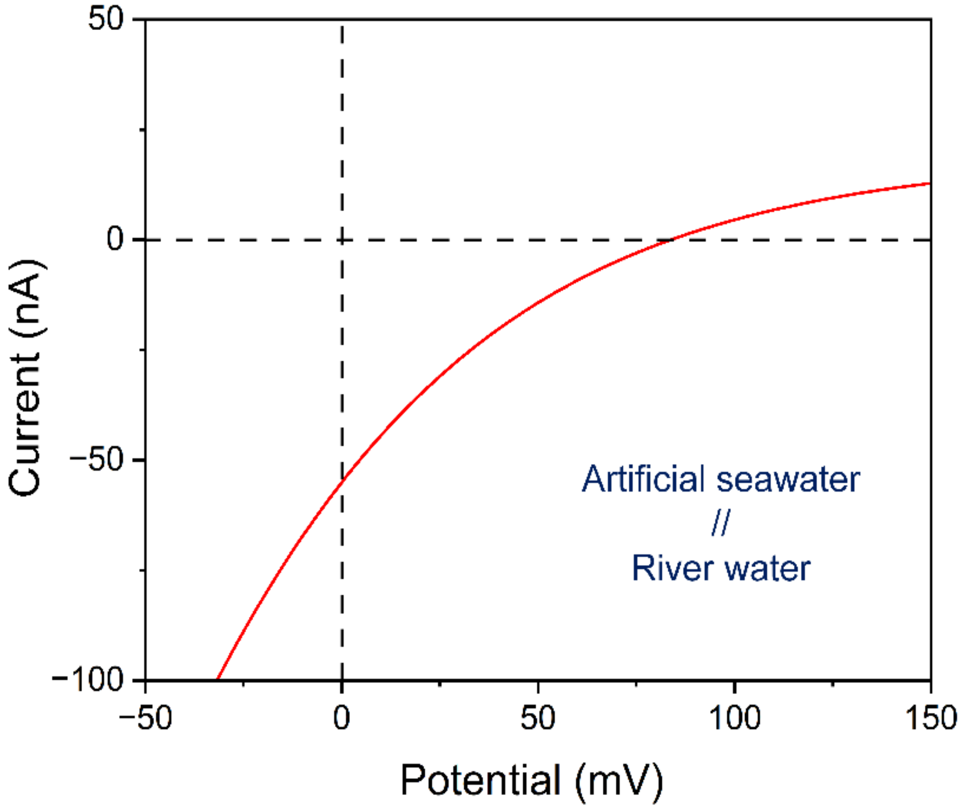

Supplementary Fig. 45| Osmotic current generation of sV2DP membranes under artificial seawater/river water mixing. *I-V* curves of the sV2DP membrane by mixing the artificial seawater and river water.

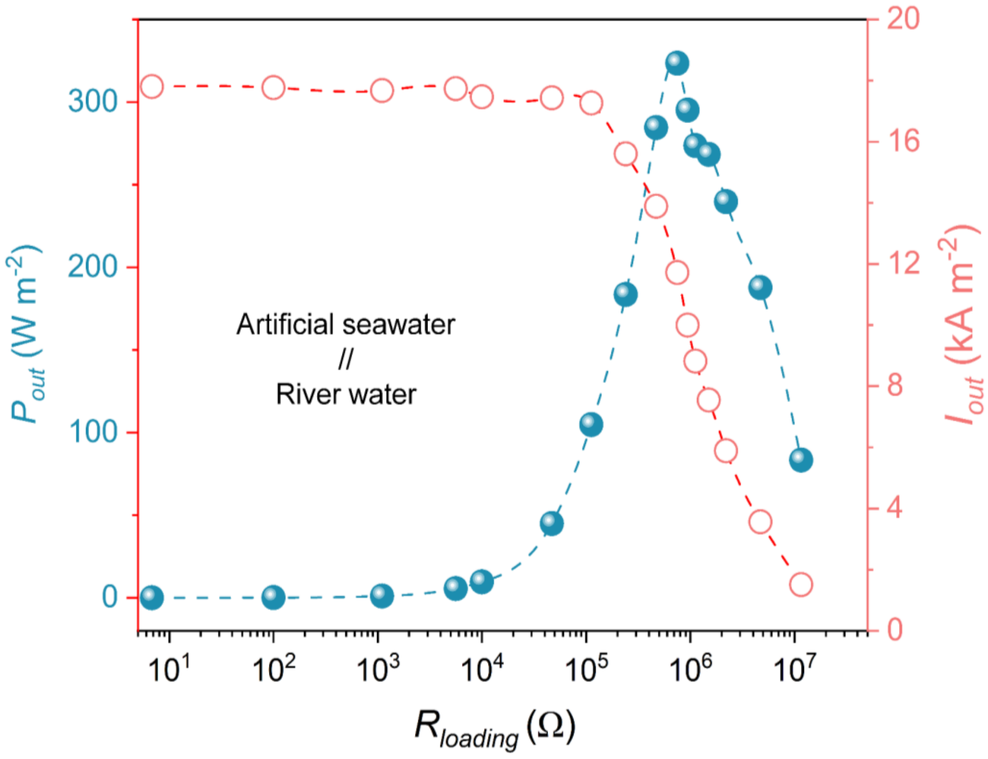

Supplementary Fig. 46| External-load-dependent power output of sV2DP membranes under artificial seawater/river water mixing. P_{out} on the external circuit from the artificial seawater and river water mixing.

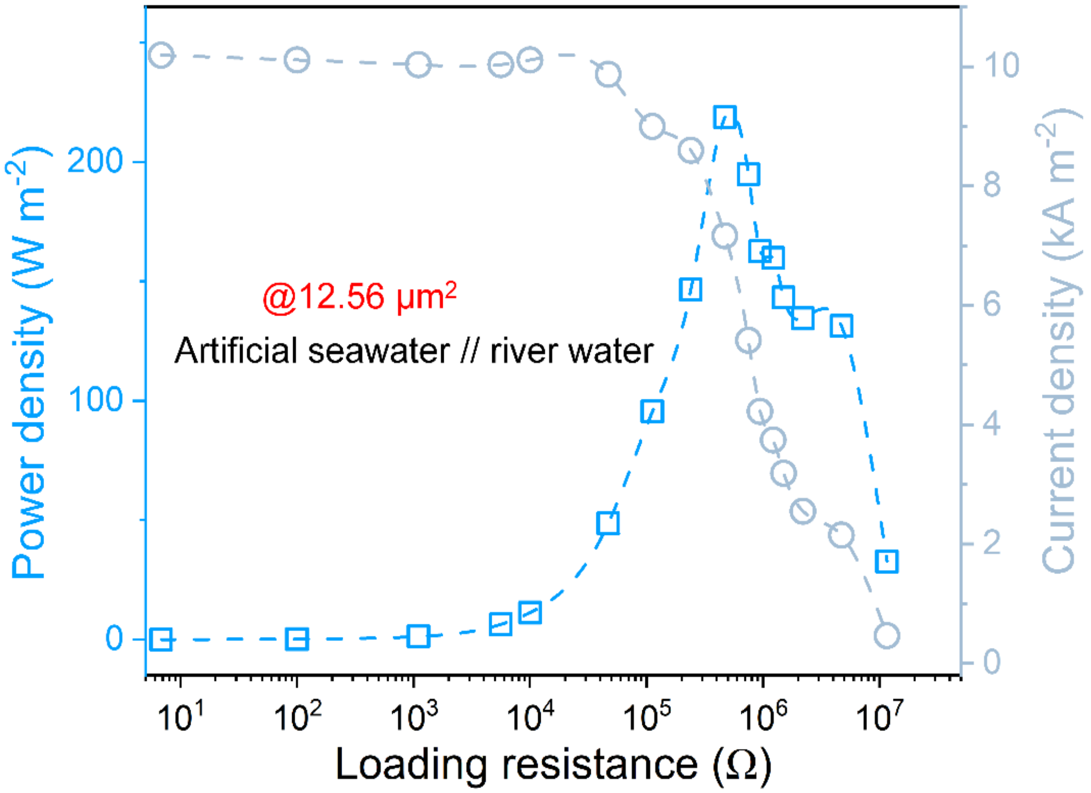

Supplementary Fig. 47| Power output of a 12.56-µm^2 sV2DP membrane under artificial seawater/river water mixing. P_{out} of a 12.56-µm^2 sV2DP membrane in an external circuit by mixing the artificial seawater and river water.

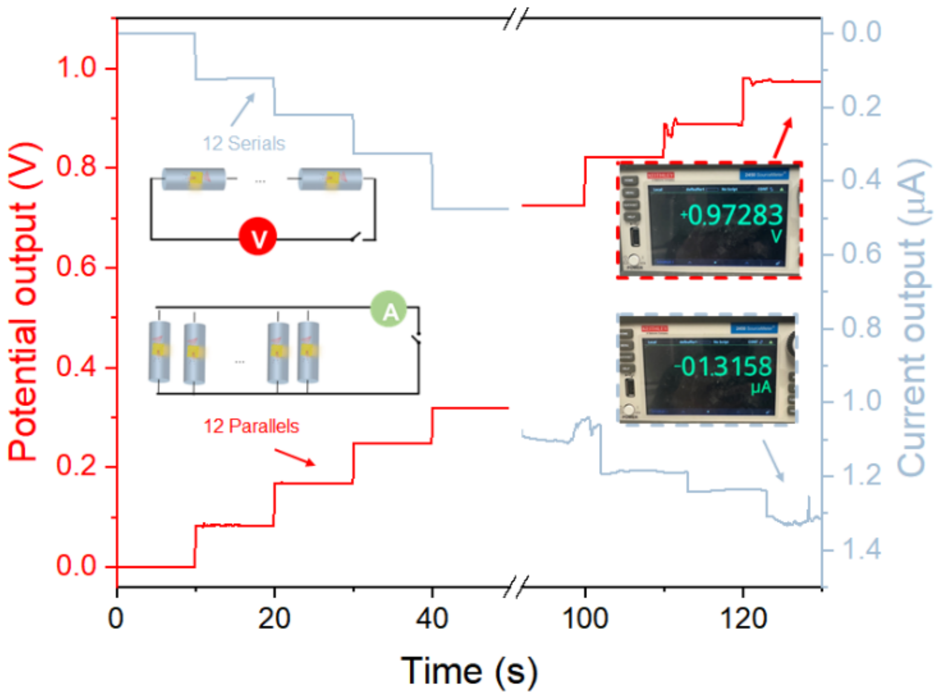

Supplementary Fig. 48| Tandem integration of sV2DP-based osmotic power generators. *V*-*T* and *I*-*T* curves of tandem configurations with 12-unit sV2DP-based systems.

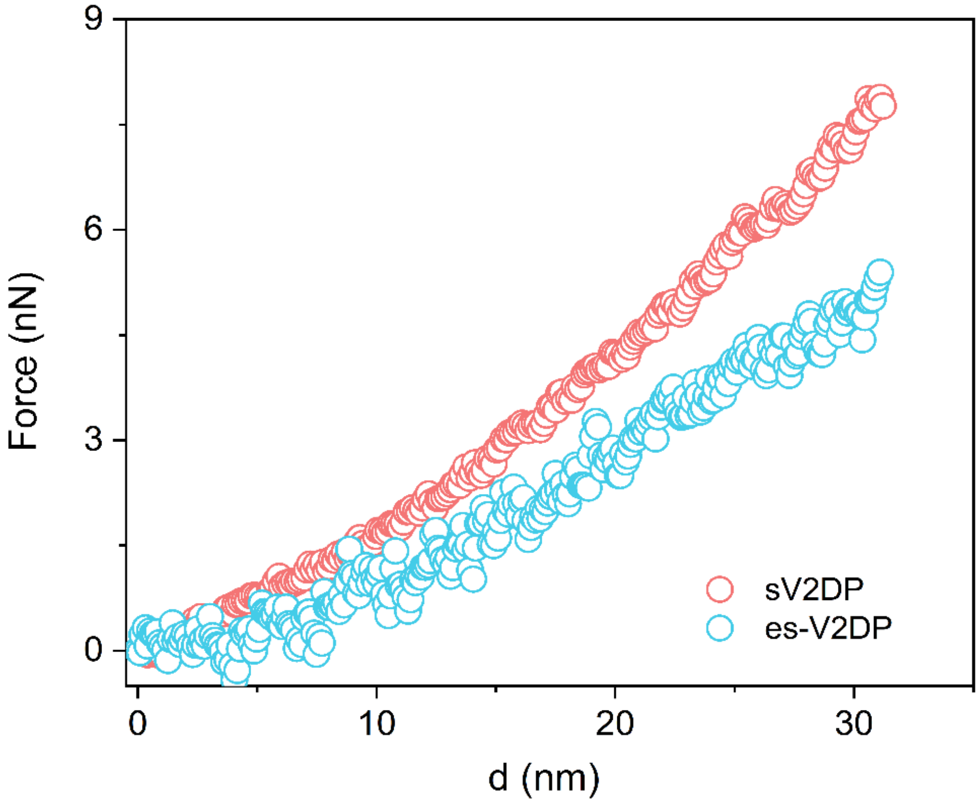

Supplementary Fig. 49| Nanoindentation testing for the resultant sV2DP and es-V2DP membranes. Representative force–displacement curves obtained from nanoindentation measurements of sV2DP and es-V2DP membranes.

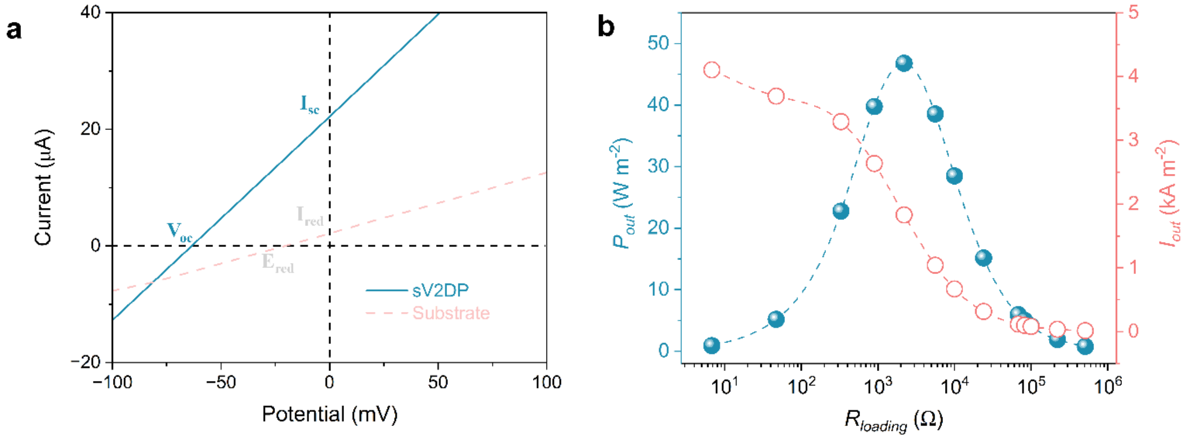

Supplementary Fig. 50| Osmotic power output of aperture-array sV2DP membrane devices. **a**, *I*-*V* curves of the sV2DP membrane measured between artificial seawater and river water. Due to the significantly reduced system resistance from scaled-up pores, the intrinsic resistance of commercial Ag/AgCl electrodes with KCl salt bridges becomes non-negligible. To ensure accurate measurements, a pair of self-made AgCl electrodes was used. The V_{os} and I_{os} were obtained by subtracting baseline values (E_{red} and I_{red}) measured with the bare substrate. **b**, P_{out} of the aperture-array sV2DP-based OPG under an artificial seawater/river water salinity gradient.

Evaluation of system resistance in single-pore and array devices.

The non-linear decrease in the total resistance with increasing testing area (A) leads to reduced device performance, consistent with previous reports.[27] In general, R_{total} can be expressed as an equivalent circuit consisting of the reservoir resistance (R_{res}), the reservoir/membrane interface resistance (R_{inter}) and the membrane resistance (R_{mem}) (inset of Supplementary Fig. 47a). In single-pore devices (A = 3.14 μm^2), R_{mem} dominates R_{total} at the MΩ level, and the resulting performance is therefore mainly governed by the intrinsic properties of the tested membrane. With the array device (A = 6,358.5 μm^2), R_{total} decreases substantially (from 1.58 MΩ to 2.8 kΩ) due to the reduction in R_{mem}, becoming comparable to the R_{res} and R_{inter} (Supplementary Fig. 51a). Consequently, the contributions from the reservoirs and interfaces become increasingly significant. According to $P = V_{oc}^2/R_{total}$, the measured device performance therefore becomes less representative of the intrinsic membrane properties, accompanied by a decrease in P_{max} from 258 to 48 W m^{-2} (Supplementary Fig. 51b). In this work, we focus mainly on the intrinsic performance of the sV2DP membranes, while system-level optimization will be explored in future studies.

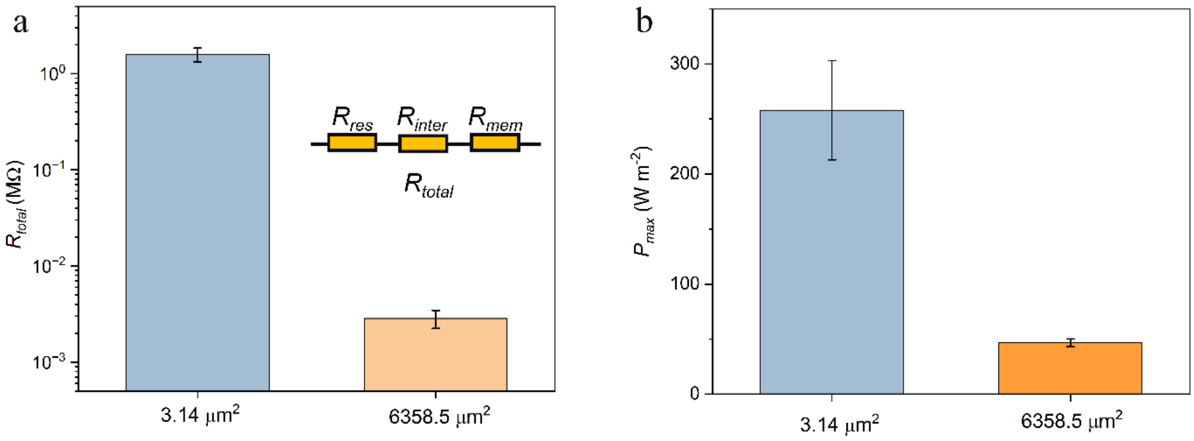

Supplementary Fig. 51| Evaluation of system resistance in single-pore and array-pore devices. a, Measured R_{total} for sV2DP devices with single- (A, 3.14 μm^2) and array-pore (A, 6,358.5 μm^2) substrates. **Inset:** Schematics of R_{total}, which contains R_{res}, R_{inter}, and R_{mem}. **b,** P_{max} values for the resulting systems under 0.5/0.01 M KCl gradients. Data are presented as mean ± SD from n = 15 independent measurements for single-pore devices and n = 3 independent measurements for array devices.

Defect density and continuity over large areas.

To directly assess defect density, we have performed high-resolution transmission electron microscopy (HRTEM) imaging to visualize grain-boundary and defect regions in our sV2DP membrane. As shown in Supplementary Fig. 48a, these non-ideal regions exist between crystalline domains and account for less than 5% of the total area. Compared to the intrinsic hexagonal lattice, these defects likely act as relatively open, less ion-selective nanoscale pathways (e.g., larger-pore-area 7-membered lattice motifs), providing a substantially larger effective cross-sectional area for ion transport (Supplementary Fig. 48 b,c).

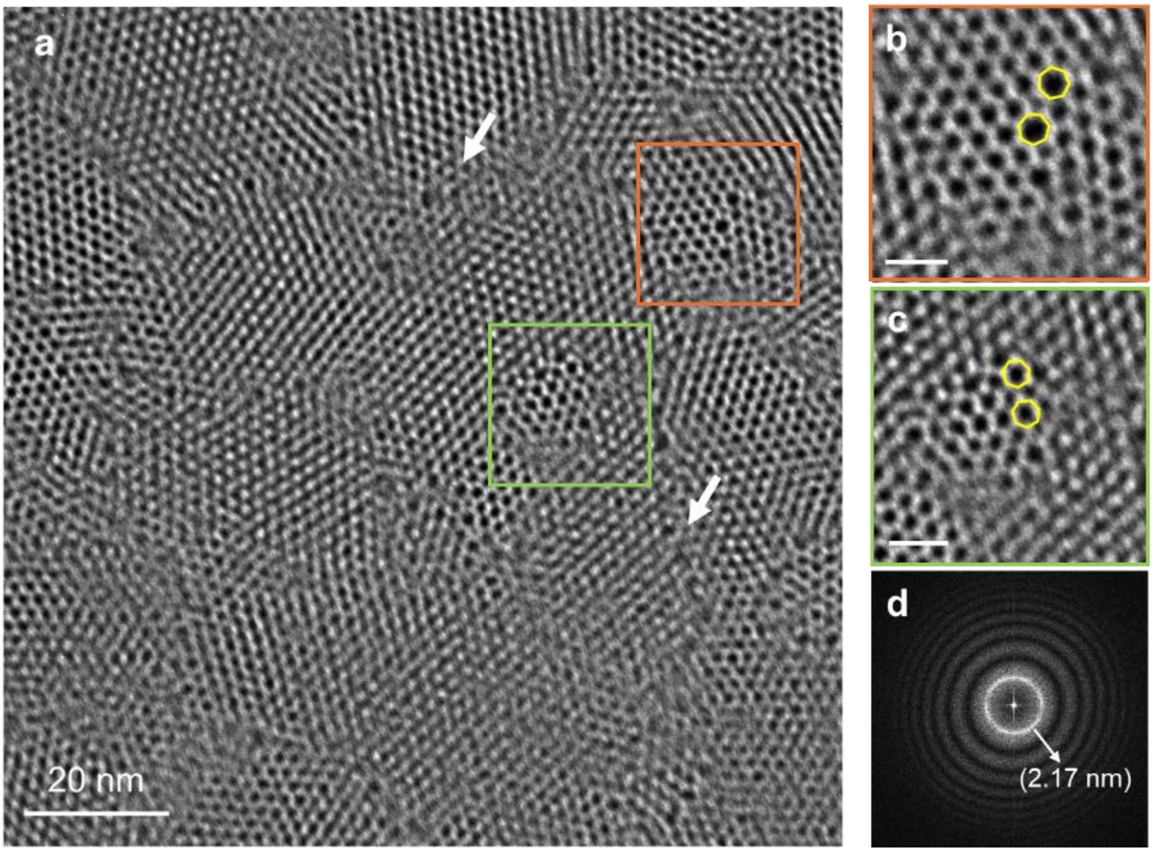

Supplementary Fig. 52| Grain boundaries and defects in the sV2DP membrane. a, 300 kV Cs-corrected HRTEM image of the sV2DP membrane. The white arrow indicates an extended grain boundary. **b-c**, Regions highlighted by the orange and green boxes in (a) are magnified in (b) and (c), revealing larger-pore-area 7-membered rings (marked in yellow). **d**, Fast Fourier transform (FFT) of (a) showing a characteristic lattice spacing of 2.17 nm. Scale bars: 5 nm in (b) and (c).

Scaling of device performance with different effective active areas.

To gain more insight into the impact of defects on ion transport, we performed contrast measurements with different active areas on multiple samples. Importantly, the active areas investigated here were confined to the square-micrometre scale, thereby largely excluding the contribution of system-level resistance to the observed performance degradation. As the A increases from 0.76 μm^2 to 12.56 μm^2, the likelihood of including a higher fraction of non-ideal regions also increases. As a result, the measured V_{oc} values decrease from ~76 to ~61.8 mV, and the power density decreases from ~ 371 to ~135 W m^{-2}. This trend indicates that larger areas are more likely to include non-ideal regions, which dilute the average ion selectivity and output (Supplementary Fig. 49).

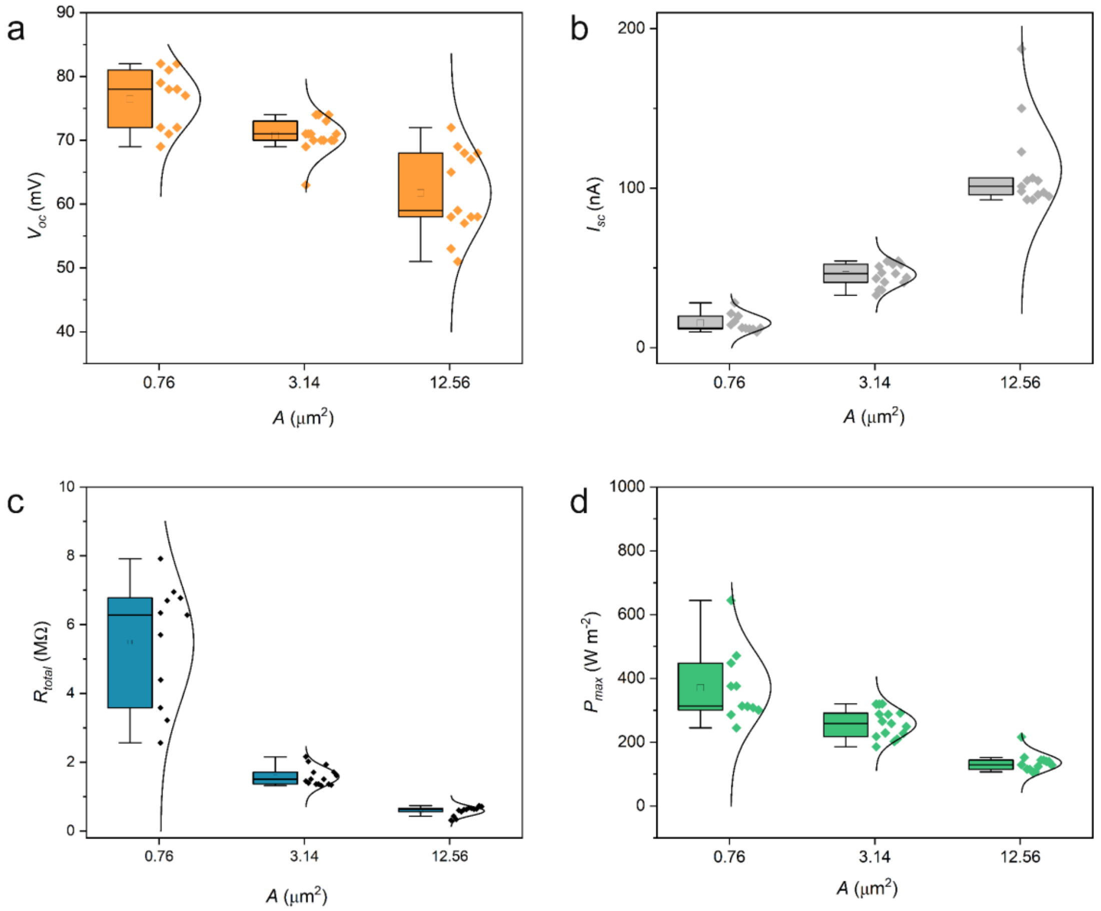

Supplementary Fig. 53| Scaling of device performance with effective active area under a fixed KCl gradient (0.5 M/0.01 M). Dependence of key output metrics on the total active area (A), including V_{oc}, I_{sc}, R_{total}, and P_{max}, on the effective active area under a fixed KCl gradient (0.5 M/0.01 M). (a) V_{oc}, (b) I_{sc}, (c) R_{total} and (d) P_{max}. Individual data points are shown alongside the box plots, and half-violin plots indicate the data distribution. Boxes indicate the 25th–75th

percentiles, center lines indicate the median, and whiskers extend to values within 1.5× the interquartile range. Sample numbers: n = 11 (0.76 μm^2), n = 15 (3.14 μm^2), n = 13 (12.56 μm^2).

Device-level defect-sensitivity test.

To further evaluate the influence of macroscopic defects on the array devices, we deliberately punctured the membrane in one aperture by FIB etching. This artifact caused the measured ionic selectivity to drop from 55% to 15.7%, approaching the level of the bare substrate and effectively short-circuiting the device (Supplementary Fig. 50). This strong sensitivity to such defects indicates that performance degradation upon scaling is dominated by microscopic defects and grain boundaries, rather than by external device- or system-level factors such as sealing or substrate support.

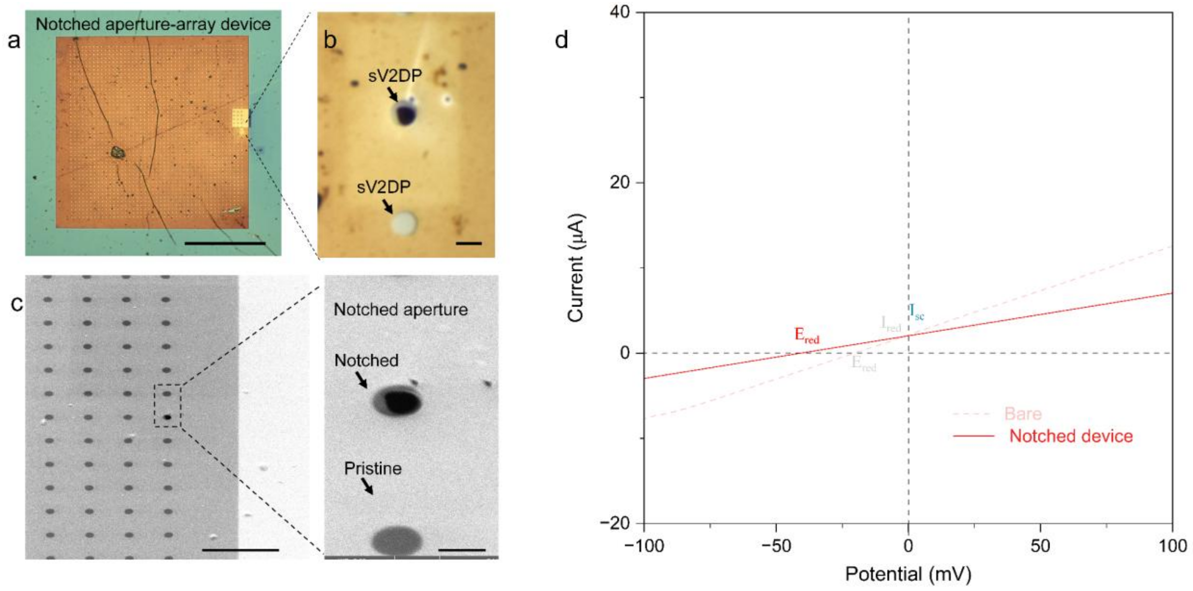

Supplementary Fig. 54| Device-level defect-sensitivity test. a, Optical image of the notched aperture-array device. Scale bar: 200 μm. **b**, Magnified view of the FIB-treated region, showing an intentionally notched membrane and a neighboring pristine membrane. Scale bar: 2 μm. **c**, SEM image of the FIB-notched region. Scale bar: 20 μm (left inset) and 2 μm (right inset). **d**, *I-V* curves of the notched device and the bare substrate, showing that introducing a local membrane defect strongly reduces ionic selectivity.

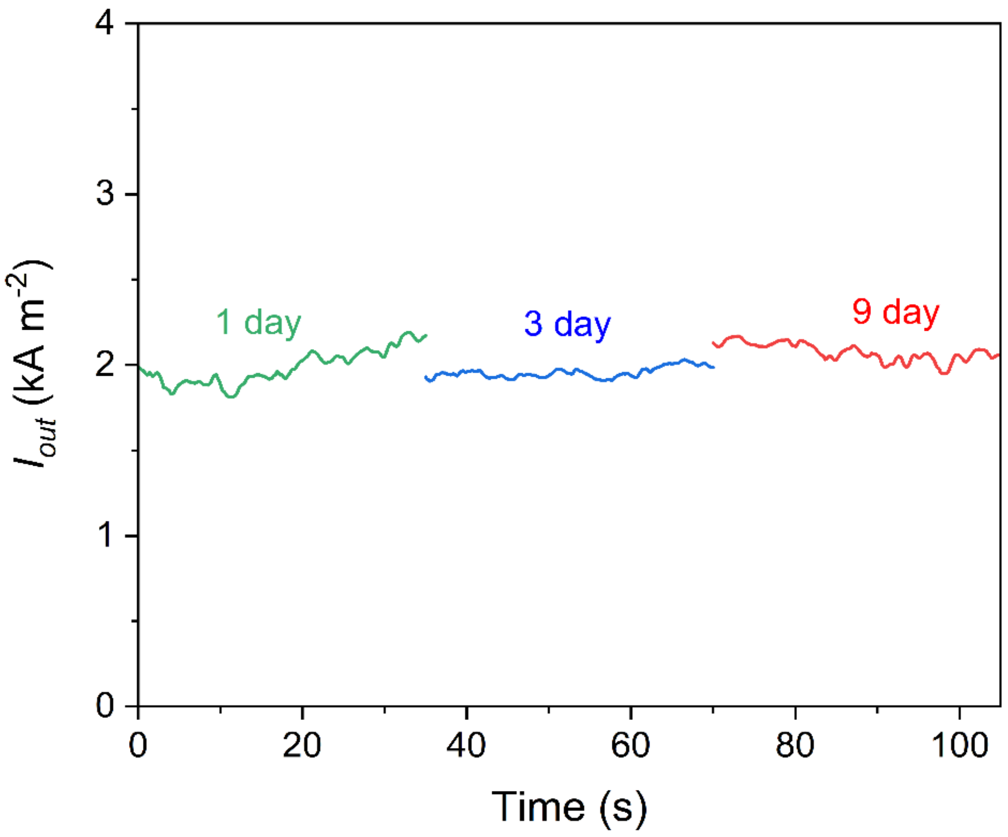

Supplementary Fig. 55| Long-term operational stability of the sV2DP-based energy harvesting device. Power output of the device measured over time under an external load resistance of 2.2 kΩ. The device was tested every three days, showing a nearly constant power output with minimal degradation, thereby demonstrating excellent long-term operational stability of the sV2DP membrane-based system.

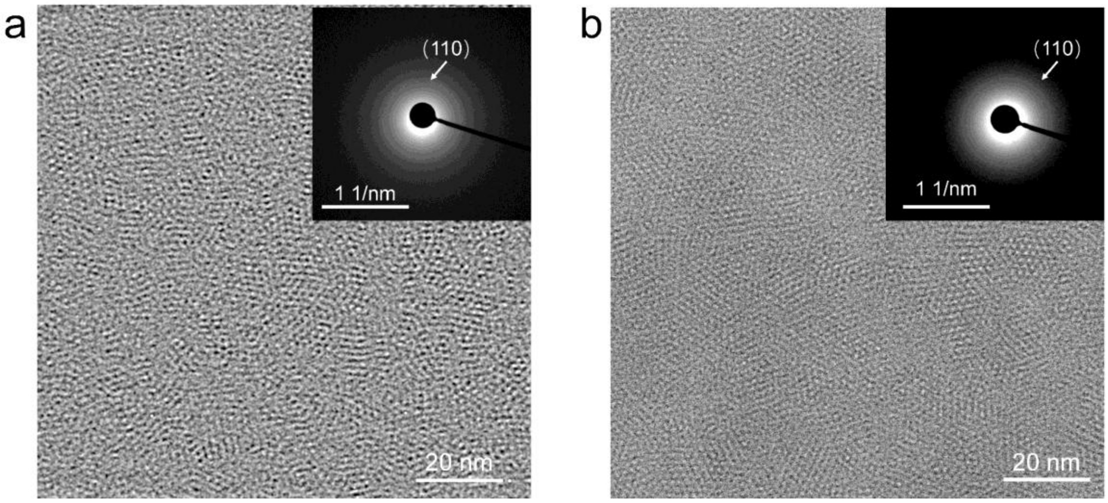

Supplementary Fig. 56| Structural stability of sV2DP membranes. HRTEM images and corresponding SAED patterns (inset) of (a) pre-measurement and (b) post-measurement sV2DP membranes.

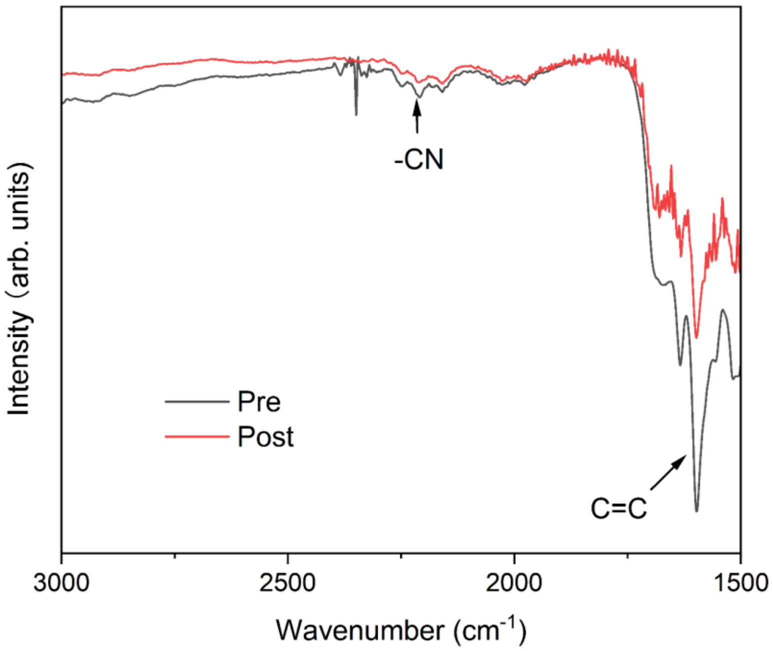

Supplementary Fig. 57| Chemical stability of sV2DP membranes. FTIR spectra of pre-measurement (black) and post-measurement (red) sV2DP membranes.

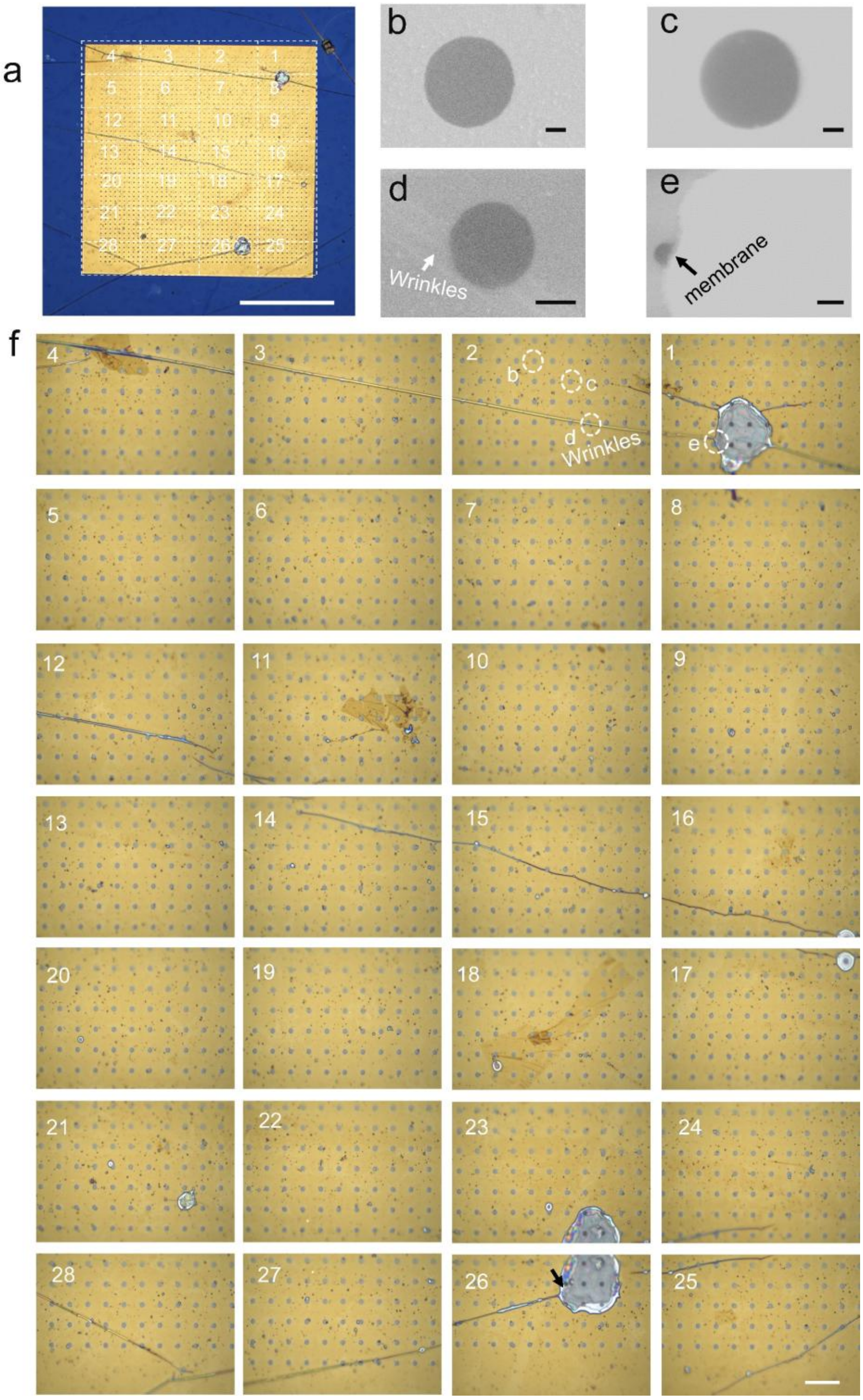

Supplementary Fig. 58| Optical microscopy and SEM images of the large-area sV2DP device after operation. **a**, Optical image of the entire device. **b-e**, SEM images of a representative sV2DP membrane on the array substrate. **f**, Enlarged optical images showing the sV2DP membrane coverage in the marked regions in (a). Note that the macroscopic wrinkles observed in (d) do not indicate damage to the membrane over the aperture. The scale bars are 500 nm in (b,c); 1 µm in (d); 2 µm in (e); and 20 µm in (f).

Supplementary Note 1| Physical grounding and key assumptions for η_{EDL}

In conventional nanofluidics, EDL overlap is typically assessed by comparing the Debye length (λ_D) with a characteristic channel size. While providing a rough estimate of when (or how) electrostatic effects dominate ion transport[27,28], such a simplified descriptor becomes insufficient for two-dimensional framework membranes (2DFMs), including two-dimensional polymers (2DPs), two-dimensional covalent organic frameworks (2D COFs), and two-dimensional metal-organic frameworks (2D MOFs). In these systems, both the channel geometry (e.g., tetragonal vs. hexagonal pores) and the spatial distribution of charges (framework-localized vs. spatially distributed) play a key role in governing ion transport[18], yet are not captured by a single length-scale comparison.

To address this limitation, we introduced η_{EDL} as a dimensionless, structure-aware scaling descriptor that combines electrostatic screening ranges with geometric factors. Its purpose is to provide a physically intuitive measure of the fraction of the channel cross-section under strong EDL influence, thereby directly linking structure to ion-transport behavior. To make this concept more concrete, we present its derivation, physical meaning, and the underlying physical assumptions below.

1. Derivation and physical meaning

In our model, discrete charge sites on the channel wall are treated as point charges that induce an electrostatic perturbation extending over a characteristic distance on the order of λ_D (Supplementary Fig. 2). To estimate the spatial extent of this influence, the EDL-influenced region associated with a single charge site, projected onto the channel cross-section, is approximated as a half-disk of radius λ_D. The factor 0.5 reflects the skeleton charges; for spatially distributed charges within the channel volume, this factor becomes 1. The corresponding area (A) is defined as:

$$A = \frac{\pi \lambda_D^2}{2} \qquad (10)$$

Let ρ denote the number density of charge sites per projected unit area; the total EDL-influenced area per projected unit is approximately ρA. Normalizing this by the effective cross-section area ($f(D)$), yields:

$$\eta_{EDL} = \frac{\rho \pi \lambda_D^2}{2 f(D)} \qquad (11)$$

From a physical perspective, η_{EDL} represents the fraction of the transport cross-section in which electrostatic interactions strongly modulate ion distributions. When $\eta_{EDL} \ll 1$, EDL regions are sparse and ion transport is weakly perturbed. In contrast, when η_{EDL} approaches or

exceeds 1, significant overlap of EDL regions occurs, leading to collective electrostatic confinement, enhanced ion selectivity, and deviation from bulk-like transport behavior. In this way, η_{EDL} provides a direct, physically transparent bridge between the microscopic charge distribution (λ_D and ρ), the channel geometry ($f(D)$), and the macroscopic ion-transport properties. Note that the η_{EDL} is not a universal predictor across arbitrary salt types or pH conditions, where ρ may vary due to specific adsorption or acid-base equilibria. Instead, it serves as a practical scaling metric to rationalize trends and guide optimization within the fixed-charge regime.

2. Key assumptions

We emphasize that η_{EDL} is intended as a heuristic scaling parameter for guiding material and structure design, rather than a replacement for rigorous Poisson-Nernst-Planck descriptions or molecular dynamics (MD) simulations. The formulation is based on the following assumptions:

1) Applicable channel-size regime. The model applies to nanochannels with characteristic dimensions typically larger than ~ 1 nm, where continuum electrostatics and Debye-length-based electrostatic intuition remain valid.[29,30]

2) Mean-field electrostatic screening. λ_D is directly taken from the bulk electrolyte condition based on standard Debye screening, assuming a quasi-equilibrium EDL formation and neglecting strong ion-ion correlations or confinement-induced dielectric.[31]

3) Discrete charge approximation. Surface charges are treated as discrete point charges, and their EDL-influenced region is approximated using a characteristic projected area defined by λ_D.[17]

4) Geometric projection simplification. The EDL influence is approximated through an effective 2D cross-section projection, without considering spatial variations, for simplified structural comparison.

Supplementary Note 2| Analysis for generated I_{sc} and V_{oc} from the osmotic gradients The I_{sc}, arises from diffusion-osmotic flow, which drags counterions within EDLs, thereby generating an electric current due to excess ionic movement. This current can be expressed as:[32]

$$I_{sc}=F f(D) \sum_i z_i J_i \qquad (12)$$

In the case of a positively charged channel containing only monovalent ions, where counterions are anions (e.g., Cl^-) and co-ions are cations (e.g., K^+), the expression simplifies to:

$$I_{sc}=F f(D)(J_- - J_+) \qquad (13)$$

where F is the Faraday constant, $f(D)$ represents the effective cross-sectional area of the channel, and J_- and J_+ are the fluxes of counterions and co-ions, respectively. This formulation highlights that the s2DP channel generates a higher I_{sc}, owing to its increased Cl^- flux and reduced K^+ flux, stemming from its increased EDLs overlap efficiency (i.e., η_{EDL}) compared to the e2DP configuration.

As for V_{oc}, it originates from charge separation driven by differential ion mobilities and selective ion transport. It can be expressed as:[20]

$$V_{oc}=\frac{(2t_- -1)RT \ln\frac{c_{high}}{c_{low}}}{F} \qquad (14)$$

, where t_- is the anion transference number, representing the fraction of total ionic flux carried by the anion (Cl^-), given by:

$$t_-=\frac{J_-}{J_- + J_+} \qquad (15)$$

Thus,

$$V_{oc} \sim t_- \qquad (16)$$

Given the substantially elevated Cl^- flux and suppressed K^+ flux in s2DP, t_- approaches unity, leading to a significantly improved V_{oc} compared to e2DP.

As a result, the theoretical maximum power output (P_{max}) of s2DP is also higher than that of e2DP, following the relationship:

$$P_{max}=\frac{V_{oc} \times I_{SC}}{4f(D)} \qquad (17)$$

Supplementary Note 3| Surface-charge-density analysis for ultranarrow sV2DP nanochannels

The surface charge density of sV2DP is derived by a constant charge model rather than an independent physical measurement. To quantify the effective (or apparent) surface charge density (Σ) of sV2DP nanochannels, we analyze the ionic conductance measured under symmetric electrolyte conditions (identical KCl concentrations on both sides of the membrane). The total conductance(G) is modeled as the sum of a bulk conductance (G_{bulk}) and a surface conductance ($G_{surface}$) for ultranarrow triangular nanochannels in sV2DP:

$$G=G_{bulk}+G_{surface}=N_{ch}\left(\frac{q(\mu^{+}+\mu^{-})C_B N_A wh}{2l}+\frac{\mu^{-}\Sigma w}{l}\right) \quad (18)$$

where q is the elementary charge (1.60217663 × 10^{-19} C); μ^{+} represents the mobility of K^{+} (7.6 ×10^{-8} m^2 s^{-1} v^{-1}); μ^{-} refers to the mobility and Cl^{-} (7.9 × 10^{-8} m^2 s^{-1} v^{-1}); C_B is the bulk electrolyte concentration; N_A is Avogadro's number (6.023 × 10^{23}); *h and w* are the side length and height for the triangular nanochannel cross-section (2.36 and 2.04 nm, respectively); and l is the thickness of membranes (50 nm).

In concentrated electrolyte (3 M KCl), EDLs are strongly screened, and the conductance is dominated by G_{bulk}. At lower concentrations (10^{-4} to 1 M), the surface contribution becomes significant, leading to deviations from linear (bulk-dominated) scaling. Thus, the conductance at 3 M KCl is used to determine the effective number of conducting (charged) channels (N_{ch}), assuming G = G_{bulk} under a strong screen. With the channel number fixed, experimental conductance values across KCl concentrations are then fitted using the above equation to extract the Σ value for sV2DP nanochannels (Fig. 2a). As a result, surface charge densities of ~ 22.4 and 10.6 mC m^{-2} were estimated for sV2DP in 0.25 M KCl and 0.1M KCl, respectively, which are stronger than reported charged 2DFMs (<10 mC m^{-2}).[19,27,30,33]

Supplementary Table 1| Summary of characteristic *D* in reported 2DFMs for ion-selective transport

2DFMs	D_{eff} (nm)	*Geometry*	$f(D)(nm^2)$	n^a	ρ (nm^{-2})^b
2DPI[20,27]	2.55	rectangle	6.5	4	0.62
C2DP[34]	3.05	rectangle	9.3	8	0.86
V2DP[35]	4.5	hexagon	50.29	12	0.24
COF-DT[36]	3.2	hexagon	8.04	6	0.74
EB-2DP[19]	2.6	hexagon	5.31	6	1.13
PI-2DP	2.6	hexagon	5.31	9	1.69
Ca-COF[30]	1.4	hexagon	1.53	3	1.96
COF-170[37]	2.3	hexagon	5.73	6	1.04
**sV2DP** in this work	**1.36**	**triangle**	**1.46**	**6**	**4.11**

Note: All the data were directly extracted or adopted from the cited references. ^{a} *n* denotes the number of charge sites per framework unit. ^{b} ρ is calculated as the ratio of *n* and *f(D)*.

Supplementary Table 2| Sizes of halide ions investigated in this study.

Ions	Atomic diameter (nm)	Hydrated diameter (nm)
Cl^-	0.360 ± 0.014	0.62
Br^-	0.396 ± 0.010	0.66
I^-	0.450 ± 0.008	0.7

Note: the atomic diameters were extracted from ref[9], and the hydrated diameter was calculated from the g(r) between ions and water molecules

Supplementary Table 3| Comparison of state-of-the-art ion-selective membranes in OPG applications (under a 50-fold NaCl gradient)

Materials			*t*	***EIR*** **(x 10^{-2} Ω cm^2)**	$\boldsymbol{P_{out}}$ **(W m^{-2})**
**2DFMs**	This work	sV2DP	0.90	2.36	241.5
	2DP films	ZnTPP–COF[27]	0.64	1.57	135.8
		V2DP[35]	~ 0.7	7.2	48.5
		PI-2DP[19]	0.82	12	48.4
		2DPI[20]	0.81	24	53.1
		Ca-COF[30]	0.78	30	180.5
		N^+-PAPD[38]	0.73	22.5	42.6
	2D COFs membranes	COF-$(SO_3Na)_1$/PAN[39]	0.9275	64.37	29
		p-BCP-1[40]	0.85	80	19.3
		TpEB@TpPa-SO_3Na[41]	0.87	165	19.2
		TFPT-TMT COF[42]	~ 0.7	133.45	13.3
		TpPa-SO_3H COF[43]	> 1[a]	690	5.9
		PyPa-SO_3H COF/SANF[44]	0.86	150	8.7
		U-IDM [45]	0.85	1500	3.56
		PIDM[46]	0.83	300	9.3
	2D MOF membranes	Cu-TCPP[47]	0.87	270	16.64
**Other 2D nanofluidic membranes**	laminar membranes	2D-NNF[29]	0.7986	180	8.61
		GO/IL[48]	> 1[a]	390	6.7
		bsGOM[49]	0.888	1080	5.5
		MoS_2/CNF[50]	> 1[a]	690	5.2
		LDH/AAO[51]	0.7837	690	2.85
		Heterogeneous MXene[52]	~ 0.943	300	8.6
		H-MXene[53]	0.972	270.6	9.47
	Nanoporous membranes	Nanoporous $Ti_3C_2T_x$[54]	~ 0.9	300	12
		GPPS membrane[55]	0.84	180	8.65

Note: All the data were directly extracted or adopted from the cited references.[a] The *t* values in this table were derived from the V_{os} values; > 1 indicates full selectivity for the membranes.

Supplementary Table 4| Hydration-shell metrics extracted from *g(r)*

Ion	First peak (Å)	Coordination number	Reported experimental range	Ref.
Cl^-	3.1	7.0	4.0-8.9	[56,57]
Br^-	3.3	6.9	6.0-7.4	[58]
I^-	3.5	7.6	4.2-10.3	[56,57]

Supplementary Table 5| Summary of solution compositions and their pH values at 25 ºC

Electrolytes	Concentration (M)	pH
KCl	0.01 M	6.10
KCl	0.5M	6.42
NaCl	0.01M	6.55
NaCl	0.5M	6.64
Artificial river water	0.01 M NaCl	6.55
Artificial seawater	NaCl (0.48)+$CaCl_2$ (0.01)+$MgCl_2$ (0.03)+$MgSO_4$ (0.03)	7.20

Supplementary Table 6| Comparison of reported OPG performance under sea-water/river water salinity gradients

Materials	Device area (mm^2)	P_{out} ($W\ m^{-2}$)
2D-NNF[29]	3×10^{-2}	8.61
TNC-MCM[59]	3×10^{-2}	7.7
MXene/Kevlar nanofiber composite[60]	3×10^{-2}	4.10
HB67.7@COF JG80.1@COF[61]	7.85×10^{-3}	36.5
Mushroom-shaped nanochannel array membrane[62]	8×10^{-3}	14.60
ZnTPP-COF[27]	6.36×10^{-3}	14.63
Ca-COF[30]	6.36×10^{-3}	24.7
sV2DP in this work	single/micro-aperture (1.256×10^{-5})	218
	aperture-array (6.36×10^{-3})	46.7

Note: All the data were directly extracted or adopted from the cited references.

Supplementary References1. Kuhne, T. D. et al. CP2K: An electronic structure and molecular dynamics software package - Quickstep: Efficient and accurate electronic structure calculations. *J. Chem. Phys.* **152,** 194103 (2020).

2. VandeVondele, J. & J. Hutter Gaussian basis sets for accurate calculations on molecular systems in gas and condensed phases. *J. Chem. Phys.* **127,** 114105 (2007).

3. S. Goedecker, M. Teter & J. Hutter Separable dual-space Gaussian pseudopotentials. *Phys. Rev. B* **3,** 1703 (1996).

4. Krack, M. Pseudopotentials for H to Kr optimized for gradient-corrected exchange-correlation functionals. *Theor. Chem. Acc.* **114,** 145-152 (2005).

5. Van Der Spoel, D. et al. GROMACS: fast, flexible, and free. *J. Comput. Chem.* **26,** 1701-1718 (2005).

6. Bussi, G., D. Donadio & M. Parrinello Canonical sampling through velocity rescaling. *J. Chem. Phys.* **126,** 014101 (2007).

7. Essmann, U. et al. A smooth particle mesh Ewald method. *J. Chem. Phys.* **103,** 8577-8593 (1995).

8. Darden, T., D. York & L. Pedersen Particle mesh ewald: an n·log(n) method for ewald sums in large systems. *J. Chem. Phys.* **98,** 10089-10092 (1993).

9. Joseph O. Hirschfelder, Charles F. Curtiss & R. B. Bird (1964). Molecular theory of gases and liquids.

10. Michael P. Allen & D. J. Tildesley (1992). Computer simulation of liquids

11. Daan Frenkel & B. Smit (2022). Understanding molecular simulation: from algorithms to applications

12. Berendsen, H. J. C., J. R. Grigera & T. P. Straatsma The missing term in effective pair potentials. *J. Phys. Chem.* **91,** 6269-6271 (2002).

13. A. D. MacKerell, J. et al. All-atom empirical potential for molecular modeling and dynamics studies of proteins. *J. Phys. Chem. B* **102,** 3586–3616 (1998).

14. Miyamoto, S. & P. A. Kollman Settle: An analytical version of the SHAKE and RATTLE algorithm for rigid water models. *J. Comput. Chem.* **13,** 952-962 (2004).

15. Hess, B. et al. LINCS: A linear constraint solver for molecular simulations. *J. Comput. Chem.* **18,** 1463-1472 (1997).

16. Hess, B. P-LINCS: A parallel linear constraint solver for molecular simulation. *J. Chem. Theory Comput.* **4,** 116-122 (2008).

17. Schott, C. M. et al. How to assess and predict electrical double layer properties. implications for electrocatalysis. *Chem. Rev.* **124,** 12391-12462 (2024).

18. Emmerich, T. et al. Nanofluidics. *Nat. Rev. Meth. Prim.* **4,** 69 (2024).
19. Liu, X. et al. Giant blue energy harvesting in two-dimensional polymer membranes with spatially aligned charges. *Adv. Mater.* **36,** 2310791 (2024).
20. Zhang, Z. et al. Cation-selective two-dimensional polyimine membranes for high-performance osmotic energy conversion. *Nat. Commun.* **13,** 3935 (2022).
21. Yang, Y. et al. On-water surface synthesis of vinylene-linked cationic two-dimensional polymer films as the anion-selective electrode coating. *Angew. Chem. Int. Ed.* **63,** e202316299 (2024).
22. Yin, H. Q., F. Yin & X. B. Yin Strong dual emission in covalent organic frameworks induced by ESIPT. *Chem. Sci.* **10,** 11103-11109 (2019).
23. Ghosh, S. et al. Identification of prime factors to maximize the photocatalytic hydrogen evolution of covalent organic frameworks. *J. Am. Chem. Soc.* **142,** 9752-9762 (2020).
24. Wu, X. et al. Control interlayer stacking and chemical stability of two-dimensional covalent organic frameworks via steric tuning. *J. Am. Chem. Soc.* **140,** 16124-16133 (2018).
25. Zou, Y. et al. Cooperative steric modulation of flexibility, disorder, and pore size in two-dimensional covalent organic framework membranes for enhanced selective ion sieving. *J. Am. Chem. Soc.* **147,** 32580-32590 (2025).
26. I. S. Joung, T. E. C. Determination of alkali and halide monovalent ion parameters for use in explicitly solvated biomolecular simulations. *J. Phys. Chem. B* **112,** 9020-9041 (2008).
27. Yang, J. et al. Advancing osmotic power generation by covalent organic framework monolayer. *Nat. Nanotechnol.* **17,** 622-628 (2022).
28. Zhang, Z., L. Wen & L. Jiang Nanofluidics for osmotic energy conversion. *Nat. Rev. Mater.* **6,** 622-639 (2021).
29. Tang, J. et al. All-natural 2D nanofluidics as highly-efficient osmotic energy generators. *Nat. Commun.* **15,** 3649 (2024).
30. Jiang, W. et al. Axial alignment of covalent organic framework membranes for giant osmotic energy harvesting. *Nat. Sustain.* **8,** 446-455 (2025).
31. Li, X. et al. Constructing new-generation ion exchange membranes under confinement regime. *Natl. Sci. Rev.* **12,** nwae439 (2025).
32. Garaj, S. et al. Graphene as a subnanometre trans-electrode membrane. *Nature* **467,** 190-193 (2010).
33. Cheng, B. et al. Giant Gateable Osmotic Power Generation from a Goldilocks Two-Dimensional Polymer. *J Am Chem Soc* **145,** 5261-5269 (2023).

34. Wang, Z. et al. On-water surface synthesis of charged two-dimensional polymer pingle crystals via the irreversible katritzky reaction. *Nat. Synth.* **1,** 69-76 (2021).
35. Liu, X. et al. Highly anion-conductive viologen-based two-dimensional polymer membranes as nanopower generators. *Angew. Chem. Int. Ed.* **63,** e202409349 (2024).
36. Cao, L. et al. Oriented two-dimensional covalent organic framework membranes with high ion flux and smart gating nanofluidic transport. *Angew. Chem. Int. Ed.* **61,** e202113141 (2022).
37. Meng, Q. et al. Optimizing selectivity via membrane molecular packing manipulation for simultaneous cation and anion screening. *Sci. Adv.* **10,** eado8658 (2024).
38. Zheng, S. et al. Quantitative skeletal-charge engineering of anion-selective cof membrane for ultrahigh osmotic power output. *J. Am. Chem. Soc.* **147,** 15777-15786 (2025).
39. Zuo, X. et al. Thermo-osmotic energy conversion enabled by covalent-organic-framework membranes with record output power density. *Angew. Chem. Int. Ed.* **61,** e202116910 (2022).
40. Li, C. et al. One porphyrin per chain self-assembled helical ion-exchange channels for ultrahigh osmotic energy conversion. *J. Am. Chem. Soc.* **144,** 9472-9478 (2022).
41. Cao, L. et al. An ionic diode covalent organic framework membrane for efficient osmotic energy conversion. *ACS Nano* **16,** 18910-18920 (2022).
42. Wang, K. et al. Monolayer-assisted surface-Initiated Schiff-base-mediated aldol polycondensation for the synthesis of crystalline sp^2 carbon-conjugated covalent organic framework thin films. *J. Am. Chem. Soc.* **145,** 5203-5210 (2023).
43. Hou, S. et al. Free-standing covalent organic framework membrane for high-efficiency salinity gradient energy conversion. *Angew. Chem. Int. Ed.* **60,** 9925-9930 (2021).
44. Man, Z. et al. Serosa-mimetic nanoarchitecture membranes for highly efficient osmotic energy generation. *J. Am. Chem. Soc.* **143,** 16206-16216 (2021).
45. Yang, Y. et al. Unipolar ionic diode nanofluidic membranes enabled by stepped mesochannels for enhanced salinity gradient energy harvesting. *J. Am. Chem. Soc.* **146,** 19580-19589 (2024).
46. Chang, J. N. et al. Plasmonic ion diode membrane (PIDM) for enhanced nanofluidic ion transport and osmotic energy harvesting. *Angew. Chem. Int. Ed.* **64,** e202502591 (2025).
47. Wang, J. et al. Light-responsive and ultrapermeable two-dimensional metal-organic framework membrane for efficient ionic energy harvesting. *Nat. Commun.* **15,** 2125 (2024).
48. Hu, Y. et al. Confined ionic liquid-mediated cation diffusion through layered membranes for high-performance osmotic energy conversion. *Adv. Mater.* **35,** e2301285 (2023).
49. Qian, Y. et al. Boosting osmotic energy conversion of graphene oxide membranes via self-exfoliation behavior in nano-confinement spaces. *J. Am. Chem. Soc.* **144,** 13764-13772 (2022).

50. Zhu, C. et al. Metallic two-dimensional MoS_2 composites as high-performance osmotic energy conversion membranes. *J. Am. Chem. Soc.* **143,** 1932-1940 (2021).
51. Liu, Y., J. Ping & Y. Ying Anion-selective layered double hydroxide composites-based osmotic energy conversion for real-time nutrient solution detection. *Adv. Sci.* **9,** e2103696 (2022).
52. Ding, L. et al. Bioinspired $Ti_3C_2T_x$ MXene-based ionic diode membrane for high-efficient osmotic energy conversion. *Angew. Chem. Int. Ed.* **61,** e202206152 (2022).
53. Qian, H. et al. Horizontal transport in $Ti_3C_2T_x$ mxene for highly efficient osmotic energy conversion from saline-alkali environments. *Angew. Chem. Int. Ed.* **63,** e202414984 (2024).
54. Hong, S. et al. Porous Ti_3C_2Tx mxene membranes for highly efficient salinity gradient energy harvesting. *ACS Nano* **16,** 792-800 (2022).
55. Zhi, H. et al. Large-area graphene-based ion-selective membranes with micro/meso-pores for osmotic energy harvesting. *Adv. Funct. Mater.* **34,** 2401922 (2024).
56. Marcus, Y. Effect of ions on the structure of water: structure making and breaking. *Chem. Rev.* **109,** 1346-1370 (2009).
57. Migliorati, V., P. D'Angelo & F. Sessa Going beyond radial hydration models: the hidden structures of chloride and iodide aqua ions revealed by the use of lone pairs. *J. Phys. Chem. B.* **127,** 10843-10850 (2023).
58. D'Angelo, P., V. Migliorati & L. Guidoni Hydration properties of the bromide aqua ion: the interplay of first principle and classical molecular dynamics, and X-ray absorption spectroscopy. *Inorg. Chem.* **49,** 4224-4231 (2010).
59. Zou, K. et al. Turing-type nanochannel membranes with extrinsic ion transport pathways for high-efficiency osmotic energy harvesting. *Nat. Commun.* **15,** 10231 (2024).
60. Zhang, Z. et al. Mechanically strong MXene/Kevlar nanofiber composite membranes as high-performance nanofluidic osmotic power generators. *Nat. Commun.* **10,** 2920 (2019).
61. Chen, S. et al. Imparting ion selectivity to covalent organic framework membranes using de novo assembly for blue energy harvesting. *J. Am. Chem. Soc.* **143,** 9415-9422 (2021).
62. Chao Li et al. Large-scale, robust mushroom-shaped nanochannel array membrane for ultrahigh osmotic energy conversion. *Sci. Adv.* **7,** eabg2183 (2021).
