# Supplementary material for: Ultranarrow nanochannels in a staggered two-dimensional polymer membrane enhance electric double-layer coverage for osmotic energy harvesting
Source: Nat Commun. 2026 Jun 19;17:5460. doi: 10.1038/s41467-026-74696-4 (PMC13282372; doi:10.1038/s41467-026-74696-4)
Supplement: Supplementary file 2 — Transparent Peer Review file [file 41467_2026_74696_MOESM2_ESM.pdf]

nature portfolio

Peer Review File

Ultranarrow nanochannels in a staggered two-dimensional polymer membrane enhance electric double-layer coverage for osmotic energy harvesting

Corresponding Author: Professor Xinliang Feng

This file contains all reviewer reports in order by version, followed by all author rebuttals in order by version.

Version 0:

Reviewer comments:

Reviewer #1

(Remarks to the Author)
This work reports a two-dimensional polymer membrane featuring stacked, triangular one-dimensional nanochannels for osmotic energy harvesting. The authors attribute the enhanced osmotic energy conversion performance to the combination of a high surface charge density and the stacked nanochannel architecture, which effectively reduces the effective pore size and thereby amplifies surface-charge-governed ion selectivity. The concept is technically sound and the introduction of an unique “screw-like” anion migration pathway—an aspect that has not been systematically explored in previous studies—offers a certain degree of novelty and conceptual interest. However, the membrane’s power output is not particularly competitive when benchmarked against leading single-layer polymer systems. In addition, several of the design elements emphasized in the manuscript—most notably the influence of surface charge and the benefits of aligned nanochannel architectures—have been previously recognized in the literature. That said, the introduction of a screw-like anion migration pathway represents an interesting and less-explored structural feature, and this aspect could offer fresh insight and stimulate further progress in the field. The manuscript is clearly written and well structured. The material synthesis and characterization are competently executed. The performance evaluation and mechanistic interpretation require further clarification and revision. Several issues remain unresolved and should be addressed before the work can be considered for publication.
1. On page 2, the author introduces a simplified conceptual expression to quantify the efficiency of EDL coverage. However, the physical meaning of this formula and the rationale behind its specific mathematical form are not clearly articulated. A more detailed explanation is needed to clarify how the expression is derived, what assumptions are embedded in it, and how it relates to the underlying ion-transport physics within the nanochannels.
2. On page 2, please clarify how the function f(D) is defined and calculated in the author’s analysis. In Supplementary Table 1, the values of f(D) reported for other literatures are consistently much larger than the corresponding pore diameter D, whereas for the membrane developed in this work, f(D) appears smaller than D. The author should explain the physical basis and methodological rationale for this discrepancy.
3. Why the author chose to rely on a stacked architecture to reduce the effective pore size instead of directly fabricating membranes with intrinsically smaller nanochannel diameters. What specific advantages does the stacked configuration provide over simply designing smaller straight-through pores? A justification is needed, given that channels with genuinely small diameters would, in principle, offer more straightforward control over selectivity and may support faster ion transport.
4. In Supplementary Fig. 12, the author claims that the membrane exhibits pronounced ion rectification. However, the reported rectification ratio is only 1.12, which is extremely small and, in my view, insufficient to substantiate the presence of a meaningful rectification effect. In addition, it is unclear how ion rectification could arise in the present system given that the fabricated membrane is structurally symmetric. The supporting substrate contains large (~2 μm) pores whose surface charge is unlikely to impose any appreciable regulation on ion transport, and the overall membrane architecture appears symmetric with respect to both pore size and surface charge along the transport direction. The authors should explain the origin of the observed asymmetric current response under these conditions.
5. In Supplementary Fig. 13, the manuscript presents ion conductance curves, but it is not clear how the conductance values were calculated. Was the author accounting for the purported ion rectification in this analysis? If rectification is indeed present, the conductance in the forward and reverse directions should differ. How, then, were the conductance values extracted or averaged? A detailed explanation of the calculation procedure is required.
6. In Fig. 2b, the trend in the measured Zeta potentials at different electrolyte concentrations is not clearly explained.

Theoretically, higher ionic strength should lead to stronger screening of surface charges, and one would therefore expect a decreasing trend. The authors should clarify the physical mechanism underlying the observed behaviors.
7. Please provide the redox potential data corresponding to Supplementary Fig. 18. It is unclear why the blank substrate does not exhibit any measurable redox potential under a tenfold concentration gradient. Furthermore, the I–V curves shown in this figure cover only a very narrow voltage range. Please supply the full I–V curves and explain the origin of the deviation from linearity observed in their shape.
8. For the MD simulations presented in Fig.3, it is not clear whether the calculations account for the relationship between the hydrated ion size and the pore size. The pore diameter of the eV2DP membrane appears to be comparable to the size of the hydrated ions themselves. Under such pore–ion size matching conditions, the reduction of the effective pore size arising from the stacked configuration may not yield a substantial enhancement in ion-selective transport. The author should clarify how hydration effects were treated in the simulations and discuss the implications for the claimed performance improvement.

Reviewer #2

(Remarks to the Author)
This manuscript reports an ultrathin (~50 nm), fully crystalline, ABC-stacked viologen-incorporated 2D polymer membrane (sV2DP) featuring vertically aligned triangular nanochannels (reported $D \approx 1.2$ nm) with densely charged pyridinium sites (reported ~67.1 mC m^{-2}). The authors argue that the staggered (ABC) stacking effectively narrows the transport pathway, increases electric double-layer (EDL) overlap/“coverage efficiency,” and thereby boosts anion selectivity and permeability. The membrane is integrated into osmotic power generators and claimed to deliver very high outputs (e.g., 243 W m^{-2} under a 50-fold NaCl gradient) and a macroscopic aperture-array device reaching ~46.7 W m^{-2} under artificial seawater/river-water conditions with multi-day stability. The concept is interesting and potentially impactful, but several central quantitative claims (surface charge density, selectivity metrics, and area-normalized power density) rely on assumptions that are not convincingly justified for ~1 nm channels with strongly overlapping EDLs and possible surface conduction. Substantial clarification and additional validation are needed.
Major comments
1. Surface charge density derived from electrokinetic analysis appears unreliable for ~1 nm channels
The manuscript reports a high surface charge density and uses it as a key mechanistic pillar. However, the pathway “streaming current/streaming potential → zeta potential → surface charge density” is typically derived under assumptions that are questionable in ultra-confined channels with strong EDL overlap, ion-specific effects, and potentially significant surface conduction.
In this regime, the extracted zeta potential may not represent a physically meaningful interfacial potential in the usual sense, and converting it to surface charge density via classical relations can produce large systematic errors.
What I need to see: a regime-appropriate electrokinetic model (explicitly including/estimating surface conductance effects and overlap) and at least one independent cross-validation of charge density (e.g., counterion uptake/charge titration, stoichiometry + accessible site density constraints, or concentration-dependent conductance fitted with a physically grounded model rather than a single-point zeta conversion).
2. “EDL coverage efficiency” (η_{EDL}) requires stronger physical grounding and validation
The proposed η_{EDL} metric is presented as a unifying descriptor that explains performance gains. At present it reads as a heuristic quantity tuned to this specific geometry, with limited evidence that it is predictive rather than descriptive.
In highly confined ~1 nm channels, Debye-length-based intuition can break down (finite-ion size, correlations, dielectric effects), so any scalar “coverage efficiency” must be carefully justified.
What I need to see: sensitivity analysis showing how η_{EDL} changes with the assumed effective diameter/cross-section and how conclusions hold if screening deviates from ideal Debye behavior. Ideally, demonstrate predictive capability across multiple salts, ionic strengths, and pH values (not just one best condition).
3. Power density and current density normalization: effective area/leakage/edge pathways must be ruled out
The reported selective current density (e.g., 13.2 kA m^{-2} under a 50-fold KCl gradient) and power density (e.g., 243 W m^{-2} under 50-fold NaCl) are exceptionally high for a membrane system, making rigorous validation essential.
For ultrathin membranes on apertures, small defects, imperfect sealing, or edge leakage can dominate current while preserving “reasonable-looking” I–V curves. A bare-substrate control is not sufficient.
What I need to see: a) Statistics across many devices (n, yield, distribution) for Voc, Isc, Pmax, and internal resistance. b) Post-measurement imaging of the exact tested membranes (or a robust set) to exclude pinholes/tears and to verify uniform coverage. c) Additional leakage controls (intentional defect control, thickness series, or tracer permeability test) to show that transport is genuinely through the designed nanochannels.
4. Selectivity and transference number extraction may be confounded under strong gradients
Selectivity inferred from reversal potentials can be distorted by non-ideal solution behavior at high concentration, junction potentials, and concentration polarization near the membrane. These effects can bias transference numbers and selectivity estimates upward.
What I need to see: a) Evidence that concentration polarization is controlled/quantified (e.g., stirring rate dependence, flow-through tests, or boundary layer analysis). b) Ideally, direct ionic flux measurements (ion chromatography/ICP) under operating conditions to validate transference numbers independent of voltage-only inference. c) Discussion or correction strategy for non-ideality when using large concentration ratios.
5. Scale-up gap: micro-aperture vs macroscopic array performance needs quantitative diagnosis
The drop from micro-aperture performance to the macroscopic aperture-array level is not surprising, but the manuscript’s explanation (defects/grain boundaries) remains qualitative.
What I need to see: quantitative mapping of where resistance arises in the array device (membrane intrinsic vs aperture edge vs sealing vs support), and characterization of defect density/continuity over large areas. A scaling plot of performance vs active area with an error model would help.
6. Simulation–experiment linkage is currently too qualitative

The “screw-like” anion pathway and spiral charge arrangement are visually intriguing, but the manuscript does not yet demonstrate that this mechanism quantitatively explains the measured selectivity/power enhancements.
What I need to see: simulation outputs that connect directly to measurable quantities (conductance vs concentration, transference number, ion distributions comparable to extracted parameters) and robustness checks (multiple trajectories/initializations, salt types, concentrations, and sensitivity to chosen force field/charges).
7. Mechanical integrity and long-term operation
The manuscript claims multi-day stability in output. For ultrathin (~50 nm) freestanding/partly supported films, mechanical integrity and chemical stability under gradients are critical.
What I need to see: clearer evidence that stability reflects membrane robustness rather than stabilization of external factors (electrodes, reservoir chemistry, polarization). Include pre-/post-operation structural and chemical characterization, and specify whether any performance drift correlates with resistance changes.
Minor comments
Please add uncertainty/error bars and replicate numbers for all key extracted metrics (zeta potential, inferred charge density, selectivity/transference number, permeability/current density, and power density).
Clearly define all symbols (including η_EDL) at first use, and provide a transparent derivation (or assumptions) with dimensional checks.
Provide full measurement conditions for electrokinetic and osmotic tests (electrode type, electrode spacing, reservoir volumes, stirring/flow conditions, temperature control, and measurement duration per point).
Clarify whether “channel size” refers to geometric pore size from structure, an “effective” electrostatic diameter, or an effective hydraulic diameter—and remain consistent throughout.
Improve figure readability: ensure axes include units, specify concentration ratios explicitly (e.g., 50-fold = C_high/C_low), and avoid mixing normalized and absolute quantities without clear labeling.
Correct typographical issues (e.g., spelling of “Debye length” and any inconsistent unit formatting such as m−2, kA m−2).
If possible, include additional controls: pH dependence, multivalent ions, and concentration-dependent conductance to support the claimed mechanism.

Version 1:

Reviewer comments:

Reviewer #1

(Remarks to the Author)
All of our comments have been solved and this work can be published now.

Reviewer #2

(Remarks to the Author)
The revised manuscript is substantially improved, and the authors have made a serious effort to address the reviewers’ concerns. The work is interesting, the membrane synthesis and characterization are generally strong, and the revision has clarified several previously ambiguous points. However, a number of important issues remain only partially resolved, mainly related to the quantitative interpretation of surface charge density, selectivity analysis under strong gradients, the simulation-to-experiment link, and the framing of benchmarking claims. In my view, the manuscript is closer to publishable form, but these remaining points should still be corrected before acceptance.

Comment 1: Surface charge density analysis remains internally inconsistent.
The revised manuscript improves the discussion of charge density by moving away from an overinterpretation of zeta potential and introducing a conductance-based constant-charge model. However, the manuscript still contains an internal inconsistency because the Results section attributes the charge density to a constant-charge conductance fit, while the Methods section continues to describe a Gouy–Chapman/zeta-potential-based estimate. This inconsistency affects a central mechanistic pillar of the paper and must be corrected so that the charge-density analysis is described in one consistent manner throughout.

Comment 2: Charge density is still not independently validated.
Although the revised conductance-based fitting is more appropriate than the earlier treatment, the effective fixed-charge density still rests on a model-dependent estimate without an orthogonal validation method. For such highly confined channels, this is a nontrivial limitation. The authors should either provide an independent cross-check of charge density or clearly soften the language and state that the reported value is an effective fitted parameter rather than a directly validated physical surface charge density.

Comment 3: The η_EDL metric is better framed, but still functions mainly as a heuristic descriptor.
The revision appropriately repositions η_EDL as a heuristic, first-order design metric rather than a rigorous predictive theory. That is a positive change. However, the manuscript still uses η_EDL somewhat aggressively to rationalize performance improvements without demonstrating broader predictive capability across systems or conditions. The authors should keep the interpretation modest and consistently present η_EDL as a qualitative design descriptor rather than a validated universal metric.

Comment 4: The selectivity and transference-number analysis is still not fully closed.

The authors have clarified how selectivity is extracted and have improved the explanation of activity versus concentration ratios, junction potential minimization, and voltage stabilization. However, the reported selectivity and transference values still rely entirely on reversal-potential inference under strong concentration gradients, without an independent validation such as direct ionic-flux measurements or a more explicit boundary-layer analysis. The approach is reasonable, but the claims should be tempered because the analysis remains indirect.

Comment 5: Concentration polarization is acknowledged but not rigorously quantified.
The rebuttal explains why concentration polarization is expected to be limited under their testing geometry and open-circuit protocol, which is helpful. Still, the manuscript does not provide a quantitative assessment of boundary-layer effects, nor does it show stirring-rate or flow-dependent controls. Because strong gradients are central to the reported performance, the discussion should be more explicit that residual concentration polarization could influence the extracted transport parameters and remains a limitation of the current analysis.

Comment 6: The rectification discussion remains somewhat overstated.
The authors now acknowledge that the rectification ratio is modest, which is appropriate. Nevertheless, the text still presents ion rectification as a notable characteristic, even though the measured asymmetry is weak and the mechanistic basis remains somewhat indirect. The authors should further soften this point and avoid implying that the system exhibits strong or architecturally intrinsic rectification.

Comment 7: Leakage control is much improved, but some doubt remains because the key evidence is mostly in the Supplementary Information.
The added controls on intentional defects, thickness dependence, defective membranes, and post-measurement imaging considerably strengthen the manuscript, and these are among the most convincing parts of the revision. That said, because the extraordinary current and power densities are central to the paper, the main text should more clearly emphasize the most important leakage-sensitive controls. The current presentation still leaves too much of the validation burden in the Supplementary Information.

Comment 8: The scale-up discussion is improved but still partly inferential.
The revised manuscript now provides a more credible explanation for the performance drop from micro-aperture devices to the array format, including resistance partitioning and the role of grain boundaries and defects. This is a significant improvement. However, the resistance decomposition is still not directly measured in a fully quantitative way, and the array-device statistics remain limited. The explanation is plausible, but the authors should avoid presenting it as fully established.

Comment 9: The simulation–experiment connection is still qualitative.
The MD results are visually compelling and provide a plausible mechanistic picture for the proposed screw-like pathway. However, they still do not quantitatively connect to the measured conductance, selectivity, or power output. As a result, the simulations mainly support a structural interpretation rather than providing a direct validation of the transport metrics. The manuscript would benefit from explicitly stating this limitation in the main text, not only in the rebuttal.

Comment 10: Benchmarking claims appear overstated and should be corrected.
The manuscript still frames the membrane as outperforming state-of-the-art 2DFMs using language that appears too broad relative to the current literature landscape. Even if the reported values are impressive, the comparative claims should be narrowed and carefully qualified by test conditions, device geometry, and scaling level. The benchmarking language should be revised to avoid overstatement.

Comment 11: Some methodological details are still not fully transparent.
The revision adds many useful experimental details, but the measurement conditions are still not completely comprehensive, especially regarding reservoir handling and possible interfacial polarization control. Since these details can affect extracted electrokinetic and osmotic metrics, the Methods section would benefit from one final pass to ensure full clarity and consistency.

Version 2:

Reviewer comments:

Reviewer #2

(Remarks to the Author)
The manuscript is now close to publishable form and, in my judgment, satisfies the core Nature Communications threshold of a significant specialist advance supported by substantial evidence. The revision has meaningfully improved methodological transparency, leakage control, interpretive honesty around η_{EDL} and the simulation work, and the treatment of scale-up limitations. I do not see a remaining flaw that fatally undermines the central empirical result that the sV2DP membrane exhibits strong anion-selective transport and competitive osmotic-power performance.

That said, I would not recommend unconditional acceptance in its current wording. Before publication, the authors should make the final version fully internally consistent and more restrained in three places: first, everywhere label Σ as effective/apparent and t-based selectivity metrics as inferred where appropriate; second, explicitly acknowledge that the experimental sV2DP versus es-V2DP comparison is not a pure stacking-only control because chemistry/linkage differences remain; third, further narrow benchmarking and practical-performance claims so the distinction between micro-aperture peak

power density and larger-area/device-level output is unmistakable. I would also require a final editorial pass over figure captions, typos, and data/code availability wording.

If those final corrections are made carefully, the paper would be suitable for publication.

Open Access This Peer Review File is licensed under a Creative Commons Attribution 4.0 International License, which permits use, sharing, adaptation, distribution and reproduction in any medium or format, as long as you give appropriate credit to the original author(s) and the source, provide a link to the Creative Commons license, and indicate if changes were made.
In cases where reviewers are anonymous, credit should be given to 'Anonymous Referee' and the source.
The images or other third party material in this Peer Review File are included in the article’s Creative Commons license, unless indicated otherwise in a credit line to the material. If material is not included in the article’s Creative Commons license and your intended use is not permitted by statutory regulation or exceeds the permitted use, you will need to obtain permission directly from the copyright holder.
To view a copy of this license, visit https://creativecommons.org/licenses/by/4.0/

Point-to-point response to the comments from the reviewers

Reviewer #1

General comment. *This work reports a two-dimensional polymer membrane featuring stacked, triangular one-dimensional nanochannels for osmotic energy harvesting. The authors attribute the enhanced osmotic energy conversion performance to the combination of a high surface charge density and the stacked nanochannel architecture, which effectively reduces the effective pore size and thereby amplifies surface-charge-governed ion selectivity. The concept is technically sound and the introduction of an unique "screw-like" anion migration pathway—an aspect that has not been systematically explored in previous studies—offers a certain degree of novelty and conceptual interest. However, the membrane's power output is not particularly competitive when benchmarked against leading single-layer polymer systems. In addition, several of the design elements emphasized in the manuscript—most notably the influence of surface charge and the benefits of aligned nanochannel architectures—have been previously recognized in the literature. That said, the introduction of a screw-like anion migration pathway represents an interesting and less-explored structural feature, and this aspect could offer fresh insight and stimulate further progress in the field. The manuscript is clearly written and well structured. The material synthesis and characterization are competently executed. The performance evaluation and mechanistic interpretation require further clarification and revision. Several issues remain unresolved and should be addressed before the work can be considered for publication.*

Response: We are grateful to the reviewer for the thorough review and positive comments on our work. Point-by-point responses to all the specific comments raised have been provided below and changes have been made accordingly.

Comment 1. *On page 2, the author introduces a simplified conceptual expression to quantify the efficiency of EDL coverage. However, the physical meaning of this formula and the rationale behind its specific mathematical form are not clearly articulated. A more detailed explanation is needed to clarify how the expression is derived, what assumptions are embedded in it, and how it relates to the underlying ion-transport physics within the nanochannels.*

Response: We sincerely appreciate the reviewer's insightful comment and the opportunity to clarify the physical meaning and formulation of electrostatic double layer (EDL) coverage efficiency (η_{EDL}). In conventional nanofluidics, EDL overlap is typically assessed by

comparing the Debye length (λ_D) with a characteristic channel size. While provide a rough estimation of when (or how) electrostatic effects dominate ion transport (*Nat. Nanotechnol.* **2010**, 5, 848-852; *Nat. Rev. Mater.* **2021**, 6, 622-639), such a simplified descriptor becomes insufficient for two-dimensional framework membranes (2DFMs), including two-dimensional polymers (2DPs), two-dimensional covalent organic frameworks (2D COFs) COFs, and two-dimensional metal-organic frameworks (2D MOFs). In these systems, both the channel geometry and the charge distributions critically influence ion transport (*Nat. Rev. Methods Primers* **2024**, 4, 69), yet are not captured by a single λ_D-to-size comparison.

To address this limitation, we introduced η_{EDL} as a dimensionless, structure-aware scaling descriptor that combines electrostatic screening and geometric factors. Its purpose is to provide a physically intuitive measure of the fraction of the channel cross-section under strong EDL influence, thereby directly linking structural features to ion-transport behavior. Below, we further clarify its derivation, physical meaning, and key assumptions.

Derivation and physical meaning

In our model, discrete charge sites on the channel wall are treated as point charges that induce an electrostatic perturbation extending over a characteristic distance on the order of λ_D (Supplementary Fig. 2). The EDL-influenced region associated with a single charge site, projected onto the channel cross-section, is approximated as a half-disk of radius λ_D. The factor 0.5 reflects the skeleton charges; for spatially distributed charges within the channel volume, this factor becomes 1. The corresponding area (*A*) is defined as:

$$A = \frac{\pi \lambda_D{}^2}{2}$$

Let ρ denote the number density of charge sites per projected unit area; the total EDL-influenced area per projected unit is approximately ρA. Normalizing this by the effective cross-section area (*f(D)*), yields:

$$\eta_{EDL} = \frac{\rho \pi \lambda_D{}^2}{2 f(D)}$$

From a physical perspective, η_{EDL} represents the fraction of the transport cross-section in which electrostatic interactions strongly modulate ion distributions. For example, a higher η_{EDL} value indicates broader EDL coverage within the channels, which generally contributes to enhanced ion selectivity. In this way, η_{EDL} provides a direct, physically transparent bridge between the microscopic charge distribution (λ_D and ρ), the channel geometry (*f(D)*), and the macroscopic ion-transport properties.

1. Key assumptions

We emphasize that η_{EDL} is intended as a heuristic scaling parameter for guiding material and structure design, rather than a replacement for rigorous Poisson-Nernst-Planck descriptions or molecular dynamics (MD) simulations. The formulation is based on the following assumptions:

1) Applicable channel-size regime. The model applies to nanochannels with characteristic dimensions typically larger than ~ 1 nm, where continuum electrostatics and Debye-length-based electrostatic intuition remain valid (*Nat. Commun.* **2024**, 15, 3649; *Nat. Sustani.* **2025**, 8, 446-455).

2) Mean-field electrostatic screening. λ_D is directly taken from bulk electrolyte condition based on standard Debye screening, assuming a quasi-equilibrium EDL formation and neglecting strong ion-ion correlations or confinement-induced dielectric effects (*Natl. Sci. Rev.* **2025**,12, nwae439).

3) Discrete charge approximation. Surface charges are treated as discrete point charges, and their EDL-influenced region is approximated using a characteristic projected area defined by λ_D (*Chem. Rev.* **2024**, 124, 12391-12462).

4) Geometric projection simplification. The EDL influence is approximated through an effective 2D cross-section projection, without considering spatial variations, for simplified structural comparison.

<u>Actions:</u>

a) We have added the above discussion as Supplementary Note 1 in the revised Supplementary Information on Page 65.

b) We have highlighted the role of η_{EDL} in this work in the revised manuscript on Page 2.

'*To quantify the interplay between electrostatic screening and channel geometry in a structure-aware yet simple manner, we introduce an EDL coverage efficiency (η_{EDL}), a dimensionless, first-order design metric, defined as:*

$$\eta_{EDL} = \frac{\rho\pi{\lambda_D}^2}{2f(D)}$$

where ρ represents the number of charge sites per projected unit (reflecting ∑) and f(D) denotes the effective cross-sectional area available at given D. Conceptually, η_{EDL} estimates the fraction of the channel cross-section expected to lie within an EDL-dominated region, and higher η_{EDL} values imply stronger electrostatic effects and greater ionic selectivity. Note that this is a heuristic scaling descriptor intended for design guidance; its derivation, assumptions, and scope are detailed in Supplementary Note 1.'

Comment 2. *On page 2, please clarify how the function f(D) is defined and calculated in the author's analysis. In Supplementary Table 1, the values of f(D) reported for other literatures are consistently much larger than the corresponding pore diameter D, whereas for the membrane developed in this work, f(D) appears smaller than D. The author should explain the physical basis and methodological rationale for this discrepancy.*

Response: We thank the reviewer for pointing this out and apologize for the confusion in the previous version. The *D* value of 1.2 nm was only a rough geometric distance between two neighboring charge sites on adjacent walls, which was incorrectly used to represent the channel size.

In the revised manuscript, we have recalculated the channel size directly from the crystal structure and clarified the definition of *f(D)*. Specifically, *f(D)* is defined as the effective ion-accessible cross-sectional area of the channel (units, nm^2), while *D* is an effective diameter (units, nm) derived from the same cross-section. For the ABC-stacked sV2DP membrane, the channel window can be approximated as an equilateral triangle with side length a = 2.36 nm (Fig. R1). To obtain a physically meaningful descriptor for ion transport, instead of using the triangular side length directly, we adopt two commonly used equivalent diameters (*Adv. Mater.* **2024**, 36, 2310791, *J. Phys. Chem. B* **2021**, 125, 40, 11256–11263):

1) Inscribed-circle (effective electrostatic) diameter:

$$D_{in} = \frac{\sqrt{3}}{3} a \approx 1.36 \text{ nm}$$

2) Hydraulic diameter:

$$D_h = \frac{4A}{P} = \frac{\sqrt{3}}{3} a \approx 1.36 \text{ nm}$$

From an equilateral triangle, D_{in} = D_h. We therefore adopt D = 1.36 nm as a conservative estimate of the ion-accessible permeation diameter. The corresponding effective channel area is then calculated as:

$$f(D) = \pi \left(\frac{D}{2}\right)^2 \approx 1.46 \text{ nm}^2$$

With this revised definition of *D* and *f(D)*, the apparent discrepancy with other reported 2DFM systems is resolved. Notably, when evaluated on this unified basis, the ABC-stacked sV2DP membrane exhibits more pronounced electrostatic advantages compared to other state-of-the-art membranes, further supporting the structural superiority of sV2DP in enabling

selective ion transport (Supplementary Table 1).

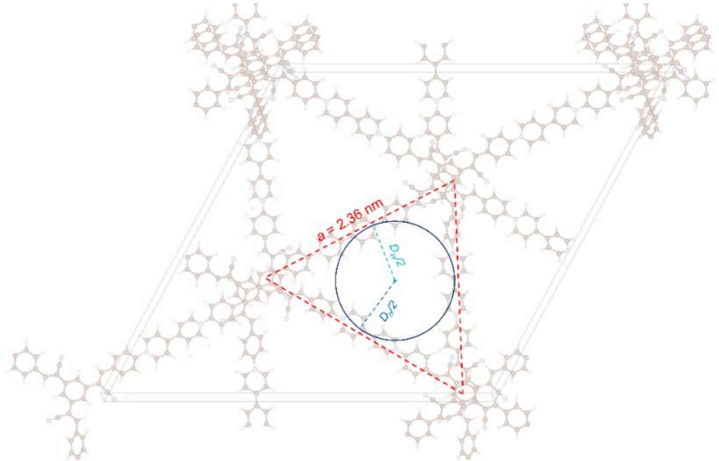

Fig. R1. Schematics of effective *D* (D_{in} and D_h) within the sV2DP units.

Actions:

a) We have corrected the reported pore sizes by using the effective electrostatic diameter (D_{eff}) throughout the revised manuscript.

'*Here, we report an ultrathin (~50 nm), fully crystalline, ABC-stacked, viologen-incorporated 2D polymer (sV2DP) membrane, which features vertically aligned triangular nanochannels (D_{eff} = ~1.36 nm) densely lined with pyridinium sites (+ 22.4 mC m^{-2}).*' (On page 1)

'*The sV2DP is fully crystalline, exhibiting face-on orientation with through-plane triangular nanochannels (D_{eff} = ~1.36 nm) and strongly positively-charged surfaces (Σ, + 22.4 mC m^{-2}).*' (On Page 3)

'*In contrast, a staggered interlayer arrangement (ABC stacking mode, s2DP) generates 1D triangular nanochannels along the* ***c****-axis with a drastically reduced size (D_2 = 1.36 nm) (Fig. 1b and Supplementary Fig. 1).*' (On Page 4)

'Computational modeling and experimental investigations indicate that its ultranarrow nanochannels (D_{eff} = ~1.36 nm) and strongly charged channel surface (Σ, + 22.4 mC m^{-2}) in sV2DP promote extensive η_{EDL}, intensifying the electrostatic effects and boosting anion-selective transport compared with its eclipsed counterpart.' (On Page 16)

b) We have added Fig. R1 as Supplementary Fig. 1 and included the corresponding discussion on the effective channel size of sV2DP in the revised Supplementary Information on Page 8.

'***Effective ion-accessible cross-sectional area (f(D)) and effective diameter (D) of sV2DP:***

As shown in Supplementary Fig.1, the channel window in the sV2DP units can be approximated as an equilateral triangle with side length a = 2.36 nm. For this geometry, instead of using the triangular side length directly, we characterize the channel using two commonly adopted equivalent diameters.

c) We have revised Supplementary Table 1 in the revised Supplementary Information on Page 68.

'Supplementary Table 1| Summary of characteristic D in reported 2DFMs for ion-selective transport

2DFMs	***D_{eff} (nm)***	***Geometry***	***f(D)(nm^2)***	***n^a***	***ρ (nm^{-2})^b***
2DPI[20,27]	*2.55*	*rectangle*	*6.5*	*4*	*0.62*
C2DP[34]	*3.05*	*rectangle*	*9.3*	*8*	*0.86*
V2DP[35]	*4.5*	*hexagon*	*50.29*	*12*	*0.24*
COF-DT[36]	*3.2*	*hexagon*	*8.04*	*6*	*0.74*
EB-2DP[19]	*2.6*	*hexagon*	*5.31*	*6*	*1.13*
PI-2DP	*2.6*	*hexagon*	*5.31*	*9*	*1.69*
Ca-COF[30]	*1.4*	*hexagon*	*1.53*	*3*	*1.96*
COF-170[37]	*2.3*	*hexagon*	*5.73*	*6*	*1.04*
**sV2DP** in this work	***1.36***	***triangle***	***1.46***	***6***	***4.11***

Note: All the data were directly extracted or adopted from the cited references. ^{a} n denotes the number of charge sites per framework unit. ^{b} ρ is calculated as the ratio of n and f(D).'

Comment 3. *Why the author chose to rely on a stacked architecture to reduce the effective pore size instead of directly fabricating membranes with intrinsically smaller nanochannel diameters. What specific advantages does the stacked configuration provide over simply designing smaller straight-through pores? A justification is needed, given that channels with genuinely small diameters would, in principle, offer more straightforward control over selectivity and may support faster ion transport.*

Response: Thanks for the valuable comment. While, in principle, reducing the intrinsic pore diameter is an effective route to enhance ion selectivity, the adoption of a staggered stacked architecture in our system is motivated by both structural constraints and distinct transport advantages that cannot be achieved by simply shrinking straight-through pores:

1) Decoupling the trade-off between charge density and pore aperture

In crystalline 2DFMs, there exists an inherent structural trade-off between high charge density and small pore size. Incorporating densely packed ionic groups (e.g., sulfonic groups, quaternary ammonium) typically requires sufficient molecular spacing to maintain structural integrity. Attempting to force such bulky, highly charged groups into intrinsically nanometer pores often results in severe steric hindrance, disrupting both in-plane and out-of-plane structural order (*J. Am. Chem. Soc.* **2022**, 144, 12400-12409; *J. Am. Chem. Soc.* **2023**, 145, 27984-27992). In contrast, the staggered stacked architecture allows the in-plane framework structure and the out-of-plane transport channel to be regulated more independently. This enables to retain high crystallinity of the 2DP lattice while achieving a high structural charge density (ρ), which surpasses most previously reported 2DFMs (see Supplementary Table 1).

2) Enhanced directional transport via an 'offset' electrostatic landscape

The staggered stacked architecture introduces a fundamentally different mechanism by creating a spatially offset electrostatic landscape along the transport direction. Specifically, the sequential displacement of charged sites in the ABC stacking generates a non-uniform, periodically modulated potential field, in contrast to the relatively uniform electrostatic environment in straight-through pores (Supplementary Fig. 31). Molecular dynamics (MD) simulations reveal that this configuration gives rise to a 'screw-like' transport pathway, in which counterions follow a continuous, energetically favorable trajectory through the channel, effectively lowering the transport energy barrier (Fig. 3c-e). To further verify the generality of this structure-induced transport property, we analyzed the trajectories of different counterions (e.g., Cl^-, Br^-, I^-) using MD simulations. We found that all these counterions display 'screw-like' transport behavior within the stacked sV2DP nanochannels (Fig. R2), which confirms that this phenomenon is structure-induced rather than a specific effect associated with any individual ion. This behavior goes beyond the typically random transport observed in AA-stacked 2DPs, where ions typically undergo random, 3D diffusion with minimal modulation of the electrostatic landscape.

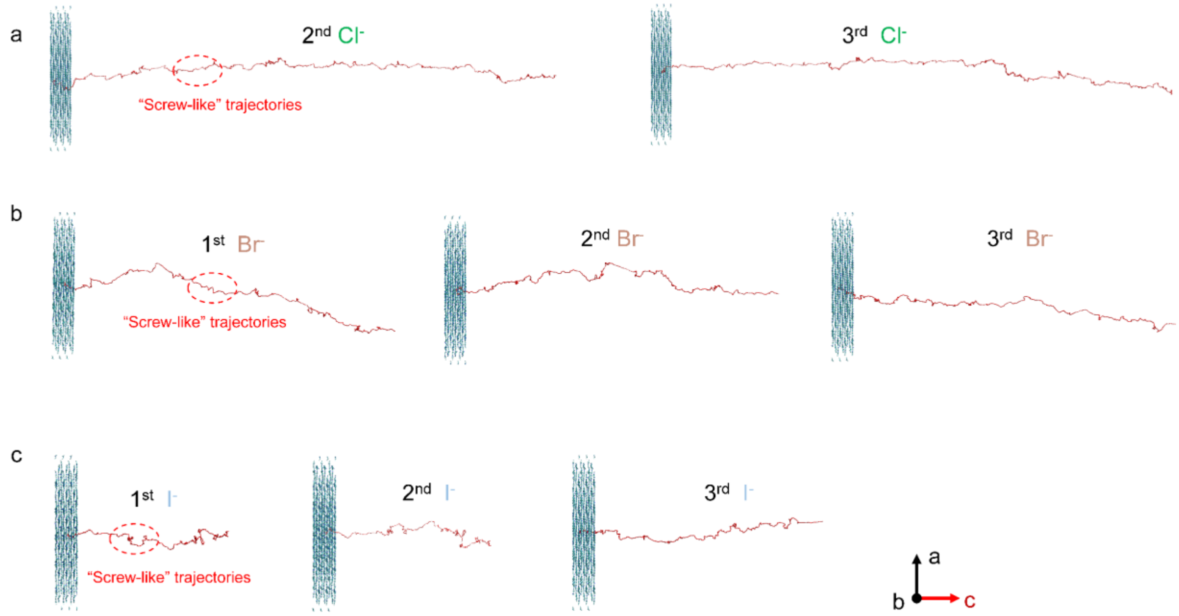

Fig. R2. Trajectory visualizations of halide-ion transport in sV2DP. Representative trajectories of (a) Cl^-, (b) Br^-, and (c) I^- obtained from three independent MD runs in the sV2DP channel. The red traces show the projected ion transport paths, highlighting reproducible 'screw-like' motion along the c-axis.

Actions:

a) We have added the above discussions in the revised manuscript on Page 3.

'However, most reported 2DFMs either feature relatively wide 1D nanochannels (D > 1.5 nm) and/or sparsely distributed charges (low ρ), reflecting a practical trade-off between pore narrowing and dense incorporation of ionic sites in crystalline frameworks.[26,27]. As a result, the achievable η_{EDL} is often limited, leading to only moderate permselectivity and constraining overall membrane performance (Supplementary Table 1)'

'The ABC-stacked sV2DP architecture not only enhances EDL coverage (up to a 3.3-fold enhancement in η_{EDL}) by effectively narrowing the transport cross-section, but also organizes pyridinium charges into a staggered distribution along the transport direction. Molecular dynamics (MD) reveal that this structured electrostatic landscape guides anions into a characteristic "screw-like" migration pathway along the ***c****-axis, in contrast to the more random trajectories observed in non-staggered 2DP analogues.'*

b) We have added Fig. R2 as Supplementary Fig. 33 in the revised Supplementary Information on Page 43.

Comment 4. *In Supplementary Fig. 12, the author claims that the membrane exhibits pronounced ion rectification. However, the reported rectification ratio is only 1.12, which is extremely small and, in my view, insufficient to substantiate the presence of a meaningful rectification effect. In addition, it is unclear how ion rectification could arise in the present system given that the fabricated membrane is structurally symmetric. The supporting substrate contains large (~2 μm) pores whose surface charge is unlikely to impose any appreciable regulation on ion transport, and the overall membrane architecture appears symmetric with respect to both pore size and surface charge along the transport direction. The authors should explain the origin of the observed asymmetric current response under these conditions.*

Response: We thank the reviewer for this constructive comment. We agree that the rectification ratio (~1.12) is modest. We have therefore revised the text to avoid overstating this effect. The purpose of Supplementary Fig. 12 (Supplementary Fig. 13 in the revised version) is only to highlight a detectable polarity dependence in the *I-V* characteristics after integrating the sV2DP membranes, rather than to claim pronounced rectification.

Regarding the origin of the observed asymmetry in the *I-V* curves, we attribute the rectification to the coupling between the charged sV2DP pores and the SiN_x substrate pore. Although the substrate pores are large (~2 μm) and structurally symmetric, the introduction of sV2DP creates effectively asymmetric ionic boundary conditions along the transport direction. Under opposite bias polarities, these boundary conditions result in differing degrees of concentration polarization (CP), leading to a polarity-dependent current response. Importantly, the bare SiN_x aperture shows a fully symmetric *I-V* shape, indicating that the substrate alone does not contribute to rectification (Supplementary Fig. 13 and 14a). The rectification only appears after introducing the sV2DP membrane, consistent with a CP-driven rectification mechanism reported in other ion-selective nanochannel/membrane systems (*Nat. Nanotechnol.* **2022**, 17, 622-628; *Nat. Commun.* **2022**, 13, 3935).

Actions:

a) We have revised the related statements in the revised manuscript on Page 7.

'***Selective ion transport in sV2DP membrane.*** *For electrochemical measurements, the as-synthesized sV2DP membrane was transferred and sealed over a 2 μm-diameter aperture on a*

SiN_x substrate (Supplementary Fig. 11). The measured current-voltage (I-V) curve exhibits ion-rectification behavior, which is characteristic of charged pores in the sV2DP membrane (Supplementary Fig. 13).'

b) We have also added the above discussions in the revised Supplementary Information on Page 22.

'***Supplementary Fig. 13| a***, *I–V curves recorded in 0.1 M KCl for the bare SiN_x substrate (control) and after integration with a sV2DP membrane.* ***b***, *Corresponding rectification ratio ($R = |I_+/I_-|$ at ± 200 mV). Note that this rectification behavior is attributed to the coupling between the sV2DP membrane pores and the SiN_x substrate pore, which creates effectively asymmetric ionic boundary conditions and thus different degrees of concentration polarization (CP) under opposite bias polarities.*

Comment 5. *In Supplementary Fig. 13, the manuscript presents ion conductance curves, but it is not clear how the conductance values were calculated. Was the author accounting for the purported ion rectification in this analysis? If rectification is indeed present, the conductance in the forward and reverse directions should differ. How, then, were the conductance values extracted or averaged? A detailed explanation of the calculation procedure is required.*

Response: Thanks to the reviewer for raising these questions. In Supplementary Fig. 13 (now Supplementary Fig. 14 in the revised version), the conductance was extracted from the ohmic (linear) region of the *I-V* curve. Specifically, we performed a linear fit within a small bias window from −50 to +50 mV, where the current response remains essentially linear and any concentration-polarization-induced nonlinearity is negligible. Then, the conductance was defined as the slope of the fitted line,

$$G = \frac{dI}{dV}$$

This single linear fit over the low-bias range allows for consistent, reproducible conductance measurements across 6 different samples (e.g., Fig. R3). Therefore, complications arising from rectification at higher voltages can be avoided, ensuring that the measured conductance values reflect the intrinsic ion transport properties of the sV2DP membrane.

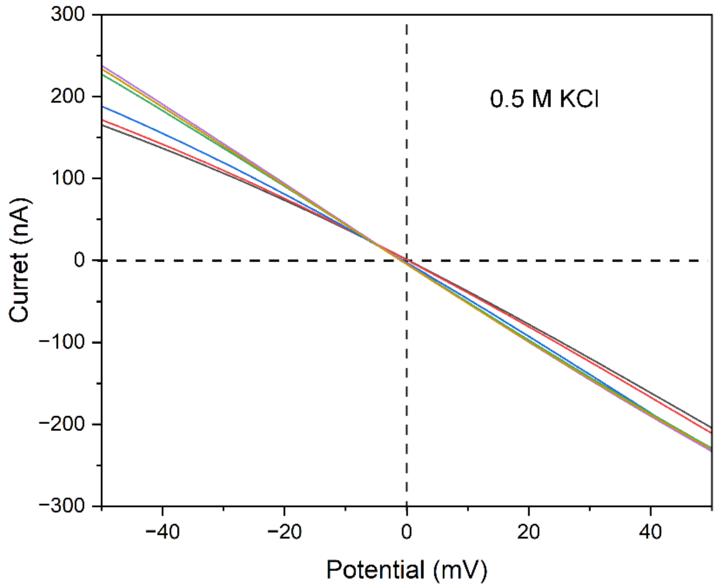

Fig. R3. Multiple *I-V* curves of the sV2DP membranes recorded from 6 independent measurements in 0.5 M KCl solution within a small bias window of -50 to +50 mV.

Actions:

We have added the discussion above to the revised manuscript on Page 20.

'***Ion transport measurements***

... where A is the effective membrane area. To extract the intrinsic conductance of the sV2DP membranes while avoiding CP-induced nonlinear behavior, the conductance was determined from the ohmic (linear) region of the I-V curve by performing a linear fit within a narrow bias window of -50 to +50 mV, using:

$$G=\frac{dI}{dV}$$

The ion selectivity coefficient (S) was calculated using the following equation.[53]'

Comment 6. *In Fig. 2b, the trend in the measured Zeta potentials at different electrolyte concentrations is not clearly explained. Theoretically, higher ionic strength should lead to stronger screening of surface charges, and one would therefore expect a decreasing trend. The authors should clarify the physical mechanism underlying the observed behaviors.*

Response: We appreciate the reviewer's insightful comments. In the previous version, the 'Zeta potential' values in Fig. 2b were back-extracted from the streaming-current response using a simplified electrokinetic relation, rather than being a direct measurement of the

interfacial potential. We acknowledge that such a classical interpretation becomes unreliable in ultranarrow nanochannels, where strong EDL overlap, ion-specific effects, and surface conduction can invalidate the underlying assumptions. Therefore, the concentration dependence observed in Fig. 2b should be interpreted as an effective electrokinetic parameter, instead of a literal surface (Zeta) potential in the conventional sense.

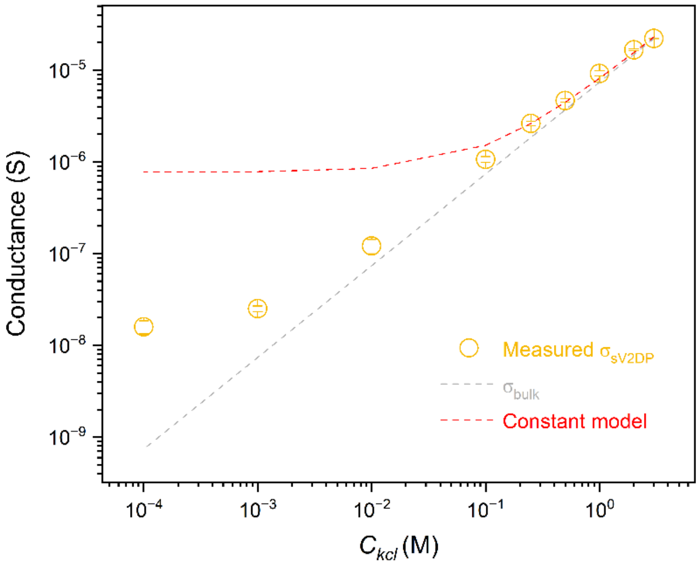

Fig. R4. Transmembrane conductance of the sV2DP membrane as a function of KCl concentration (C_{KCl}). The red curve shows the theoretical fit using a constant-charge model (Σ = 22.4 mC m^{-2}). Data are mean ± s.d, and were obtained from 6 independent measurements.

To provide a more physically grounded analysis, we have revised our charge-density extraction by applying concentration-dependent conductance fitting using a model that explicitly includes surface-conduction contributions (Supplementary Note 3 and Figure R4). This approach provides a more reliable estimate of the effective fixed-charge density and avoids over-interpreting the classical Zeta potential trend. Using this model, sV2DP exhibits a high surface charge density of ~ 22.4 mC m^{-2} in 0.25 M KCl, which is significantly higher than that of typical 2DFMs (< 10 mC m^{-2}) (*Nat. Nanotechnol.* **2022**,17, 622-628; *Adv. Mater.* **2024**, 36, 2310791; *Nat. Sustani.* **2025**, 8, 446-455). We have incorporated these clarifications in the revised manuscript and present the updated charge-density values accordingly.

Actions:

a) We have added the surface-charge-density analysis as Supplementary Note 3 in the revised Supplementary Information on Page 64.

‘***Supplementary Note 3| Surface-charge-density analysis for ultranarrow sV2DP nanochannels***

To quantify the surface charge density (Σ) of sV2DP nanochannels, we analyze the ionic conductance measured under symmetric electrolyte conditions (identical KCl concentrations on both sides of the membrane). The total conductance(G) is modeled as the sum of a bulk conductance (G_{bulk}) and a surface conductance ($G_{surface}$) for ultranarrow triangular nanochannels in sV2DP:

$$G=G_{bulk}+G_{\ surface}=N_{ch}\left(\frac{q(\mu^{+}+\mu^{-})C_B N_A wh}{2l}+\frac{\mu^{-}\Sigma w}{l}\right)$$

where q is the elementary charge (1.60217663 × 10^{-19} C); μ^{+} represents the mobility of K^{+} (7.6 × 10^{-8} m^2 s^{-1} v^{-1}); μ^{-} refers to the mobility and Cl^{-} (7.9 × 10^{-8} m^2 s^{-1} v^{-1}); C_B is the bulk electrolyte concentration; N_A is Avogadro's number (6.023 × 10^{23}); h and w are the side length and height for the triangular nanochannel cross-section (2.36 and 2.04 nm, respectively); and l is the thickness of membranes (50 nm).

In concentrated electrolyte (3 M KCl), EDLs are strongly screened, and the conductance is dominated by G_{bulk}. At lower concentrations (10^{-4} to 1 M), the surface contribution becomes significant, leading to deviations from linear (bulk-dominated) scaling. Thus, the conductance at 3 M KCl is used to determine the effective number of conducting (charged) channels (N_{ch}), assuming G = G_{bulk} under strong screening. With the channel number fixed, experimental conductance values across KCl concentrations are then fitted using the above equation to extract the Σ value for sV2DP nanochannels. As a result, surface charge densities of ~ 22.4 and 10.6 mC m^{-2} for sV2DP in 0.25 M KCl and 0.1M KCl, respectively, which are stronger than reported charged 2DFMs (< 10 mC m^{-2}).[19,30-32]’

b) Fig. R4 is presented as Figure 2a in the revised manuscript on page 9.

Comment 7. *Please provide the redox potential data corresponding to Supplementary Fig. 18. It is unclear why the blank substrate does not exhibit any measurable redox potential under a tenfold concentration gradient. Furthermore, the I–V curves shown in this figure cover only a very narrow voltage range. Please supply the full I–V curves and explain the origin of the deviation from linearity observed in their shape.*

Response: Following the reviewer's suggestion, we have provided the full *I-V* curves corresponding to Supplementary Fig. 18 (now presented as Fig. R5), instead of only the low-

bias region. The observed nonlinearity in the *I-V* curves is attributed to ion concentration polarization (ICP) under asymmetric electrolyte conditions. The two sides of the membrane experience different ionic strengths, leading to unequal EDL thicknesses. When a bias is applied, this asymmetry induces local ion enrichment or depletion near the membrane entrances, leading to a deviation from linearity at higher voltages (*Nano Lett.* **2007**, 7, 10, 3165-3171). Such ICP-induced nonlinearity is consistent with highly charged, permselective nanochannel transport governed by EDL. Similar behavior has been reported in other charged nanochannel and membrane systems (*Science* **2017**, 358, 511-513; *Sci. Adv.* **2023**, 9, eadi8493).

Regarding the redox potential of the blank SiN_x substrate under a tenfold concentration gradient, our measurements were performed using Ag/AgCl electrodes coupled with 3 M KCl salt bridges to minimize contributions from liquid junction potentials. Since the blank substrate is structurally symmetric and non-selective, it does not sustain ion-selective transport or concentration polarization. Consequently, no significant electrochemical potential difference develops across the membrane, and the measured open-circuit voltage (V_{oc}) value is close to 0, consistent with the absence of a measurable redox or diffusion potential.

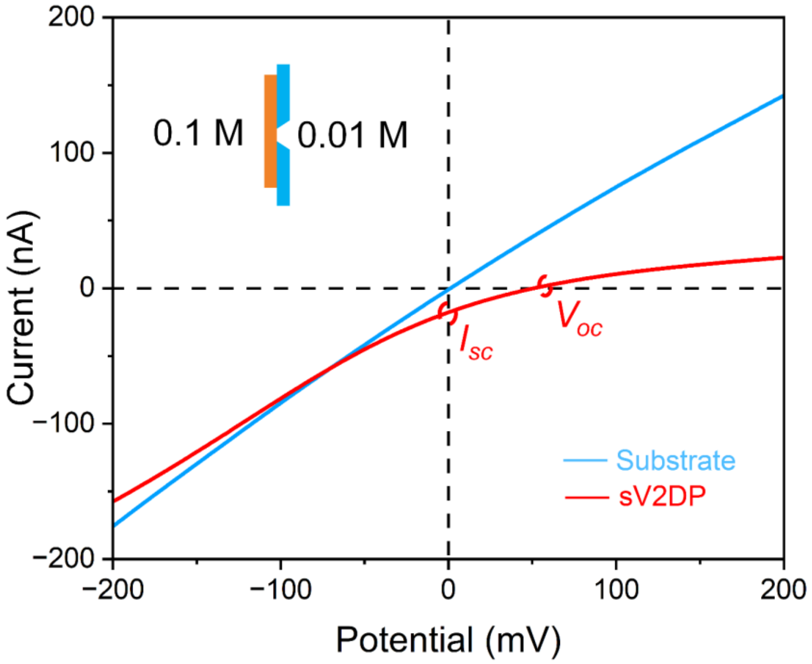

Fig. R5. *I-V* curves of the substrate before and after being covered with an sV2DP membrane under a 10-fold KCl gradient.

Actions:

a) Fig. R5 is presented as Supplementary Fig. 16 in the revised Supplementary Information on Page 25.

b) We have added the following discussion regarding the junction potential in the revised manuscript on Page 19.

'*Standard Ag/AgCl electrodes with 3 M KCl salt bridges (CHI111) were used to record voltage and current signals, effectively eliminating junction potential caused by salt concentration differences.*'

Comment 8. *For the MD simulations presented in Fig.3, it is not clear whether the calculations account for the relationship between the hydrated ion size and the pore size. The pore diameter of the eV2DP membrane appears to be comparable to the size of the hydrated ions themselves. Under such pore–ion size matching conditions, the reduction of the effective pore size arising from the stacked configuration may not yield a substantial enhancement in ion-selective transport. The author should clarify how hydration effects were treated in the simulations and discuss the implications for the claimed performance improvement.*

Response: We thank the reviewer for this suggestion. In our MD simulations, hydration is treated explicitly: all ions are fully solvated in a box of TIP3P water molecules, with water-water and water-ions interactions described by Coulombic and Lennard-Jones potentials. As such, the hydration structure around each ion emerges naturally from the simulation and is not imposed a priori.

Regarding the concern about pore-ion size matching, the effective pore size of the ABC-stacked sV2DP is estimated to be ~ 1.36 nm (Fig. R1), substantially larger than the hydrated diameters of the halide ions studied. For comparison, the eV2DP membrane has an even larger effective pore diameter of ~ 4.4 nm (Supplementary Fig. 27). The hydrated diameters of the Cl^-, Br^-, I^- are all well below 10 Å (Cl^-: 6.2 Å; Br^-: 6.6 Å; I^-: 7.0 Å, Supplementary Table. 2), as confirmed by radial distribution functions *g(r)* between ions and water molecules (Fig. R6), consistent with literature reports (Supplementary Table. 4). Therefore, the system is not a strict pore-ion size matching regime, and ions are expected to transit the pore without significant dehydration. Consequently, the observed ion selectivity in both experiment and simulation cannot be attributed to steric size exclusion or differential dehydration, but rather to differences in ions-framework interactions along the transport pathway, as discussed in our response to *Comment 3*.

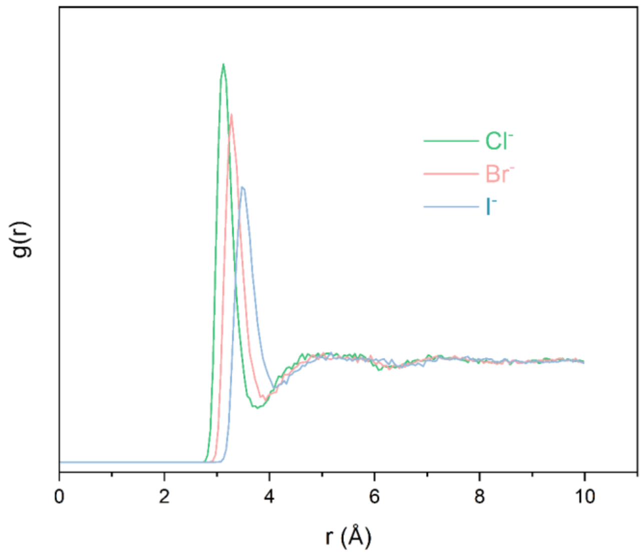

Fig. R6. Radial distribution functions (*g(r)*) between halide ions (Cl^-, Br^-, I^-) and water oxygen atoms in aqueous solution computed using TIP3P water and Joung-Cheatham ion parameters. The first-peak positions (in Å) increase from Cl^- to Br^- to I^-, consistent with the expected trend in hydration structure.

<u>Actions:</u>

a) We have added the above discussion in the revised Supplementary Information on Page 41.

'*All simulations were performed using the Amsterdam Modeling Suite (AMS 2024), with parameters derived from the UFF4MOFII force field. TIP3P model for water and Joung–Cheatham parameters for halide ions were used.*[26] *Visualization of trajectory files and anion motion tracking were carried out using VMD 1.9.3. Analysis of trajectory files, including the extraction of interaction statistics between anions and adsorption sites, was conducted using the MD Analysis package. The cut-off distance of interaction between the anions and absorption sites was set as 5.6 Å. For each step, the number of anions that fell in the interaction range within the adsorption sites was counted. Kernel Density Estimation (KDE*

[26] I. S. Joung, T. E. C. Determination of alkali and halide monovalent ion parameters for use in explicitly solvated biomolecular simulations. J. Phys. Chem. B 112, 9020-9041 (2008).'

b) We have presented Fig. R6 as Supplementary Fig. 32 in the revised Supplementary Information on Page 42.

c) Supplementary Table 4 has been updated by including the first peak, coordination number and reported value ranges on Page 71.

Supplementary Table 4| Hydration-shell metrics extracted from g(r)

Ion	***First peak (Å)***	***Coordination number***	***Reported experimental range***	***Ref.***
Cl^-	*3.1*	*7.0*	*4.0-8.9*	[55,56]
Br^-	*3.3*	*6.9*	*6.0-7.4*	[57]
I^-	*3.5*	*7.6*	*4.2-10.3*	[55,56]

[55] Marcus, Y. Effect of Ions on the Structure of Water: Structure Making and Breaking. Chem. Rev. ***2009****, 109, 1346–1370.*

[56] Migliorati, V.; D'Angelo, P.; Sessa, F. Going beyond Radial Hydration Models: The Hidden Structures of Chloride and Iodide Aqua Ions Revealed by the Use of Lone Pairs. J. Phys. Chem. B ***2023****, 127, 10843–10850.*

[57] D'Angelo, P. et al. Hydration Properties of the Bromide Aqua Ion: the Interplay of First Principle and Classical Molecular Dynamics, and X-ray Absorption Spectroscopy. Inorg. Chem. ***2010****, 49, 4224–4231.*

Reviewer #2

General comment. *This manuscript reports an ultrathin (~50 nm), fully crystalline, ABC-stacked viologen-incorporated 2D polymer membrane (sV2DP) featuring vertically aligned triangular nanochannels (reported D ≈ 1.2 nm) with densely charged pyridinium sites (reported ~67.1 mC m⁻²). The authors argue that the staggered (ABC) stacking effectively narrows the transport pathway, increases electric double-layer (EDL) overlap/"coverage efficiency," and thereby boosts anion selectivity and permeability. The membrane is integrated into osmotic power generators and claimed to deliver very high outputs (e.g., 243 W m⁻² under a 50-fold NaCl gradient) and a macroscopic aperture-array device reaching ~46.7 W m⁻² under artificial seawater/river-water conditions with multi-day stability. The concept is interesting and potentially impactful, but several central quantitative claims (surface charge density, selectivity metrics, and area-normalized power density) rely on assumptions that are not convincingly justified for ~1 nm channels with strongly overlapping EDLs and possible surface conduction. Substantial clarification and additional validation are needed.*

Response: We thank the reviewer for careful evaluation of our manuscript and for the constructive suggestions. We have addressed all comments thoroughly and revised the manuscript accordingly to improve its clarity and quality.

Comment 1. *Surface charge density derived from electrokinetic analysis appears unreliable for ~1 nm channels. The manuscript reports a high surface charge density and uses it as a key mechanistic pillar. However, the pathway "streaming current/streaming potential → zeta potential → surface charge density" is typically derived under assumptions that are questionable in ultra-confined channels with strong EDL overlap, ion-specific effects, and potentially significant surface conduction. In this regime, the extracted zeta potential may not represent a physically meaningful interfacial potential in the usual sense, and converting it to surface charge density via classical relations can produce large systematic errors. What I need to see: a regime-appropriate electrokinetic model (explicitly including/estimating surface conductance effects and overlap) and at least one independent cross-validation of charge density (e.g., counterion uptake/charge titration, stoichiometry + accessible site density constraints, or concentration-dependent conductance fitted with a physically grounded model rather than a single-point zeta conversion).*

Response: We thank the reviewer for raising these concerns. We fully agree that the classical pathway of ‘streaming current/streaming potential → zeta potential → surface charge density’

(i.e., ζ-to-charge) may become unreliable in ~1 nm-sized channels, where strong EDL overlap, ion-specific effects, and surface conduction can invalidate the assumptions underlying conventional electrokinetic analysis. In the revised manuscript, we have explicitly clarified these limitations and avoided over-highlighting the extracted Zeta potential as a physically meaningful interfacial quantity in this regime.

At the same time, independent of any specific model, the large measured streaming current response itself provides direct evidence of substantial counterion enrichment within the channel. Under an applied pressure gradient, the advective transport of these excess counterions generates a pronounced streaming current. The observed magnitude of this response, which exceeds that reported for other 2DFM systems (*J. Am. Chem. Soc.* **2023**, 145, 17786-17794), together with its systematic concentration dependence, is consistent with a high fixed-charge density and extensive EDL coverage/overlap.

To address the reviewer's request for a regime-appropriate model, we have re-evaluated the effective charge density using concentration-dependent conductance measurements over a wide ionic-strength range, fitted with a constant charge model that explicitly includes surface-conduction contributions (Supplementary Note 3 and Fig. R4). This approach captures key nanoscale effects neglected in classical analysis, thereby providing an independent, self-consistent estimate of the effective fixed-charge density. The resulting value of 22.4 mC m^{-2} for sV2DP remains significantly higher than those reported for typical 2DFMs (< 10 mC m^{-2}) (*Nat. Nanotechnol.* **2022**,17, 622-628; *Adv. Mater.* **2024**, 36, 2310791; *Nat. Sustani.* **2025**, 8, 446-455), corroborating the conclusion that the sV2DP carries a high density of surface charges. These clarifications and the revised analysis have been incorporated into the manuscript to ensure a more rigorous and physically consistent interpretation of the electrokinetic data.

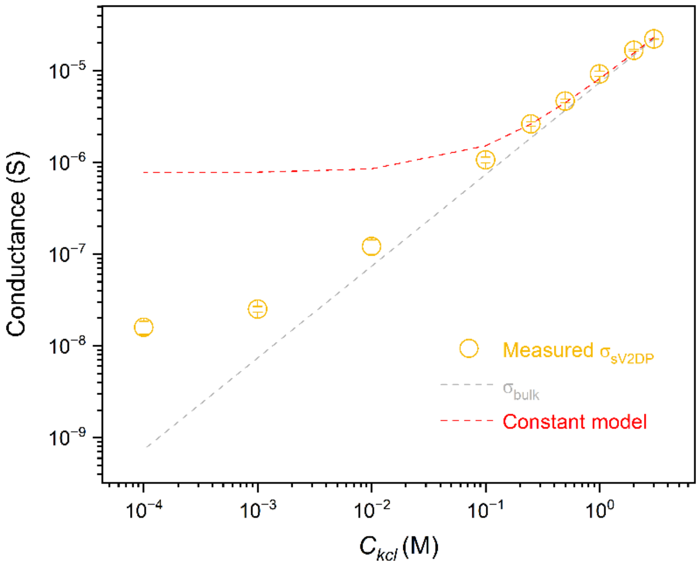

Fig. R4, Transmembrane conductance of the sV2DP membrane as a function of KCl concentration (C_{KCl}). The red curve shows the theoretical fit using a constant-charge model (Σ = 22.4 mC m^{-2}). Data are mean ± s.d, and were obtained from 6 independent measurements.

Actions:

a) We have added the above discussion and provided a new version of concentration-dependent conductance for calculating the effective surface charge density using a constant charge model in the revised manuscript on Page 7.

'*The Σ value of sV2DP is further estimated to be + 22.4 mC m^{-2} using a constant charge model (Supplementary Note 2), significantly higher than those of typical 2DFMs.*'

b) Fig. R4 is presented as Figure 2a in the revised manuscript on page 9.

c) The surface charge density analysis has been added as Supplementary Note 3 in the revised Supplementary Information on Page 67.

'***Supplementary Note 3| Surface-charge-density analysis for ultranarrow sV2DP nanochannels***

To quantify the surface charge density (Σ) of sV2DP nanochannels, we analyze the ionic conductance measured under symmetric electrolyte conditions (identical KCl concentrations on both sides of the membrane). The total conductance(G) is modeled as the sum of a bulk conductance (G_{bulk}) and a surface conductance ($G_{surface}$) for ultranarrow triangular nanochannels in sV2DP:

$$G = G_{bulk} + G_{surface} = N_{ch}\left(\frac{q(\mu^{+}+\mu^{-})C_B N_A wh}{2l} + \frac{\mu^{-}\Sigma w}{l}\right)$$

where q is the elementary charge (1.60217663 × 10^{-19} C); μ^+ represents the mobility of K^+ (7.6 ×10^{-8} m^2 s^{-1} v^{-1}); μ^- refers to the mobility and Cl^- (7.9 ×10^{-8} m^2 s^{-1} v^{-1}); C_B is the bulk electrolyte concentration; N_A is Avogadro's number (6.023 × 10^{23}); h and w are the side length and height for the triangular nanochannel cross-section (2.36 and 2.04 nm, respectively); and l is the thickness of membranes (50 nm).

In concentrated electrolyte (3 M KCl), EDLs are strongly screened, and the conductance is dominated by G_{bulk}. At lower concentrations (10^{-4} to 1 M), the surface contribution becomes significant, leading to deviations from linear (bulk-dominated) scaling. Thus, the conductance at 3 M KCl is used to determine the effective number of conducting (charged) channels (N_{ch}), assuming G = G_{bulk} under strong screening. With the channel number fixed, experimental conductance values across KCl concentrations are then fitted using the above equation to extract the Σ value for sV2DP nanochannels. As a result, surface charge densities of ~ 22.4 and 10.6 mC m^{-2} for sV2DP in 0.25 M KCl and 0.1M KCl, respectively, which are stronger than reported charged 2DFMs (< 10 mC m^{-2}).[19,30-32]'

Comment 2. *"EDL coverage efficiency" (η_EDL) requires stronger physical grounding and validation. The proposed η_EDL metric is presented as a unifying descriptor that explains performance gains. At present, it reads as a heuristic quantity tuned to this specific geometry, with limited evidence that it is predictive rather than descriptive. In highly confined ~1 nm channels, Debye-length-based intuition can break down (finite-ion size, correlations, dielectric effects), so any scalar "coverage efficiency" must be carefully justified.*

What I need to see: sensitivity analysis showing how η_EDL changes with the assumed effective diameter/cross-section and how conclusions hold if screening deviates from ideal Debye behavior. Ideally, demonstrate predictive capability across multiple salts, ionic strengths, and pH values (not just one best condition).

Response: Following the reviewer's suggestion, we have clarified the physical basis, assumptions, and intended scope of η_{EDL}, and included additional sensitivity analyses in the revised manuscript to demonstrate that our conclusions are not dependent on a single geometric assumption.

1. Physical grounding and intended scope.

In the revised manuscript, we have explicitly defined η_{EDL} as a dimensionless first-order design descriptor for 2DFMs with well-defined 1D nanochannels (e.g., 2DP, 2D COF membranes). In these systems, the characteristic channel sizes are typically > 1 nm and larger than hydrated-ion diameters, such that ion selectivity is primarily governed by the extent of EDL coverage/overlap. Under this mean-field electrostatic approximation, the Debye length (λ_D) provides a reasonable first-order estimate of the screening range and the spatial extent of electrostatic perturbations near charged sites. We emphasize that η_{EDL} is not intended to replace classic Poisson-Nernst-Planck (PNP) or molecular dynamics (MD) simulations, but rather to serve as a structure-guided scaling metric. Compared with conventional 'pore size vs. λ_D' criterion (*Nat. Rev. Mater.* ***2021***, *6*, *622-639* and *Nat. Rev. Methods Primers* ***2024***, *4*, *69*), η_{EDL} provides a more explicit way to quantify how channel geometry and charge distribution collectively influence electrostatic coverage, thereby enabling rational comparison across different 2DFM architectures to guide membrane design and to rationalize trends across 2DFMs configurations (Supplementary Note 1).

2. Sensitivity analysis.

To address concerns regarding geometric dependence, we have clarified the definition of *f(D)* as the ion-accessible transport cross-sectional area and performed a sensitivity analysis using alternative, physically reasonable geometric sizes for the same channel apertures, including: (i) an inscribed-circle (effective electrostatic) aperture; (ii) a hydraulic-diameter-based aperture (Fig. R1). While the absolute value of η_{EDL} changes modestly across these definitions, the relative ordering of different structures is preserved, and the key conclusion that the stacked architecture enhances η_{EDL} compared to the non-stacked remains unchanged.

3. Robustness to deviations from ideal Debye screening.

We acknowledge that classical Debye screening may deviate from ideal behavior in highly confined systems. In this work, we adopt Debye screening as a first-order approximation for channels >1 nm, consistent with previous studies (*Nat. Commun.* **2024**, 15, 3649; *Nat. Sustani.* **2025**, 8, 446-455; *Natl. Sci. Rev.* **2025**,12, nwae439). Note that η_{EDL} is introduced primarily for structure comparison within a fixed-charge regime. In our system, the viologen units are permanently cationic (*Angew. Chem. Int. Ed.* **2024**, e202316299), and the effective fixed-charge density (ρ) is only weakly dependent on pH over the tested range (pH 4-9) (Fig. R7). Under these conditions, variations in ionic strength affect η_{EDL} only through λ_D ($\eta_{EDL} \sim \lambda_D^2$), which acts as a common multiplicative factor for all structures. Thus, the relative rankings and the structural design conclusions remain preserved, even if deviations from ideal screening occur. We do not position η_{EDL} as a universal predictor across arbitrary salt types or pH

conditions, where ρ may vary due to specific adsorption or acid-base equilibria. Instead, it serves as a practical scaling metric to rationalize trends and guide optimization within the fixed-charge regime.

We have incorporated these clarifications into the revised manuscript and supplementary information to address the reviewer's concerns, while preserving η_{EDL} as a concise, physically grounded descriptor for guiding the design of ion-selective transport in 2DP and 2D COF membranes.

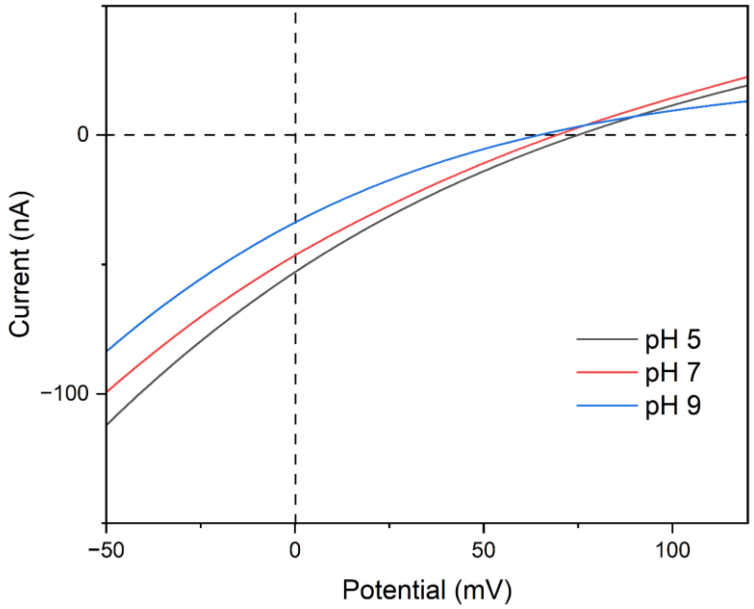

Fig. R7. pH-dependent ion-selective transport of the sV2DP membrane. Ion-selective measurements of the sV2DP membrane were performed at pH 5, 7, and 9 under the 0.5/0.01 M KCl gradients.

<u>Actions:</u>

a) We have included the above discussion as Supplementary Note 1 in the revised Supplementary Information on Page 65.

b) We have highlighted the role of η_{EDL} in the revised manuscript on Page 2.

'*To quantify the interplay between electrostatic screening and channel geometry in a structure-aware yet simple manner, we propose an EDL coverage efficiency (η_{EDL}), a dimensionless, first-order design metric, defined as:*

$$\eta_{EDL} = \frac{\rho \pi {\lambda_D}^2}{2f(D)}$$

where ρ represents the number of charge sites per projected unit (reflecting ∑) and f(D) denotes the effective cross-sectional area available at given D. Conceptually, η_{EDL} estimates the fraction of the channel cross-section expected to lie within an EDL-dominated region, and higher η_{EDL} values imply stronger electrostatic effects and greater ionic selectivity. Note that this is a heuristic scaling descriptor intended for design guidance, and the derivation, assumptions, and scope are provided in Supplementary Note 1.'

c) Fig. R7 is presented as Supplementary Fig. 26 in the revised Supplementary Information on Page 35.

Comment 3. *Power density and current density normalization: effective area/leakage/edge pathways must be ruled out. The reported selective current density (e.g., 13.2 kA m^{-2} under a 50-fold KCl gradient) and power density (e.g., 243 W m^{-2} under 50-fold NaCl) are exceptionally high for a membrane system, making rigorous validation essential. For ultrathin membranes on apertures, small defects, imperfect sealing, or edge leakage can dominate current while preserving "reasonable-looking" I–V curves. A bare-substrate control is not sufficient.*

What I need to see: a) Statistics across many devices (n, yield, distribution) for Voc, Isc, Pmax, and internal resistance. b) Post-measurement imaging of the exact tested membranes (or a robust set) to exclude pinholes/tears and to verify uniform coverage. c) Additional leakage controls (intentional defect control, thickness series, or tracer permeability test) to show that transport is genuinely through the designed nanochannels.

Response: We appreciate the reviewer's insightful comments. Indeed, for ultrathin membranes supported on apertures, practical factors such as imperfect sealing, edge leakage, and defects may potentially affect the measured current and power densities. In response, we have carried out a series of rigorous validations and clarified the measurement procedure in the revised manuscript and Supplementary Information, as follows:

1. **Performance statistics and reproducibility**.

We respectfully note that the performance of sV2DP reported in this work is not anomalously high, but rather remains within the same order of magnitude as that of state-of-the-art 2DFMs (*Nat. Nanotechnol.* **2022**, 17, 622–628; *J. Am. Chem. Soc.* **2023**, 145, 5261–5269; *Nat. Sustain.* **2025**, 8, 446–455). These literature values (typically, > 100 W m^{-2}) have been included in the comparative tables in the Supplementary Information (Supplementary Table 3 and Table 6).

Although our performance is somewhat higher, it still falls within a physically reasonable range for well-defined 1D ion-transport channels with high surface charge density.

Following the reviewer's suggestion, we have evaluated the ion-selective performance of sV2DP across 15 independent measurements on different devices. Figs. R8-9 show the statistical distributions of V_{oc}, I_{sc}, R_{in}, t-, and P_{max}. All metrics exhibit narrow distributions without significant outliers, indicating good device-to-device reproducibility. In addition, the device yield is high, with the majority of fabricated devices exhibiting consistent ion-selective behavior within a similar performance range. These results demonstrate that the reported performance is robust and representative, rather than from anomalous measurements.

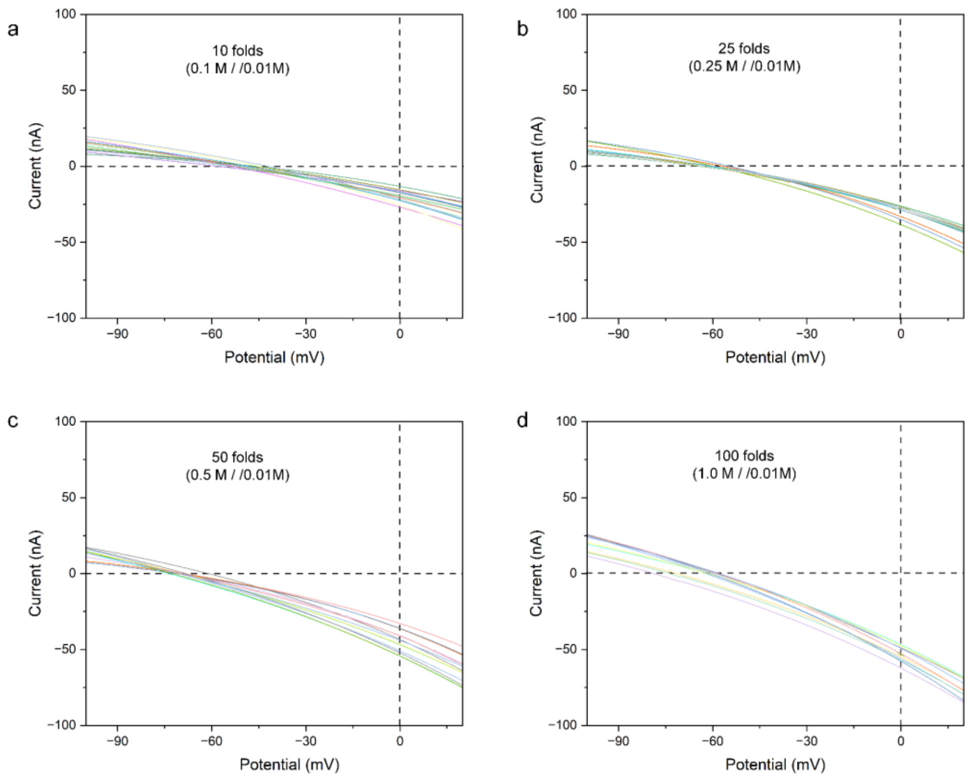

Fig. R8. *I*-*V* curves of sV2DP membranes recorded from 15 independent measurements under KCl concentration gradients of (a) 10-fold (0.1/0.01 M), (b) 25-fold (0.25/0.01 M), (c) 50-fold (0.5/0.01 M), and (d) 100-fold (1/0.01 M).

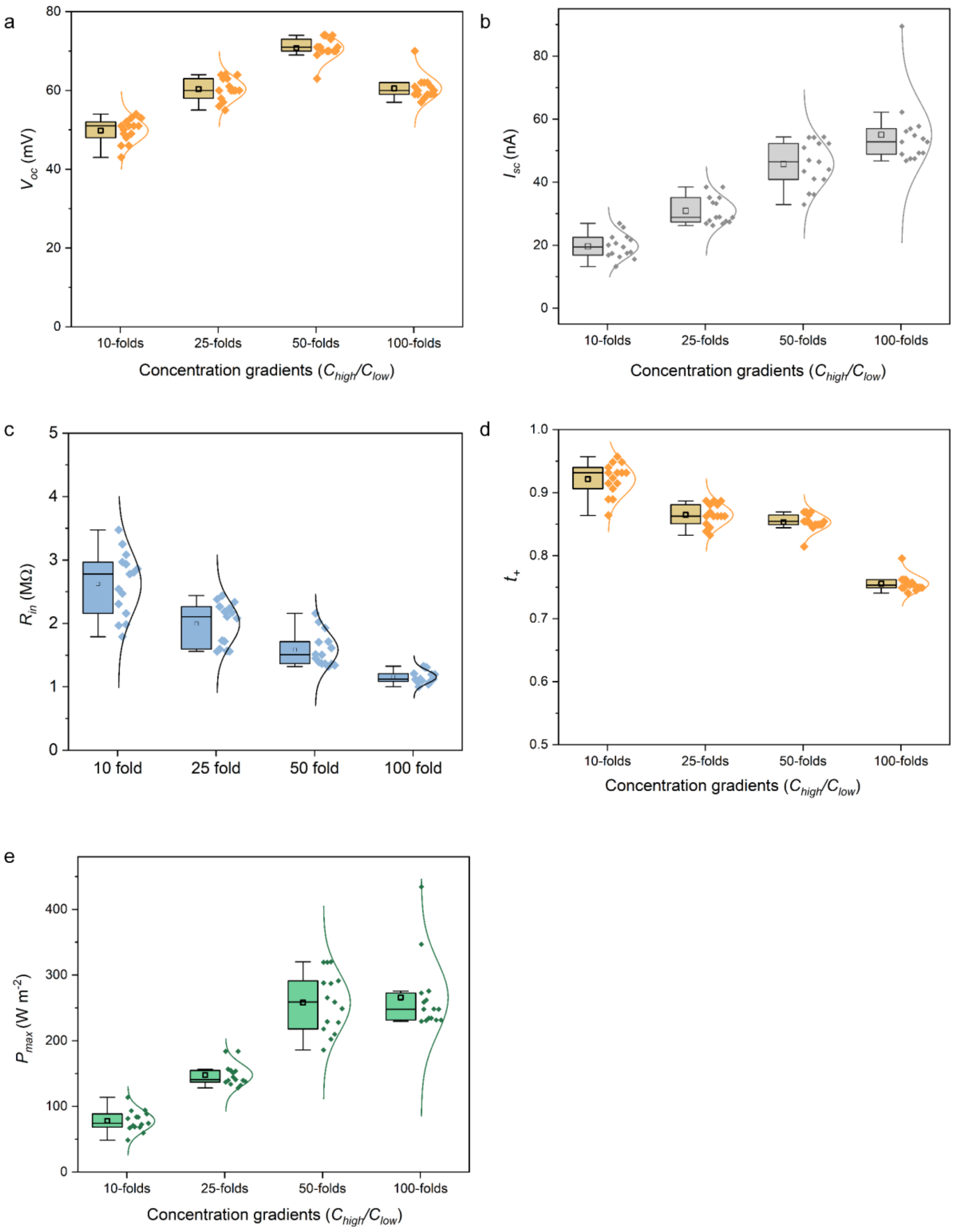

Fig. R9. Performance metrics extracted from 15 independent measurements under varied KCl gradients. (a) V_{oc}, (b) I_{sc}, (c) R_{in}, (d) t_- and (e) P_{max}, respectively.

2. Post-measurement integrity imaging.

We have provided post-measurement imaging of the tested membranes to directly assess structural integrity after ion-transport measurements. As shown in Fig. R10, the sV2DP

membrane exhibits continuous and uniform coverage across the SiN_x window before and after measurements, with no observable defects or tears. In addition, we note that if the measured ion transport were dominated by macroscopic edge-leakage pathways, ions would need to traverse lateral distances on the order of tens to hundreds of micrometres along the substrate. Such pathways would introduce a much higher ohmic resistance than transport through the 50 nm-thick membrane nanochannels, and therefore, edge-leakage contributions are negligible.

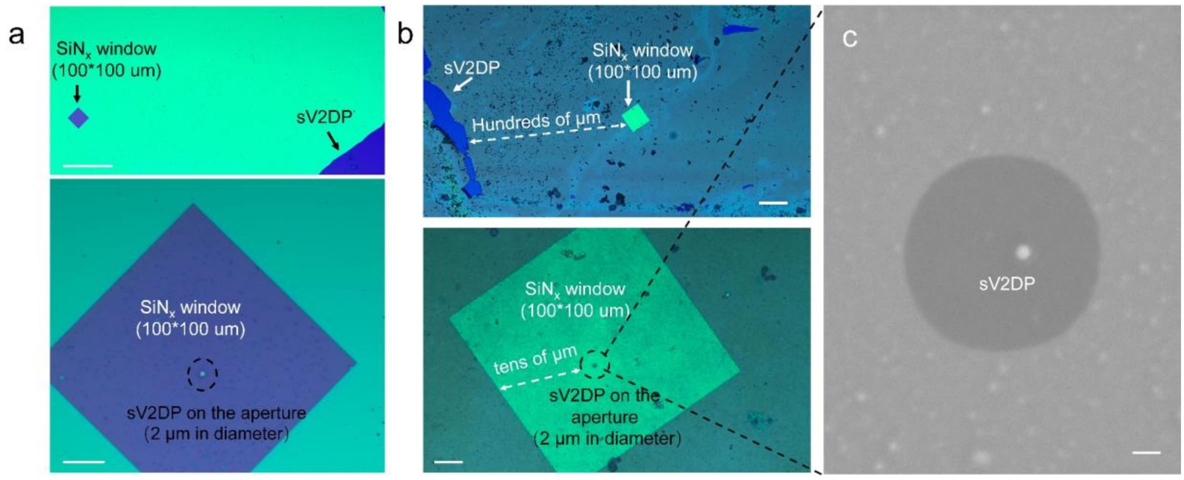

Fig. R10. Pre- and post-measurement imaging of the sV2DP devices. a, Optical images of the device before measurement. Scale bars: 100 μm (upper) and 10 μm (bottom). **b**, Optical images of the device after measurement. Scale bars: 100 μm (upper) and 10 μm (bottom). **c**, SEM image of the device after measurement. Scale bars: 200 nm.

3. Leakage-sensitive controls

To further assess the potential contribution of leakage pathways, we have implemented multiple control experiments:

1) Intentional-defect control

Microscopic defects (crystallinity control): Without using a surfactant during synthesis, we obtained amorphous sV2DP membranes with a higher defect density (Fig. R11a,b). Compared with the crystalline sV2DP membranes, these amorphous and highly defective membranes show a markedly reduced I_{sc} (from 45.874 to 19.18 nA), confirming that the observed high performance of sV2DP originates from the designed nanochannels rather than from defect-mediated transport pathways (Fig. R11 c-d).

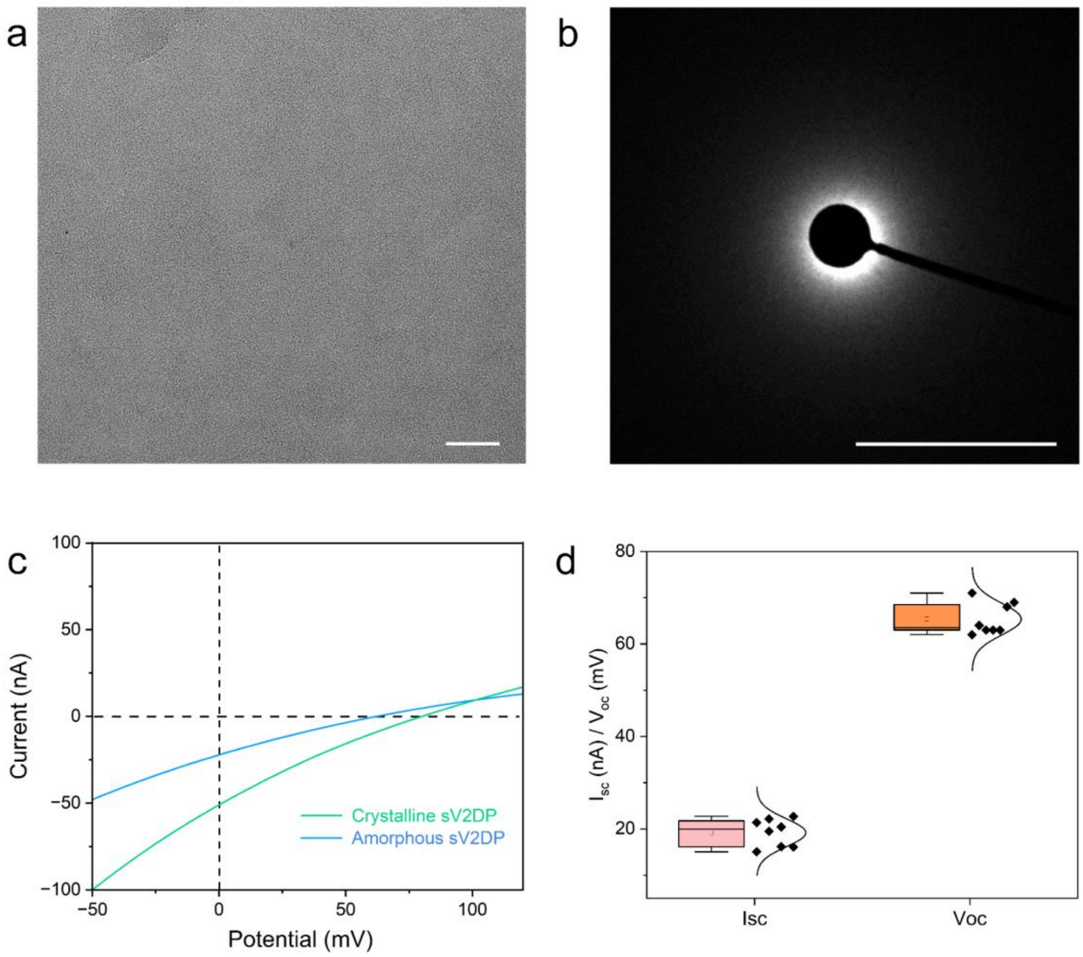

Fig. R11. Microscopic defects control for selective ion transport. a, HRTEM image and **b**, SAED pattern of the sV2DP membranes synthesized without using SOS. Scale bar: 20 nm in (a) and 1 1/nm in (b). **c**, *I-V* curves under the 0.5/0.01 M KCl gradients. **d**, I_{sc} and V_{oc} of the amorphous sV2DP membranes, under the 0.5/0.01 M KCl gradients, recorded from 8 independent measurements.

Macroscopic defects: To probe the impact of macroscopic 'defects', we introduced a nanoscale notch with a diameter of ~ 600 nm in the membrane using the focused ion beam (FIB) technology. This defect causes an enhanced conductance response but a lower V_{oc} (11 mV) than the pristine samples (V_{oc} = 76 mV), indicating a pronounced loss of anionic selectivity (S_{Cl^-}, 0.11 vs. 0.76). This result indicates that even a small macroscopic defect can effectively short-circuit the membrane and eliminate ionic selectivity (Fig. R12).

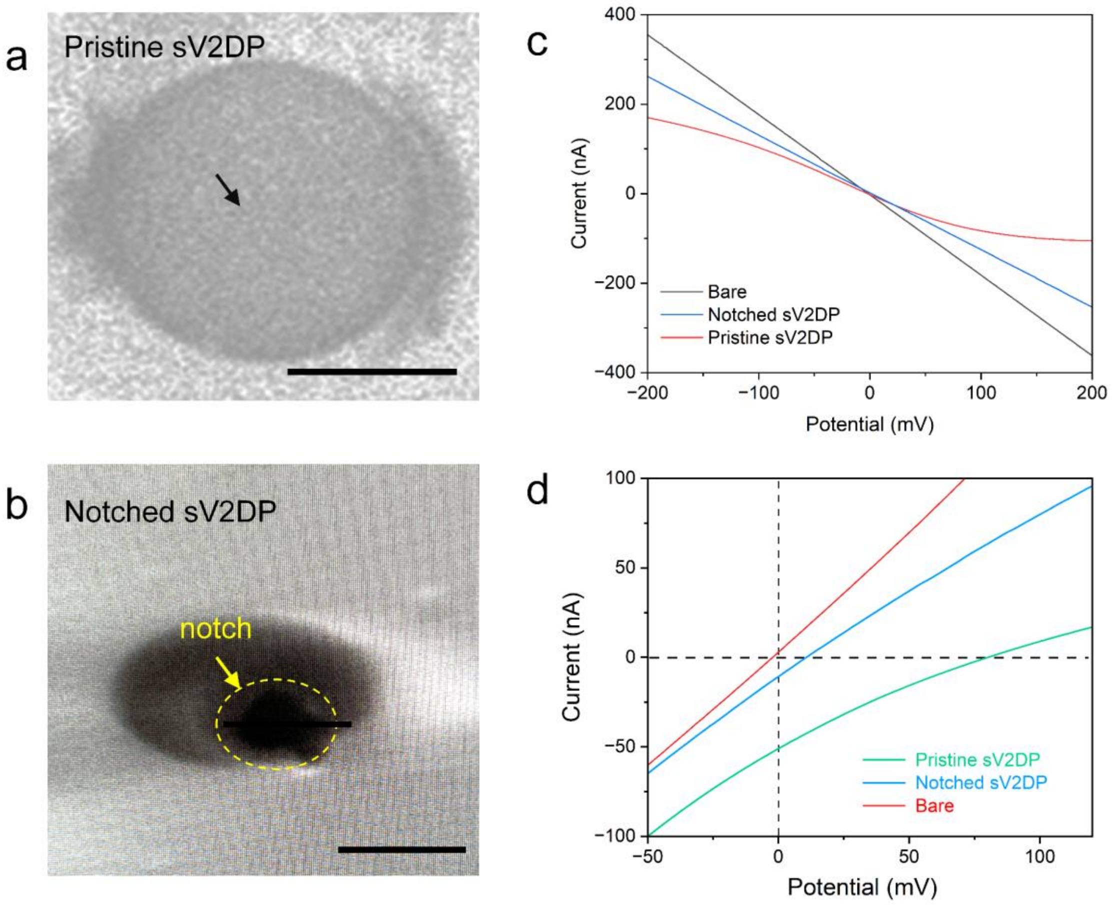

Fig. R12. Intentional-defect (notch) control to probe leakage-sensitive transport. a, **b**, SEM images of representative devices: pristine and notched sV2DP membrane samples. Scale bar, 1 μm. **c**, *I*-*V* curves of pristine and notched sV2DP membranes, and a bare aperture substrate in 0.1 M KCl solutions, showing that the conductance follows the order: bare substrate (1.79 μS) > notched sV2DP (1.25 μS) > pristine sV2DP (1.10 μS). **d**, Corresponding *I*-*V* under 0.5/0.01 M KCl. The notched sV2DP exhibits *I*-*V* behavior shifted toward the bare-substrate response, consistent with leakage-dominated transport and a loss of ionic selectivity.

2) Thickness-dependent validation

By systematically varying the reaction time (7-21 days), we synthesized sV2DP membranes with controlled thicknesses of 32-80 nm to evaluate thickness-dependent transport behavior. As shown in Fig. R13, measured V_{oc} values increase monotonically from 56.11 to 83.89 mV with increasing membrane thickness, while the measured I_{sc} values decrease correspondingly (from 40.51 to 20.58 nA) due to the extended ion-transport pathway. This inverse correlation between selectivity and conductance is characteristic of transport governed by well-defined nanochannels, where thicker membranes enhance selectivity by suppressing co-ion transport. In contrast, if leakage through defects or edge pathways dominated, the selectivity would be

largely insensitive to thickness, and the conductance would not exhibit a systematic dependence on membrane thickness.

Overall, these additional results and analyses provide strong evidence that the observed ion transport in sV2DP membranes occurs through the intrinsic nanochannels rather than via leakage pathways or macroscopic defects.

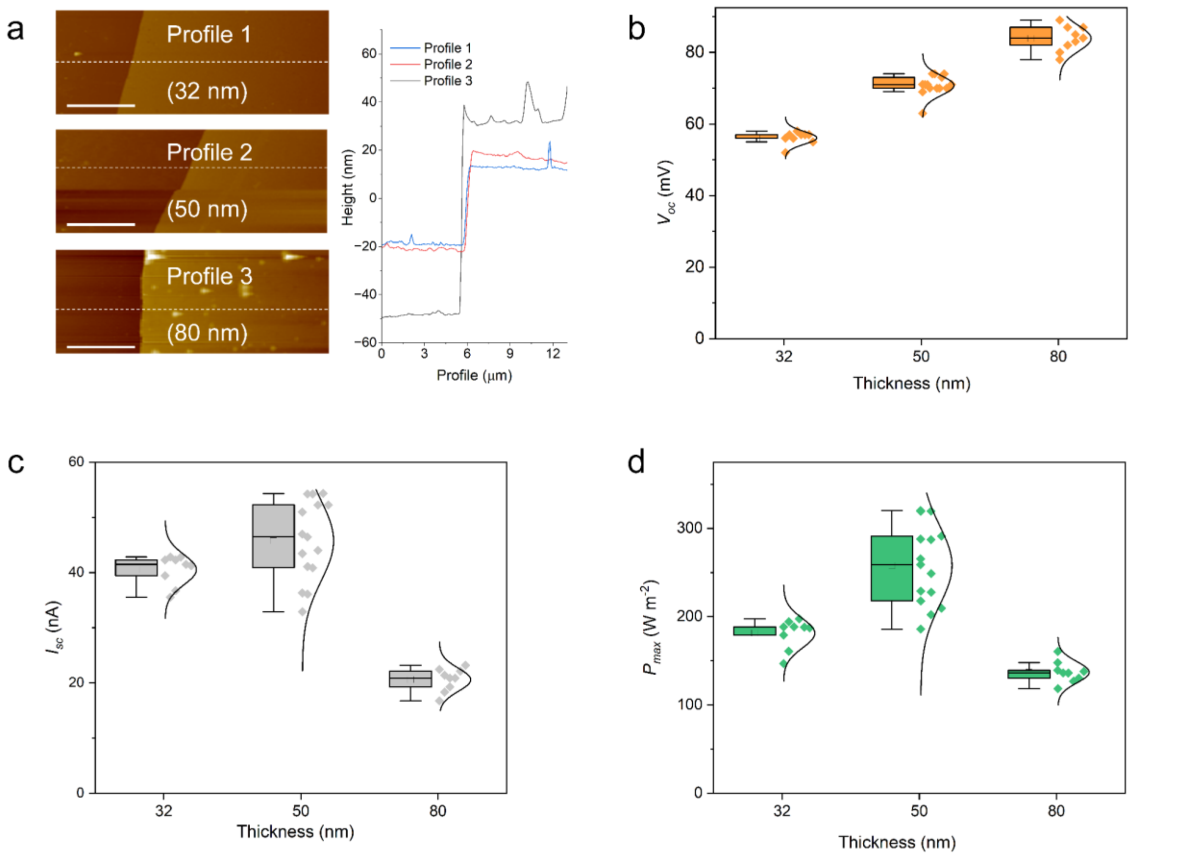

Fig. R13. Thickness-dependent performance of sV2DP membranes. a, Representative AFM images and corresponding height profiles used to determine membrane thicknesses of ~32, 50, and 80 nm. Scale bar: 3 μm. **b-d**, Thickness dependence of key osmotic-energy metrics, (b) V_{oc}, (c) I_{sc}, and (d) P_{max}, all evaluated under 0.5/0.01 M KCl gradients, and recorded from 9 independent measurements for 32- and 80-nm-thick membranes, and 15 independent measurements for 50-nm-thick membranes.

Actions:

a) We have added Fig. R8 as Supplementary Fig. 21 in the revised Supplementary Information on Page 30.

b) Fig. R9 is presented as Fig 2f – 2i in the revised manuscript on Page 9.

c) We have added Figs. R10, 12, and 13 as Supplementary Fig. 25, 23, and 24 in the revised Supplementary Information, respectively.

Comment 4. *Selectivity and transference number extraction may be confounded under strong gradients. Selectivity inferred from reversal potentials can be distorted by non-ideal solution behavior at high concentration, junction potentials, and concentration polarization near the membrane. These effects can bias transference numbers and selectivity estimates upward. What I need to see: a) Evidence that concentration polarization is controlled/quantified (e.g., stirring rate dependence, flow-through tests, or boundary layer analysis). b) Ideally, direct ionic flux measurements (ion chromatography/ICP) under operating conditions to validate transference numbers independent of voltage-only inference. c) Discussion or correction strategy for non-ideality when using large concentration ratios.*

Response: Thanks for the thoughtful comments. We fully agree that selectivity and transference numbers inferred from measured V_{oc} can, in principle, be influenced by non-ideal solution behavior and concentration polarization (CP) under large concentration gradients. In the revised manuscript, we have clarified the measurement protocol, provided additional analysis, and expanded the discussion to address these concerns:

1. CP control and quantification

To minimize bias-induced CP, V_{oc} is measured strictly under open-circuit conditions (I = 0) and before any *I*-*V* sweeps, thereby avoiding current-induced enrichment or depletion effects that become prominent at higher applied biases. As shown in Fig. R14, the representative V_{oc}-time (t) traces show that the V_{oc} reaches a stable plateau and remains constant over 300 s, indicating negligible drift due to CP. Furthermore, the effective transport area is defined by a 2-μm aperture (A = 3.14 μm^2), resulting in an extremely low absolute ion flux under open-circuit conditions. This significantly suppresses the development of concentration boundary layers compared to macroscopic membrane systems. While some gradient-induced interfacial polarization may still persist in quiescent reservoirs, such effects would reduce the effective activity gradient at the membrane interface, thereby lowering V_{oc}. Thus, any residual CP would again lead to underestimation, rather than overestimation, of selectivity.

Indeed, the long-term, practical ion-selective output depends on the time-evolving effective ion activities at the membrane interfaces, which are coupled to device scale and CP control. Mitigating these effects typically requires engineering solutions, such as controlled

flow-through operation, periodic electrolyte refresh/circulation, and optimized stirring/rotation. In this work, our focus is on the fundamental ion-transport behavior of the sV2DP membrane. A systematic investigation of these device-level factors and their impact on long-term performance will be addressed in a follow-up study. In addition, we used the Ag/AgCl electrodes with 3 M KCl salt bridges in this work to minimize liquid-junction contributions. Consistent with this, the blank SiN_x substrate exhibits negligible V_{oc} (~ 2 mV) under the same gradient, confirming that electrode/junction offsets are small (Fig. R5).

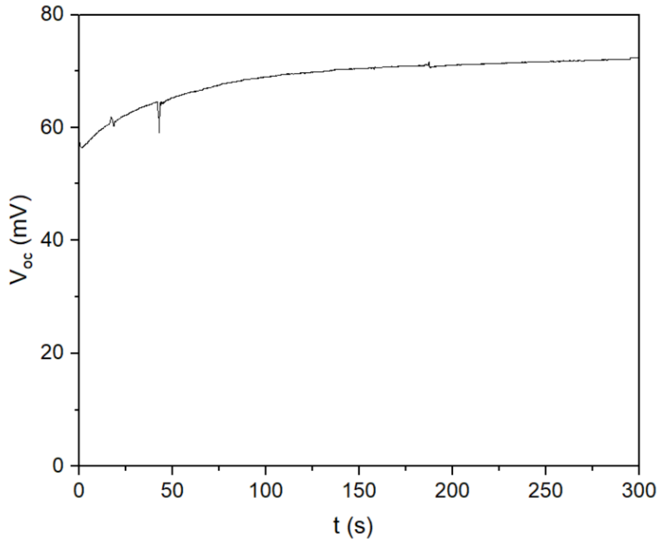

Fig. R14. V_{oc} recorded as a function of time after establishing the concentration gradient, showing stabilization to a steady plateau.

2. Direct ionic flux measurements

We thank the reviewer for this suggestion. For osmotic energy conversion, direct ionic flux measurements are not the most appropriate metric for evaluating membrane selectivity. The reason is that the primary function of an osmotic membrane in our system is not to achieve ion separation itself, but to generate a transmembrane electrochemical potential and, ultimately, electrical output from the salinity gradient. Therefore, the most functionally relevant measure of selectivity should be derived from the membrane potential (or reversal potential / osmotic voltage), because this parameter directly reflects the effective permselectivity that governs voltage generation. In contrast, direct ionic flux measurements primarily quantify the absolute transport rates of individual ions, which are better suited to separation-oriented membrane studies than to evaluating osmotic power generation. Since the target output of our device is voltage rather than ion separation, voltage-based selectivity provides a more meaningful assessment of membrane performance in the context of osmotic energy conversion.

3. Non-idealities of solutions (activity vs. concentration).

We would like to highlight that reversal-potential-based permselectivity is a widely adopted, community-standard method in nanofluidic membranes studies, where concentration ratios (c_{high}/c_{low}) are used as practical approximations to activity ratios (a_{high}/a_{low}) (*Nat. Rev. Mater.* **2019**, 4, 588-605; *Nat. Rev. Mater.*, **2021**, 622-639). Notably, when activities are explicitly considered ($a = \gamma C$), the term $\ln(a_{high}/a_{low})$ is generally smaller than $\ln(c_{high}/c_{low})$ at higher concentrations. Therefore, the concentration-based selectivity values reported represent conservative estimates, rather than overestimation. This clarification has been added to the revised manuscript.

Actions:

a) We have added the protocol for calculating the ion selectivity coefficient in the revised manuscript on Page 20.

'*The ion selectivity coefficient (S) was calculated using the following equation:*

$$S = \frac{V_{oc} F}{RT \ln \frac{a_{high}}{a_{low}}}$$

where a_{high} and a_{low} are the activities on the concentrated and diluted sides, respectively ($a = \gamma C$). For a conservative estimate and to facilitate direct comparison with prior reports, the concentration ratio (C_{high}/C_{low}) is used as an approximation of a_{high}/a_{low} in this work.'

b) We have the added discussion regarding the junction potential in the revised manuscript on Page 19.

'*Standard Ag/AgCl electrodes with 3 M KCl salt bridges (CHI111) were used to record voltage and current signals, effectively eliminating junction potential caused by salt concentration differences. The electrode spacing was approximately 25 mm. KCl solutions at pH 6.7 were chosen as electrolytes due to the similar bulk diffusion coefficients of K^+ and Cl^- ions. All measurements were conducted at room temperature (25 °C), and I-V scans were recorded at a scan rate of 10 mV s^{-1}. Prior to each I-V measurement, the V_{oc} was monitored until a stable value was reached.*'

c) We have presented Fig. R14 as Supplementary Fig. 20 in the revised Supplementary Information on Page 29.

Comment 5. Scale-up gap: micro-aperture vs macroscopic array performance needs *quantitative diagnosis. The drop from micro-aperture performance to the macroscopic aperture-array level is not surprising, but the manuscript's explanation (defects/grain boundaries) remains qualitative.*

What I need to see: quantitative mapping of where resistance arises in the array device (membrane intrinsic vs aperture edge vs sealing vs support), and characterization of defect density/continuity over large areas. A scaling plot of performance vs active area with an error model would help.

Response: We thank the reviewer for raising this important point. In the revised manuscript, we have elucidated the performance decline and quantitatively assessed this degradation through resistance analysis, scaling measurements, microscopic structural characterization, and device-level intentional defect-control experiments:

1. Evaluation of system resistance in single-pore and array devices

The non-linear decrease in the total resistance with increasing testing area (*A*) leads to reduced device performance, consistent with previous reports (*Nat. Nanotechnol.* **2022**,17, 622-628). In general, R_{total} (or R_{in} in *Response to Comment 3*) can be expressed as an equivalent circuit consisting of the reservoir resistance (R_{res}), the reservoir/membrane interface resistance (R_{inter}) and the membrane resistance (R_{mem}) (inset of Fig. R15a). In single-pore devices (A = 3.14 μm^2), R_{mem} dominates R_{total} at the MΩ level, and the resulting performance is therefore mainly governed by the intrinsic properties of the tested membrane. With the array device (A = 6356.5 μm^2), R_{total} decreases substantially (from 1.58 MΩ to 2.8 kΩ) due to the reduction in R_{mem}, becoming comparable to the R_{res} and R_{inter} (Fig. R15a). Consequently, the contributions from the reservoirs and interfaces become increasingly significant. According to $P = V_{oc}^2/R_{total}$, the measured device performance therefore becomes less representative of the intrinsic membrane properties, accompanied by a decrease in P_{max} from 258 to 48 W m^{-2} (Fig. R15b). In this work, we focus mainly on the intrinsic performance of the sV2DP membranes, while system-level optimization will be explored in future studies.

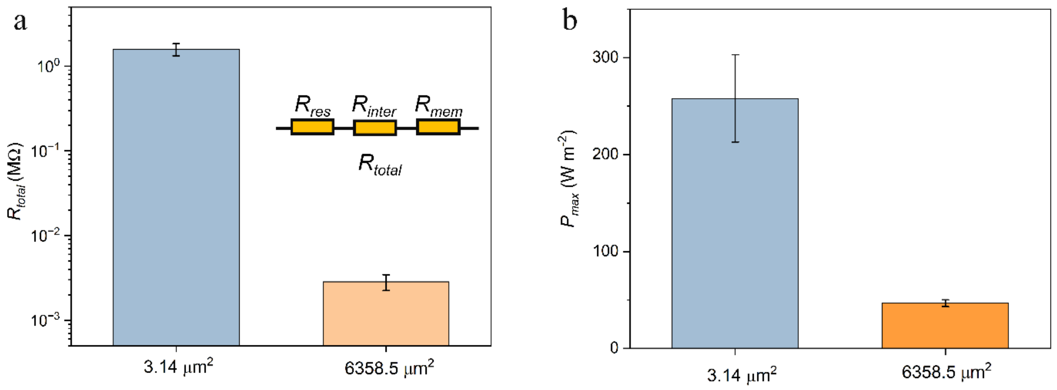

Fig. R15. Evaluation of system resistance in single-pore and array-pore devices. a, Measured R_{total} for sV2DP devices with single- (A, 3.14 μm^2) and array-pore (A, 6356.5 μm^2) substrates. Inset: Schematics of R_{total}, which contains R_{res}, R_{inter}, and R_{mem}. **b**, P_{max} values for the resulting systems under 0.5/0.01 M KCl gradients. Sample numbers: n = 15 (single pore devices) and n=3 (array devices).

2. Defect density and continuity over large areas.

To directly assess defect density, we have performed high-resolution transmission electron microscopy (HRTEM) imaging to visualize grain-boundary and defect regions in our sV2DP membrane. As shown in Fig. R16a, these non-ideal regions exist between crystalline domains and account for less than 5% of the total area. Compared to the intrinsic hexagonal lattice, these defects likely act as relatively open, less ion-selective nanoscale pathways (e.g., larger-pore-area 7-membered lattice motifs), providing a substantially larger effective cross-sectional area for ion transport (Fig. R16 b,c).

To gain more insight into the impact of defects on ion transport, we performed contrast measurements with different active areas on multiple samples. Importantly, the active areas investigated here were confined to the square-micrometre scale, thereby largely excluding the contribution of system-level resistance to the observed performance degradation. As the A increases from 0.76 μm^2 to 12.56 μm^2, the likelihood of including a higher fraction of non-ideal regions also increases. As a result, the measured V_{oc} values decrease from ~76 to ~61.8 mV, and the power density decreases from ~ 371 to ~135 W m^{-2}. This trend indicates that larger areas are more likely to include non-ideal regions, which dilute the average ion selectivity and output (Fig. R17).

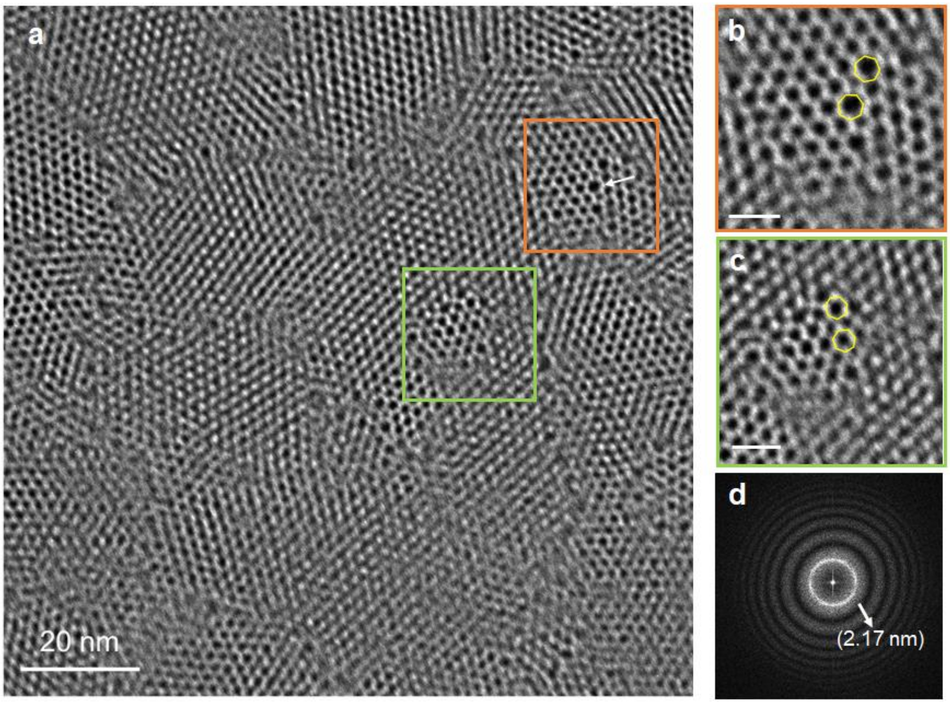

Fig. R16. Grain boundaries and defects in the sV2DP membrane. a, 300 kV Cs-corrected HRTEM image of the sV2DP membrane. The white arrow indicates an extended grain boundary. **b-c**, Regions highlighted by the orange and green boxes in (a) are magnified in (b) and (c), revealing larger-pore-area 7-membered rings (marked in yellow). **d**, Fast Fourier transform (FFT) of (a) showing a characteristic lattice spacing of 2.17 nm. Scale bars: 5 nm in (b) and (c).

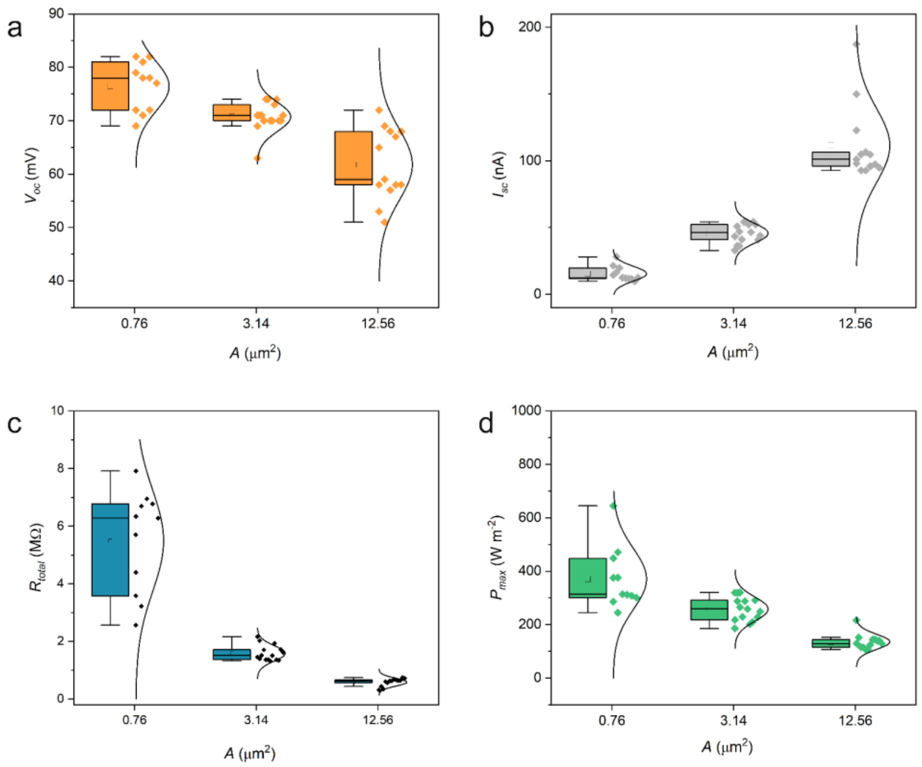

Fig. R17. Scaling of device performance with effective active area under a fixed KCl gradient (0.5 M/0.01 M). Dependence of key output metrics on the total active area (A), including V_{oc}, I_{sc}, R_{total}, and P_{max}, on the effective active area under a fixed KCl gradient (0.5 M/0.01 M). (a) V_{oc}, (b) I_{sc}, (c) R_{total} and (d) P_{max}. Sample numbers: n = 11 (0.76 μm^2), n = 15 (3.14 μm^2), n = 13 (12.56 μm^2).

To further evaluate the influence of macroscopic defects on the array devices, we deliberately punctured the membrane in one aperture by FIB etching. This artifact caused the measured ionic selectivity to drop from 55% to 15.7%, approaching the bare-substrate level and effectively short-circuiting the device (Fig. R18). This strong sensitivity to such defects indicates that performance degradation upon scaling is dominated by microscopic defects and grain boundaries, rather than by external device- or system-level factors such as sealing or substrate support.

Overall, the combination of HRTEM and SEM characterization, resistance analysis, and area-scaling performance provides a comprehensive and quantitative picture of defect density, membrane continuity, and their impact on device performance.

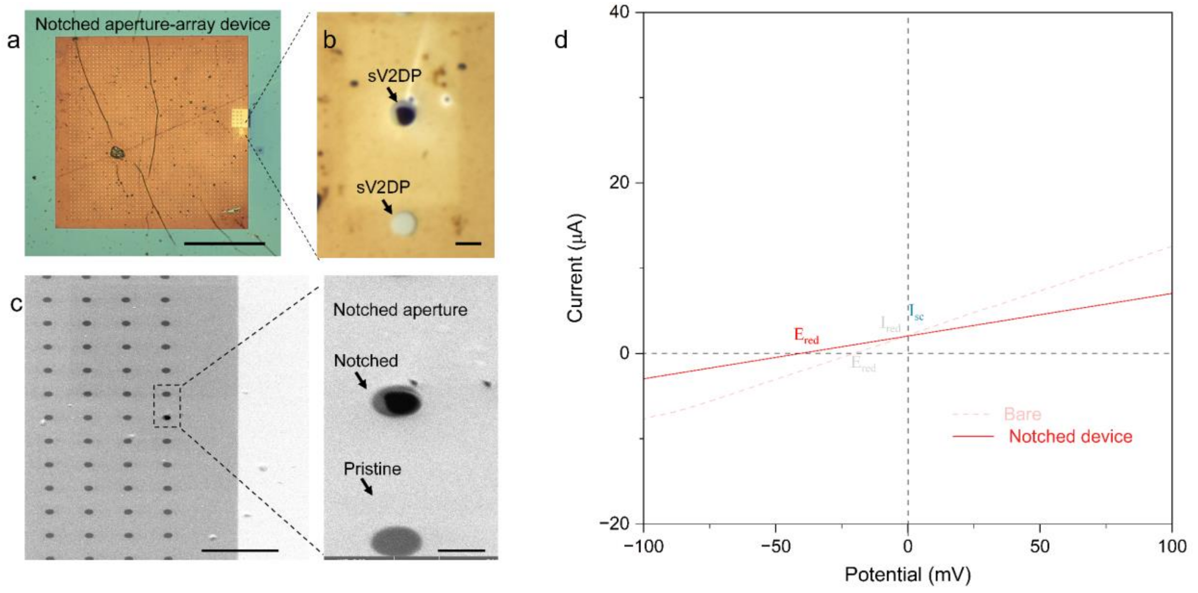

Fig. R18. Device-level defect-sensitivity test. a, Optical image of the notched aperture-array device. **b**, Magnified view of the FIB-treated region, showing an intentionally notched membrane and a neighboring pristine membrane. **c**, SEM image of the FIB-notched region. **d**, *I-V* curves of the notched device and the bare substrate, showing that introducing a local membrane defect strongly reduces ionic selectivity.

<u>Actions:</u>

a) Figs. R15-18 have been included as Supplementary Fig. 47-50, respectively, in the revised Supplementary Information on Pages 57-60.

b) We have added the above discussion in the revised manuscript on Pages 13-14.

'*When operated under a salinity gradient between artificial seawater and river water, the integrated system achieved an impressive P_{out} of 46.7 W m^{-2}, outperforming the thus-far reported macroscopic OPGs (Supplementary Fig. 46 and Table 6). However, a similar scale-up issue is observed for sV2DP membranes.[13,56] We attribute this behavior to the increasing impact of two factors: (i) the nonlinear decline in membrane resistance with increasing active area, and (ii) the presence of intrinsic grain boundaries and defects in larger-area membranes. As the membrane area increases, the decrease in intrinsic membrane resistance (R_{mem}) enhances the relative contributions of reservoir and interfacial resistances, leading to a progressive decline in P_{out} (Supplementary Fig. 47). In addition, grain boundaries and structural defects induce relatively open, less ion-selective transport pathways (e.g., larger-pore-area 7-membered lattice motifs) (Supplementary Fig. 48), which dilute the overall*

permselectivity. Consequently, the device performance shows an approximately exponential decay with increasing active area of single-aperture measurements (Supplementary Fig. 49). This conclusion is further supported by our device-level defect-sensitivity test: introducing an intentional single-pore defect causes a dramatic loss of ion selectivity (short-circuit-like behavior) (Supplementary Fig. 50). Despite this scale-up challenge, sV2DP-based devices demonstrate excellent operational stability. Under continuous operation in realistic conditions, stable output is maintained over nine days without noticeable degradation (Supplementary Fig. 51). Post-measurement structural, chemical, and device-level characterizations reveal no discernible changes compared to the pre-measurement state (Supplementary Fig. 52-54), confirming the robustness of the membrane. These results highlight the potential of sV2DP membranes as scalable, robust, and high-performance platforms for OPG.'

Comment 6. *Simulation–experiment linkage is currently too qualitative The "screw-like" anion pathway and spiral charge arrangement are visually intriguing, but the manuscript does not yet demonstrate that this mechanism quantitatively explains the measured selectivity/power enhancements.*

What I need to see: simulation outputs that connect directly to measurable quantities (conductance vs concentration, transference number, ion distributions comparable to extracted parameters) and robustness checks (multiple trajectories/initializations, salt types, concentrations, and sensitivity to chosen force field/charges).

Response: Thanks for the constructive suggestions. In fact, the MD simulations in this work are intended to provide a mechanistic, molecular-level perspective on how the channel structure and ion-framework interactions govern ion transport, rather than to quantitatively predict device-level metrics (e.g., conductance, power density, or transference numbers), which depend strongly on macroscopic factors such as membrane thickness, porosity, defects/grain boundaries, and measurement geometry. Predicting device-scale performance from MD would require additional assumptions about membrane microstructure and device configuration, which would introduce substantial uncertainty. We have revised the manuscript to clarify this scope, in line with common practice in the field (*Nat. Water* **2024**, 2, 706-718; *Nat. Water* **2026**, 4, 120-137)

1. Quantitative links to experimental observations

Within the scope of single-pore molecular simulations, we provide several quantitative results that support the experimental observations. First, the effective diffusion coefficients extracted

from the mean-square displacement reproduce the experimentally consistent selectivity ranking (Cl^- > Br^- > I^- in Supplementary Fig. 18). Moreover, the ions-framework interaction analysis (Fig. 2e, f) reveals distinct dynamics for each halide. Cl^- shows a broad distribution of interaction counts with pyridinium sites, indicating frequent binding–release events and higher mobility, whereas I^- maintains a nearly constant interaction count, suggesting stronger, more persistent interactions and thus reduced mobility. These quantitative interaction statistics provide a mechanistic basis for the observed selectivity differences.

2. Generality across ion species

To address the reviewer's concern regarding salt types, we have added additional trajectory visualizations for Br^- and I^- ions in sV2DP (Fig. R2). All three-halide species exhibit qualitatively similar 'screw-like' migration pathways along the c-axis, indicating that the surface-governed transport mechanism is general and not specific to Cl^-.

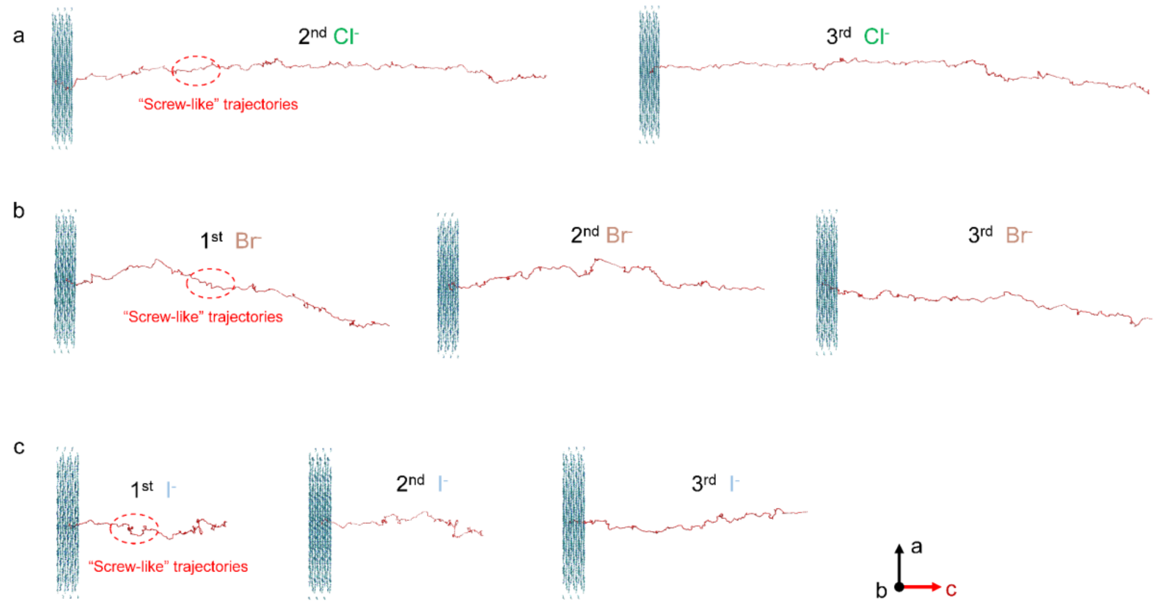

Fig. R2. Trajectory visualizations of halide-ion transport in sV2DP.Representative trajectories of (a) Cl^-, (b) Br^-, and (c) I^- obtained from three independent MD runs in the sV2DP channel. The red traces show the projected ion transport paths, highlighting reproducible screw-like motion along the c-axis.

3. Force-field validation and physical fidelity

Regarding force-field sensitivity, we validated the parameter set used in our simulations, namely UFF4MOF for the framework, TIP3P for water, and Joung-Cheatham parameters for halide ions (*J. Phys. Chem. B* **2008**, 112, 9020-9041). The Joung-Cheatham ion parameters were specifically optimized to reproduce key thermodynamic and structural properties (e.g.,

hydration free energies and crystal lattice properties) in combination with TIP3P water. As an internal consistency check relevant to ion hydration structure, we computed radial distribution functions *g(r)* for each halide ion in bulk aqueous solution and extracted the corresponding first-shell coordination numbers (Fig. R6). The resulting coordination numbers are in good agreement with reported experimental values (Supplementary Table 4), confirming that the ion-water hydration environment is described reasonably within our simulation framework.

The computed values fall within the experimentally reported ranges for all three ions, and the expected trend of increasing ion-water distance from Cl^- to Br^- to I^- is correctly reproduced. This indicates that the force field captures the hydration structure of halide ions with sufficient fidelity, ensuring that the relative differences in ions-framework and ions-water interactions observed in our simulations are physically meaningful. Moreover, because the same force field is applied consistently across all ion types, any systematic offset is expected to largely cancel when comparing relative selectivity trends.

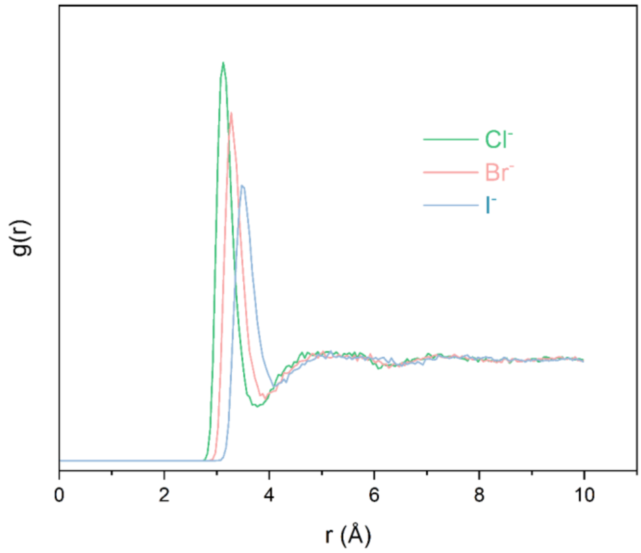

Fig. R6. Radial distribution functions (*g(r)*) between halide ions (Cl^-, Br^-, I^-) and water oxygen atoms in aqueous solution computed using TIP3P water and Joung-Cheatham ion parameters. The first-peak positions (in Å) increase from Cl^- to Br^- to I^-, consistent with the expected trend in hydration structure.

Supplementary Table 4| Hydration-shell metrics extracted from *g(r)*

Ion	First peak (Å)	Coordination number	Reported experimental range	Ref.
Cl^-	3.1	7.0	4.0-8.9	[55,56]

Br^-	3.3	6.9	6.0-7.4	[57]
I^-	3.5	7.6	4.2-10.3	[55,56]

[55] Marcus, Y. Effect of Ions on the Structure of Water: Structure Making and Breaking. *Chem. Rev.* **2009**, 109, 1346–1370.

[56] Migliorati, V.; D'Angelo, P.; Sessa, F. Going beyond Radial Hydration Models: The Hidden Structures of Chloride and Iodide Aqua Ions Revealed by the Use of Lone Pairs. *J. Phys. Chem. B* **2023**, 127, 10843–10850.

[57] D'Angelo, P. et al. Hydration Properties of the Bromide Aqua Ion: the Interplay of First Principle and Classical Molecular Dynamics, and X-ray Absorption Spectroscopy. *Inorg. Chem.* **2010**, 49, 4224–4231.

4. Statistical robustness

To assess statistical reliability, we performed multiple independent simulations with different random initial velocity seeds for each anion type (Cl^-, Br^-, and I^-). Trajectory visualizations from replicate runs confirm that the 'screw-like' anion migration pathway is reproducible across independent simulations, demonstrating that the observed behavior is robust and not an artefact of a particular initial configuration (Fig. R2).

<u>Actions:</u>

a) Fig. R2 is presented as Supplementary Fig. 33 in the revised Supplementary Information on Page 43.

b) We have included Fig. R6 as Supplementary Fig. 32 in the revised Supplementary Information on Page 42.

c) We have added the above discussion in the revised Supplementary Information on Page 41.

'*TIP3P model for water and Joung–Cheatham parameters for halide ions were used.*[28] *The Joung-Cheatham ion parameters were specifically optimized to reproduce key thermodynamic and structural properties (e.g., hydration free energies and crystal lattice properties) in combination with TIP3P water. As an internal consistency check relevant to ion hydration structure, we computed radial distribution functions g(r) for each halide ion in bulk aqueous*

solution and extracted the corresponding first-shell coordination numbers (Supplementary Fig. 32). The resulting coordination numbers are in good agreement with reported experimental values (Supplementary Table 4), supporting that the ion–water hydration environment is described reasonably within our simulation framework.'

Comment 7. *Mechanical integrity and long-term operation. The manuscript claims multi-day stability in output. For ultrathin (~50 nm) freestanding/partly supported films, mechanical integrity and chemical stability under gradients are critical.*

What I need to see: clearer evidence that stability reflects membrane robustness rather than stabilization of external factors (electrodes, reservoir chemistry, polarization). Include pre-/post-operation structural and chemical characterization, and specify whether any performance drift correlates with resistance changes.

Response: We agree that, for ultrathin (~50 nm) membranes, it is essential to distinguish intrinsic membrane robustness from potential stabilization of external factors (e.g., electrodes, reservoirs, or polarization effects). First, our defect-sensitivity tests at both the material level (see our response to *Comment 3*) and the device level (see our response to *Comment 5*) demonstrate that membrane integrity is critical for maintaining pronounced ion selectivity. Once structural defects are introduced, the ion-selective performance drops substantially, approaching short-circuit-like behavior. This confirms that the stable ion-selective outputs reported in this work originate from intact sV2DP membranes during operation.

Following the reviewer's suggestion, we further provide comprehensive post-operation structural and chemical characterizations to support the membrane's robustness. As shown in Fig. R19, TEM imaging and the corresponding SAED patterns after long-term operation preserve the hexagonal lattice and the characteristic (110) diffraction, indicating that the polycrystalline ABC-stacked structure and ultranarrow 1D channels remain intact. In addition, FTIR spectra collected after operation (Fig. R20) retain the key vibrational features (e.g., C=C, at 1,599 cm^{-1}), confirming that the chemical structure is maintained under operating conditions. Optical microscopy and SEM images (Fig. R21) further confirm that the membrane continuously covers the entire SiN_x array window after operation, with no observable tears or delamination, thereby excluding leakage or edge-dominated transport after operation. These characterizations provide clear evidence that the sV2DP membrane maintains both structural and chemical integrity during operation, supporting the long-term robustness of the ion-selective performance.

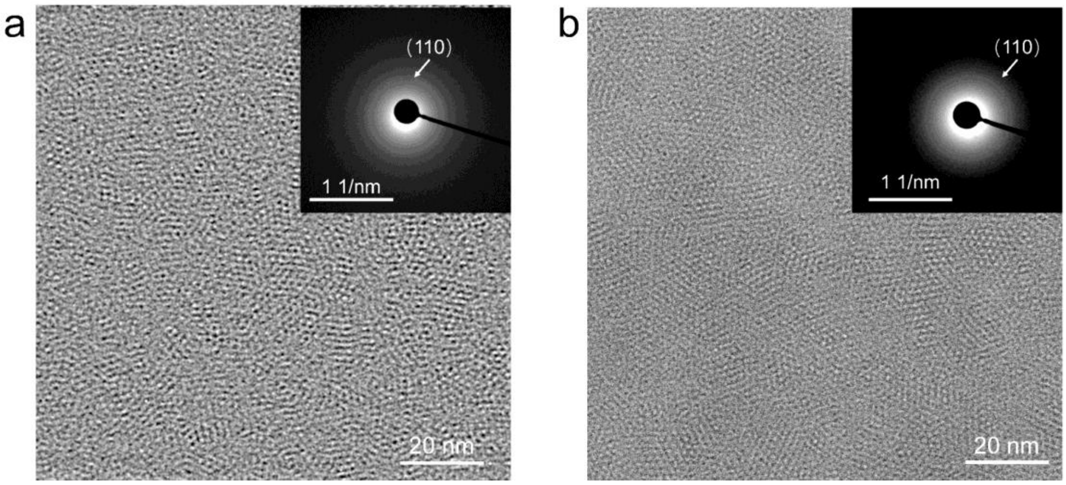

Fig. R19. HRTEM images and corresponding SAED patterns (**Inset**) of (a) pre-measurement and (b) post-measurement sV2DP membranes.

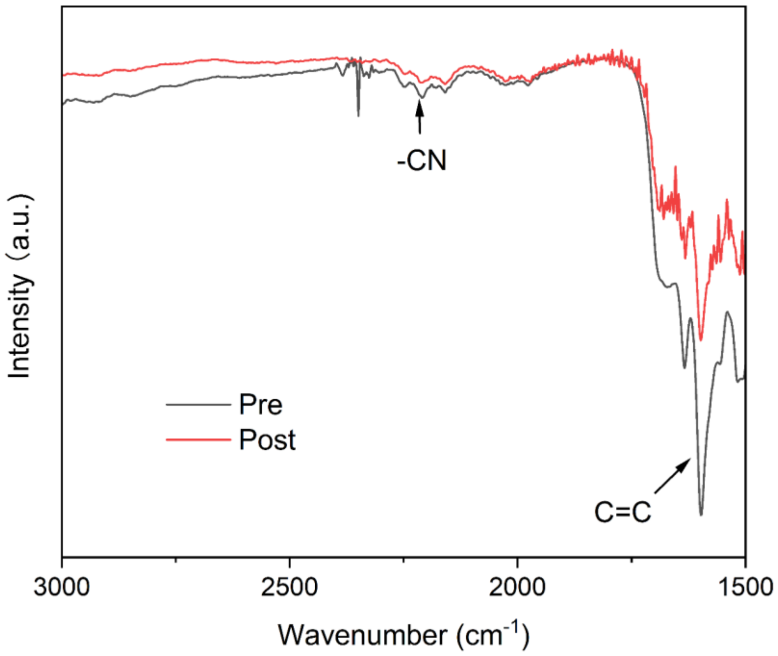

Fig. R20. FTIR spectra of pre-measurement (black) and post-measurement (red) sV2DP membranes.

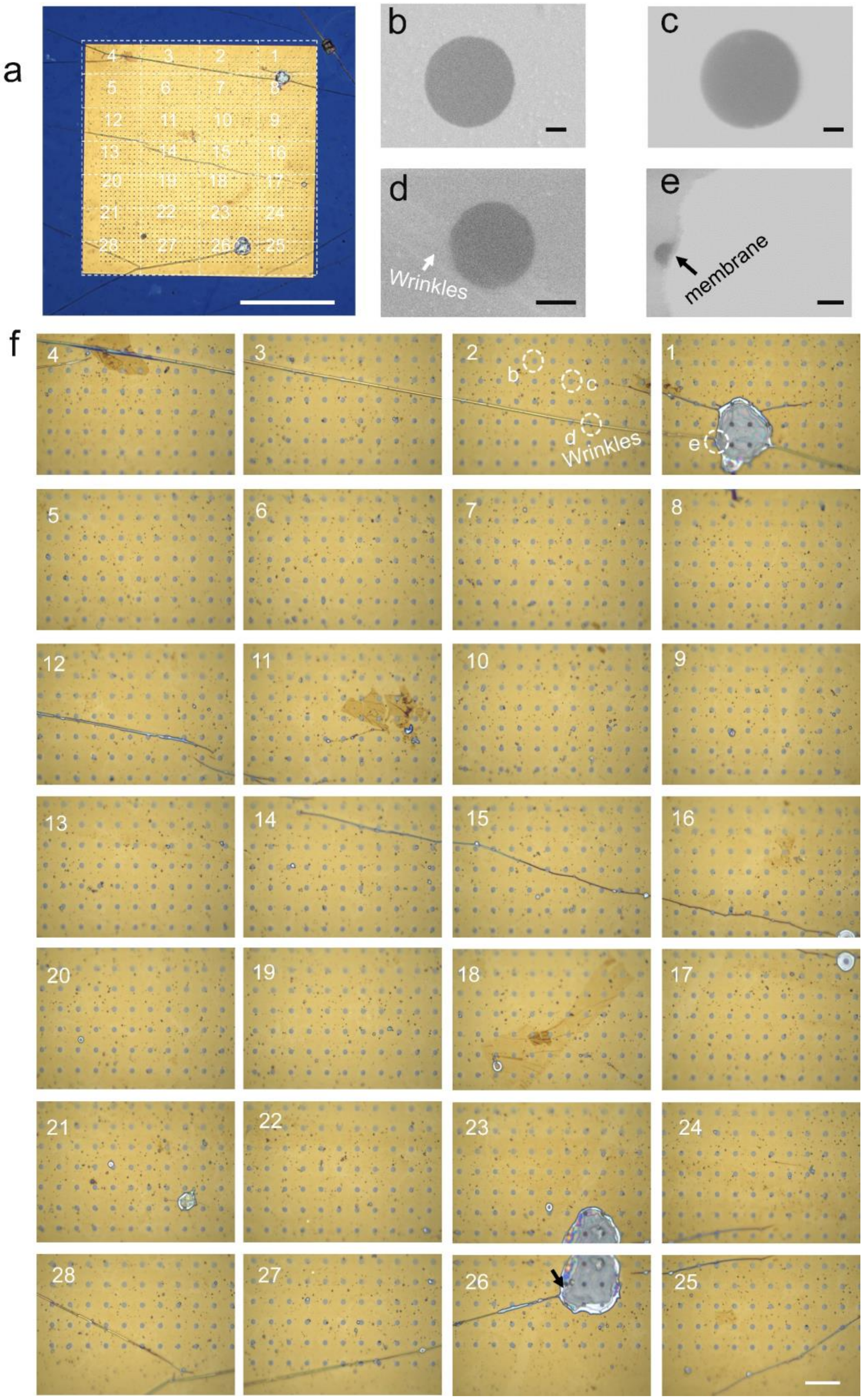

Fig. R21. Optical microscopy and SEM images of the large-area sV2DP device after operation. a, Optical image of the entire device. **b-e**, SEM images of a representative sV2DP membrane on the array substrate. **f**, Enlarged optical images showing the sV2DP membrane coverage in the marked regions in (a). Note that the macroscopic wrinkles observed in (d) do

not indicate damage to the membrane over the aperture. The scale bars are 500 nm in (b,c); 1 μm in (d); 2 μm in (e); and 20 μm in (f).

Actions:

a) Figs. R19-21 have been presented as Supplementary Figs. 52-54, respectively, in the revised Supplementary Information on Pages 62-64.

d) We have added the above discussion in the revised manuscript on Page 14.
'*We also performed post-measurement characterization, including structural and chemical analyses as well as device-level imaging, all of which show no discernible changes compared with the pre-measurement state (Supplementary Fig. 52-54), confirming the robustness of the membrane under operating conditions.*'

Comment 8. *Please add uncertainty/error bars and replicate numbers for all key extracted metrics (zeta potential, inferred charge density, selectivity/transference number, permeability/current density, and power density).*

Response: We thank the reviewer for this valuable suggestion. In the revised manuscript and Supplementary Information, we have added error bars and reported the number of independent replicates (n) for all extracted metrics. Specifically, we have added the replicate numbers in Fig. R4, Fig. R15, Supplementary Fig. 17, Supplementary Fig. 19, Supplementary Fig. 28, Supplementary Fig. 29, Supplementary Fig. 30, Supplementary Fig. 38, Supplementary Fig. 39, and Supplementary Fig. 40. In addition, we have performed statistical analysis for the key metrics (e.g., selectivity, V_{oc}, I_{sc}, P_{max}). The corresponding revisions have been incorporated into Figs. R9, R11, R13, R17, and R22, which are now presented as Figs. 2a and 2f-i in the revised manuscript, respectively.

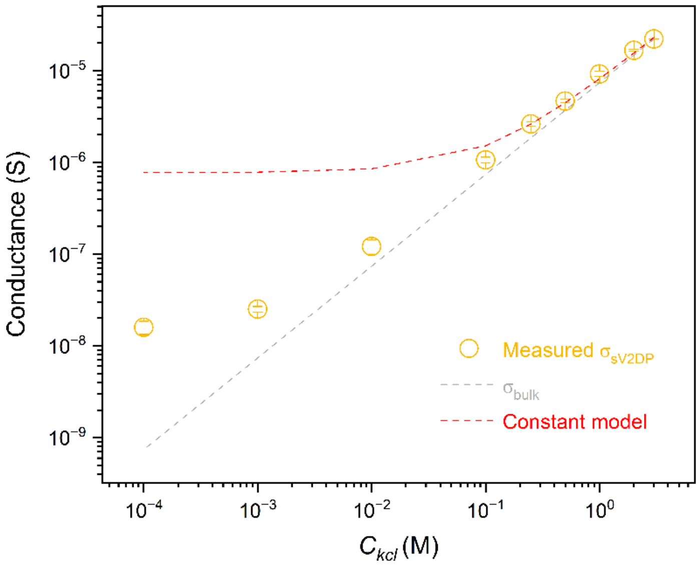

Fig. R4. Transmembrane conductance of the sV2DP membrane as a function of KCl concentration (C_{KCl}). The red curve shows the theoretical fit using a constant-charge model (Σ = 22.4 mC m^{-2}). Data are mean ± s.d, and were obtained from 6 independent measurements.

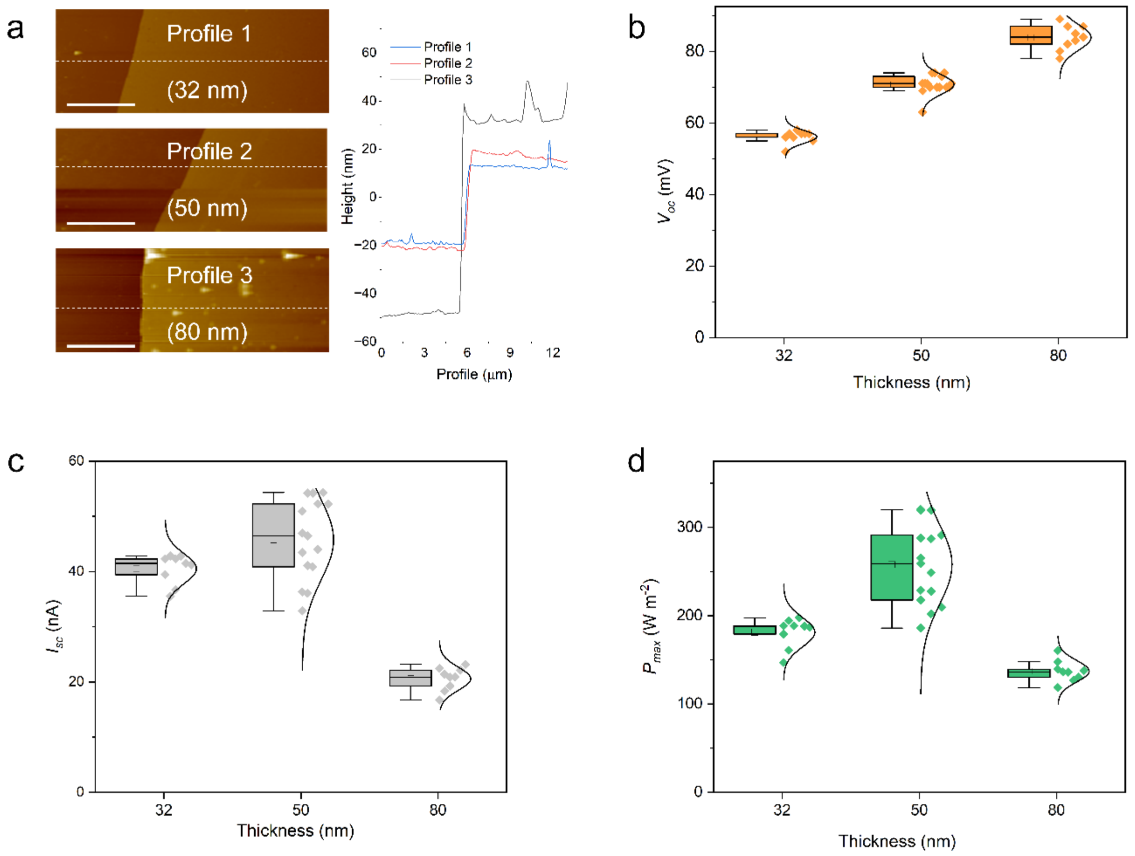

Fig. R13. Thickness-dependent performance of sV2DP membranes. a, Representative AFM images and corresponding height profiles used to determine membrane thicknesses of ~32, 50, and 80 nm. Scale bar: 3 μm. **b-d**, Thickness dependence of key osmotic-energy metrics, (b) V_{oc}, (c) I_{sc}, and (d) P_{max}, all evaluated under 0.5/0.01 M KCl gradients, and recorded from 9 independent measurements for 32- and 80-nm-thick membranes, and 15 independent measurements for 50-nm-thick membranes.

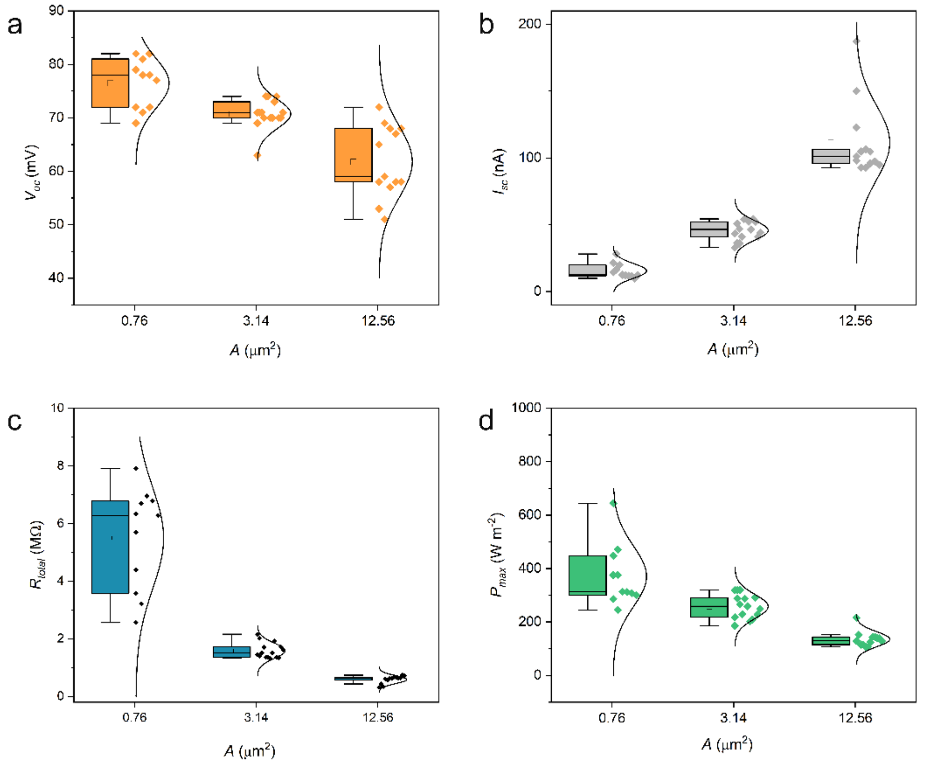

Fig. R17. Scaling of device performance with effective active area under a fixed KCl gradient (0.5 M/0.01 M). Dependence of key output metrics on the total active area (A), including V_{oc}, I_{sc}, R_{total}, and P_{max}, on the effective active area under a fixed KCl gradient (0.5 M/0.01 M). (a) V_{oc}, (b) I_{sc}, (c) R_{total} and (d) P_{max}. Sample numbers: n = 11 (0.76 μm^2), n = 15 (3.14 μm^2), n = 13 (12.56 μm^2).

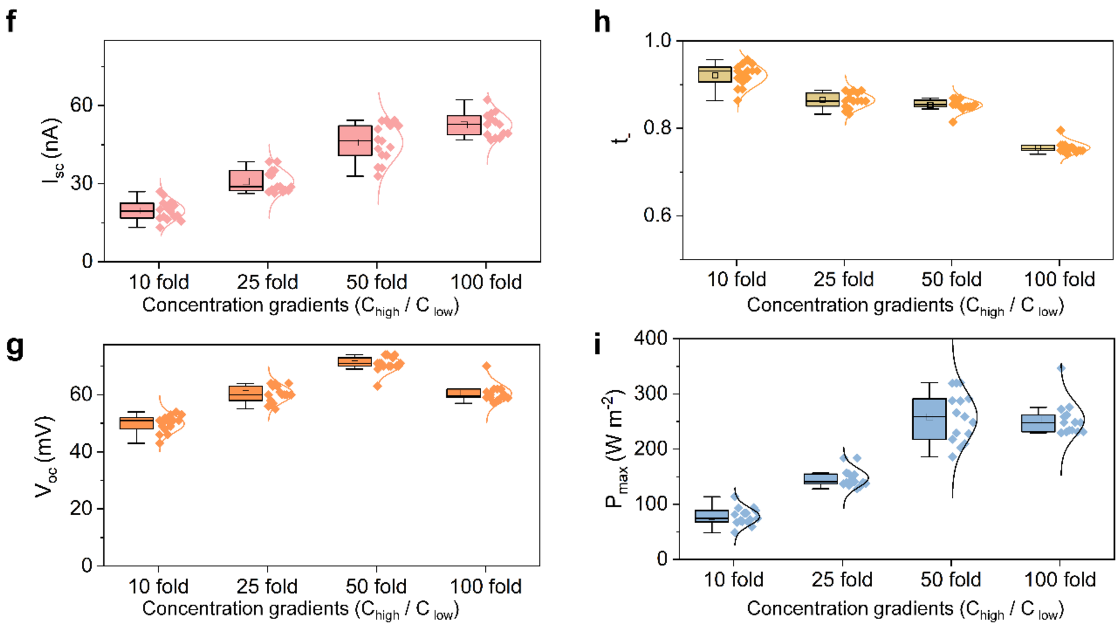

Fig. R22. Performance metrics extracted under varied KCl gradients. f, I_{sc}, **g**, V_{oc}, **h**, t_-, and **i**, P_{max}, respectively. Data were acquired from 15 independent measurements.

<u>**Actions:**</u>

a) Fig. R4 and R22 are presented as Fig. 2a and 2f-i in the revised manuscript on Page 9.

b) We have included replicates (n) in the revised Supplementary Information on Pages 28, 31, 33, 37, 38, 39, 48, 49, 50.

c) Fig. R13 and R17 have been added as Supplementary Fig. 24 and Fig. 49, in the revised Supplementary Information on Pages 33 and 59, respectively.

Comment 9. *Clearly define all symbols (including η_EDL) at first use, and provide a transparent derivation (or assumptions) with dimensional checks. Provide full measurement conditions for electrokinetic and osmotic tests (electrode type, electrode spacing, reservoir volumes, stirring/flow conditions, temperature control, and measurement duration per point).*

Response: Following the reviewer's suggestion, we have clearly defined all symbols at their first occurrence (e.g., η_{EDL}) in the revised manuscript. In addition, we now provide transparent derivations of the relevant equations (e.g., Supplementary Note 1), explicitly stating the underlying assumptions and including dimensional consistency checks to ensure clarity and

rigor. Furthermore, we have expanded the experimental section and Supplementary Information to include full measurement conditions for both electrokinetic and osmotic tests.

Actions:

a) We have defined the η_{EDL} at its first use, provided a detailed derivation, and stated the associated assumptions in the revised Supplementary Information on Page 62.

b) We have provided measurement details in the revised manuscript and Supplementary Information.

'***Supplementary Fig. 12***| *Setups for electrochemical measurements. a, Self-made setup with two reservoirs. The reservoir volume of each chamber is 8.0 ml.*' (Page 21 in the revised Supplementary Information)

'*Standard Ag/AgCl electrodes with 3 M KCl salt bridges (CHI111) were used to record voltage and current signals, effectively eliminating junction potential caused by salt concentration differences. The electrode spacing was approximately 25 mm. KCl solutions at pH 6.7 were chosen as electrolytes due to the similar bulk diffusion coefficients of K^+ and Cl^- ions. All measurements were conducted at room temperature (25 °C), and I-V scans were recorded at a scan rate of 10 mV s^{-1}. Prior to each I-V measurement, the V_{oc} was monitored until a stable value was reached (Supplementary Fig. 20).*' (Page 19 in the revised Supplementary Information)

Comment 10. *Clarify whether "channel size" refers to geometric pore size from structure, an "effective" electrostatic diameter, or an effective hydraulic diameter—and remain consistent throughout.*

Improve figure readability: ensure axes include units, specify concentration ratios explicitly (e.g., 50-fold = C_high/C_low), and avoid mixing normalized and absolute quantities without clear labeling.

Response: We have revised the manuscript and figures to clarify the definition of "channel size" and to improve figure readability and consistency.

1. Definition of channel sizes of the sV2DP membranes

In the revised manuscript, we have recalculated the channel size directly from the crystal structure and clarified the definition of *f(D)*. Specifically, *f(D)* is defined as the effective ion-

accessible cross-sectional area of the channel (units, nm^2), while D is an effective diameter (units, nm) derived from the same cross-section. For the ABC-stacked sV2DP membrane, the channel window can be approximated as an equilateral triangle with side length a = 2.36 nm (Fig. R1). To obtain a physically meaningful descriptor for ion transport, instead of using the triangular side length directly, we adopt two commonly used equivalent diameters (*Adv. Mater*. **2024**, 36, 2310791, *J. Phys. Chem. B* **2021**, 125, 40, 11256–11263):

1) Inscribed-circle (effective electrostatic) diameter:

$$D_{in}= \frac{\sqrt{3}}{3}a \approx 1.36 \text{ nm}$$

2) Hydraulic diameter:

$$D_h= \frac{4A}{P} = \frac{\sqrt{3}}{3}a \approx 1.36 \text{ nm}$$

From an equilateral triangle, D_{in} = D_h. We therefore adopt D = 1.36 nm as a conservative estimate of the ion-accessible permeation diameter. The corresponding effective channel area is then calculated as:

$$f(D)= \pi \left(\frac{D}{2}\right)^2 \approx 1.46 \text{ nm}^2$$

With this revised definition of D and $f(D)$, the apparent discrepancy with other reported 2DFM systems is resolved. Notably, when evaluated on this unified basis, the ABC-stacked sV2DP membrane exhibits more pronounced electrostatic advantages compared to other state-of-the-art membranes, further supporting the structural superiority of sV2DP in enabling selective ion transport (Supplementary Table 1).

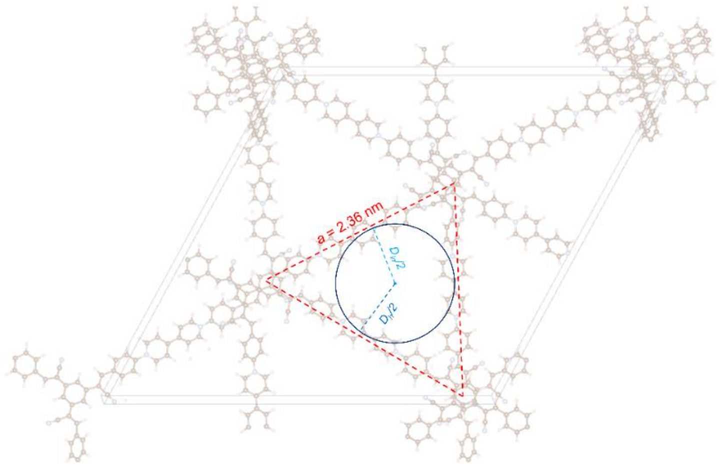

Fig. R1. Schematics of effective D (D_{in} and D_h) within the sV2DP units.

2. Figure readability

We have revised all figures to improve readability. For example, in Fig. 2f-i (i.e., Fig. R22), the horizontal axis is now labeled as the concentration gradient (C_{high}/C_{low}).

Actions:

a) We have redefined the channel sizes and added Fig. R1 as Supplementary Fig. 1, in the revised Supplementary Information on Page 8.

b) We have revised the figures (e.g., Fig. 2f-2i) in the revised manuscript.

Comment 11. *Correct typographical issues (e.g., spelling of "Debye length" and any inconsistent unit formatting such as m−2, kA m−2). If possible, include additional controls: pH dependence, multivalent ions, and concentration-dependent conductance to support the claimed mechanism.*

Response: Thanks for pointing out these issues. We have carefully revised the manuscript and corrected the typographical and formatting errors throughout. Regarding the requested additional controls, we have conducted pH-dependent measurements (pH: 5-9). Based on our previous studies (*Angew. Chem. Int. Ed.* **2024**, e202316299, *Angew. Chem. Int. Ed.* **2024**, e202409349), the positively charged nature of sV2DP is maintained over a wide pH range from 4 to 10 due to the permanently cationic viologen units. While the solution pH can influence counterion association with viologen sites and thus modulate charge screening and the apparent charge density, the membrane remains net positively charged. This is further supported by the pH-dependent ion-selective transport behavior. As shown in Fig. R7, both Cl^- selectivity (derived from V_{oc}) and net permeability (reflected by I_{sc}) show a slight decrease with increasing pH. Importantly, across the entire tested pH range, sV2DP consistently maintains a strong positive charge and retains anion-selective transport characteristics.

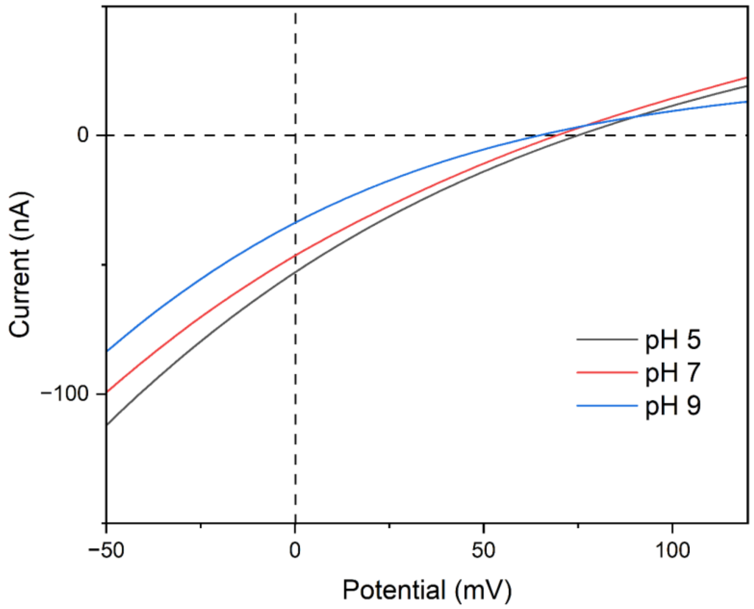

Fig. R7. pH-dependent ion-selective transport of the sV2DP membrane. Ion-selective measurements of the sV2DP membrane were performed at pH 5, 7, and 9 under the 0.5/0.01 M KCl gradients.

Following the reviewer's suggestion, we have also included multivalent-ion tests and concentration-dependent conductance measurements (see our response to *Comment 1*). Guided by our MD and experiment results indicating that transport is dominated by anion-framework interactions, the multivalent-ion experiments were designed by fixing the anion (Cl^-) while varying the cation valence and hydration size (K^+, Na^+, Ca^{2+}, and Mg^{2+}) (Fig. R23a). As shown in Fig. R23b and c, the Cl^- selectivity of the sV2DP membrane becomes more pronounced as the hydrated diameter of the cation increases. This result indicates that cation transport is increasingly suppressed when larger hydrated cations are involved, which can be attributed to the stronger steric hindrance and higher energetic cost for their passage through the confined sV2DP nanochannels. Because the transport of Cl^- is comparatively less hindered, the overall ion transport behavior shifts toward stronger anion selectivity. This size-dependent trend provides further support for our proposed anion-dominated transport mechanism in the sV2DP nanochannels.

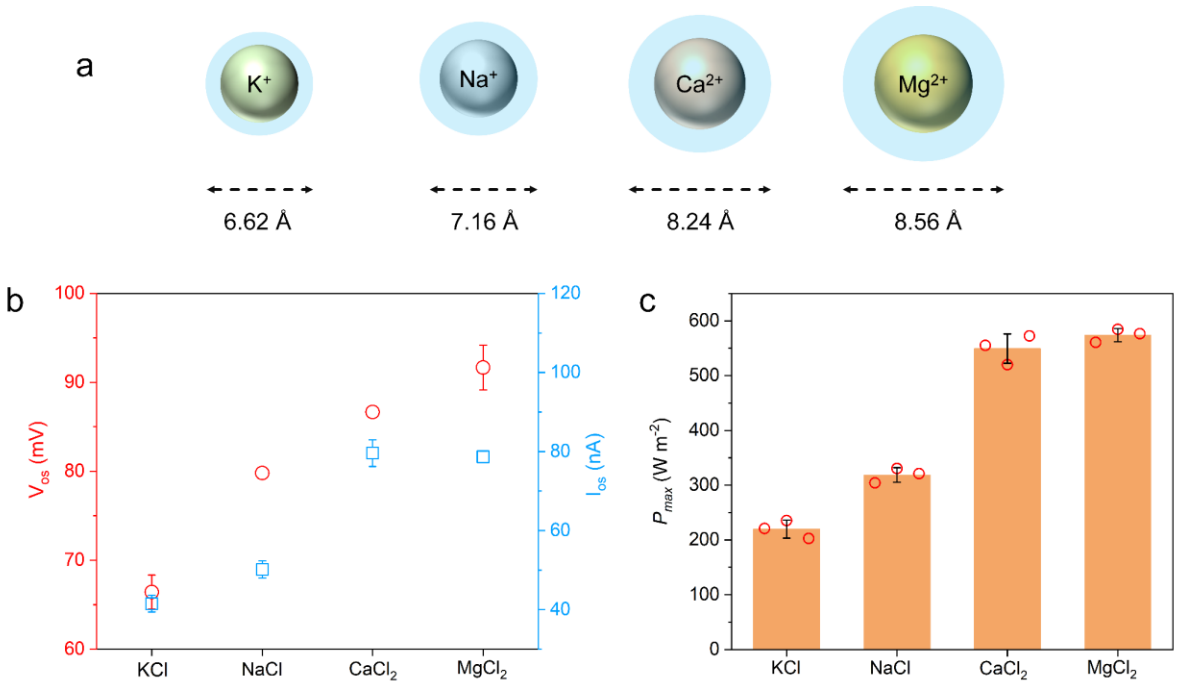

Fig. R23. Multivalent-cation effects on osmotic performance of sV2DP. a, Schematic comparison of hydrated diameters for common seawater cations (K^+, Na^+, Ca^{2+} and Mg^{2+}). **b**, Comparison of the V_{os} and I_{os} of the sV2DP membrane under various 50-fold electrolyte gradients (KCl, NaCl, $CaCl_2$, and $MgCl_2$). **c**, P_{max} values derived from (b). Data were obtained from 3 independent measurements in (b) and (c).

Actions:

a) We have corrected the typographical issues in the revised manuscript on Page 1 and Page 2.

b) Fig. R7 and R23 are presented as Supplementary Fig. 26 and Fig. 40 in the revised Supplementary Information, respectively.

Point-to-point response to the comments from the reviewers

Reviewer #1

General comment. *All of our comments have been solved and this work can be published now.*

Response: We sincerely thank the reviewer for the careful re-evaluation of our work and for the positive feedback. We are pleased that all concerns have been addressed and that the manuscript is now considered suitable for publication.

Reviewer #2

General comment. *The revised manuscript is substantially improved, and the authors have made a serious effort to address the reviewers' concerns. The work is interesting, the membrane synthesis and characterization are generally strong, and the revision has clarified several previously ambiguous points. However, a number of important issues remain only partially resolved, mainly related to the quantitative interpretation of surface charge density, selectivity analysis under strong gradients, the simulation-to-experiment link, and the framing of benchmarking claims. In my view, the manuscript is closer to publishable form, but these remaining points should still be corrected before acceptance.*

Response: We sincerely thank the reviewer for the thorough reassessment of our manuscript and for recognizing the improvements made in the revision. We fully acknowledge the remaining concerns and have carefully addressed each of these points in the revised manuscript. Point-by-point responses are provided below, and all corresponding revisions have been incorporated accordingly.

Comment 1: *Surface charge density analysis remains internally inconsistent.*

The revised manuscript improves the discussion of charge density by moving away from an overinterpretation of zeta potential and introducing a conductance-based constant-charge model. However, the manuscript still contains an internal inconsistency because the Results section attributes the charge density to a constant-charge conductance fit, while the Methods section continues to describe a Gouy–Chapman/zeta-potential-based estimate. This inconsistency affects a central mechanistic pillar of the paper and must be corrected so that the charge-density analysis is described in one consistent manner throughout.

Response: We thank the reviewer for pointing this inconsistency out and apologize for the lack of alignment in the previous version. In the revised manuscript, we have removed the zeta-

potential-based (Gouy-Chapman) estimation from the Methods section to avoid any ambiguity. The surface charge density is now defined and derived exclusively based on the conductance-based constant-charge model throughout the manuscript.

Actions:

a) We have deleted the zeta-potential-based (Gouy-Chapman) charge density estimation from the Methods section in the revised manuscript on Page 20.

Comment 2: *Charge density is still not independently validated.*

Although the revised conductance-based fitting is more appropriate than the earlier treatment, the effective fixed-charge density still rests on a model-dependent estimate without an orthogonal validation method. For such highly confined channels, this is a nontrivial limitation. The authors should either provide an independent cross-check of charge density or clearly soften the language and state that the reported value is an effective fitted parameter rather than a directly validated physical surface charge density.

Response: We thank the reviewer for this insightful comment. We fully agree that, in highly confined nanochannels, the extracted charge density from transport measurements is inherently model-dependent and does not constitute a direct, independently validated physical quantity. In response, we have redefined the reported charge density as an effective (or apparent) charge density, rather than an absolute physical surface charge density. All related statements have been carefully softened to avoid overinterpretation. In addition, we have explicitly clarified in the main text and Supplementary Note 3 that the reported values are obtained from a conductance-based fitting procedure. We emphasize that these values should be understood as an effectively fitted parameter that captures the ion-transport behavior of the system, rather than an independently validated physical surface charge density.

Actions:

a) We have revised the expression of the charge density value in the revised manuscript on Page 7.

'By fitting a constant charge model, an effective (or apparent) Σ value of sV2DP is further estimated to be + 22.4 mC m^{-2} (Supplementary Note 3), significantly higher than those of typical 2DFMs (< 10 mC m^{-2})[38-42]'

b) We have revised the corresponding discussion in the *Supplementary Information* on Page 68.

‘***Supplementary Note 3| Surface-charge-density analysis for ultranarrow sV2DP nanochannels***

The surface charge density of sV2DP is derived by a constant charge model rather than an independent physical measurement. To quantify the effective (or apparent) surface charge density (Σ) of sV2DP nanochannels, ...’

Comment 3: *The η_EDL metric is better framed, but still functions mainly as a heuristic descriptor.*

The revision appropriately repositions η_EDL as a heuristic, first-order design metric rather than a rigorous predictive theory. That is a positive change. However, the manuscript still uses η_EDL somewhat aggressively to rationalize performance improvements without demonstrating broader predictive capability across systems or conditions. The authors should keep the interpretation modest and consistently present η_EDL as a qualitative design descriptor rather than a validated universal metric.

Response: We fully agree that the interpretation of η_{EDL} should remain appropriately modest and not be overstated as a predictive or universal metric. Following the reviewer’s suggestion, we have further refined the manuscript to ensure that η_{EDL} is consistently presented as a qualitative design descriptor. Specifically, we clarify that η_{EDL} is most meaningful in well-defined nanochannel geometries, where it provides intuitive insight into the extent of electric double-layer (EDL) overlap and its potential influence on ion selectivity, but does not constitute a rigorous or generally predictive framework. Accordingly, we now emphasize that η_{EDL} is primarily useful for rationalizing relative differences between specific channel structures under comparable conditions, rather than serving as a general predictive parameter across different systems or operating regimes. In the revised manuscript, its use is therefore confined to a comparative analysis of the AA- and ABC-stacked channel architectures. The observed performance enhancement is interpreted in terms of the relative increase in η_{EDL} arising from structural modulation, rather than as evidence of a universal relationship. All related descriptions have been carefully revised to avoid overgeneralization.

Actions:

a) We have revised the above discussion in the revised manuscript on Page 1.

'*Compared with its non-staggered AA-stacked counterpart, sV2DP exhibits 3.2-fold enhancement in η_{EDL} under a 50-fold KCl gradient*, *combining high anionic selectivity* ...'

Comment 4: *The selectivity and transference-number analysis is still not fully closed.*

The authors have clarified how selectivity is extracted and have improved the explanation of activity versus concentration ratios, junction potential minimization, and voltage stabilization. However, the reported selectivity and transference values still rely entirely on reversal-potential inference under strong concentration gradients, without an independent validation such as direct ionic-flux measurements or a more explicit boundary-layer analysis. The approach is reasonable, but the claims should be tempered because the analysis remains indirect.

Response: We appreciate the reviewer's insightful comments. We acknowledge that the selectivity and transference-number analysis in this work is based on reversal-potential inference under concentration gradients and therefore represents an indirect approach. To address the reviewer's concern, we have clarified in the revised manuscript that the reported selectivity and transference numbers are derived from the measured reversal potentials (V_{oc}), and should be interpreted as inferred quantities rather than directly measured transport parameters. The corresponding descriptions have been carefully revised to avoid overinterpretation.

At the same time, we note that this methodology is widely adopted in osmotic energy conversion studies (*Nat. Nanotechnol.* **2022**, 17, 622-628; *Nat. Sustani.* **2025**, 8, 446-455; *Nat Energy* **2026**, 11, 470-478), particularly for highly confined nanochannels where direct ionic-flux measurements and detailed boundary-layer analyses remain experimentally challenging. The benchmarking in this work is therefore based on literature values obtained using comparable approaches, ensuring consistency and a fair basis for comparison across systems.

Actions:

a) We have updated the above discussion in the revised manuscript on Page 8.

'*Based on measured V_{oc} values, the sV2DP membrane maintains the Cl^- selectivity coefficients (S_{Cl^-}) above 50% across all tested C_s gradients, with an inferred t^- of up to 0.75 even under a 100-fold gradient* (*Fig. 2h and Supplementary Fig. 22*).'

Comment 5: *Concentration polarization is acknowledged but not rigorously quantified.*

The rebuttal explains why concentration polarization is expected to be limited under their testing geometry and open-circuit protocol, which is helpful. Still, the manuscript does not provide a quantitative assessment of boundary-layer effects, nor does it show stirring-rate or flow-dependent controls. Because strong gradients are central to the reported performance, the discussion should be more explicit that residual concentration polarization could influence the extracted transport parameters and remains a limitation of the current analysis.

Response: In the revised manuscript, we have explicitly clarified that residual concentration polarization may still exist and could partially influence the measured transport response under strong concentration gradients. We further acknowledge that this introduces a limitation in the current analysis, particularly in the extraction of transport-related parameters under non-equilibrium conditions. To improve transparency, we have added this discussion directly in the relevant section of the manuscript. In addition, we note that additional mitigation strategies, such as introducing structural asymmetry in the membrane or applying system-level optimization (e.g., stirring or thermal convection), while important, are beyond the scope of the present work and will be considered in future studies.

Actions:

a) We have included the following discussion in the revised manuscript on Page 17.

'*It should be noted that residual concentration polarization may partially reduce the measured transport performance and could be mitigated through structural asymmetry in the membrane or system-level optimization (e.g., stirring or thermal convection) in future studies.*'

Comment 6: *The rectification discussion remains somewhat overstated.*

The authors now acknowledge that the rectification ratio is modest, which is appropriate. Nevertheless, the text still presents ion rectification as a notable characteristic, even though the measured asymmetry is weak and the mechanistic basis remains somewhat indirect. The authors should further soften this point and avoid implying that the system exhibits strong or architecturally intrinsic rectification.

Response: Following the reviewer's suggestions, we have substantially toned down the discussion of rectification, which is no longer presented as a key or intrinsic feature of the system. Instead, we now focus the analysis on the dominant charge-governed ion transport behavior. The previously discussed *I-V* asymmetry is only briefly mentioned as a minor and secondary observation, without mechanistic or performance implications.

Actions:

a) We have revised the relevant discussion in the revised manuscript on Page 7.

'*The sV2DP membrane exhibits characteristics consistent with charged nanochannels, as indicated by the asymmetric I-V response* (Supplementary Fig. 13).'

Comment 7: *Leakage control is much improved, but some doubt remains because the key evidence is mostly in the Supplementary Information.*

The added controls on intentional defects, thickness dependence, defective membranes, and post-measurement imaging considerably strengthen the manuscript, and these are among the most convincing parts of the revision. That said, because the extraordinary current and power densities are central to the paper, the main text should more clearly emphasize the most important leakage-sensitive controls. The current presentation still leaves too much of the validation burden in the Supplementary Information.

Response: We agree that, given the central importance of the reported current and power densities, the most critical leakage-sensitive controls should be more clearly emphasized in the main text. In the revised manuscript, we have therefore strengthened the presentation of these key control experiments to improve visibility and clarity. In particular, we highlight the most direct evidence from (i) intentional defect-introduction, (ii) thickness-dependent transport measurements, and (iii) post-measurement imaging of the working area, all of which collectively support the integrity of the membrane and rule out significant leakage contributions.

Actions:

a) In the revised manuscript on Page 9, we have added:

'*To elucidate the dominant ion-transport pathway, we performed a series of leakage-sensitive control experiments, including an intentional nanoscale notch via focused ion beam (FIB), thickness-dependent measurements, post-measurement imaging, and pH-dependent selectivity tests (Supplementary Figs. 23-26). The intentional-defect experiment shows that introducing a nanoscale notch leads to transport behavior approaching that of the bare substrate, accompanied by a pronounced loss of ion selectivity, consistent with leakage-dominated transport (Supplementary Fig. 23). In contrast, intact membranes exhibit significantly lower conductance and preserved selectivity. Thickness-dependent measurements further reveal*

consistent trends in V_{oc}, I_{sc}, and P_{max} across membranes with different thicknesses, supporting that ion transport is governed by the intrinsic nanochannel structure rather than uncontrolled leakage pathways (Supplementary Fig. 24). In addition, pre- and post-measurement optical and SEM imaging confirm that the membranes remain structurally intact during operation, excluding failure-induced leakage (Supplementary Fig. 25). The pH-dependent selectivity measurements further demonstrate stable ion-selective transport behavior across different conditions (Supplementary Fig. 26). Overall, these results suggest that transport in intact devices is primarily governed by the designed sV2DP nanochannels rather than by leakage pathways.'

Comment 8: *The scale-up discussion is improved but still partly inferential.*
The revised manuscript now provides a more credible explanation for the performance drop from micro-aperture devices to the array format, including resistance partitioning and the role of grain boundaries and defects. This is a significant improvement. However, the resistance decomposition is still not directly measured in a fully quantitative way, and the array-device statistics remain limited. The explanation is plausible, but the authors should avoid presenting it as fully established.
Response: Thanks for the constructive suggestions. In the revised manuscript, we have modified the relevant discussion to avoid overinterpretation. Specifically, the scale-up effects are now presented as plausible and physically consistent interpretations, rather than definitive conclusions. We have also clarified that the resistance decomposition is semi-quantitative in nature and based on simplified partitioning considerations, and that the statistics for array-scale devices remain limited. Therefore, we now emphasize that the purpose of the scale-up demonstration is to illustrate the feasibility and potential of extending the membrane toward larger-area implementations, rather than to establish a fully quantitative scaling law or fully resolved resistance model.

Actions:

a) The discussion in the revised manuscript on Page 14-15 has been updated as follows:

'*We attribute this behavior to two main factors: (i) a nonlinear decline in membrane resistance with increasing active area, and (ii) the presence of intrinsic grain boundaries and defects in larger-area membranes. Based on a semi-quantitative analysis of resistance partitioning, the decrease in intrinsic membrane resistance (R_{mem}) with increasing membrane area likely*

enhances the relative contributions of reservoir and interfacial resistances, which may contribute to the observed decline in P_{out} (Supplementary Fig. 47). In addition, grain boundaries and structural defects are expected to introduce relatively open, less ion-selective transport pathways (e.g., larger-pore-area 7-membered lattice motifs) (Supplementary Fig. 48), thereby reducing the overall permselectivity. Consequently, the device performance exhibits an approximately exponential decay with increasing active area in single-aperture measurements (Supplementary Fig. 49). These interpretations are further supported by our device-level defect-sensitivity test: introducing an intentional single-pore defect causes a dramatic loss of ion selectivity (short-circuit-like behavior) (Supplementary Fig. 50). Despite this scale-up challenge, sV2DP-based devices demonstrate excellent operational stability. Under continuous operation in realistic conditions, stable output is maintained over nine days without noticeable degradation (Supplementary Fig. 51). Post-measurement structural, chemical, and device-level characterizations reveal no discernible changes compared to the pre-measurement state (Supplementary Fig. 52-54), confirming the robustness of the membrane. Overall, these results highlight a feasible pathway toward extending membrane performance to scalable, robust, and high-performance formats for OPGs, although a fully quantitative description of the scaling behavior remains an important subject for future investigation.'

Comment 9: *The simulation–experiment connection is still qualitative.*

The MD results are visually compelling and provide a plausible mechanistic picture for the proposed screw-like pathway. However, they still do not quantitatively connect to the measured conductance, selectivity, or power output. As a result, the simulations mainly support a structural interpretation rather than providing a direct validation of the transport metrics. The manuscript would benefit from explicitly stating this limitation in the main text, not only in the rebuttal.

Response: In response, we have revised the main text to explicitly clarify this limitation. We now emphasize that the MD simulations are used to elucidate the structure-induced ion transport mechanism, in particular the screw-like migration pathway, whereas the experimentally measured conductance, selectivity, and power output arise from a more complex interplay of factors, including interfacial effects, structural defects, and device-level conditions, which are not fully captured in the simulations.

Actions:

a) We have included the following discussion in the revised manuscript on Page 11.

'*It should be noted that the connection between simulation and experiment remains primarily qualitative, as the experimentally observed transport metrics (e.g., conductance, selectivity, and power output) can be further influenced by factors such as interfacial polarization, structural defects, and device-level configurations that are not fully captured in the MD models.*'

Comment 10: *Benchmarking claims appear overstated and should be corrected.*

The manuscript still frames the membrane as outperforming state-of-the-art 2DFMs using language that appears too broad relative to the current literature landscape. Even if the reported values are impressive, the comparative claims should be narrowed and carefully qualified by test conditions, device geometry, and scaling level. The benchmarking language should be revised to avoid overstatement.

Response: We thank the reviewer for raising this important point. In the revised manuscript, we have systematically moderated the benchmarking language to avoid overly broad or absolute claims. In particular, we have replaced general claims such as "outperforming state-of-the-art 2DFMs" with more qualified and context-specific descriptions, such as "placing it among the highest-performing systems" or "demonstrating competitive performance under comparable conditions".

In addition, we would like to clarify that all comparisons were made with careful consideration of key experimental parameters, including device configuration, active area, and testing conditions. These details are now more clearly summarized in Supplementary Table 6. Where appropriate, we have also highlighted the specific conditions under which the performance is achieved, rather than making generalized claims.

Actions:

a) We have revised the relevant text in the Abstract in the revised manuscript on Page 2.

'*Integration of the sV2DP membrane into osmotic power generators (OPGs) delivers an ultrahigh power output of 243 W m^{-2} under a 50-fold NaCl gradient, placing it among the highest-performing systems.*'

b) We have made consistent revisions to the corresponding discussion in the revised manuscript on Page 3 and Page 14.

'When applied in osmotic power generators (OPGs), the sV2DP membrane delivers an outstanding power output (P_{out}) of 243 W m^{-2} under a 50-fold NaCl gradient, which exceeds many previously reported values for 2DFM-based systems (< 100 W m^{-2}).' (Page 3)

'When operated under a salinity gradient between artificial seawater and river water, the integrated system achieved an impressive P_{out} of 46.7 W m^{-2}, demonstrating competitive performance relative to previously reported macroscopic OPGs under comparable testing conditions and device configurations (Supplementary Fig. 46 and Table 6).' (Page 14)

Comment 11: *Some methodological details are still not fully transparent.*

The revision adds many useful experimental details, but the measurement conditions are still not completely comprehensive, especially regarding reservoir handling and possible interfacial polarization control. Since these details can affect extracted electrokinetic and osmotic metrics, the Methods section would benefit from one final pass to ensure full clarity and consistency.

Response: Following the reviewer's suggestion, we have carefully revised the Methods section to improve both transparency and completeness of the experimental details. For instance, we have now provided more explicit descriptions of the H-cell configuration, including the reservoir volume (8.0 mL per chamber), effective solution volume (~7 mL), and electrode spacing (~25 mm). We have also clarified the electrode setup (Ag/AgCl electrodes with 3 M KCl salt bridges) used to minimize junction potentials associated with concentration differences. In addition, we have detailed the measurement protocol, including chamber rinsing procedures before each measurement, and the requirement that the V_{oc} be stabilized before data acquisition, to reduce the influence of transient interfacial polarization.

Actions:

a) We have revised the Methods section in the revised manuscript on Page 20.

'Ion transport measurements

The membrane sample was prepared as follows. An as-prepared membrane was transferred onto a freestanding 200-nm-thick SiNx film supported by a silicon wafer, covering a 2 μm-diameter aperture (Supplementary Fig. 12a). After drying at room temperature for 1 h, membrane coverage was confirmed via SEM and optical microscopy. To remove residual contaminants, the membrane/substrate assembly was sequentially rinsed with ethanol and Milli-Q water, followed by drying at 80 °C for 1 h to ensure stability and cleanliness.

All ion transport measurements were performed using a custom-built H-cell device (Supplementary Fig. 12b). The membrane/substrate assembly was clamped between two chambers (reservoir volume: 8.0 mL each), allowing contact with aqueous electrolyte solutions (~7 mL) on both sides. Standard Ag/AgCl electrodes with 3 M KCl salt bridges (CHI111) were used to record voltage and current signals, effectively eliminating junction potential caused by salt concentration differences. The electrode spacing was approximately 25 mm. KCl solutions at pH 6.7 were chosen as electrolytes due to the similar bulk diffusion coefficients of K^+ and Cl^- ions. Electrochemical tests were performed on a CHI 770E workstation to evaluate ion transport and OPG performance. All measurements were conducted at room temperature (25 ^oC), and I-V scans were recorded at a scan rate of 10 mV s^{-1}. Before each I-V measurement, both chambers were rinsed three times with ultrapure water to minimize cross-contamination. The V_{oc} was then monitored and allowed to stabilize until a steady value was reached before data acquisition, ensuring minimal influence from transient interfacial polarization (Supplementary Fig. 20).'

Point-to-point response to the comments from the reviewers

Reviewer #2

General comment. *The manuscript is now close to publishable form and, in my judgment, satisfies the core Nature Communications threshold of a significant specialist advance supported by substantial evidence. The revision has meaningfully improved methodological transparency, leakage control, interpretive honesty around η_EDL and the simulation work, and the treatment of scale-up limitations. I do not see a remaining flaw that fatally undermines the central empirical result that the sV2DP membrane exhibits strong anion-selective transport and competitive osmotic-power performance.*

Response: We sincerely thank the reviewer for the positive evaluation of our revised manuscript and for recognizing the improvements. We have carefully considered the remaining comments and have addressed them point by point below. Corresponding revisions have been incorporated into the revised manuscript where appropriate.

Comment 1: *That said, I would not recommend unconditional acceptance in its current wording. Before publication, the authors should make the final version fully internally consistent and more restrained in three places: first, everywhere label Σ as effective/apparent and t-based selectivity metrics as inferred where appropriate.*

Response: We appreciate the reviewer for this constructive and helpful suggestion. We have revised the manuscript and Supplementary Information accordingly. Specifically, we have added "effective" before the measured/fitted surface charge density *Σ* (where appropriate), and "inferred" before the transference number *t* and related *t*-based selectivity metrics (where appropriate).

Actions:

a) We have added "effective" or "apparent" before the measured/fitted *Σ* values, as follows:

'*The sV2DP is fully crystalline, exhibiting face-on orientation with through-plane triangular nanochannels (D_{eff}: ~1.36 nm) and strongly positively-charged surfaces (effective Σ: +22.4 mC m^{-2}).*' (On Page 3)

'*By fitting a constant-charge model, an effective (or apparent) fitted surface charge density (Σ) of sV2DP is further estimated to be +22.4 mC m^{-2} (Supplementary Note 3),*' (On Page 6)

'This amplified surface charge effect is consistent with the observed high effective Σ of the resulting sV2DP membranes' (On Page 9)

'Computational modeling and experimental investigations indicate that its ultranarrow nanochannels (D_{eff} = 1.36 nm) and strongly charged channel surface (effective Σ, +22.4 mC m^{-2}) in sV2DP' (On Page 14)

'The red curve shows the theoretical fit using a constant-charge model (effective Σ = +22.4 mC m^{-2} in 0.25 M KCl).' (On Page 25)

b) We have revised the descriptions of the transference number t and related t-based selectivity metrics by adding "inferred" where appropriate:

'This structural arrangement increases EDL coverage and enables efficient anion transport, giving an inferred chloride transference number of 0.85' (On Page 1)

'This structural configuration promotes EDL overlap contents, leading to a high inferred chloride transference number (t_{Cl^-}: ~0.85) and an ultrahigh net chloride flux' (On Page 3)

'Based on measured V_{oc} values, the sV2DP membrane maintains the inferred Cl^- selectivity coefficients (S_{Cl^-}) above 50% across all tested C_s gradients,' (On Page 8)

'Its inferred S_{Cl^-} drops to just 21.6% under a 50-fold gradient, less than one-third of the value observed for the sV2DP membrane (Supplementary Fig. 32).' (On Page 8)

'The inferred t_{Cl^-} reaches ~ 0.90, surpassing that under the same gradient of KCl (Supplementary Fig. 43).' (On Page 10)

'As a result, sV2DP achieves a high inferred t_{Cl^-} of 0.85 and ultrahigh Cl^- flux (current output of 14.6 kA m^{-2}) under a 50-fold KCl gradient.' (On Page 15)

'For a conservative estimate and to facilitate direct comparison with prior reports, the concentration ratio (C_{high}/C_{low}) is used as an approximation of a_{high}/a_{low} to obtained the inferred S values in this work. Additionally, the inferred t_- is calculated as[57]:'(On Page 18)

*'**h**, Inferred t_{Cl^-}, and **i**, P_{max}, respectively. Individual data points are shown alongside the box plots, with half-violin plots indicating the data distribution.'* (On Page 26)

*'P_{out} is plotted as a function of the inferred t values and EIR (reflecting area-normalized ion conductance). **d**, Inferred t_{Cl^-} of sV2DP membranes as a function of the cation diffusion coefficient.'* (On Page 26)

Comment 2: *Second, explicitly acknowledge that the experimental sV2DP versus es-V2DP comparison is not a pure stacking-only control because chemistry/linkage differences remain;*

Response: We thank the reviewer for this helpful suggestion. We agree that the experimental comparison between sV2DP and eV2DP should not be considered a pure stacking-only control, because chemistry/linkage differences remain between the two membranes. We have now explicitly acknowledged this point in the main text. At the same time, we clarify that MD simulations using the corresponding structural models support the view that the distinct channel geometry and spatial distribution of fixed pyridinium charges play a major role in regulating Cl^- migration pathways. Accordingly, we have revised the wording to present this comparison more cautiously and to avoid attributing the observed differences solely to stacking.

Actions:

a) We have added a statement in the main text to explicitly acknowledge that the comparison between sV2DP and eV2DP, as follows:

'Although the experimental comparison between sV2DP and es-V2DP is not a pure stacking-only control because chemistry/linkage differences remain, MD simulations using the corresponding structural models support the view that the distinct channel geometry and spatial distribution of fixed pyridinium charges play a major role in regulating Cl^- *migration pathways.'* (On Page 9)

Comment 3: *Third, further narrow benchmarking and practical-performance claims so the distinction between micro-aperture peak power density and larger-area/device-level output is unmistakable.*

Response: We thank the reviewer for pointing out this important issue. We have further restrained the benchmarking and practical-performance claims in the revised manuscript. We now clearly distinguish the peak power density obtained from single micro-aperture OPGs from the larger-area aperture-array/device-level output, to avoid any overgeneralization of practical performance.

Actions:

a) We have revised the related text to avoid overgeneralizing the practical performance, as follows:

'When integrated into micro-aperture osmotic power generators, the sV2DP membrane delivered a peak power density of 243 W m^{-2} under a 50-fold NaCl gradient.' (On Page 2)

'When applied in single micro-aperture osmotic power generators (OPGs), the sV2DP membrane delivers a high-power output (P_{out} *) of 243 W* m^{-2} *under a 50-fold NaCl gradient,*

which exceeds many previously reported values for 2DFM-based systems (< 100 W m^{-2}). Owing to its high mechanical robustness, the ultrathin sV2DP membrane was successfully integrated into an aperture-array OPG with a total aperture area of 6358.5 μm^2 and sustained stable power generation over nine days, suggesting its potential for scaled-area operation and durability.' (On Page 3)

'We then evaluated osmotic energy harvesting by integrating the resulting sV2DP membrane into a single micro-aperture OPG under a 50-fold NaCl gradient (Fig. 4a).' (On Page 10)

'When operated under an artificial seawater/river water salinity gradient, the resulting aperture-array OPG delivered a P_{out} of 46.7 W m^{-2}.' (On Page 12)

'When integrated into a single micro-aperture OPG, the sV2DP membrane delivers a peak power density of 243 W m^{-2} under a 50-fold NaCl gradient. In an aperture-array OPG with a total aperture area of 6,358.5 μm^2, the membrane sustains a P_{out} of 46.7 W m^{-2} over nine days under artificial seawater/river water conditions, suggesting its potential for scaled-area operation and long-term durability, suggesting its potential for scaled-area operation and long-term durability.' (On Page 15)

Comment 4: *I would also require a final editorial pass over figure captions, typos, and data/code availability wording.*

Response: Thanks for this helpful suggestion. We have carefully performed a final editorial check of the revised manuscript, including figure captions, typographical errors, and the data/code availability statements. The relevant wording has been corrected and standardized where appropriate.

Actions:

a) We have carefully checked and corrected typographical errors throughout the manuscript and Supplementary Information. For example, we corrected errors such as "pridinium" to "pyridinium, "Fig.1a" to "Fig. 1a," and standardized spacing in units and symbols, such as "300kV" to "300 kV" and "50°C" to "50 °C."

b) We have revised and standardized the figure captions where appropriate to ensure consistency with the main text. For example, we replaced "insert" with "inset," standardized η_{EDL}, P_{out}, P_{max}, formatting, added "effective/apparent Σ" and "inferred t" where appropriate, and consistently distinguished single micro-aperture OPGs from aperture-array OPGs in the figure captions.

c) We have checked and updated the *Data Availability* and *Code Availability* statements to ensure that the wording is accurate and clear.
